# Piperaquine-resistant PfCRT mutations differentially impact drug transport, hemoglobin catabolism and parasite physiology in *Plasmodium falciparum* asexual blood stages

**DOI:** 10.1371/journal.ppat.1010926

**Published:** 2022-10-28

**Authors:** John Okombo, Sachel Mok, Tarrick Qahash, Tomas Yeo, Jade Bath, Lindsey M. Orchard, Edward Owens, Imhoi Koo, Istvan Albert, Manuel Llinás, David A. Fidock

**Affiliations:** 1 Department of Microbiology and Immunology, Columbia University Irving Medical Center, New York, New York, United States of America; 2 Center for Malaria Therapeutics and Antimicrobial Resistance, Division of Infectious Diseases, Department of Medicine, Columbia University Irving Medical Center, New York, New York, United States of America; 3 Department of Biochemistry & Molecular Biology, The Pennsylvania State University, University Park, Pennsylvania, United States of America; 4 Huck Center for Malaria Research, The Pennsylvania State University, University Park, Pennsylvania, United States of America; 5 Department of Veterinary and Biomedical Sciences, The Pennsylvania State University, University Park, Pennsylvania, United States of America; 6 The Huck Institutes of the Life Sciences, The Pennsylvania State University, University Park, Pennsylvania, United States of America; 7 Department of Chemistry, The Pennsylvania State University, University Park, Pennsylvania, United States of America; University of South Florida, UNITED STATES

## Abstract

The emergence of *Plasmodium falciparum* parasite resistance to dihydroartemisinin + piperaquine (PPQ) in Southeast Asia threatens plans to increase the global use of this first-line antimalarial combination. High-level PPQ resistance appears to be mediated primarily by novel mutations in the *P*. *falciparum* chloroquine resistance transporter (PfCRT), which enhance parasite survival at high PPQ concentrations *in vitro* and increase the risk of dihydroartemisinin + PPQ treatment failure in patients. Using isogenic Dd2 parasites expressing contemporary *pfcrt* alleles with differential *in vitro* PPQ susceptibilities, we herein characterize the molecular and physiological adaptations that define PPQ resistance *in vitro*. Using drug uptake and cellular heme fractionation assays we report that the F145I, M343L, and G353V PfCRT mutations differentially impact PPQ and chloroquine efflux. These mutations also modulate proteolytic degradation of host hemoglobin and the chemical inactivation of reactive heme species. Peptidomic analyses reveal significantly higher accumulation of putative hemoglobin-derived peptides in the PPQ-resistant mutant PfCRT isoforms compared to parental PPQ-sensitive Dd2. Joint transcriptomic and metabolomic profiling of late trophozoites from PPQ-resistant or -sensitive isogenic lines reveals differential expression of genes involved in protein translation and cellular metabolism. PPQ-resistant parasites also show increased susceptibility to an inhibitor of the *P*. *falciparum* M17 aminopeptidase that operates on short globin-derived peptides. These results reveal unique physiological changes caused by the gain of PPQ resistance and highlight the potential therapeutic value of targeting peptide metabolism in *P*. *falciparum*.

## Author summary

The emergence of multidrug resistance in *Plasmodium falciparum* parasites presents a significant obstacle to the malaria elimination agenda. Resistance to piperaquine (PPQ), an important first-line partner drug, has spread across Southeast Asia where it has contributed to widespread treatment failures. The genetic basis of high-level resistance to PPQ is primarily attributable to a novel set of amino acid substitutions in the *P*. *falciparum* chloroquine resistance transporter (PfCRT). Using genetically-modified parasite lines, we show that PPQ-resistant asexual blood stage parasites only modestly reduce the intracellular concentration of PPQ, in contrast with the related drug chloroquine where resistant forms of PfCRT lead to minimal accumulation. We also report that PPQ-resistant, PfCRT mutant parasites accumulate higher levels of putative hemoglobin-derived peptides than do their PPQ-sensitive counterparts. These two groups of parasites exhibit differential expression of genes involved in multiple processes including protein translation and phosphorylation, cellular metabolism, and RNA processing. We also observed increased susceptibility of the PPQ-resistant mutant PfCRT parasites to inhibition of an enzyme involved in cleaving globin-derived peptides, supporting the hypothesis that PfCRT mutations interfere with the vital process of hemoglobin catabolism.

## Introduction

Malaria continues to exert a massive global impact, with an estimated 241 million cases and 627,000 deaths in 2020, mostly in sub-Saharan Africa [1]. Artemisinin (ART)-based combination therapies (ACTs), which partner fast-acting yet short-lived ART derivatives with longer-lasting drugs such as piperaquine (PPQ), constitute first-line treatments worldwide [2]. A major threat to their efficacy is the emergence and dissemination of drug-resistant *Plasmodium falciparum* parasites [3–5].

PPQ is characterized by a plasma half-life of 14 to 23 days, thereby providing post-treatment prophylaxis in addition to curing active infection; a good safety profile in children and pregnant women; and potent activity against chloroquine (CQ)-resistant parasites [6–8]. PPQ plus dihydroartemisinin (DHA) shows promise in Africa for chemoprevention programs [9,10]. DHA+PPQ plus mefloquine as a triple ACT also provides effective treatment and aims to delay the emergence of multidrug resistance [11]. Reports of widespread treatment failures with DHA+PPQ in SE Asia [4,12–14] highlight the need to better characterize the physiological adaptations in *P*. *falciparum* that track with PPQ resistance (PPQR).

PPQ comprises two CQ-like 4-aminoquinoline weak-base moieties and accumulates in the parasite’s digestive vacuole (DV) as a poly-protonated species. PPQ, like CQ, is thought to exert its antimalarial action primarily by binding to Fe(III)-heme that is released during hemoglobin (Hb) degradation in the DV, thus preventing the incorporation of toxic heme into chemically inert hemozoin (Hz) crystals [15]. PPQR *in vitro* is characterized by atypical bimodal dose-response profiles with elevated IC_90_ values that correlate with higher levels of parasite survival (≥10%) in the PPQ survival assay (PSA) [16–20].

Recent studies of PPQR have revealed a key role for the *P*. *falciparum* chloroquine resistance transporter (PfCRT), a 49-kDa member of the drug/metabolite transporter superfamily that localizes to the DV membrane of asexual blood stage (ABS) parasites [21]. Several mutant isoforms of PfCRT are known to mediate CQ resistance (CQR) by permitting the efflux of this drug away from its heme target in the DV [22–28]. These isoforms include Dd2, predominant in SE Asia and containing eight point mutations in PfCRT compared with the wild-type (WT) 3D7 isoform (**Table 1**). In the case of PPQR, additional PfCRT mutations have arisen in the Dd2 isoform, including T93S, H97Y, F145I, I218F, M343L and G353V that have been associated with PPQR or DHA+PPQ treatment failure [14,16,29–31]. A PfCRT C350R mutation has also been observed in PPQ-resistant South American parasites [32]. Gene editing studies show that these novel PfCRT mutations can cause a range of PPQ resistance levels in *P*. *falciparum* parasites [15,20,29,32,33]. Interestingly, several of these PPQ-resistant mutations cause a partial or total loss of CQR [15,34]. Amplification of the plasmepsin II and III hemoglobinases, which may accelerate Hb degradation or heme incorporation into Hz, has also been identified as a marker of PPQR in field isolates [14,17,18,30,35,36].

**Table 1:** PfCRT haplotypes and PPQ and CQ phenotypes of lines used herein.

	PfCRT Haplotypes												*pm2/3*	*pfmdr1*	Phenotype	
Parasite Line	74	75	76	97	145	220	271	326	343	353	356	371	Copies	Copies	PPQ	CQ
Dd2	I	E	T	H	F	S	E	S	M	G	T	I	1	2	S	R
Dd2^Dd2crt^	I	E	T	H	F	S	E	S	M	G	T	I	1	2	S	R
Dd2^F145Icrt^	I	E	T	H	I	S	E	S	M	G	T	I	1	2	R^high^	S
Dd2^M343Lcrt^	I	E	T	H	F	S	E	S	L	G	T	I	1	2	R^low^	S
Dd2^G353Vcrt^	I	E	T	H	F	S	E	S	M	V	T	I	1	2	R^mod^	S
Dd2^3D7crt^	M	N	K	H	F	A	Q	N	M	G	I	R	1	2	S	S
RF7	I	E	T	H	F	S	E	S	L	G	T	I	3	1	R^high^	R
RF12	I	E	T	Y	F	S	E	S	M	G	T	I	2	1	R^high^	R

All parasite lines were cloned prior to use (Dd2-B2 was used for Dd2). RF7 and RF12 carry the K13 C580Y mutation that confers *in vitro* resistance to artemisinin derivatives. CQ, chloroquine; mod, moderate; *pm2/3*, plasmepsins 2 and 3; *pfmdr1*, *P*. *falciparum* multidrug resistance protein 1; PPQ, piperaquine.

In this study, we leveraged a panel of Dd2-based isogenic PPQ-sensitive and PPQ-resistant parasites (**Table 1**) to further characterize PPQR. Dd2^Dd2crt^ and Dd2^3D7crt^ parasites constituted the PPQ-sensitive pair with <1% PSA values while lines expressing Dd2 PfCRT M343L (Dd2^M343Lcrt^), G353V (Dd2^G353Vcrt^) and F145I (Dd2^F145Icrt^) alleles comprised the PPQ-resistant set with mean PSA values of between 12 to 25% [20]. These latter three lines acquired PPQR with only a single copy of plasmepsins II and III. Using these lines, we present data on the impact of mutant PfCRT on PPQ transport, Hb catabolism, drug-heme interactions, metabolomics, peptidomics, and transcriptomics. Our data reveal a broad impact of PPQR-conferring PfCRT mutations on *P*. *falciparum* intracellular physiology and highlight the potential of targeting amino acid metabolism in PPQ-resistant parasites.

## Results

### Novel PfCRT mutations confer differential PPQ and CQ cellular accumulation profiles

**Fig 1:**
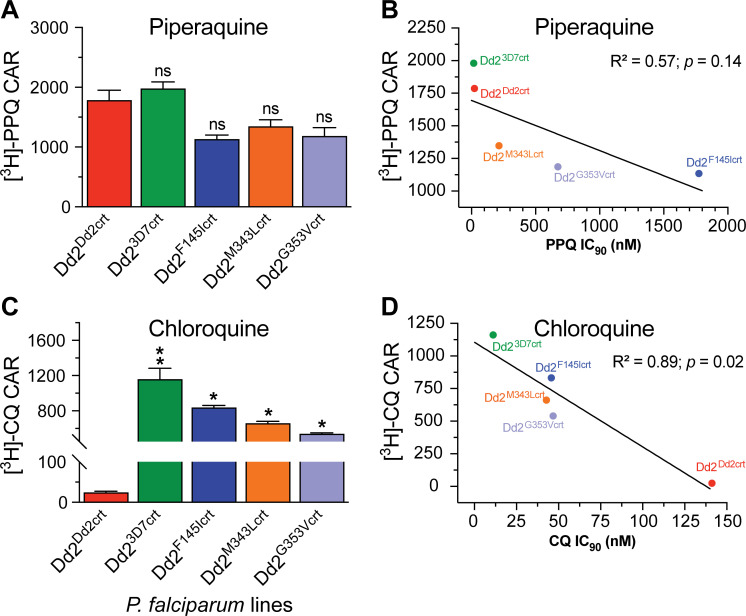
*P*. *falciparum* mutant PfCRT-mediated resistance to piperaquine (PPQ), unlike the related 4-aminoquinoline chloroquine (CQ), cannot be explained solely by reduced intracellular drug accumulation. Cellular accumulation ratios (CAR) represent the ratio of intracellular to extracellular **(A)** [^3^H]-PPQ and **(C**) [^3^H]-CQ and were measured after 1 hr incubation at 37°C. Results were plotted against **(B)** PPQ and **(D)** CQ *in vitro* antiplasmodial activities as indexed by their 90% growth inhibitory concentrations (IC_90_). Assays employed synchronized trophozoites, when the DV is fully formed and Hb degradation is maximal. CAR, IC_50_ and IC_90_ results for each line are presented as the mean ± SEM value (4 to 5 independent experiments with technical duplicates; see **S1 Table** and raw data in **S1 Data**). Statistical significance was determined using Mann–Whitney *U* tests comparing the *pfcrt*-edited lines with their isogenic Dd2^Dd2crt^ parent. *p<0.05; **p<0.01; ns; not significant.

CQR *in vitro* is generally attributed to diminished drug accumulation in the parasite DV due to mutant PfCRT-mediated CQ efflux [24]. Here, we investigated whether the PfCRT F145I, M343L and G353V mutations, which evolved in SE Asia as individual PPQR-conferring mutations added to the PfCRT CQ-resistant Dd2 isoform, confer a comparable influence on parasite intracellular accumulation of PPQ. We therefore measured the steady-state uptake of [^3^H]-PPQ in synchronized trophozoites of the mutant lines Dd2^F145Icrt^, Dd2^M343Lcrt^ and Dd2^G353Vcrt^, and compared these findings against isogenic Dd2^Dd2crt^ and Dd2^3D7crt^ lines (expressing the PfCRT Dd2 and the CQS 3D7 WT isoform, respectively). These studies employed 20 nM [^3^H]-PPQ, with parallel assays using 5 nM [^3^H]-CQ as the comparator. These concentrations are within the range of prior published studies [20,22,37–40]. From these data, we derived the cellular accumulation ratio (CAR), defined as the ratio of intracellular versus extracellular drug. Dd2^3D7crt^ showed the highest [^3^H]-PPQ accumulation ratio (mean CAR of 1,980; **Fig 1A**). However, this value did not differ substantially from the level of accumulation observed in the PPQ-sensitive Dd2^Dd2crt^ line or the three PPQ-resistant mutant lines (Dd2^F145Icrt^, Dd2^M343Lcrt^ and Dd2^G353Vcrt^; **Fig 1A**). At this low PPQ concentration, Dd2^Dd2crt^ only accumulated 1.6-fold more drug compared to the most PPQ-resistant line, Dd2^F145Icrt^ (**Fig 1A** and **S1 Table**). Although we observed a positive correlation between [^3^H]-PPQ uptake and *in vitro* antiplasmodial activity, this association was borderline and not statistically significant (*R*^2^ = 0.57; *p*>0.05) suggesting that the mechanism of mutant PfCRT-mediated PPQR cannot be explained solely by a simple drug transport model. These results also hint that PPQ transport is likely only discernible at higher concentrations. This absence of a clear association between intracellular accumulation and *in vitro* sensitivity suggests a complex interaction between these PfCRT mutations and PPQR and a possible contribution of other physiological phenomena.

For CQ, we observed a strong correlation between levels of [^3^H-CQ] accumulation and *in vitro* sensitivity across lines (R^2^ = 0.89; *p* = 0.02), with the CQ-resistant Dd2^Dd2crt^ showing 21 to 47 fold lower levels of CQ accumulation relative to the CQ-sensitive Dd2^3D7crt^ line and the Dd2^F145Icrt^, Dd2^M343Lcrt^ and Dd2^G353Vcrt^ lines. For the latter three lines the gain of PPQR was accompanied by substantial resensitization to CQ (**Fig 1B** and **1D and S1 Table**).

### Piperaquine-resistant PfCRT mutations are associated with different levels of intracellular total heme and undigested hemoglobin

Hb endocytosis into the DV and its subsequent proteolytic digestion liberates free ferrous (Fe^2+^) heme, which is rapidly oxidized to its insoluble ferric (Fe^3+^) form and then biomineralized into inert Hz crystals to shield the parasite from the oxidative cytotoxicity of free heme [41]. Based on a prior report associating CQR with an excessive buildup of putative globin-derived peptides [42], we speculated that PPQR may also track with defective Hb catabolism in the parasite. To explore this hypothesis, we leveraged the pyridine-based heme fractionation protocol [43] to examine the baseline levels of total subcellular heme-iron (heme-Fe) in all lines and quantify the constituent species present as Hb (in its undigested form), ‘free’ heme-Fe (the labile redox-active form) and Hz (the alkali-soluble crystalline form into which the bulk of total heme is incorporated). These assays were conducted with extracts of parasites obtained from saponin-lysed and washed *P*. *falciparum*-infected RBCs and thus represent heme-Fe levels only in parasites (and not the global content of parasites and the host RBCs).

The spectrophotometrically-determined sum of all heme species in parasites was noted to be higher in Dd2^3D7crt^ compared to Dd2^Dd2crt^ (61.3 ± 0.4 vs 56.5 ± 0.7 fg/cell, *p* = 0.03; **S2 Table**). These values are consistent with previously reported total amounts of 58 ± 2 fg/cell in trophozoites extracted from the CQ-sensitive D10 strain [44]. Control assays with Dd2^Dd2crt^ cultures measured total heme-Fe concentrations of 97.5 ± 1.4 fg/infected RBC, indicating that ~58% of the total heme-Fe measured in Dd2^Dd2crt^ infected RBCs was observed in the parasite fraction. This value closely matches an earlier estimation of 61% [44]. We also measured a mean heme-Fe concentration in uninfected RBCs of 104.1 ± 1.1 fg/cell (**S2 Table**).

**Fig 2:**
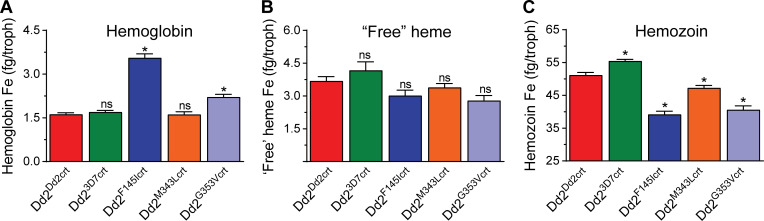
The highly PPQ-resistant lines Dd2^F145Icrt^ and Dd2^G353Vcrt^ display reduced levels of hemoglobin (Hb) proteolysis and hemozoin (Hz) formation. Amounts of **(A)** Hb, **(B)** “free” heme-Fe and **(C**) Hz measured spectrophotometrically and expressed in femtogram (fg) per trophozoite (“troph”) purified from PPQ-sensitive and PPQ-resistant lines. Bar graphs indicate mean ± SEM absolute amounts of each species for each line, calculated from four independent experiments with technical duplicates. For each parasite line, values obtained were compared against those of the Dd2^Dd2crt^ parental control (red bars) and statistical significance was calculated using Mann Whitney *U* tests (*p<0.05).

Our parasite assays identified significantly lower levels of total heme species in all the PfCRT mutant PPQ-resistant lines compared to Dd2^Dd2crt^, with total amounts in the Dd2^F145Icrt^ and Dd2^G353Vcrt^ mutants the lowest at 45.7 ± 1.1 and 45.6 ± 1.4 fg/infected red blood cell (RBC), respectively (**S2 Table**). Interestingly, the mean ± SEM basal levels of undigested Hb in parasites were significantly higher in the highly PPQ-resistant lines Dd2^F145Icrt^ (3.6 ± 0.1 fg/cell) and Dd2^G353Vcrt^ (2.1 ± 0.1 fg/cell) compared to the Dd2^M343Lcrt^ line that showed lower-grade PPQR and the PPQ-sensitive lines Dd2^Dd2crt^ and Dd2^3D7crt^ (the latter three yielded values of 1.6 to 1.7 ± 0.1 fg/cell) (**Fig 2A** and **S2 Table**). These amounts of undigested Hb in Dd2^F145Icrt^ and Dd2^G353Vcrt^ represent 7.9% and 4.6%, respectively, of total heme species compared to 2.8% to 3.1% in Dd2^M343Lcrt^, Dd2^Dd2crt^, and Dd2^3D7crt^. These findings align with previously published parasite fitness results [20] and suggest less effective Hb catabolism in the PfCRT mutant PPQ-resistant lines, Dd2^F145Icrt^ and Dd2^G353Vcrt^. There were, however, no significant differences in the levels of ‘free’ heme-Fe between the PPQ-sensitive and PPQ-resistant lines (**Fig 2B** and **S2 Table**), suggesting that these parasites retain a competent heme detoxification machinery irrespective of their PPQ phenotype. The proportion of highly toxic labile heme-Fe that can interact with other macromolecules was maintained at between 6 to 7% of total Hb digested. In contrast, Hz was significantly higher in Dd2^3D7crt^ compared to Dd2^Dd2crt^ (55.4 ± 0.5 vs 51.2 ± 0.8 fg/cell, *p* = 0.03; **Fig 2C and S2 Table**). Hz levels were lowest in Dd2^F145Icrt^ (39.1 ± 1 fg/cell) and Dd2^G353Vcrt^ (40.6 ± 1.2 fg/cell) relative to Dd2^Dd2crt^, likely a downstream reflection of their defective breakdown of internalized Hb.

### PfCRT mutant PPQ-resistant lines accumulate higher levels of short Hb-derived peptides

**Fig 3:**
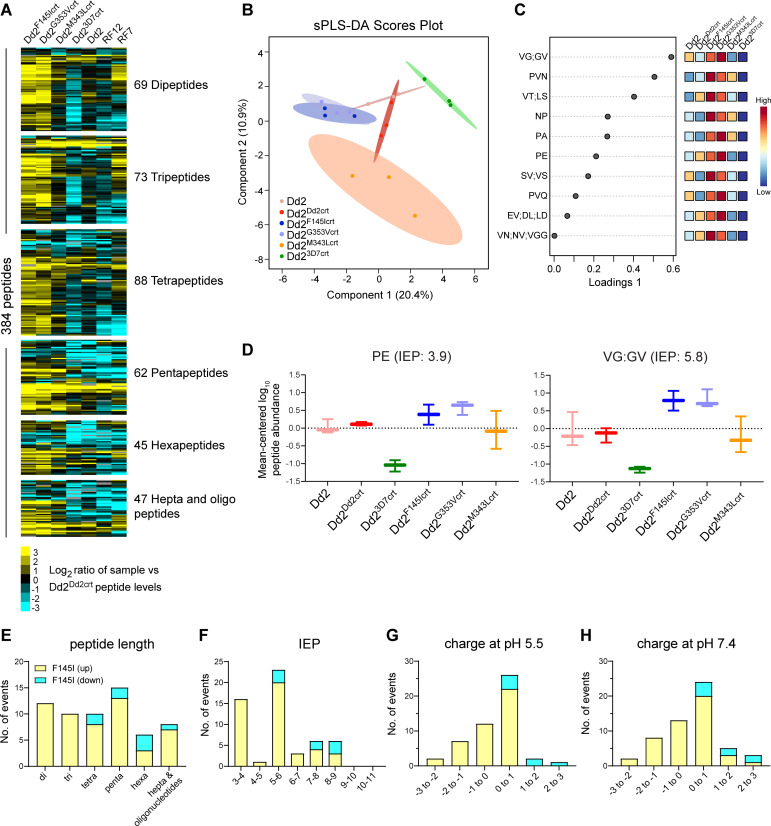
PPQ-resistant and PPQ-sensitive parasites differ in their intracellular repertoire of hemoglobin-derived peptides. **(A)** Heatmap of 384 peptides (detected in both modes and present in at least 6 out of 7 parasite lines), classified by peptide lengths, showing differences in the accumulation of peptides computed from log_2_ fold changes in the average abundance of each peptide identified from each line compared to Dd2^Dd2crt^ (N, n = 3, 3). Hepta and oligo refers to peptides ≥ 7 amino acids in length. **(B)** sparse Partial least-squares discriminant analysis (sPLS-DA) of mean-centered log_10_-transformed peptide abundance, showing that parasite lines cluster as groups separated by their CQ and PPQ phenotypes. Points represent individual sample replicates while the 95% confidence interval is represented by the shaded region. **(C)** Plot showing the top 10 variables selected by the sPLS-DA model for Component 1 (listed as Loadings 1). The variables are ranked by the absolute values of their loadings. Heat maps represent the relative abundance of each metabolite, with red and blue showing high and low levels, respectively. **(D)** Box and whisker plots showing mean-centered log_10_ abundance of select peptides that show a significant difference between the two PPQ-R Dd2^F145Icrt^ and Dd2^G353Vcrt^ lines and the others. (**E-H**) Characteristics of peptides whose abundance differs significantly between Dd2^F145Icrt^ and Dd2^Dd2crt^. Differences are shown for (**E**) peptide lengths, (**F**) isoelectric points (IEP), (**G**) peptide charge at pH 5.5, and (**H**) peptide charge at pH 7.4. Details are provided in **S4 Table**, which also lists Dd2^G353Vcrt^ and Dd2^M343Lcrt^ each vs. Dd2^Dd2crt^.

PfCRT is localized on the membrane of the DV within which the majority of Hb metabolism occurs [21]. Given the differential levels of Hb demonstrated above, the growth defects in the F145I and G353V mutant parasites [20], and the atypical distended DV phenotype observed in these lines, we hypothesized that these PfCRT mutations may also impact Hb processing. To this end, we conducted peptidomic analyses to examine the populations of peptides present in the different lines. This analysis identified a total of 393 putative endogenous Hb-derived peptides in both positive and negative mode (**S3 Table**), a comparable abundance to the 362 earlier observed in a metabolic quantitative trait loci analysis that associated CQR with impaired Hb catabolism [42]. These peptides differed in charge and length (dipeptides to 13-mers) and could be mapped to either the α or β chains of Hb (**S2 Fig**) while appearing to exist in overlapping sequence-related clusters. This observation is consistent with a hemoglobinase-mediated semi-ordered Hb digestion cascade involving proteases and aminopeptidases [45]. We observed higher levels of peptides in CQ-resistant parasites expressing the Dd2 PfCRT isoform compared to the isogenic CQ-sensitive parasite line expressing WT PfCRT (**Figs 3A and** S1A), in agreement with earlier studies [42].

Our results also reveal that the PPQ-resistant parasites harboring the F145I, M343L and G353V mutations on a Dd2 PfCRT isoform accrue higher levels of specific peptides relative to Dd2^Dd2crt^. This was evidenced by increased peptide abundance (mostly of 2–5 amino acids in length) in more than half of the total number of detected peptides (**Figs 3A** and S1B). These data, for the first time, link PPQR with excess accumulation of short peptides in PfCRT mutants. We also noted a significant logarithmic difference in the mean level of Hb-derived peptides in the PPQ-resistant clinical isolate RF7 (that contains the Dd2+M343L PfCRT isoform as well as three copies of plasmepsins II and III; **Table 1** and **Fig 3A**). Collectively, these results reveal that the additional PPQR-conferring F145I and G353V mutations on the Dd2 PfCRT isoform led to the accumulation of peptides to a larger extent than between the PPQ-sensitive Dd2 and WT isoforms. These isoform-specific differences likely reflect their differential location in PfCRT, with F145I and G353V lining the inner central cavity on the DV side through which peptides and drugs are suspected to move, as opposed to M343L that is positioned on the cytosolic side (**S3 Fig**).

Using sparse partial least-squares discriminant analysis, we identified discriminatory sets of peptides that clustered PPQ-resistant and PPQ-sensitive lines into different groups (**Fig 3B** and **3C**). We observed that these were predominantly di- and tri-peptides including PE and LD (**Fig 3C–3E** and **S4 Table**), which were previously associated with mutant *pfcrt* alleles and CQR [42]. These differences were most notable for the Dd2^F145Icrt^ line. Among longer peptides, we also observed DPVNF, a truncated version of VDPVNF that was identified to be a putative PfCRT substrate in [^3^H]-CQ *cis*-inhibition experiments in the *Xenopus laevis* oocyte model system [46]. Overall, the peptidomic analysis identified 61 and 7 peptides that showed significant differences in accumulation between the PPQ-resistant PfCRT mutant Dd2^F145Icrt^ and Dd2^G353Vcrt^ lines, respectively, compared to PPQ-sensitive Dd2^Dd2crt^. No significant differences were observed between Dd2^M343Lcrt^ and Dd2^Dd2crt^ (**Fig 3A** and **S4 Table**). Further analysis of the 61 peptides that differed significantly between Dd2^F145Icrt^ and Dd2^Dd2crt^ showed isoelectric points (IEP) of 3–6. These peptides were mostly neutral or had a single positive or negative charge at pH values of 5.5 or 7.4 (**Fig 3F–3H**). These properties are consistent with a recent study showing accumulation of short mostly neutral peptides in a Dd2 PfCRT conditional knockdown line [47].

Mapping of the peptides, derived from analysis of log_2_ differences between Dd2^3D7crt^ and either Dd2^F145Icrt^ or Dd2^G353Vcrt^, onto the α or β chains of Hb revealed the Hb sequence positions with the greatest differences in accumulation (**S2 Fig**). These findings hint at a potential disparity in enzyme cut sites between the two lines. These differences were generally not seen at the major known Hb cleavage sites, suggesting that the initial proteolysis was not affected but that the downstream digestion or transport of these peptides was impaired in Dd2^F145Icrt^ parasites, leading to an overall increased accumulation relative to Dd2^3D7crt^.

### Targeted metabolomics reveals altered levels of amino acid-related metabolites in PPQ-resistant lines

The rapid expansion of parasite biomass during ABS growth is facilitated by the breakdown and assimilation of essential nutrients including purines, glucose, vitamins, and amino acids that are either derived from catabolism of Hb or are directly scavenged from host RBCs. Parasites show some capacity to metabolically adapt in response to deprivation of such nutrients [48,49]. Studies with drug pressure have also revealed metabolic changes in ART-resistant K13-mutant parasites, presumably to accommodate altered levels of K13 functionality and enhance parasite survival [50]. We therefore posited that contrasting the metabolomes of PfCRT mutant parasites with their isogenic WT parental line can offer clues into pathways and processes utilized by parasites in the presence of PPQR-conferring PfCRT mutations.

**Fig 4:**
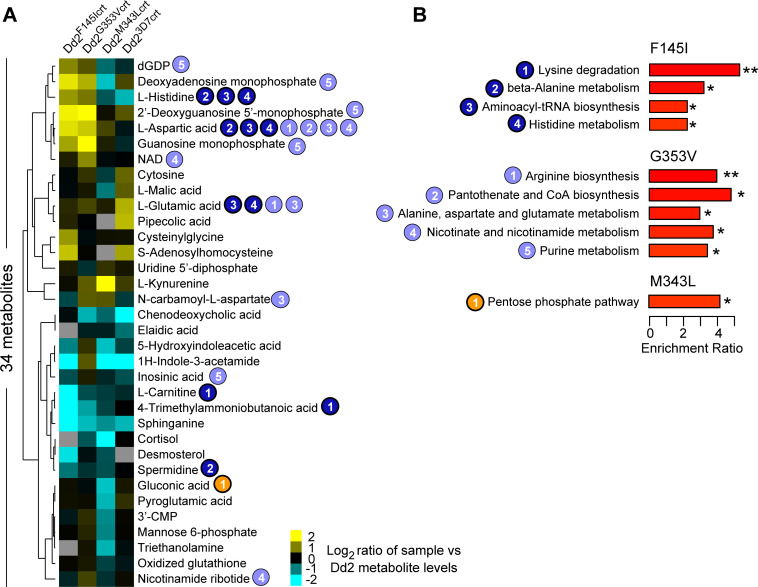
Detected metabolites and pathways altered in PPQ-resistant mutant *pfcrt* lines. (**A**) Heatmap of 34 metabolites identified in at least 3 of the 4 isogenic Dd2 lines differing in their PfCRT isoform, with metabolite levels expressed as a log_2_ ratio for each line compared to Dd2 (aggregated group of Dd2 and Dd2^Dd2crt^). Undetected metabolites are indicated in grey. Numbers correspond to pathways shown in (B). (**B**) List of KEGG metabolic pathways showing significant differences in metabolite abundance for each mutant line compared with the aggregated Dd2 data (*p<0.05; **p<0.01; Q-statistic calculated from a generalized linear model).

Targeted metabolomic analyses of all the lines detected 69 known metabolites. Metabolite analysis of each Dd2 *pfcrt*-edited line versus the Dd2 control lines revealed overlapping changes in metabolites for Dd2^F145crt^ and Dd2^G353Vcrt^ (**Fig 4A**), such as L-aspartic acid. Metabolomic quantitative set enrichment analyses applied to these PPQ-resistant mutant lines revealed that both the F145I and G353V mutations affected the basal levels of metabolites related to amino acid metabolism/degradation pathways. These pathways included alanine, aspartate, arginine and histidine metabolism, as well as aminoacyl-tRNA biosynthesis that is involved in delivering amino acids to ribosomes for incorporation into polypeptide chains produced during translation (**Fig 4B**). We also observed PfCRT isoform-specific differences, such as lysine degradation that was downregulated in the Dd2^F145crt^ line. In contrast to the highly resistant lines, Dd2^M343Lcrt^ showed smaller changes and was associated with lowered levels of gluconic acid found in the pentose phosphate pathway (**Fig 4B**).

### PPQ phenotypes exhibit differential transcriptomic profiles

**Fig 5:**
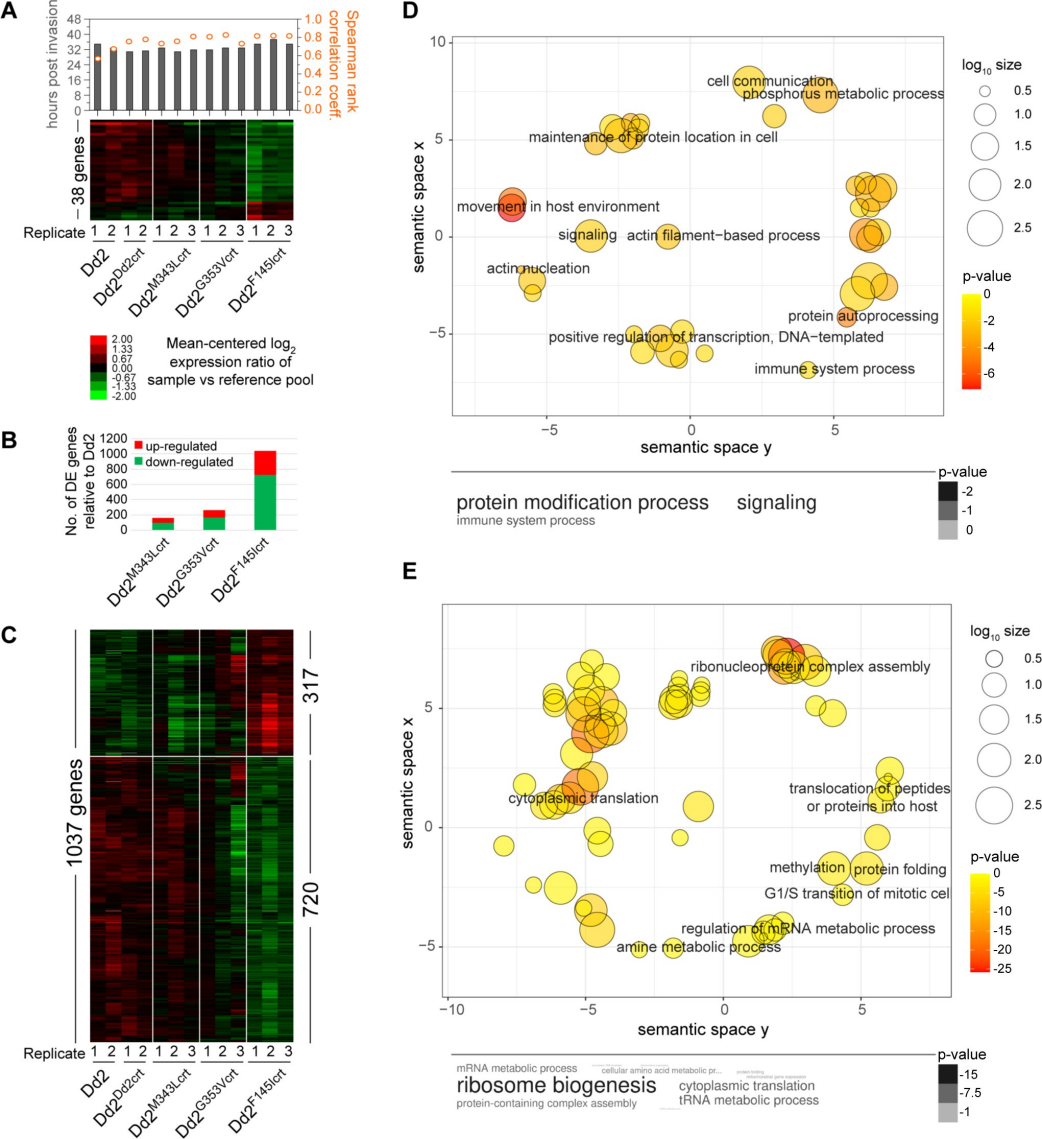
Differential gene expression in PPQ-resistant and PPQ-sensitive PfCRT variants. **(A)** Mean-centered hierarchically clustered heat map of 38 genes that exhibit differential expression between the four isogenic Dd2 parasites (N = 3–4; one-way ANOVA with permutation *p*<0.05). Bars and scatter points, shown above, depict the mapped parasite stage (in hr post-invasion) and the Spearman rank correlation coefficient of each sample, as determined against the 48 hr Dd2 reference transcriptome, respectively. (**B**) Total number of differentially expressed genes for each mutant line relative to the aggregated group of Dd2^Dd2crt^ and Dd2 parasites (N = 3–4; Student t-tests with permutation *p*<0.05)**. (C)** Mean-centered hierarchically clustered heat map analysis of 1037 genes that exhibit differential expression between the Dd2^F145Icrt^ and the aggregated Dd2 parasites (N = 3–4; Student t-tests with permutation *p*<0.05). **(D, E)** Significantly enriched pathways in the **(D)** 317 up-regulated and **(E)** 720 down-regulated genes observed in the Dd2^F145Icrt^ line relative to the aggregated Dd2 parasites (*p*<0.05). Pathways were identified using Gene Ontology (GO) enrichment of computed and curated biological processes and visualized by REVIGO to obtain representative pathways. The colors indicate the *p* value of each GO term and size indicates the frequency of the GO term in the *P*. *falciparum* database. Listed *p* values are to the power of 10.

The global transcriptional program in *P*. *falciparum* is characterized by a tightly coordinated cascade of gene regulation during the parasite’s developmental progression that enables some degree of adaptation to specific conditions [51,52]. To gain insight into transcriptional differences associated with PPQR, we compared global transcription levels between the PPQ-resistant and PPQ-sensitive lines by analyzing their steady state mRNA levels in mature parasites (32 to 34 hr post-invasion). The best fit of the stage of each experimental replicate was based on peak correlation values matched to the previously generated *P*. *falciparum in vitro* transcriptome imputed at 30 min intervals [53], as determined by Spearman rank correlation analysis (**Fig 5A**). Our coefficient values (mean ± SD: 0.76 ± 0.07) demonstrated good synchronicity of the cultures. Using one-way ANOVA (Bonferroni *p*<0.05 with permutations) to compare each isogenic PPQ-resistant PfCRT mutant line with Dd2^Dd2crt^, we identified 38 differentially expressed (DE) genes (**Fig 5A**). This set included genes encoding several ribosomal protein that were downregulated in the PPQ-resistant lines (**S5 Table**). Overall, the biggest difference was observed in the Dd2^F145Icrt^ line, in agreement with the peptidomic results (**Fig 5A**). To further examine which genes and biological pathways could account for the observed differences between the PPQ-resistant mutant lines and the PPQ-sensitive Dd2 parasites, we performed Student’s t-tests. This analysis identified 1,037 DE genes, of which 317 (30.5%) and 720 were up- or down-regulated, respectively, in Dd2^F145Icrt^ relative to Dd2^Dd2crt^ (Student’s t-test with Bonferroni corrections, *p*<0.05 with permutation and Fold Change ≥1.14) (**Fig 5B** and **5C** and **S6 Table**). The Dd2^F145Icrt^ and Dd2^G353Vcrt^ lines displayed similar trends with the former having a greater degree of difference when compared to the control **(Fig 5B**). The same transcriptional analysis performed on the Dd2^G353Vcrt^ and Dd2^M343Lcrt^ mutants revealed a smaller number of 259 and 162 DE genes, respectively, compared to Dd2 (**Figs 5B**, **S4 and S5 and S6 Table**). This finding suggests that PPQR is associated with specific changes in the transcriptional program of *P*. *falciparum* ABS parasites.

The DE genes were further characterized based on GO enrichment and clustered using REVIGO [54], using the PlasmoDB database. GO enrichment of the upregulated genes in Dd2^F145Icrt^ (based on a cutoff of *p*<0.05) comprised molecular functions involved in actin filament organization, cell signaling, and protein phosphorylation (**Fig 5D**). GO enrichment of the 720 downregulated genes in Dd2^F145Icrt^ yielded molecular functions involved in pathways that converged on protein translation, ribonucleoprotein complex assembly, protein folding, cellular metabolism and mRNA processing (**Fig 5E**). Similarly, our analysis revealed that genes involved in ribonucleoprotein complex assembly and cytoplasmic translation were also down-regulated in the Dd2^G353Vcrt^ and Dd2^M343Lcrt^ lines (**S4 and S5 Figs**).

To identify genes potentially regulating the earlier observed differences in the peptidomics/metabolomics analyses between PPQ-resistant and PPQ-sensitive lines, we conducted a joint analysis of the transcriptome and metabolome on the Dd2^F145Icrt^ line, which displayed the biggest differences at the peptide, metabolite and transcript levels in comparison to the Dd2 lines. This analysis revealed differential expression of metabolites and genes involved in the parasite’s aminoacyl-tRNA biosynthesis as well as alanine, aspartate, and glutamate metabolic pathways (**S6–S8 Figs**). These results suggest that the PPQR-conferring PfCRT F145I mutation associates with reduced expression of parasite factors required for amino acid metabolism and protein translation, which presumably results from lower availability of Hb-processed amino acids.

### PPQ-resistant mutant PfCRT parasites are sensitized to aminopeptidase inhibition

**Fig 6:**
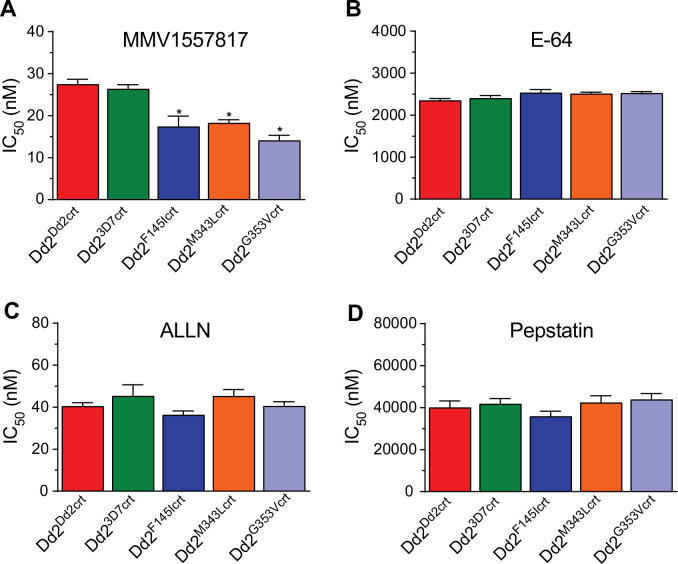
PfCRT-mutant PPQ parasites show increased susceptibility to aminopeptidase M17 inhibition. Dose–response plots illustrate susceptibility of PfCRT mutants to aminopeptidase inhibition. Half maximal inhibitory concentrations (IC_50_ and IC_90_) are presented as the mean ± SEM (N, n  =  4, 2) for **(A)** MMV1557817; **(B)** E-64; **(C)** ALLN; and **(D)** pepstatin, as determined from conventional 72 hr dose–response assays performed with asynchronous parasite cultures. Mean ± SEM IC_50_ and IC_90_ values are shown in **S6 Table**. Statistical significance was determined using Mann–Whitney *U* tests comparing the PfCRT mutant lines with their isogenic Dd2^Dd2crt^ parent. *p<0.05; ns, not significant.

Aspartic, cysteine and metallo-proteases constitute the three main catalytic enzyme groups involved in early-stage Hb hydrolysis while downstream of this process the parasite additionally encodes aminopeptidases that cleave amino acid residues from peptide chains [55]. PfA-M1 and PfA-M17 comprise the main contributors to the amino acid pool required for ABS development and are known to be essential [56]. This indispensable role in Hb breakdown and conservation across *Plasmodium* species has led to the identification of candidate inhibitors of these enzymes. Based on our observation of excessive peptide accumulation in the PfCRT mutant lines, we investigated the vulnerability of the PfCRT mutants’ aminopeptidome by profiling known inhibitors of the three Hb-degrading proteases. These inhibitors were E64 (a falcipain inhibitor), N-acetyl-Leu-Leu-norleucinal, (ALLN, a calpain inhibitor) and pepstatin (an aspartic protease inhibitor). We also included MMV1557817 (referred to as compound 6l in [57]), a putative dual inhibitor of PfA-M17 leucyl aminopeptidase (PF3D7_1446200) and PfA-M1 alanyl aminopeptidase (PF3D7_1311800), with a 4-fold lower K_i_ for inhibition of the former. Both aminopeptidases have been implicated in hydrolyzing peptides released from Hb, with PfA-M17 and PfA-M1 localized to the parasite cytosol and DV, respectively [58,59]. Our *in vitro* resistance selection studies with MMV1557817 identified an A460S mutation in PfA-M17, further implicating this enzyme in this inhibitor’s mode of action. We also observed a N846I mutation in a putative AP-3 complex beta subunit (PF3D7_0613500) and a M317I mutation in a conserved *Plasmodium* protein of unknown function (PF3D7_1144400), which we suspect are bystander mutations. There was no significant difference in the activity of E64, ALLN or pepstatin against all lines, with ALLN being the most active of the three. However, MMV1557817 exhibited significantly higher potency against the PfCRT mutant lines compared to Dd2^Dd2crt^ (**Fig 6** and **S7 Table**). These data suggest that there might be insufficient aminopeptidase activity in the PPQ-resistant PfCRT mutant lines, which would corroborate the sub-optimal breakdown of the short Hb peptides observed earlier in this study. Alternatively, the greater sensitivity of these parasites to MMV1557817 might reflect mutant PfCRT-mediated transport of this compound away from its site of action. Further studies are required to address these hypotheses, including through the use of inhibitors that have recently been shown to have increased specificity for PfA-M17 [60].

## Discussion

In this study, we leveraged a set of Dd2-based *pfcrt*-edited isogenic lines with distinct drug susceptibility profiles to characterize the cellular phenotypes associated with *P*. *falciparum* resistance to the 4-aminoquinolines PPQ and CQ. Drug accumulation and susceptibility profiling, combined with drug-heme, metabolomics, peptidomics, and transcriptomics studies, highlight the intrinsic adjustments to parasite physiology caused by the acquisition of PPQR-conferring mutations in PfCRT. The most significant changes were observed with the highly PPQ resistant Dd2^F145Icrt^ mutant, followed by the Dd2^G353Vcrt^ and Dd2^M343Lcrt^ lines that show moderate and low levels of PPQR, respectively.

Accumulation studies with tritiated PPQ and CQ revealed core differences in how mutant PfCRT mediates the transport of these related 4-aminoquinoline drugs at low nanomolar concentrations. For [^3^H]-CQ, cellular accumulation ratios were highly correlated with the level of parasite susceptibility: the highly CQ-resistant Dd2^Dd2crt^ line showed minimal accumulation, contrasting with substantially higher CQ accumulation in CQ-sensitive lines expressing the 3D7 WT or PPQR-conferring mutant isoforms. These data suggest that CQ efflux out of the DV and away from its heme target is a dominant feature of mutant PfCRT-mediated CQR [23,24,61,62]. For [^3^H]-PPQ, however, there was only a modest difference in accumulation between resistant and sensitive parasites, despite PPQ IC_90_ values being nearly 100-fold higher in the most highly-resistant Dd2^F145Icrt^ line compared to the parasites expressing the PPQ-sensitive 3D7 isoform.

Differences in CQ and PPQ resistance phenotypes were also evident in the dose-response data. For CQ the IC_50_ and IC_90_ values were both increased in resistant parasites, whereas for PPQ the resistance phenotype was only evident at high drug concentrations, with essentially unchanged IC_50_ values (**S1 Table**). Physiologically, these data suggest that the PPQR mechanism only engages in the presence of high drug concentrations. Further studies are planned to test [^3^H]-PPQ accumulation across a substantially higher range of concentrations to more closely match drug levels that reveal the resistance phenotype in 72 hr concentration-response assays.

One possible explanation for the PPQ accumulation data not matching drug susceptibility, unlike CQ, may relate to different protonation states of these drugs. At the acidic pH of the DV (estimated at ~5.2 ([63,64]), CQ is expected to become protonated at both its nitrogens, whereas PPQ (harboring two 4-aminoquinoline rings as compared with the one for CQ) can have up to four protonated nitrogens. CQ efflux is dependent on H^+^ co-transport [65], but above a certain concentration of PPQ inside the DV there may be sufficient protons to permit transport without a limiting rate of H^+^ cotransport. As a related hypothesis, PPQ expulsion from the DV may only be permitted following conformational changes to PfCRT following its binding to PPQ at higher concentrations. Two lines of evidence support this argument. First, a recent quantification of PPQ transport in *Saccharomyces cerevisiae* yeast expressing PPQR-associated PfCRT isoforms (harboring the mutations F145I, I218F, M343L, C350R or G353V), observed a clear distinction between PfCRT-catalyzed transport and passive transport only at 300 μM PPQ [66]. This PPQ concentration, premised on high PPQ levels in plasma, yielded a strong linear correlation (*R*^2^ = 0.93) between PfCRT-mediated [^3^H]-PPQ accumulation and PfCRT-dependent growth delay of yeast cells upon PPQ treatment. Secondly, the recent cryo-EM elucidation of the PfCRT 7G8 structure indicate that F145I, G353V and other PPQR-associated mutations face into the central cavity of the protein. Molecular dynamic simulations provided evidence that F145I induced a conformational change in PfCRT that involved substantial displacement of transmembrane domains 1 and 7 [21], with this work and other homology modeling suggesting that changes in cavity shape and size can affect molecular interactions essential for differential drug transport as observed for PPQ and CQ [67]. PfCRT itself may also be a target, such that drug-PfCRT interactions themselves cause growth inhibition and parasite death, separate from drug transport [34].

Our data also provide evidence for a central role for PfCRT isoforms in contributing to Hb metabolism and availability of Hb-derived peptides, which are essential to the intracellular development and replication of blood-stage *Plasmodium* parasites [55,68]. Indeed, most clinically important antimalarial drugs intersect with this pathway at some level, be it via downstream inhibition of heme detoxification (for PPQ, CQ, pyronaridine, and amodiaquine) or possibly upstream inhibition of Hb endocytosis. We observed substantial accumulation of undigested Hb in Dd2 parasites expressing the PfCRT mutations F145I and G353V, but not M343L. This observation aligns with previous reports of a substantial growth defect in Dd2 parasites expressing F145I or G353V but not M343L [20]. This growth defect was the most pronounced in the F145I mutant, an initial driver of high-level PPQR in the field that was later overtaken by fitter PfCRT variants [29,30,69]. Impaired growth of PPQ-resistant lines has also been associated with distended DVs in trophozoite and schizont stages [20,70]. This morphological defect was most pronounced with the F145I and G353V mutations, which unlike M343L face inwards to the central cavity of PfCRT through which solutes and drug are predicted to move [21].

These observations allude to potential nutritional deficiencies in PPQR lines compared to their sensitive counterparts. Although parasites expressing F145I or G353V did not differ significantly from their isogenic parent in the amounts of ‘free heme’ incorporated in their DV, they nonetheless had significantly lower levels of Hz (**Fig 2** and **S3 Table**). In a separate study, culture-adapted PPQ-sensitive *P*. *falciparum* parasites treated with physiological concentrations of PPQ accumulated high amounts of undigested vesicle-enclosed Hb and reduced Hz levels in the DV [71]. Earlier studies provide evidence that the mutational status of PfCRT can also impact DV volume and pH, both of which influence Hz formation kinetics [72,73]. These findings coupled with our observations of altered Hb and Hz levels in some PPQ-resistant PfCRT lines suggest that PPQ’s mode of action could involve inhibition of both early Hb digestion and Hz formation. The low Hz levels occasioned by impaired Hb catabolism in these PPQ-resistant PfCRT mutants may also preclude the heme biomineralization-inhibiting activity of PPQ.

Quantitative metabolomics have previously shown a link between CQR and significantly higher levels of Hb-derived peptides in PfCRT mutant parasites compared to their WT-expressing counterparts [42,46,49,73]. Our PPQ-resistant PfCRT mutants replicate this phenotype, as seen mostly in the edited F145I and G353V mutants and less so in the M343L mutant. Elevated peptide levels were also observed to a more moderate extent in the two PPQ-resistant clinical isolates RF7 and RF12 (that harbor the Dd2+M343L and Dd2+H97Y isoforms, respectively). Those isolates, respectively, carry three and two copies of plasmepsins 2/3, which have been reported to increase parasite survival at high PPQ concentrations [14,17–19,74]. These data suggest that overexpression of plasmepsins 2/3 may also act to reduce the peptide imbalance arising from novel PfCRT mutations. Interestingly, recent studies observed reduced peptide levels in ART-resistant parasites [49,50], further connecting Hb metabolism with the mode of action of 4-aminoquinolines as well as ART derivatives.

Collectively, our results suggest that PfCRT mutations play an important role in dictating levels of short oligopeptides in parasites. This impaired Hb catabolism is predicted to impact the amino acid pool available for protein synthesis, osmotic integrity, and the number of merozoites that can develop within the physical confines of the infected RBCs. This suboptimal physiological context agrees with the lower parasite multiplication rate documented in the F145I and G353V mutants [20]. Increased peptide accumulation in our set of PfCRT mutants suggests that one of the native functions of PfCRT involves shuttling peptides from the DV lumen to the parasite’s cytosol for subsequent liberation of amino acids and alleviation of osmotic stress from the DV, with this function being impaired in drug-resistant PfCRT mutants. In support of this interpretation, a study of PfCRT isoforms expressed in *X*. *laevis* oocytes found that PfCRT could transport peptides ranging from 4 to 11 amino acids, with CQ-sensitive 3D7 permitting a larger number and broader range of peptides than the CQ-resistant Dd2 and Ecu1110 isoforms [46]. That study also showed PfCRT-mediated efflux of VDPVNF. Efflux could be inhibited by several agents including verapamil, chlorpheniramine, CQ, saquinavir and a quinine dimer that might interact with the substrate-binding cavity of PfCRT. Studies are merited to now further dissect peptide transport using PfCRT protein reconstituted into proteoliposomes [21].

In light of the elevated peptide levels in the PfCRT mutants, we examined whether these parasites may also reveal alterations in other metabolic pathways via changes to their transcriptome. Time-course analysis of tightly-synchronized parasites revealed differential expression of 1,037 genes in late trophozoites from the mutant Dd2^F145Icrt^ line compared to the Dd2^Dd2crt^ parent. The biggest difference was observed in the highly PPQ-resistant line, Dd2^F145Icrt^. Gene set enrichment analysis revealed up-regulation of pathways involved in cell signaling and post-translational modifications (including protein phosphorylation). All three PPQR mutant lines also showed commonalities in down-regulated pathways including cytoplasmic translation, ribonucleoprotein complex assembly, cell cycle regulation and RNA processing. These data suggest that changes to the PfCRT sequence or expression levels impact multiple aspects of parasite physiology including protein synthesis, trafficking and turnover, as well as the transcription and translation machinery.

Our analysis of the pathways significantly enriched in both our transcriptome and metabolome datasets, when comparing Dd2^F145Icrt^ and Dd2^Dd2crt^, places metabolism of the amino acids alanine, aspartate and glutamate as well as aminoacyl-tRNA biosynthesis at the center of this convergence between changes in peptides and transcript levels. This is supported by evidence of depletion of aminoacyl-tRNA ligases and synthetases in the CQ-sensitive back-mutant 7G8^pfcrt_T76K^ compared to the CQ-resistant parental 7G8 [75]. These findings suggest that impaired PfCRT transport through the acquisition of resistance-conferring mutations might affect the cellular level of amino acids, and support our observations of excessive peptide accumulation that likely impacts amino acid reserves available for protein synthesis and parasite development.

To explore whether the parasite aminopeptidome might expose a potential vulnerability in PPQ-resistant PfCRT mutant parasites, we profiled MMV1557817, a recently published inhibitor of the PfA-M17 leucyl aminopeptidase that also has some activity against the PfA-M1 alanyl aminopeptidase [57]. PfA-M17 is a neutral metalloaminopeptidase whose selective *in vitro* inhibition is lethal to the parasites [60]. This enzyme plays a role in the final and essential processes of Hb digestion, thus its inhibition is expected to impair the release of amino acids that would in turn impact protein synthesis and intra-erythrocytic osmotic regulation [59,76]. The PfA-M17 aminopeptidase is thought to act predominantly in the parasite cytosol on short peptides exported from the DV [57]. Our data (**Fig 6**) raise the exciting possibility that targeting this aminopeptidase, or other enzymatic mediators of Hb proteolysis, might provide new opportunities to inhibit PPQ-resistant parasites.

## Materials and methods

### Ethics statement

Human RBCs used in this study were purchased from the Interstate Blood Bank (Memphis, TN) as whole blood from anonymized donors. Approval to use this material for *P*. *falciparum in vitro* culture was granted by the Columbia University Medical Center Institutional Review Board, which has classified this work as not being human subjects research.

### Parasite cultures

The *P*. *falciparum* parasites used herein comprise isogenic lines on a Dd2 background into which the PPQ-sensitive Dd2 and 3D7 *pfcrt* alleles, or the PPQ-resistant F145I, M343L or G353V mutations, were introduced via gene editing (**Table 1**; [20]). RF7 and RF12 are Cambodian clinical isolates (referred to as PH1008-C and PH1263-C, respectively in [20] and collected in 2012–2013) that harbor the PfCRT M343L and H97Y mutations, respectively, added to the Dd2 PfCRT haplotype. Both isolates have a single copy of *pfmdr1* and express the ART-resistant K13 C580Y mutation. The Dd2-B2 clone was used for the Dd2 parent [77]. Parasite lines were cultured *in vitro* in 0.5% Albumax-supplemented RPMI 1640-based *P*. *falciparum* culture medium [78]. PCR and Sanger sequencing were used to authenticate the *pfcrt* locus in each parasite line. Two consecutive 5% D-sorbitol treatments were used to tightly synchronize the cultures and magnetic column purifications were performed to obtain highly-enriched trophozoites.

### Measurement of [^3^H]-CQ and [^3^H]-PPQ uptake in cultured parasites

Sorbitol-synchronized parasite cultures were collected as early trophozoites (24 to 28 hr post-invasion) and magnet-purified (VarioMACS, Miltenyi Biotec). Enriched trophozoite-infected erythrocytes were eluted at 37°C in fresh 1× PBS containing 2 mM EDTA and supplemented with 0.5% bovine serum albumin. The eluate was then centrifuged (1,500 rpm for 5 min), and the pellet was washed and resuspended in bicarbonate-free RPMI 1640 medium (supplemented with 25 mM HEPES, 10 mM glucose and 0.2 mM hypoxanthine, adjusted to pH 7.4) at 37°C. Cell numbers were calculated by counting an aliquot of resuspended cells using a hemocytometer. Uptake measurements began with the addition of 250 μL of trophozoite suspension, at an estimated hematocrit of 30,000–120,000 cells per μL, to an equal volume of 10 nM [^3^H]-CQ (60 Ci mmol^−1^) or 40 nM [^3^H]-PPQ (15 Ci mmol^−1^) in bicarbonate-free medium to yield final [^3^H]-CQ and [^3^H]-PPQ concentrations of 5 nM and 20 nM, respectively. These labeled drugs were purchased from American Radiolabeled Chemicals. Parallel control measurements were conducted with uninfected RBCs at the same hematocrit. After 1 hr of incubation at 37°C in a water bath, duplicate 200 μL aliquots of the suspensions were transferred into 1.5-mL Eppendorf tubes containing 300 μL of dibutyl phthalate (Sigma Aldrich, 1.04 g ml^−1^) and centrifuged immediately (13,000 rpm for 2 min) to sediment the cells, thereby terminating [^3^H]-drug uptake. From each duplicate, 100 μL of supernatant was transferred into a vial containing 5 mL of scintillation fluid (CytoScint, MP Biomedicals) to determine the external drug concentration [Drug_external_]. The pellets were digested by overnight incubation at 55°C with 100 μL tissue solubilizer (NCS-II, GE Healthcare) diluted 1:2 in absolute ethanol, then bleached the next day with 30% hydrogen peroxide (25 μL) followed by the addition of 25 μL glacial acetic acid to block luminescence. Digested pellets were transferred into 10 mL of scintillation cocktail and measured in a scintillation counter to determine the internal drug concentration [Drug_internal_]. After adjusting for non-specific uptake by subtracting measurements from the uninfected erythrocyte controls, CAR was calculated as a ratio of [Drug_internal_] / [Drug_external_], normalized to 10^6^ infected RBCs and assuming a mean volume of a trophozoite-infected RBC of 75 femtoliters [79,80].

### Cellular heme fractionation assay

Baseline levels of different heme species in the parasite lines were determined using pyridine-based detergent-mediated cellular heme fractionation assays (**Fig 2**; [43]). Briefly, each parasite line was synchronized to ring stages using two cycles of sorbitol treatment and early rings (~3 hr post-invasion) were incubated at 37°C at 5% parasitemia and 2% hematocrit in 24-well plates. After 30 hr, late trophozoites were harvested by lysing RBCs with 0.05% saponin followed by multiple washes with 1×PBS (pH 7.5) to remove traces of Hb. Pellets were then resuspended in 1×PBS and an aliquot of the trophozoite suspension was stained and quantified via flow cytometry (as per above) to determine the total number of trophozoites. DV contents were then released from trophozoites by hypotonic lysis and sonication. Parasite fractions corresponding to digested Hb, free heme-Fe and Hz were then carefully recovered through centrifugation and treatment with HEPES buffer (pH 7.4), 4% SDS, 25% pyridine solution, 0.3M HCl and 0.3M NaOH. The UV-visible spectrum of each heme fraction was measured as a Fe^3+^-heme-pyridine complex using a multi-well SpectraMax P340 plate reader (Molecular Devices). The total amount of each heme-Fe species per parasite was quantified using a heme standard curve where the mass per trophozoite (fg/cell) was calculated by dividing the total amount of each species by the corresponding number of parasites in that fraction, as determined by flow cytometry. We also measured total heme-Fe concentrations in Dd2^Dd2crt^-infected RBCs as well as uninfected RBCs as reference data. Statistical analyses used Mann-Whitney *U* tests.

### *In vitro* drug susceptibility assays

*In vitro* susceptibility to PPQ, CQ, E64, ALLN, pepstatin and MMV1557817 was measured in parasites incubated at 37°C with 0.2% starting parasitemia and 1% hematocrit, assayed in 96-well plates across a range of drug concentrations with two-fold dilutions. Parasite growth in each well was assessed after 72 hr using flow cytometric analysis of cultures stained with SYBR Green I and MitoTracker Deep Red [81], with ~10,000 cells analyzed per well using an iQue Plus (Sartorius) or a FACS Celesta (Becton Dickinson). For PPQ, IC_50_ and IC_90_ values were extrapolated by linear regression, because of the unusual dose-response curves observed in PPQ-resistant lines [16,20]. For all other antimalarials the *in vitro* IC_50_ values were determined by nonlinear regression analysis with GraphPad Prism 7 software. Statistical comparisons between cell lines were made using Mann–Whitney *U* tests. Assays were repeated in duplicate on 4 to 5 independent occasions.

### Sample preparation for untargeted LC-MS metabolomics

Testing for *Mycoplasma* was performed using a MycoAlert PLUS Mycoplasma Detection Kit (Lonza) prior to the start of the sample collection. *Mycoplasma*-free parasites were sorbitol-synchronized in each generation for at least two generations followed by magnetic enrichment of 32 hr post-invasion trophozoites using MACS CS columns on a SuperMACS™ II Separator (Miltenyi Biotec) to remove uninfected RBCs. Trophozoite counts were determined on a hemocytometer. Parasites were lysed in 1 mL of 90% cold methanol, containing 0.5 μM of the internal standard [^13^C_4_, ^15^N_1_]-Aspartate (Cambridge Isotope) to correct for technical variations arising from sample processing. Samples were vortexed to disrupt cell pellets and to generate uniform homogenates, which were centrifuged (13,000× g for 10 min) and the supernatants then harvested. Supernatants were dried under nitrogen prior to resuspension in HPLC-grade water for LC-MS analysis. Samples were randomized and 5 μL of extract or processing blank was injected for analysis. Metabolites were analyzed using a previously established reversed phase ion-paired method on a HPLC Prominence 20 UFLCXR system (Shimadzu) with a Waters BEH C18 column (100mm x 2.1mm 1.7 μm particle size) at 55°C and an aqueous acetonitrile gradient run for 20 min at a flow rate of 250 μL/min. Eluate was delivered into a (QTOF) 5600 TripleTOF using a Duospray™ ion source (AB Sciex). Capillary voltage was 5.5kV in positive and negative ion mode with declustering potential of 80V. The TripleTOF was scanning 50 to 1000 m/z, and 16 MS/MS product ion scans (100 ms) per duty cycle using collision energy of 50V with a 20V spread. Solvent A was HPLC grade water with 0.1% formic acid and Solvent B was HPLC grade acetonitrile with 0.1% formic acid.

### Data analyses for metabolomics

Analyses were performed as previously described [82]. Raw data files from the 5600 (QTOF) TripleTOF (.WIFF) were converted to a format compatible with our analysis software (.mzML) and spectral data (.mzML files) were visualized in MS-DIAL version 4.80. The labeled [^13^C_4_, ^15^N_1_]-Aspartate internal standard intensity was assessed for technical reproducibility. Metabolites were identified using an MS-DIAL internal database/library and the MSP spectral kit containing EI-MS, MS/MS, and CCS values [83,84]. Identification was based on peak proximity to retention time and m/z, with the observed mass falling within 2 ppm of the expected m/z (calculated from the monoisotopic mass), and the signal/blank ratio (minimum of 10,000 ions). Peak areas from both positive and negative modes were exported for downstream analysis. The chlorpropamide standard was used as an internal control to normalize individual metabolite peak areas between runs. The peak areas for duplicate metabolites that had the same m/z values were summed before blank subtraction. Peak areas of the blanks were subtracted from the samples for each metabolite and in each experiment. Samples that had peak areas less than the blank metabolites (sample area < blank area) were defined as “NA” prior to averaging the technical triplicates. For the peptide analysis, peptides were mapped by searching all possible peptides derived from the hemoglobin α and β chains using the calculated theoretical m/z value and retention time in both positive and negative modes of the experimental observations. The isoelectric point (IEP) and charge at pH 5.5 and pH 7.4 for each oligopeptide was calculated using the web-based isoelectric point calculator IPC2.0 [85].

To investigate the effect of PPQ-resistant PfCRT mutations on *P*. *falciparum* metabolism, the log_2_ fold changes of mutant vs. Dd2^Dd2crt^ metabolites were calculated for each experimental run using the averaged replicate peak areas. Data from three independent experiments, each with three technical replicates, were used for these analyses. To further examine the effects of PPQ-resistant PfCRT mutations, we applied metabolomic set enrichment analyses and partial least-squares discriminant analyses (MetaboAnalyst 5.0 package) for which data were standardized through log_10_ transformation and mean centering.

### Sample preparation for microarray-based transcriptomics

To obtain tightly-synchronized parasites, all lines were doubly synchronized with 5% D-sorbitol for at least two cycles prior to collecting samples for RNA and stage synchrony was confirmed by microscopy. The same samples harvested for metabolomics were also collected in parallel for transcriptomics profiling. These were at ~32 hr post-invasion, as confirmed later by computationally assessing transcriptomic profiles through Spearman rank correlation calculations for each parasite sample mapped against the intra-erythrocytic developmental cycle time points of a highly-synchronized Dd2 reference transcriptome [53]. After washing the packed RBC pellets with 1×PBS (pH 7.4), a 10× pellet volume of Trizol was added to the pellet and total RNA was extracted using an acidified phenol-chloroform method [86]. Total RNA was reverse transcribed into cDNA using Superscript II reverse transcriptase and the SMART protocol, with template switching at the cDNA 3′ ends [86]. The cDNA was amino allyl-dUTP labeled using 30 cycles of PCR amplification. Four μg of each sample were then labeled with Cy5 fluorescent dyes and mixed with an equal amount of a Cy3-labeled pool comprising mixed ABS parasites from the reference 3D7 strain. Samples were hybridized at 65°C for 17 hr on a *P*. *falciparum* 60-mer long oligonucleotide DNA microarray chip containing 15,744 probes representing 5,363 coding genes (GEO Platform: GPL15130; Agilent Technologies, AMADID #037237) [87]. Arrays were washed and scanned using the Agilent G2600D microarray scanner (Agilent Technologies).

### Data analyses for transcriptomics

Normalized intensities were extracted using the Agilent feature extractor software version 11.5.1.1 and uploaded to the Princeton University Microarray Database (PUMA.princeton.edu) for analysis. After background subtraction, the median log_2_ of the (Cy5/Cy3) intensity ratio was extracted. Ratios of all probes mapping to each gene were averaged to obtain a list of gene log_2_-transformed ratios, representing the log_2_ ratio of transcript abundance in the sample to transcript abundance in the 3D7 reference pool. Genes encoding variable surface antigens of *pfemp1*, *rifin* and *stevor* were filtered out. Genes were annotated accordingly using PlasmoDB version 46 [88]. Differential expression analysis between all the parasites lines, or the pair-wise comparisons between the PPQ-resistant lines and PPQ-sensitive Dd2^Dd2crt^, were performed using one-way ANOVA or Student’s t-tests with Bonferroni corrections and permutations, respectively. GO enrichment analysis for differentially expressed genes was performed using the PlasmoDB GO analysis, *p*<0.05, and REVIGO tools [54].

### *In vitro* resistance selection studies with MMV155787 and whole-genome sequencing

*P*. *falciparum* cultures were seeded with 2×10^9^ asexual blood stage parasites (using the Dd2-B2 clone) per flask in triplicate flasks and exposed to 90 nM (5× the IC_50_ calculated at that time). Parasites recrudesced in each flask by days 17–20 and clones were obtained by limiting dilution. Concentration-response assays showed IC_50_ increases estimated at 2.0 to 3.6 fold the IC_50_ for Dd2-B2. We then chose one clone from each flask to submit for whole-genome sequencing. Genomic DNA was prepared for clones FL1-B4, FL2-F1, and FL3-C6, as well as parental Dd2-B2, using the Qiagen QIAamp DNA Blood Midi Kit. Paired-end 2×300 bp sequencing libraries were prepared using the Illumina TruSeq DNA PCR-Free library preparation protocol and run on an Illumina MiSeq instrument. Sequence data were aligned to the *P*.* falciparum* 3D7 genome (PlasmoDB version 48.0) using BWA (Burrow-Wheeler Alignment). Unmapped reads and PCR duplicates were filtered out using Samtools and Picard. The reads were realigned around indels using GATK RealignerTargetCreator and base quality scores were recalibrated using GATK BaseRecalibrator. GATK UnifiedGenotyper was used to identify all possible single nucleotide variants in resistant parasite lines. These variants were filtered based on quality scores (variant quality as function of depth QD > 1.5, mapping quality > 40, min base quality score > 16, read depth > 5) to obtain high quality single nucleotide polymorphisms (SNPs) that were annotated using snpEFF. No copy number variations were observed using BicSeq, which compared read counts of the resistant clones against the Dd2-B2 parent. Sequencing yielded good quality reads corresponding to a depth of coverage of 40 to 48-fold for the resistant clones. For each clone, greater than 94% of the *P*. *falciparum* genome had at least 10 reads. The list of variants from the resistant clones were compared against the Dd2-B2 parent to obtain homozygous SNPs present exclusively in the resistant clones.

## Supporting information

S1 Fig Heatmaps of peptide levels in *P*. *falciparum* lines studied herein. **(A)** Heatmap of 309 peptides that show differential levels between mutant *pfcrt* lines vs. Dd2^3D7crt^ (>4-fold in at least one line). **(B)** Heatmap of 393 peptides that show differential levels between mutant *pfcrt* lines vs. Dd2^Dd2crt^.
(PDF)

S2 Fig Distribution across the hemoglobin α and β chains for significantly enriched peptides. Colors represent significant log_2_ fold changes in peptide levels between Dd2^F145Icrt^ or Dd2^G353Vcrt^ parasites compared to Dd2^Dd2crt^. Data are grouped between peptides that map to the **(A)** Hbα or **(B)** Hbβ chains. If the same peptide sequence was detected in both modes then the color represents the log_2_ ratio in the negative mode. Red and blue are the most and least abundant, respectively. Asterisks indicate sequences where peptides were not detected. Dd2^F145Icrt^ parasites showed far more differences than Dd2^G353Vcrt^ parasites. No differences were observed for Dd2^M343Lcrt^ parasites.
(PDF)

S3 Fig Mapping of drug resistance mutations onto the PfCRT structure. Residues that contribute to resistance to CQ or PPQ are mapped onto the Dd2 structure (modeled from the 7G8 isoform, solved by cryo-electron microscopy to 3.2Å). Mutations have their side chains rendered as sticks and are colored based on their associated resistance profiles. The remaining structures are rendered in cartoon and colored in grey. Views are shown vertically (digestive vacuole (DV) lumen to the bottom, with the pink dashed line illustrating the predicted position of the DV membrane) and from the DV side (rotated 90° to illustrate PfCRT’s central cavity). F45I and G353V are both located on the DV side of the membrane, whereas M343L is on the cytosolic side. The structure was solved in an “open to DV” conformation, with the cavity presumably able to flip between “open to DV” and “open to cytosol” conformations during the transport of drug or solute. F145I and G353V face inwards towards the central cavity.
(PDF)

S4 Fig Differential gene expression in Dd2^G353Vcrt^ versus Dd2. **(A)** Mean-centered hierarchically clustered heat map analysis of 259 genes that exhibit differential expression between the Dd2^G353Vcrt^ and the combination of Dd2^Dd2crt^ and Dd2 parasites (N = 3–4; Student t-tests with permutation *p*<0.05). **(B, C)** Significantly enriched pathways in the **(B)** 94 up-regulated and **(C)** 165 down-regulated genes observed in the Dd2^G353Vcrt^ line relative to Dd2 parasites (*p*<0.05). Pathways were identified using Gene Ontology (GO) enrichment of computed and curated biological processes and visualized by REVIGO to obtain representative pathways. The colors indicate the *p* value of each GO term and size indicates the frequency of the GO term in the *P*. *falciparum* database. Refer to **S6 Table** for the list of genes.
(PDF)

S5 Fig Differential gene expression in Dd2^M343Lcrt^ versus Dd2. **(A)** Mean-centered hierarchically clustered heat map analysis of 162 genes that exhibit differential expression between the Dd2^M343Lcrt^ and the combination of Dd2^Dd2crt^ and Dd2 parasites (N = 3–4; Student t-tests with permutation *p*<0.05). **(B, C)** Significantly enriched pathways in the **(B)** 65 up-regulated and **(C)** 97 down-regulated genes observed in the Dd2^M343Lcrt^ line relative to Dd2 parasites (*p*<0.05). Pathways were identified using Gene Ontology (GO) enrichment of computed and curated biological processes and visualized by REVIGO to obtain representative pathways. The colors indicate the *p* value of each GO term and size indicates the frequency of the GO term in the *P*. *falciparum* database. Refer to **S6 Table** for the list of genes.
(PDF)

S6 Fig Joint analysis of metabolomic and transcriptomic data. Scatterplot of the enrichment analysis performed using metabolomic and transcriptomic data showing the -log_10_ *p* value (using a Student t-test) of the pathway and % pathway impact, comparing Dd2^F145Icrt^ and Dd2^Dd2crt^ at basal level in the trophozoite stage. Differential expression of two pathways (*p*< 0.05) is shown. Circle sizes reflect the pathway impact value, which represents the cumulative percentage of matched metabolite/gene nodes with respect to the total pathway based on pathway topological analyses. N  =  3 independent experiments.
(PDF)

S7 Fig Pathway analysis of the significantly differentially expressed genes and metabolites in aminoacyl tRNA biosynthesis, shown as squares or circles, respectively. (PDF)

S8 Fig Pathway analysis of the significantly differentially expressed genes and metabolites in alanine, aspartate and glutamate metabolism, shown as squares or circles, respectively. (PDF)

S1 Table [^3^H]-drug accumulation and 72 hr susceptibility profiling of *pfcrt*-edited lines. Mean ± SEM cellular accumulation ratios (CARs) and IC_50_ and IC_90_ values were determined from 4 to 5 independent experiments performed in duplicate. Statistical significance was determined using Mann-Whitney *U* tests. p values are reported for comparisons with Dd2^Dd2crt^. *p<0.05; **p<0.01. The raw data used for this analysis is in S1 Data.
(PDF)

S2 Table Baseline levels of heme species in *pfcrt*-edited lines. Mean ± SEM amounts of hemoglobin, free heme and hemozoin are represented as fg per trophozoite. The amounts of heme species in different parasite lines were determined by heme fractionation (see Materials and Methods). Statistical comparisons to the parental Dd2^Dd2crt^ control were performed using Mann-Whitney *U* tests. *p <0.05. Data were calculated from 4 independent experiments performed in duplicate. Control measurements with total RBC extracts prepared from cell cultures infected with Dd2^Dd2crt^ parasites yielded mean ± SEM values 97.8 ± 1.4 fg/cell (comprising the parasite and the host cell cytosol), whereas uninfected RBCs yielded a mean ± SEM value of 104.1 ± 1.1 fg/cell (from 4 separate experiments performed with technical duplicates).
(PDF)

S3 Table Averaged log_2_ fold change of the baseline peptide levels in the *pfcrt*-edited and field lines versus Dd2^Dd2crt^. (PDF)

S4 Table List of peptides showing significantly different levels in Dd2^F145Icrt^ or Dd2^G353Vcrt^ lines compared with Dd2^Dd2crt^. (PDF)

S5 Table Differentially expressed genes identified from one-way ANOVA of Dd2^F145Icrt^, Dd2^G353Vcrt^, Dd2^M343Lcrt^ and the combined set of Dd2^Dd2crt^ and Dd2 (*p*<0.05; with Bonferroni corrections and permutations). (PDF)

S6 Table List of differentially expressed genes in Dd2^F145Icrt^, Dd2^G353Vcrt^ and Dd2^M343Lcrt^, compared with the combined set of Dd2^Dd2crt^ and Dd2 (*p*<0.05; based on Student’s t-test with Bonferroni corrections and permutations). (PDF)

S7 Table *In vitro* 72 hr susceptibility of *pfcrt*-edited lines against hemoglobinase or aminopeptidase inhibitors. Data show means ± SEM, calculated from four independent experiment with technical duplicates. Significance was determined using Mann-Whitney *U* tests. *p<0.05. MMV1557817 is also known as MIPS-1778.
(PDF)

S1 Data Raw data of cellular accumulation ratios for all *pfcrt*-edited lines. (XLSX)
