# Supplementary figures and images for: Piperaquine-resistant PfCRT mutations differentially impact drug transport, hemoglobin catabolism and parasite physiology in Plasmodium falciparum asexual blood stages
Source: PLoS Pathog. 2022 Oct 28;18(10):e1010926. doi: 10.1371/journal.ppat.1010926 (PMC9645663; doi:10.1371/journal.ppat.1010926)

# S1 Figure

A

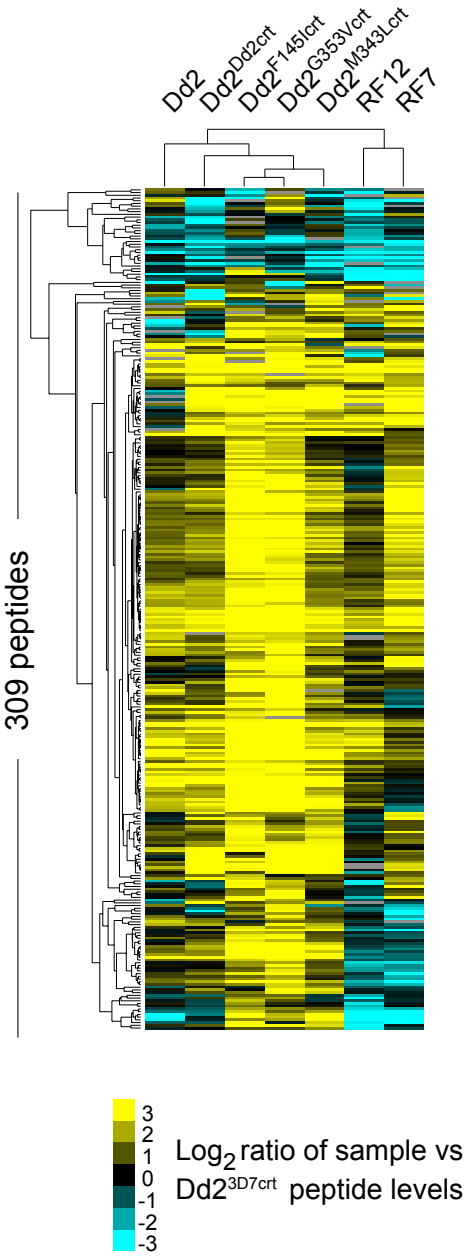

B

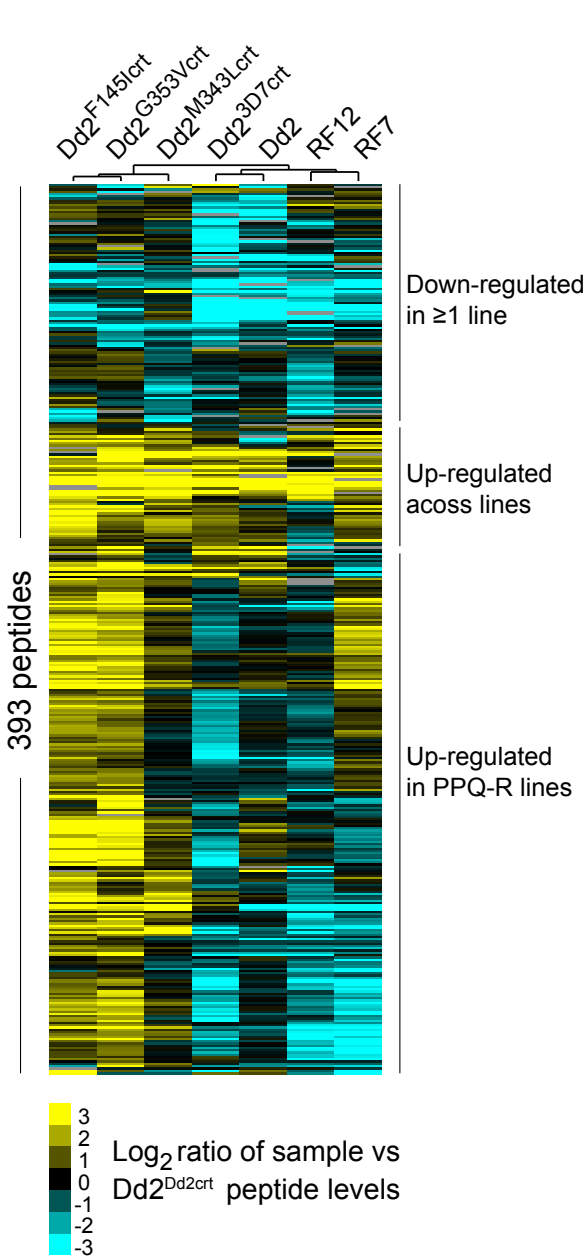

Supplement: S1 Fig — (A) Heatmap of 309 peptides that show differential levels between mutant pfcrt lines vs. Dd23D7crt (>4-fold in at least one line). (B) Heatmap of 393 peptides that show differential levels between mutant pfcrt lines vs. Dd2Dd2crt. (PDF) [file ppat.1010926.s001.pdf]

# S2 Figure

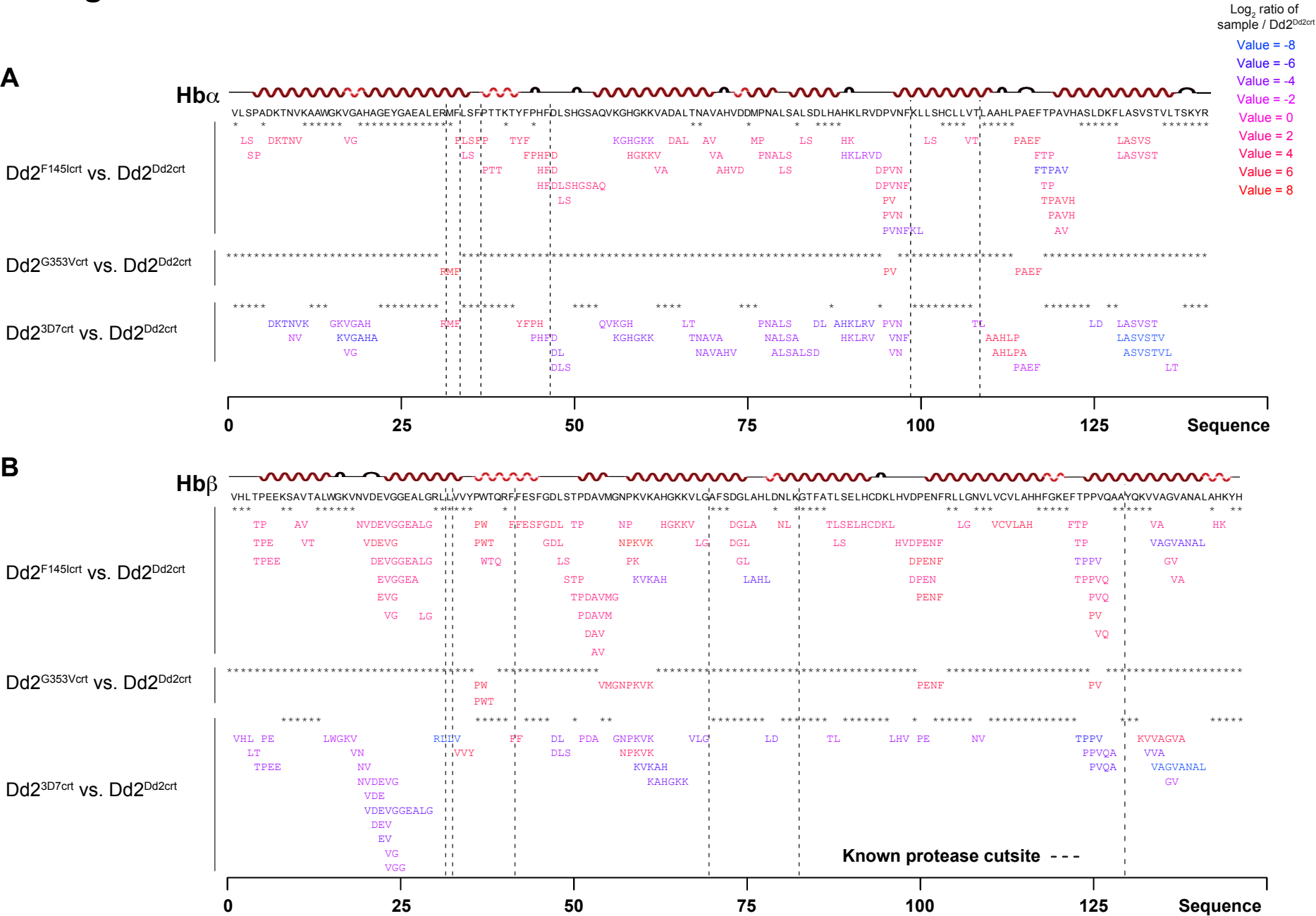

Supplement: S2 Fig — Colors represent significant log2 fold changes in peptide levels between Dd2F145Icrt or Dd2G353Vcrt parasites compared to Dd2Dd2crt. Data are grouped between peptides that map to the (A) Hbα or (B) Hbβ chains. If the same peptide sequence was detected in both modes then the color represents the log2 ratio in the negative mode. Red and blue are the most and least abundant, respectively. Asterisks indicate sequences where peptides were not detected. Dd2F145Icrt parasites showed far more differences than Dd2G353Vcrt parasites. No differences were observed for Dd2M343Lcrt parasites. (PDF) [file ppat.1010926.s002.pdf]

## S3 Figure

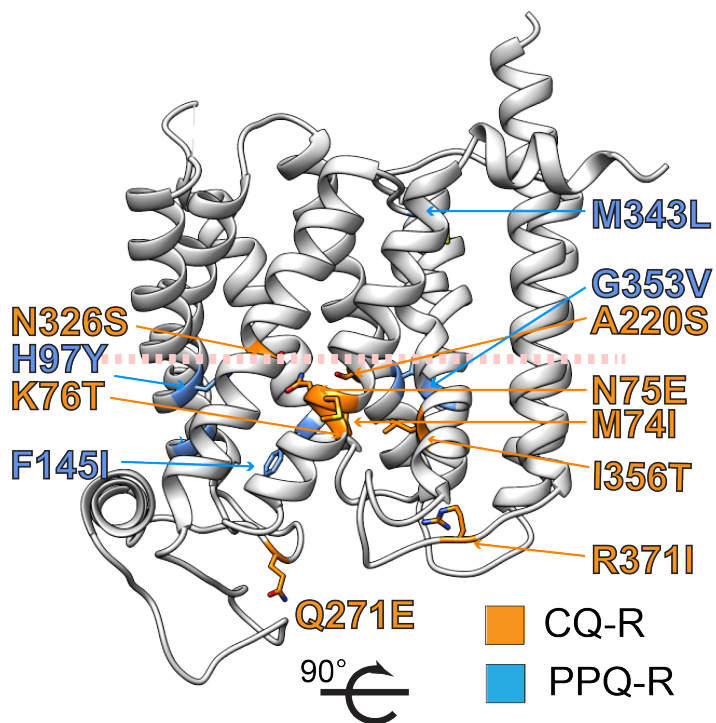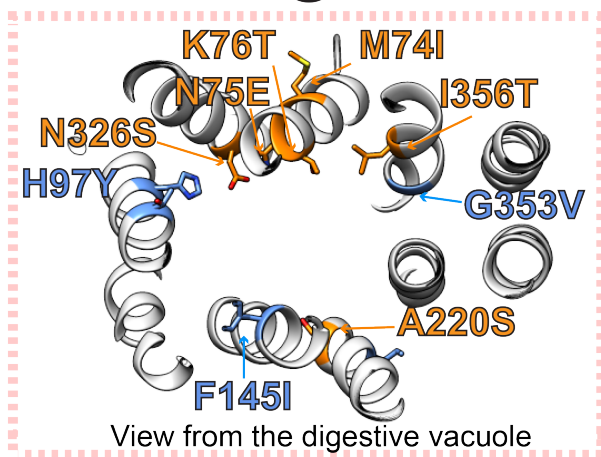

Supplement: S3 Fig — Residues that contribute to resistance to CQ or PPQ are mapped onto the Dd2 structure (modeled from the 7G8 isoform, solved by cryo-electron microscopy to 3.2Å). Mutations have their side chains rendered as sticks and are colored based on their associated resistance profiles. The remaining structures are rendered in cartoon and colored in grey. Views are shown vertically (digestive vacuole (DV) lumen to the bottom, with the pink dashed line illustrating the predicted position of the DV membrane) and from the DV side (rotated 90° to illustrate PfCRT’s central cavity). F45I and G353V are both located on the DV side of the membrane, whereas M343L is on the cytosolic side. The structure was solved in an “open to DV” conformation, with the cavity presumably able to flip between “open to DV” and “open to cytosol” conformations during the transport of drug or solute. F145I and G353V face inwards towards the central cavity. (PDF) [file ppat.1010926.s003.pdf]

# S4 Figure

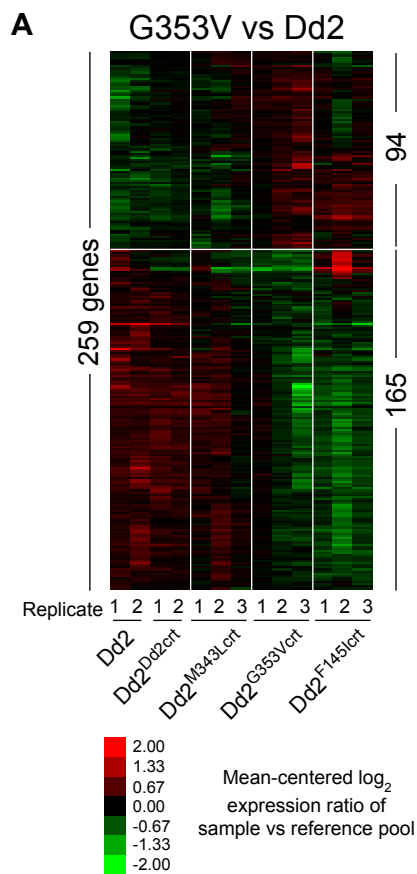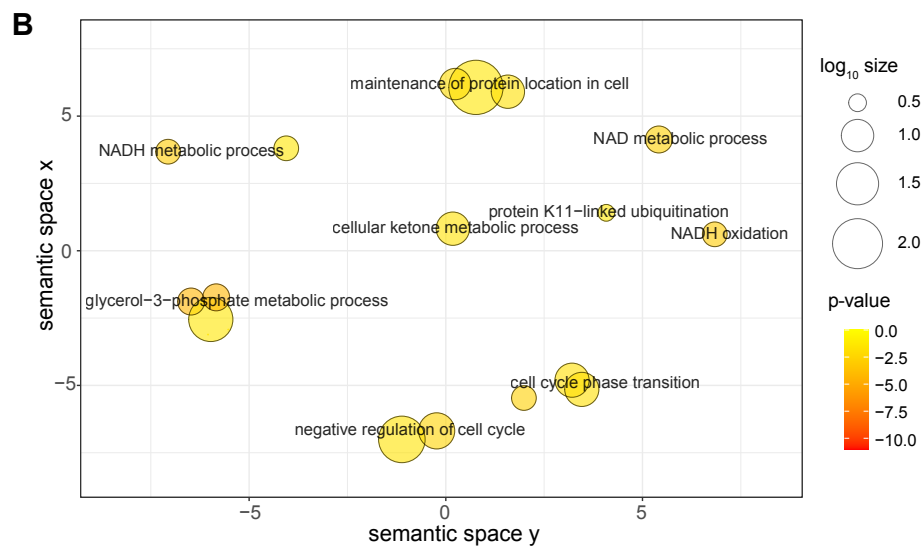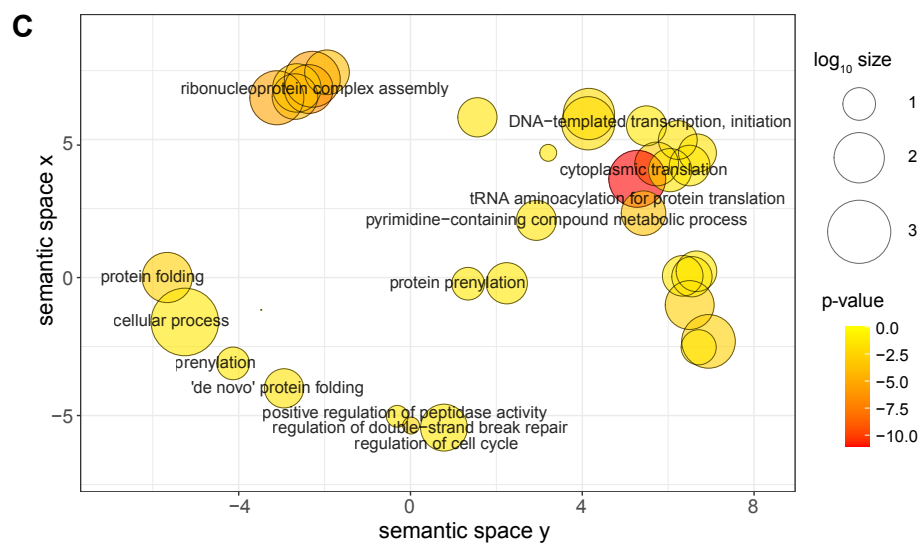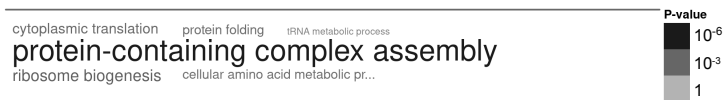

Supplement: S4 Fig — (A) Mean-centered hierarchically clustered heat map analysis of 259 genes that exhibit differential expression between the Dd2G353Vcrt and the combination of Dd2Dd2crt and Dd2 parasites (N = 3–4; Student t-tests with permutation p<0.05). (B, C) Significantly enriched pathways in the (B) 94 up-regulated and (C) 165 down-regulated genes observed in the Dd2G353Vcrt line relative to Dd2 parasites (p<0.05). Pathways were identified using Gene Ontology (GO) enrichment of computed and curated biological processes and visualized by REVIGO to obtain representative pathways. The colors indicate the p value of each GO term and size indicates the frequency of the GO term in the P. falciparum database. Refer to S6 Table for the list of genes. (PDF) [file ppat.1010926.s004.pdf]

# S5 Figure

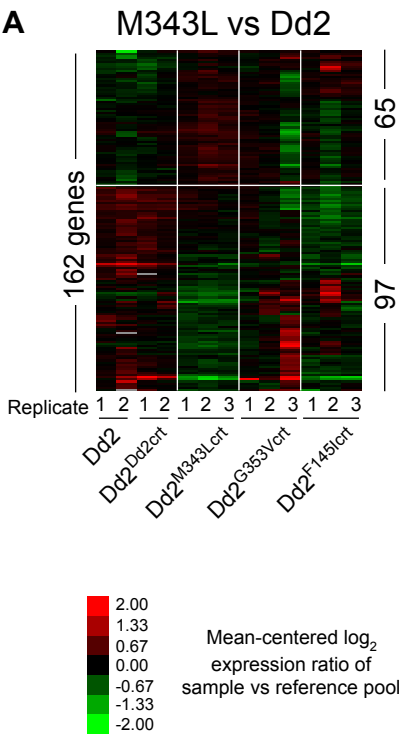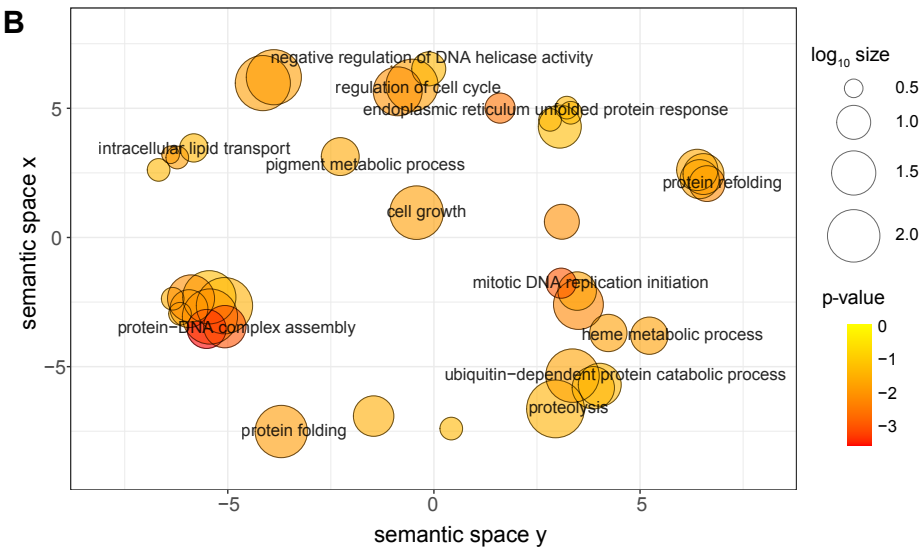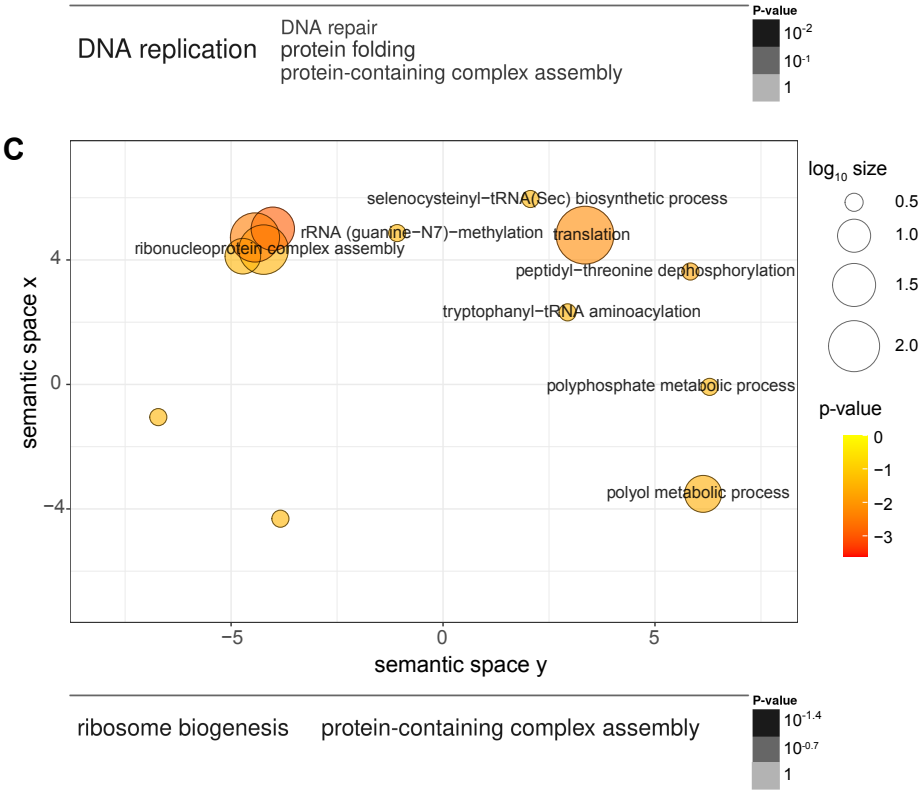

Supplement: S5 Fig — (A) Mean-centered hierarchically clustered heat map analysis of 162 genes that exhibit differential expression between the Dd2M343Lcrt and the combination of Dd2Dd2crt and Dd2 parasites (N = 3–4; Student t-tests with permutation p<0.05). (B, C) Significantly enriched pathways in the (B) 65 up-regulated and (C) 97 down-regulated genes observed in the Dd2M343Lcrt line relative to Dd2 parasites (p<0.05). Pathways were identified using Gene Ontology (GO) enrichment of computed and curated biological processes and visualized by REVIGO to obtain representative pathways. The colors indicate the p value of each GO term and size indicates the frequency of the GO term in the P. falciparum database. Refer to S6 Table for the list of genes. (PDF) [file ppat.1010926.s005.pdf]

## S7 Figure

Alanine, aspartate and glutamate metabolism

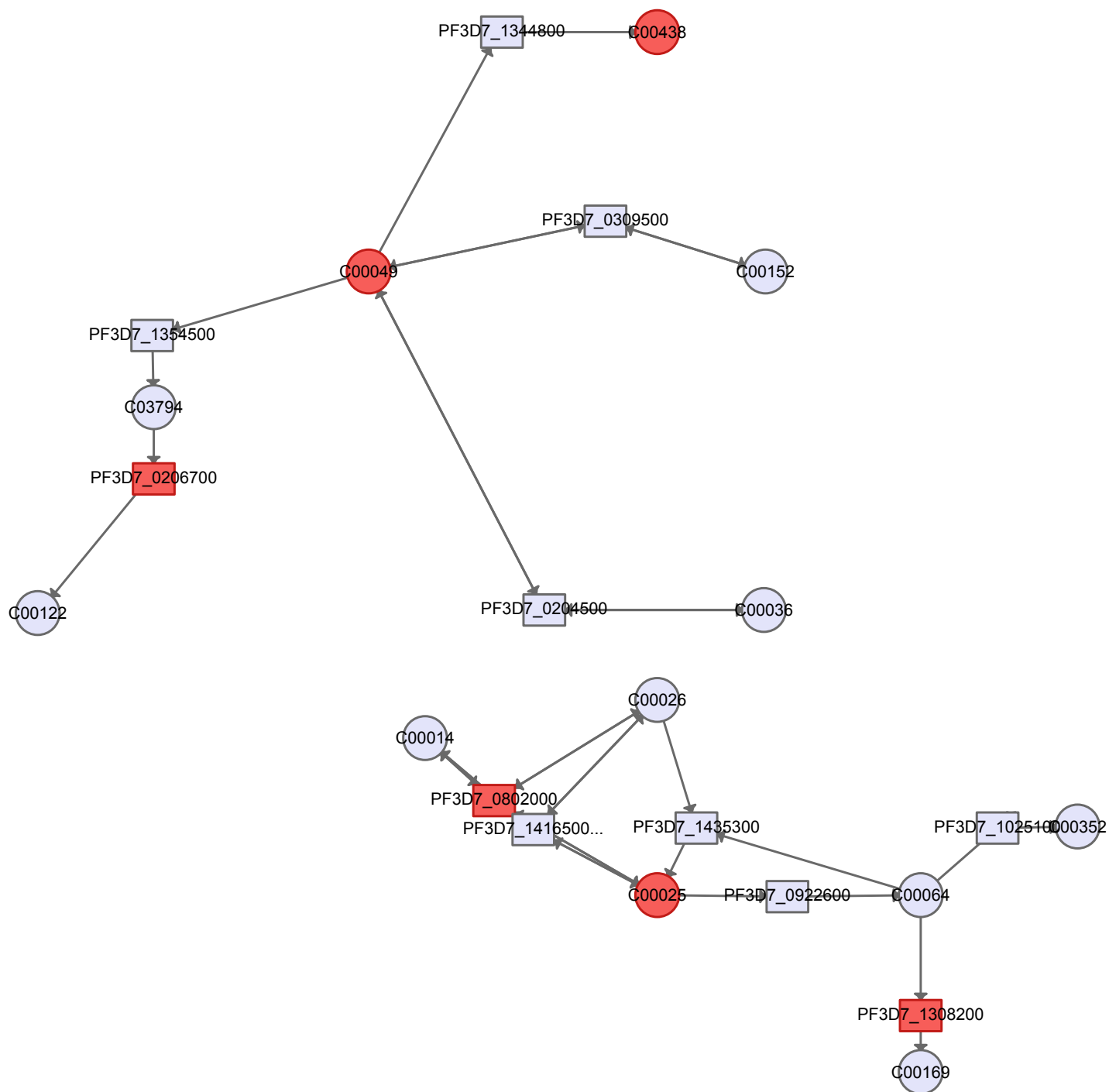

Supplement: S7 Fig — (PDF) [file ppat.1010926.s007.pdf]

# S8 Figure

## Aminoacyl-tRNA biosynthesis

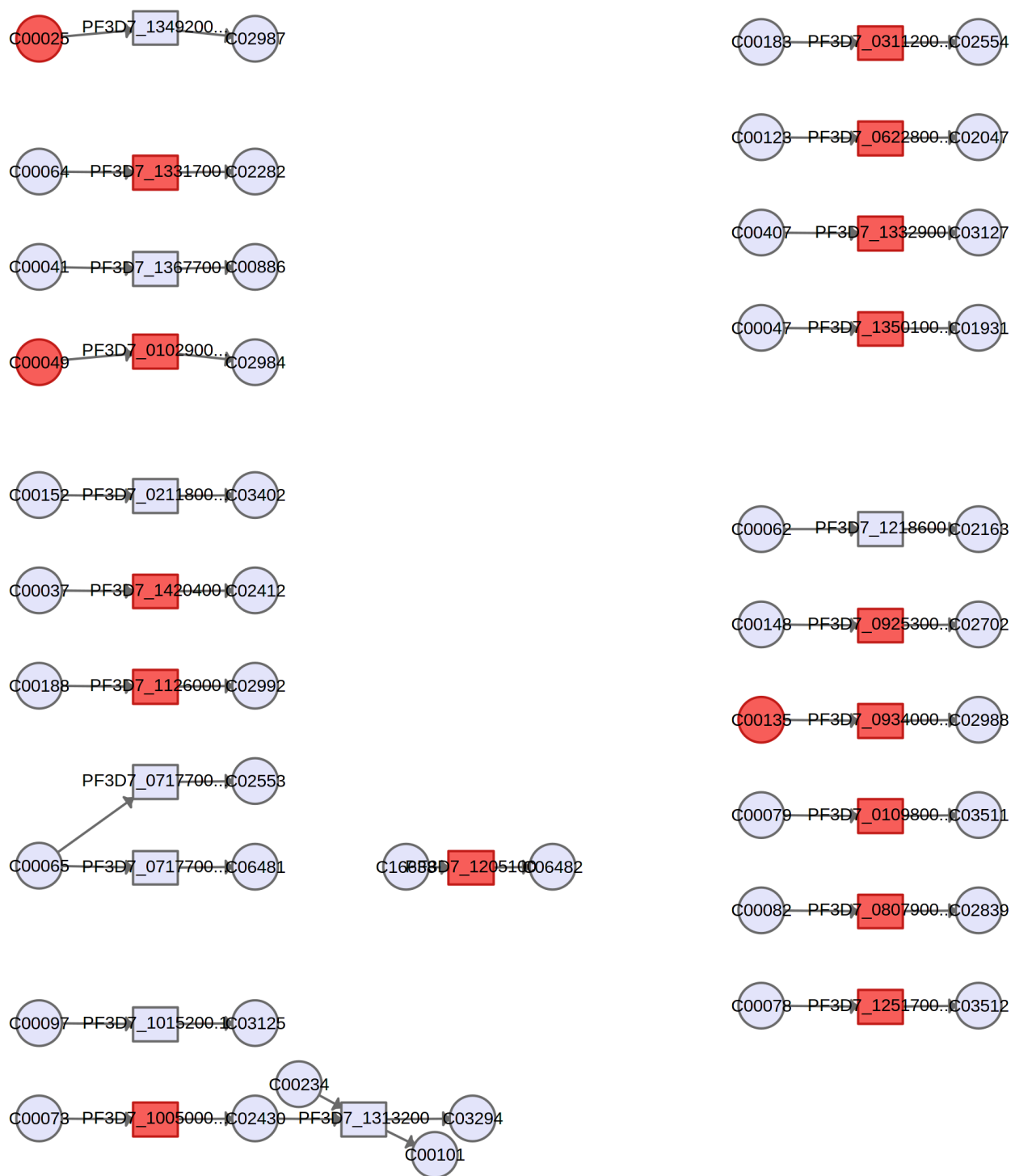

Supplement: S8 Fig — (PDF) [file ppat.1010926.s008.pdf]
