# Supplementary material for: Piperaquine-resistant PfCRT mutations differentially impact drug transport, hemoglobin catabolism and parasite physiology in Plasmodium falciparum asexual blood stages
Source: PLoS Pathog. 2022 Oct 28;18(10):e1010926. doi: 10.1371/journal.ppat.1010926 (PMC9645663; doi:10.1371/journal.ppat.1010926)
Supplement: S6 Fig — Scatterplot of the enrichment analysis performed using metabolomic and transcriptomic data showing the -log10 p value (using a Student t-test) of the pathway and % pathway impact, comparing Dd2F145Icrt and Dd2Dd2crt at basal level in the trophozoite stage. Differential expression of two pathways (p< 0.05) is shown. Circle sizes reflect the pathway impact value, which represents the cumulative percentage of matched metabolite/gene nodes with respect to the total pathway based on pathway topological analyses. N = 3 independent experiments. (PDF) [file ppat.1010926.s006.pdf]

S6 Figure

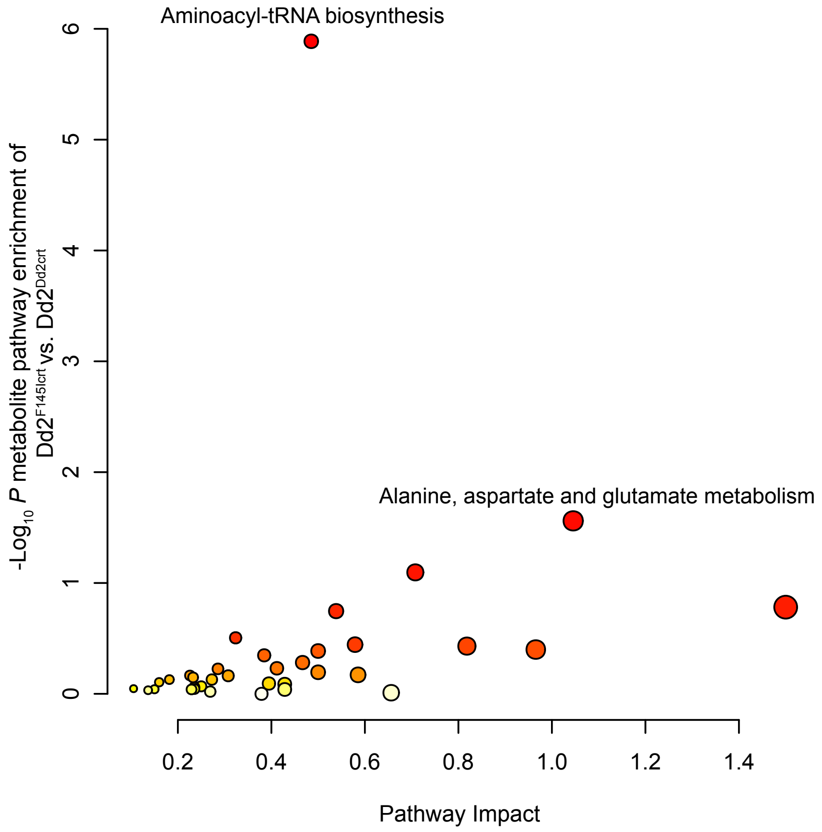

Pathway	Total	Expected	Hits	Raw p	-LOG10(p)	Holm adjust	FDR	Impact
Aminoacyl-tRNA biosynthesis	69	7.34	21	1.30E-06	5.89	6.48E-05	6.48E-05	0.49
Alanine, aspartate and glutamate metabolism	23	2.45	6	0.027506	1.56	1	0.68766	1.05
Purine metabolism	90	9.58	14	0.080213	1.10	1	1	0.71
Nitrogen metabolism	7	0.75	2	0.16524	0.78	1	1	1.50
Nicotinate and nicotinamide metabolism	14	1.49	3	0.17939	0.75	1	1	0.54
Glutathione metabolism	35	3.73	5	0.31241	0.51	1	1	0.32
Glyoxylate and dicarboxylate metabolism	20	2.13	3	0.35997	0.44	1	1	0.58
Arginine biosynthesis	12	1.28	2	0.37107	0.43	1	1	0.82
Pentose phosphate pathway	30	3.19	4	0.39867	0.40	1	1	0.97
Lysine degradation	13	1.38	2	0.41089	0.39	1	1	0.50
Selenocompound metabolism	14	1.49	2	0.44947	0.35	1	1	0.38
Glycine, serine and threonine metabolism	16	1.70	2	0.52223	0.28	1	1	0.47
Arginine and proline metabolism	18	1.92	2	0.58841	0.23	1	1	0.41
Phosphonate and phosphinate metabolism	8	0.85	1	0.59548	0.23	1	1	0.29
Starch and sucrose metabolism	9	0.96	1	0.63903	0.19	1	1	0.50
Glycolysis or Gluconeogenesis	42	4.47	4	0.67425	0.17	1	1	0.59
Porphyrin and chlorophyll metabolism	32	3.41	3	0.68268	0.17	1	1	0.23
Pyrimidine metabolism	53	5.64	5	0.68728	0.16	1	1	0.31
Glycerophospholipid metabolism	44	4.68	4	0.711	0.15	1	1	0.23
Glycerolipid metabolism	12	1.28	1	0.74377	0.13	1	1	0.18
Vitamin B6 metabolism	12	1.28	1	0.74377	0.13	1	1	0.27
Cysteine and methionine metabolism	26	2.77	2	0.78586	0.10	1	1	0.16
Citrate cycle (TCA cycle)	39	4.15	3	0.80735	0.09	1	1	0.39
One carbon pool by folate	15	1.60	1	0.8184	0.09	1	1	0.43
Pantothenate and CoA biosynthesis	17	1.81	1	0.85577	0.07	1	1	0.25
Biotin metabolism	18	1.92	1	0.87149	0.06	1	1	0.24
Phosphatidylinositol signaling system	20	2.13	1	0.89804	0.05	1	1	0.11
Pyruvate metabolism	35	3.73	2	0.90508	0.04	1	1	0.24
Thiamine metabolism	21	2.24	1	0.90921	0.04	1	1	0.15
Amino sugar and nucleotide sugar metabolism	49	5.22	3	0.91304	0.04	1	1	0.23
Folate biosynthesis	36	3.83	2	0.91362	0.04	1	1	0.43
Fructose and mannose metabolism	23	2.45	1	0.92804	0.03	1	1	0.14
Propanoate metabolism	27	2.87	1	0.9549	0.02	1	1	0.27
Valine, leucine and isoleucine degradation	33	3.51	1	0.97774	0.01	1	1	0.66
Fatty acid biosynthesis	96	10.22	1	0.99999	0.00	1	1	0.38
