# Supplementary material for: Piperaquine-resistant PfCRT mutations differentially impact drug transport, hemoglobin catabolism and parasite physiology in Plasmodium falciparum asexual blood stages
Source: PLoS Pathog. 2022 Oct 28;18(10):e1010926. doi: 10.1371/journal.ppat.1010926 (PMC9645663; doi:10.1371/journal.ppat.1010926)
Supplement: S1 Table — Mean ± SEM cellular accumulation ratios (CARs) and IC50 and IC90 values were determined from 4 to 5 independent experiments performed in duplicate. Statistical significance was determined using Mann-Whitney U tests. p values are reported for comparisons with Dd2Dd2crt. *p<0.05; **p<0.01. The raw data used for this analysis is in S1 Data. (PDF) [file ppat.1010926.s009.pdf]

S1 Table. [^{3}H]-drug accumulation and 72 hr susceptibility profiling of *pfcrt* -edited lines.

	Piperaquine			Chloroquine		
Line	CAR	IC_{50} (nM)	IC_{90} (nM)	CAR	IC_{50} (nM)	IC_{90} (nM)
$Dd2^{Dd2crt}$	1786 ± 164	19.9 ± 2.4	34.4 ± 1.2	24.7 ± 3.0	82.7 ± 5.6	144 ± 11.6
$Dd2^{F145Icrt}$	1135 ± 67	76.8 ± 11.8 *	1775 ± 334 *	838 ± 24.0 *	20.3 ± 0.8 *	45.5 ± 2.8 *
$Dd2^{M343Lcrt}$	1348 ± 109	32.1 ± 1.8 *	214 ± 12.0 *	662 ± 20.0 *	20.8 ± 1.1 *	43.0 ± 0.7 *
$Dd2^{G353Vcrt}$	1186 ± 140	51.5 ± 6.7 *	674 ± 66 *	540 ± 10.7 *	19.8 ± 1.1 *	46.8 ± 1.1 *
$Dd2^{3D7crt}$	1980 ± 110	9.5 ± 1.1 *	18.9 ± 2.0 *	1161 ± 123 **	9.5 ± 1.1 *	11.2 ± 0.2 *

Mean ± SEM cellular accumulation ratios (CARs) and IC_{50} and IC_{90} values were determined from 4 to 5 independent experiments performed in duplicate. Statistical significance was determined using Mann-Whitney *U* tests. *p* values are reported for comparisons with $Dd2^{Dd2crt}$. *p <0.05; **p <0.01. The raw data used for this analysis is in **S1 Dataset**.
