# Supplementary material for: Piperaquine-resistant PfCRT mutations differentially impact drug transport, hemoglobin catabolism and parasite physiology in Plasmodium falciparum asexual blood stages
Source: PLoS Pathog. 2022 Oct 28;18(10):e1010926. doi: 10.1371/journal.ppat.1010926 (PMC9645663; doi:10.1371/journal.ppat.1010926)
Supplement: S2 Table — Mean ± SEM amounts of hemoglobin, free heme and hemozoin are represented as fg per trophozoite. The amounts of heme species in different parasite lines were determined by heme fractionation (see Materials and Methods). Statistical comparisons to the parental Dd2Dd2crt control were performed using Mann-Whitney U tests. *p <0.05. Data were calculated from 4 independent experiments performed in duplicate. Control measurements with total RBC extracts prepared from cell cultures infected with Dd2Dd2crt parasites yielded mean ± SEM values 97.8 ± 1.4 fg/cell (comprising the parasite and the host cell cytosol), whereas uninfected RBCs yielded a mean ± SEM value of 104.1 ± 1.1 fg/cell (from 4 separate experiments performed with technical duplicates). (PDF) [file ppat.1010926.s010.pdf]

S2 Table. Baseline levels of heme species in *pfcrt* -edited lines.

	Amounts of heme species (fg/trophozoite)			
Line	Hemoglobin	"Free" heme	Hemozoin	Total heme
Dd2^{Dd2crt}	1.6 ± 0.1	3.7 ± 0.2	51.2 ± 0.8	56.5 ± 1.5
Dd2^{F145Icrt}	3.6 ± 0.1 *	3.0 ± 0.3	39.2 ± 1.0 *	45.7 ± 1.1 *
Dd2^{M343Lcrt}	1.6 ± 0.1	3.4 ± 0.2	47.2 ± 0.8 *	52.2 ± 0.7 *
Dd2^{G353Vcrt}	2.1 ± 0.1 *	2.8 ± 0.2	40.6 ± 1.2 *	45.6 ± 1.4 *
Dd2^{3D7crt}	1.7 ± 0.1	4.2 ± 0.4	55.4 ± 0.5 *	61.3 ± 0.9 *

Mean ± SEM amounts of hemoglobin, free heme and hemozoin are represented as fg per trophozoite. The amounts of heme species in different parasite lines were determined by heme fractionation (see Methods). Statistical comparisons to the parental Dd2^{Dd2crt} control were performed using Mann-Whitney *U* tests. *p<0.05. Data were calculated from 4 independent experiments performed in duplicate. Control measurements with total RBC extracts prepared from cell cultures infected with Dd2^{Dd2crt} parasites yielded mean ± SEM values 97.8 ± 1.4 fg/cell (comprising the parasite and the host cell cytosol), whereas uninfected RBCs yielded a mean ± SEM value of 104.1 ± 1.1 fg/cell (from 4 separate experiments performed with technical duplicates).
