# Supplementary material for: Piperaquine-resistant PfCRT mutations differentially impact drug transport, hemoglobin catabolism and parasite physiology in Plasmodium falciparum asexual blood stages
Source: PLoS Pathog. 2022 Oct 28;18(10):e1010926. doi: 10.1371/journal.ppat.1010926 (PMC9645663; doi:10.1371/journal.ppat.1010926)
Supplement: S3 Table — (PDF) [file ppat.1010926.s011.pdf]

S3 Table. Averaged log_2 fold change of the baseline peptide levels in the *pfcrt* -edited and field lines versus $Dd2^{Dd2crt}$.

Peptide	Mass Spec mode	$Dd2^{F145Icrt}$ $/Dd2^{Dd2crt}$	$Dd2^{G353Vcrt}$ $/Dd2^{Dd2crt}$	$Dd2^{M343Lcrt}$ $/Dd2^{Dd2crt}$	$Dd2^{3D7crt}$ $/Dd2^{Dd2crt}$	Dd2 $/Dd2^{Dd2crt}$	RF12 $/Dd2^{Dd2crt}$	RF7 $/Dd2^{Dd2crt}$
AAHLP;AHLPA	AAHLP;AHLPA_pos	-0.989	-2.397	1.124	3.316	-1.772	0.519	-0.645
PAVHAS	PAVHAS_pos	0.544	-5.296	2.729	-0.189	-3.797	0.253	
DGLAH	DGLAH_neg	0.335	-0.259	-1.631	1.699	1.452	0.761	0.208
LVVYP	LVVYP_neg	1.064	-2.927		2.032	1.166	-0.351	1.208
LSFP	LSFP_pos	-1.956		1.446	-0.509	-1.026	-0.236	0.904
HAHKLRV	HAHKLRV_pos	-2.537	-1.031	1.107	-1.539	-3.210		0.613
VNFKL	VNFKL_pos	-1.035	-0.688	-0.187	-1.204	-2.795	0.329	0.162
SPADKT	SPADKT_pos	0.431	0.998	0.269	-1.503	-2.072	0.948	1.298
VKAA	VKAA_pos	0.516	0.864	-0.236	-2.457	-2.050	0.870	0.509
LWGKV	LWGKV_pos	1.316	1.090	0.666	-4.209	-3.307	2.689	1.976
PKVK	PKVK_pos	0.877	-0.181	0.086	-1.926	-1.499	0.030	1.438
FKLLSH	FKLLSH_pos	1.502	0.652	1.043	-2.998	-5.306	0.831	-0.435
TNAVA;NALSA	TNAVA;NALSA_pos	0.707	0.101	0.242	-4.121	-5.660	0.974	0.114
PVQAAY	PVQAAY_pos	1.135	0.644	0.930		-3.134	-0.728	-0.153
VGGEALG	VGGEALG_pos	1.062	0.670	0.395	-1.209	-1.754	-0.186	0.409
WGKVNV;ALAHKY	WGKVNV;ALAHKY_pos	0.671	-0.041	1.989	-2.881	-4.012	-0.712	1.145
ALSALSD	ALSALSD_pos	-1.325	-0.082	-0.405	-3.012		-1.821	-0.514
ALGRLLVV	ALGRLLVV_pos		-0.257	-0.680	-3.768	0.132	-2.869	-0.929
GE;DA	GE;DA_pos	0.124	0.345	-0.684	-1.496	-0.518	-1.280	0.044
DLS	DLS_pos	0.320	0.528	-1.312	-2.316	-0.590	-1.639	0.170
KFLASV	KFLASV_pos	0.523	0.333	0.693	-3.557	-1.488	-0.216	0.685
FLAS	FLAS_pos	0.133	0.150	-0.824	-2.938	-0.870	0.079	-0.274
LASVSTV;ASVSTVL	LASVSTV;ASVSTVL_pos	-1.597	1.625	0.185	-9.587	-1.599	-0.212	0.679
AGVAN;GVANA	AGVAN;GVANA_pos	0.349	-0.091	-0.037	-4.024	0.184	-0.034	-0.192
AHKLRV	AHKLRV_pos	0.766	-0.169	0.822	-5.563		-1.076	-2.284
KSAVTAL	KSAVTAL_pos	1.108	0.148	0.342	-4.947	-3.588		-1.028
NF	NF_pos	0.256	1.308	-0.523	-2.677	-1.065	-0.376	-1.193
VLSPADK	VLSPADK_pos	-0.091		-0.116	-2.324	-0.966	0.167	-0.614
LT;TL	LT;TL_neg	-0.373	2.244	-0.135	-3.063	-0.842	-0.233	-1.044
VAGVANAL	VAGVANAL_pos	0.464	-0.949	0.848	-4.604	-0.140	-0.430	-3.525
AEA	AEA_pos	0.210	-0.132	0.111	-0.033	-0.936	-0.328	0.039
NAVAHV	NAVAHV_pos	1.284	-1.638	0.227	-3.487	-3.547	-2.466	-0.235
HGKKV	HGKKV_pos	0.364	-0.476	-1.037		-3.513	-3.959	1.120
WTQR	WTQR_pos	0.055	-1.979	-0.508	-2.974	-3.444	-3.700	0.787
FFE	FFE_pos	-0.213	-0.475	0.819	-1.426	-0.374	-0.912	0.583
PVNFKL	PVNFKL_pos	-5.026		-0.115	-3.087	-1.867	-1.681	-2.283
VKGHG	VKGHG_pos	-2.924	-2.129	-1.397	-1.235	-0.933	-0.258	
LAHL	LAHL_pos	-5.422	-5.114	-0.770		-3.520	-3.828	0.338
VKGHGKKV	VKGHGKKV_pos	-1.702	-0.225	-1.791		-2.125	-1.347	-3.383
DLSHG	DLSHG_pos	-0.729	-0.780	-0.699	-0.710	-1.192	-1.005	-0.586
TLAAHLP	TLAAHLP_pos	-0.604	-0.698	-0.734	-0.581	-1.692	-1.640	-1.223

LSH	LSH_pos	-0.821	-1.876	-0.895	-1.719	-3.164	-1.229	-1.104
KVADAL	KVADAL_pos	-2.979	-2.151	-1.477	-3.840	-5.167		-4.747
KVKAH	KVKAH_pos	-3.079	-1.754	-1.830	-5.437	-6.414	-2.257	-3.302
LAAHLPA	LAAHLPA_pos	-1.247	-1.688	-2.272	-3.776		-2.315	-3.612
KVGAHA	KVGAHA_neg	-1.485	-3.780	-2.215	-6.391	-4.401	-4.221	-5.409
LDKFLA	LDKFLA_pos	0.767	-0.280	0.265	-2.143	-1.195	-2.688	-1.245
VDPEN	VDPEN_neg	-0.078	0.560	3.425	-4.395	-4.490	-6.003	-3.232
KAHGK;AHGKK	KAHGK;AHGKK_pos	-0.240	-0.557	-0.415	-1.759	-1.900	-2.926	-1.897
QKVVAGV	QKVVAGV_pos	-0.623	0.490	-0.226		-1.464	-2.767	-1.604
DKTNVK	DKTNVK_pos	-1.286	-0.420	-0.258	-3.943		-1.831	-1.322
HKLRV	HKLRV_pos	-0.618	-1.057	-0.370	-5.586	-6.538	-2.355	-1.547
AHKLR	AHKLR_pos	-1.782	-1.578	-0.730	-3.312	-3.532	-3.438	-0.359
KGHGKKVA	KGHGKKVA_pos	-4.194	-0.428	0.578	-4.460	-3.659	-4.110	-3.320
GKVGAH;QVKGH	GKVGAH;QVKGH_neg	-2.912	-0.805	0.366	-4.091	-4.659	-3.943	-3.127
VAGVANAL	VAGVANAL_neg	-5.154	-3.517	-0.556	-7.518	-5.940	-7.444	-5.656
KAHGKK	KAHGKK_neg	-1.658	-1.199	1.454	-5.069	-4.126		-2.438
HKLRV	HKLRV_neg	-1.709	-1.462	0.680	-5.961	-3.696		-3.397
KGHGKK	KGHGKK_neg	-1.699	-0.874	0.355	-4.991	-3.592	-5.277	-2.215
AHKLR	AHKLR_neg	-2.783	-0.615	0.855	-3.661	-3.448	-5.601	-2.697
KLLS	KLLS_pos	-0.569	-1.224	-1.360	-3.091	-1.834	-0.124	-1.019
KVLG;KVVA	KVLG;KVVA_pos	-1.831	-1.107	-1.490	-2.235	-1.104	0.010	-0.699
TPPV	TPPV_pos	-5.635	-3.147	-9.619	-8.666	-0.563	-3.145	-0.069
HKLRVD	HKLRVD_pos	-0.962	-5.119	-2.426	-2.177	-1.646	-0.935	-4.423
AYQKVVAG	AYQKVVAG_pos	-1.053	-2.592	-1.496	-1.226	-0.375	-1.643	-0.027
ALWG	ALWG_pos	0.071	-1.261	-0.390	-0.869	-0.791	-0.793	-0.531
GNPK	GNPK_pos	0.909	-1.541	-0.606	-1.383	-0.663	-0.770	-0.303
GNPKVK	GNPKVK_pos	-0.678	-1.763	-1.530	-2.781	-1.635	-1.373	-0.635
VAGVAN	VAGVAN_neg	-0.651	-2.057	-2.309	-3.241	-1.461	-3.311	-1.274
NLK;QKV	NLK;QKV_pos	0.539	-1.122	-0.881	-1.394	0.200	-0.274	0.253
KA	KA_pos	-0.712	-2.612	-0.406	-1.767		-0.318	1.466
HCDK	HCDK_pos	0.002	-2.791	-0.545	-1.318	-0.952	1.650	
KLRVDPV	KLRVDPV_pos	0.387	-0.393	-1.746		-0.650	-0.514	-1.307
GAFS;AYQ	GAFS;AYQ_pos	0.978	-0.029	-1.112	2.014	-1.060	-0.922	-0.867
VLSP	VLSP_pos	0.705	0.892	-0.790	0.725	0.680	-0.555	0.747
TVLT	TVLT_pos	0.293	1.247	-1.324	-0.276	0.444	-0.945	-0.625
SFGDL;FGDLS;FSDGL	SFGDL;FGDLS;FSDGL_p	-0.079	0.912	-1.057	0.221	0.534	-0.844	-0.073
SFG	SFG_pos	0.322	0.823	-1.091	-0.361	-0.017	-1.246	0.077
HHFG	HHFG_pos	0.772	0.797	-1.229	-0.069	0.000	-0.938	0.535
SPA	SPA_pos	0.312	-0.278	0.137	-0.101	-0.514	-1.968	0.196
TKTY	TKTY_pos	-0.158	0.983	-0.962	-0.663	0.263	-2.344	-0.244
SVS	SVS_pos	0.929	1.159	-0.210	-0.357	0.205	-2.019	-0.018
HVDD	HVDD_pos	0.678	0.674	-0.622	-0.584	-0.014	-1.468	-0.451
PVQA	PVQA_pos	0.837	0.922	-0.618	-1.337	-0.966	-1.968	-0.095
VNVD	VNVD_pos	0.752	0.626	-0.618	-0.850	-0.629	-2.134	-0.249
LRVD	LRVD_neg	1.094	1.200	-1.352	-0.651	-1.606	-4.196	-1.120

SFPTTK;ASLDKF;DKFLAS	SFPTTK;ASLDKF;DKFLAS	-0.183	0.762	-1.971	-0.956	-1.578	-2.313	-1.016
EL	EL_pos	-0.010	-0.063	-1.113	-0.019	-0.611	-1.035	-0.512
SAQV;TNAV;NALS	SAQV;TNAV;NALS_pos	-0.353	0.174	-1.589	-0.551	0.236	-0.911	0.167
AL;LA	AL;LA_pos	-0.724	0.121	-2.561	-1.996	-0.424	-2.055	-1.006
GEALGRLL	GEALGRLL_pos	-1.147	0.177	-3.132		-0.041	-1.969	-1.779
YR	YR_pos	-0.869	-0.555	-2.442	-0.471	-0.085	-1.429	-0.792
VAGVAN	VAGVAN_pos	-0.272	-0.914	-1.991	-0.306	-0.326	-1.289	-0.505
VHASL	VHASL_pos	0.503	-0.719	-0.600	-1.449	0.179	-1.899	-0.542
DEVGG	DEVGG_neg	2.129	-0.059	-3.134	-3.660	-0.979	-8.203	-2.941
SFGDLS	SFGDLS_pos	0.426	-0.711	-0.923	-0.171	-0.089	-1.348	-0.550
VHASL	VHASL_neg	0.325	-1.144	-1.336	-0.164	0.184	-2.095	-0.818
DPE	DPE_neg	1.721	-1.000	-1.378	-0.283	-0.565	-3.176	-2.465
EE	EE_neg	0.713	-0.109	-1.346	0.182	-0.138	-1.528	-1.435
PTTK	PTTK_pos	-2.115	0.880	-1.659	0.014	1.389	-1.459	
KVKA	KVKA_pos	-3.793		-1.294	-0.667	0.476	-3.749	-1.044
VNV	VNV_pos	-1.880	-0.309	-0.398	-1.506	1.003	-2.258	
KVNV	KVNV_pos	-3.992	-0.159	-2.534		-0.970	-0.758	0.621
DKL	DKL_neg	-2.522	0.401	-2.475	-0.537	0.730	-0.167	-0.146
RLLVVYP	RLLVVYP_pos	-2.072	1.356	-3.581	-0.372	-0.841		1.784
ALSALSD	ALSALSD_neg	-0.346	0.366	-0.544	0.299	-0.202	-0.096	0.045
GKKVADA	GKKVADA_pos	0.645	-0.438	0.380	-0.964		1.012	0.734
VKA;LKG	VKA;LKG_pos	0.753	-0.425	0.680	-0.300	1.191		0.574
LTP	LTP_pos	1.320	0.280	2.560	-0.715	0.415	1.871	6.757
MPNA	MPNA_pos	1.894	0.328	0.653	0.907	-1.090	-0.543	0.110
TSKY	TSKY_pos	0.719	0.429	1.396	0.815	-1.242	-0.579	0.030
HCLLV	HCLLV_pos	2.447	3.380	1.891	1.147		2.686	3.806
AVTALW	AVTALW_pos	1.365	1.499	0.628	0.140	-3.394	1.812	2.018
TYF	TYF_pos	3.724	3.152	2.110	0.162	-2.691	0.991	2.791
PPV	PPV_pos	1.987	1.783	1.950	-0.235	-1.252	0.035	0.514
TVLTSK	TVLTSK_pos	1.584	1.400	0.824	1.173	-0.398	0.201	2.283
VDEV	VDEV_pos		2.590	1.129	2.627	0.499	1.482	2.942
CLL	CLL_pos	1.633	2.828	1.404	1.958	2.285	0.715	0.319
YFPH	YFPH_pos		6.450	4.510	5.055	7.716	2.398	1.665
RMF	RMF_pos	-0.340	7.682	3.715	4.545	6.447	3.519	
HFDLSHGSAQ	HFDLSHGSAQ_pos	3.388	5.279	3.681	2.178	2.043	-0.608	1.971
FG	FG_pos	1.657	3.912	1.463	1.818	1.573	0.402	1.471
HVDPENF	HVDPENF_pos	1.925	3.084	1.182	0.694	2.188	0.068	1.238
EVGG;VGGE;LDN;DNL	EVGG;VGGE;LDN;DNL_p	1.829	1.876	0.620	1.334	0.962	-0.094	1.425
LSALSDLH	LSALSDLH_pos	4.109	5.411	4.208	4.170	5.146	-0.292	3.416
NPK	NPK_pos	1.808	2.448	2.769	3.325	2.194		1.278
VC;CV	VC;CV_neg	1.181	1.583		1.510	1.026	-0.257	0.519
AHA;AAH	AHA;AAH_pos	4.603	5.463	5.296	3.625	3.961	4.736	4.843
EALGRL	EALGRL_pos	1.814	2.784	2.374	1.003		2.418	2.471
FLSFP	FLSFP_pos	5.914	4.849	4.437	2.213		3.356	3.522
LASVS	LASVS_pos	5.406	3.955	5.280	3.665	5.731	4.393	

NPKVK	NPKVK_pos	8.214	6.992	8.377	4.952	6.335	3.588	7.674
DKTNV	DKTNV_pos	5.008	4.268	3.963	4.273	3.270	2.768	3.245
VVY	VVY_pos		5.677	4.347	5.830	5.109	5.008	4.846
AA	AA_pos		1.941	2.494	1.066	3.535	3.125	2.206
HGKKV	HGKKV_neg	4.964	6.474	7.184	4.334	2.076	5.941	1.339
VCVLAH	VCVLAH_pos	7.025	6.101	8.341	3.352	1.790	4.403	
FPHFD	FPHFD_neg	5.080	3.851	4.919	1.529	1.418	2.383	0.718
VVAGVANA	VVAGVANA_pos	2.652	1.493	2.551	0.059	0.748	2.898	1.635
NVDEVGGEALG	NVDEVGGEALG_pos	2.033	3.067	1.147	0.214	0.173	1.838	1.008
SPADK	SPADK_pos	1.768	-0.209	1.086	0.346	-0.192	-1.134	0.589
TFATLS	TFATLS_pos	2.300	-0.660	2.423	1.729	1.205	-0.444	0.110
SP	SP_pos	3.825	3.148	0.987	0.682	-1.534	-2.132	2.813
KTNV;TNVK	KTNV;TNVK_pos	2.320	2.426	0.565	1.195	-0.019	-0.806	0.899
EVG;GDL;STP;DAV;DGL	EVG;GDL;STP;DAV;DGL_	4.111	3.502	2.330	1.216	0.785	-1.578	2.598
WTQ	WTQ_pos	3.159	1.783	0.108	0.794	0.522	-0.247	0.254
FPTT	FPTT_pos	2.532	0.892	1.451	1.668	0.586	-0.393	1.545
FTP	FTP_pos	3.239	1.760	1.752	1.278	0.959	-0.497	1.328
DPVNF	DPVNF_pos	3.247	1.699	2.381	1.675	1.051	0.181	1.970
DPEN	DPEN_pos	2.810	1.205	2.240	1.451	0.418	0.171	1.581
TPDAVMG	TPDAVMG_pos	3.163	1.084	2.208	1.281	1.130	0.513	1.402
DPENF	DPENF_pos	2.653	0.817	1.758	0.842	0.217	-0.914	0.954
TPPVQ	TPPVQ_pos	2.436	0.981	1.953	0.950	0.437	-0.759	0.485
YPWT	YPWT_pos	2.243	0.315	1.729	1.080	0.120	-0.949	0.751
LWGK	LWGK_pos	2.728	1.353	0.999	0.887	1.218	-1.822	0.600
TPDAVM	TPDAVM_pos	2.065	0.528	0.954	0.709	0.807	-1.433	0.802
YP	YP_pos	1.374	0.791	0.049	1.094	0.831	-0.739	0.839
KGHGK;GHGKK	KGHGK;GHGKK_neg	0.844	0.240	1.651	0.928	1.568	-2.163	2.834
KTYFP	KTYFP_pos	0.715	0.837	0.153	1.274	-0.303	-1.128	-0.832
DGLAHL;GLAHLD	DGLAHL;GLAHLD_pos	0.444	0.503	-0.708	1.216	0.269		-0.537
VKGH	VKGH_pos	1.758	4.253	0.546	2.526	3.831	-3.117	
DKL	DKL_pos	0.371	1.225	0.093	1.109	1.106	-0.918	-0.474
FF	FF_pos	2.298	4.102	-0.129	4.799	2.826		0.048
KLRVDP	KLRVDP_pos		2.293	-1.176	2.829	3.059	-1.937	-2.725
VLGAFSDG	VLGAFSDG_pos	0.434	0.930	-0.641	0.770	1.087	-0.731	-0.429
DE	DE_neg	-0.153	0.433	-1.028	0.701	0.485	-1.593	-1.306
ADALT	ADALT_pos	0.631	1.154	-0.190	0.376	0.060	-0.203	-0.392
HAGEYG	HAGEYG_pos	2.422	3.608	-0.954	0.746	2.213	0.228	-1.316
HF	HF_pos	2.143	4.176	2.377	1.359	1.594	-0.144	-1.150
HVDPENF	HVDPENF_neg	4.007	6.043	4.082	2.889	2.696	1.682	-2.746
NVDEVGGEALG	NVDEVGGEALG_neg	2.858	2.662	1.696	0.820	0.571	0.531	-2.927
ESFGDL	ESFGDL_pos	0.432	0.652	0.173	0.631	0.091	-0.088	-0.386
VDEVG	VDEVG_neg	7.315	6.623	2.854	4.136	-0.768	-1.533	-2.801
DGLAH	DGLAH_pos	0.054	0.142	0.560	1.711	1.480	0.242	-1.069
PAVH	PAVH_pos	3.274	1.836	0.813	-0.650	3.878		2.054
NVKAA;AQVKG	NVKAA;AQVKG_pos	1.895	1.564	1.108	-0.975	1.514		0.521

KEFT	KEFT_pos	1.699	1.521	1.145	-0.926	1.594		0.747
SFGD;FSDG	SFGD;FSDG_pos	1.194	1.643	0.705	-1.017	0.609	-0.778	0.883
GLAH	GLAH_pos	0.700	0.994	0.052	-0.542	1.146	-0.155	-0.116
SFPTT	SFPTT_pos	0.807	1.263	0.035	0.061	0.792	-0.422	0.466
HFG	HFG_pos	1.514	3.563	0.040	-0.037	0.598	-0.845	0.847
TF;FT	TF;FT_pos	0.861	2.694	-0.281	-1.337	0.288	-0.555	0.330
LT;TL	LT;TL_pos	1.992	2.754	0.120	-1.350	0.971	-0.023	0.654
VAH;AHV;AVH;VHA	VAH;AHV;AVH;VHA_pos	1.521	2.853	1.669	-0.356	-0.303	-0.513	3.067
HFD	HFD_pos	3.560	3.242	1.183	-1.653	-0.359	-3.213	2.500
HLDN	HLDN_pos	1.722	2.083	0.390	-0.999	-0.607	-1.191	1.164
LASVST	LASVST_pos	3.492	4.461	3.985	-0.756	-3.125	-3.103	2.858
GGEALG	GGEALG_pos	1.891	2.381	1.429	-0.871	-0.932	-1.881	1.454
DEVGGEA	DEVGGEA_pos	1.264	1.913	1.041	-1.310	-1.175	-0.880	1.625
EEK	EEK_pos	0.871	1.034	-0.303	-0.543	0.030	0.421	1.262
PAEF	PAEF_pos	2.066	1.774	-0.175	-0.893	-0.277	0.245	1.268
PNA	PNA_pos	2.375	1.679	0.835	-1.503	-0.489	0.304	0.811
NP	NP_pos	3.504	2.525	1.131	-1.623	-0.147	0.203	1.791
PADK	PADK_pos	2.356	2.095	0.514	-1.062	-0.100	-0.073	0.620
PW	PW_pos	2.959	2.316	0.112	-0.942	0.127	-0.624	1.070
PVN	PVN_pos	4.092	3.085	0.913	-2.422	-0.926	-1.032	3.563
TPA	TPA_pos	2.679	2.889	0.605	-1.641	-0.895	-0.925	2.786
PEEK	PEEK_pos	1.965	1.606	0.932	-1.487	-0.321	-1.167	2.024
PA	PA_pos	2.454	1.817	0.705	-2.019	-0.264	-0.260	2.437
SV;VS	SV;VS_pos	2.641	2.288	0.459	-1.536	0.101	-0.057	2.638
HG	HG_pos	2.441	1.823	0.033	-1.414	-0.095	-0.664	2.124
PDAV	PDAV_pos	1.574	1.626	-0.141	-1.111	-0.055	-0.549	1.632
PVQ	PVQ_pos	3.124	2.457	0.376	-1.436	-0.049	-0.443	2.133
VNVDE;NVDEV	VNVDE;NVDEV_pos	1.972	1.966	0.124	-1.186	0.267	-0.477	1.461
MP	MP_pos	3.258	2.814	1.214	-1.277	-0.274	-0.815	2.567
TPE	TPE_pos	3.476	3.710	1.336	-1.400	-1.057	-0.813	2.377
DEVGGEALG	DEVGGEALG_pos	2.847	2.847	1.499	-0.496	-0.149	-0.721	1.967
AHVD	AHVD_pos	3.809	4.164	1.430	0.757	0.988	1.248	2.281
ESFGDLS	ESFGDLS_pos	1.443	1.483	0.387	0.100	0.383	0.419	0.652
GAHAGE	GAHAGE_pos	1.396	1.732	0.754	0.152	0.163	-0.117	1.209
VDP	VDP_pos	1.882	2.276	0.711	-0.478	0.720	0.084	1.233
VG;GV	VG;GV_pos	4.075	4.046	1.498	-0.816	0.213	0.381	2.035
TQ	TQ_pos	2.657	2.934	0.887	-0.833	0.046	0.191	1.485
TP	TP_pos	3.041	2.149	0.528	-0.191	-0.184	0.008	1.688
PDAVM	PDAVM_pos	3.006	2.325	0.484	-0.181	0.084	0.288	0.742
EAL	EAL_pos	1.495	2.010	0.091	0.060	0.321	-0.758	1.283
CVLAH	CVLAH_pos	2.092	1.789	0.360	-0.423	0.611	-0.220	1.597
HK	HK_pos	2.809	2.915	0.376	-0.465	0.282	-0.863	1.909
VNVDEVG;EVGGEALG	VNVDEVG;EVGGEALG_p	1.891	1.327	0.252	-0.048	0.238	-0.442	1.101
NL;VQ	NL;VQ_pos	4.490	3.922	2.237	-0.684	0.748	1.539	5.279
AHLD	AHLD_pos	2.779	2.426	1.081	0.817	1.351	-0.011	2.387

PK	PK_pos	4.670	3.342	2.245	1.342	1.251	0.912	4.443
PWT	PWT_pos	4.176	3.222	1.181	0.260	1.048	0.533	3.687
LP	LP_pos	0.465	0.712	-0.468	-1.440	-0.814	-0.258	0.328
LST;TLS	LST;TLS_pos	0.738	1.331	-0.875	-2.065	-0.956	-0.881	1.113
GAFSD;AFSDG	GAFSD;AFSDG_pos	1.218	0.880	-0.428	-2.746	-2.698	-1.215	0.645
DKF	DKF_pos	1.893	1.913	0.461	-1.629	-0.470	-0.532	0.607
PHFD	PHFD_pos	2.451	2.675	0.532	-2.370	-1.235	-1.172	1.141
VD	VD_pos	1.436	1.190	0.173	-1.434	-0.488	-0.676	0.926
VDEVGGEALG	VDEVGGEALG_pos	1.724	1.512	0.729	-1.971	-0.709	-0.849	0.934
PV	PV_pos	2.841	2.369	0.580	-2.045	-0.229	-1.294	0.795
PEN	PEN_pos	2.307	2.574	0.857	-2.487	-0.487	-1.329	0.230
DNLK	DNLK_pos	1.748	1.586	0.056	-1.460	-0.506	-1.609	-0.057
FD	FD_pos	1.914	1.675	0.443	-1.783	-0.616	-1.802	0.683
TPD	TPD_pos	1.445	1.142	0.007	-1.697	-0.362	-1.234	0.521
PWTQ	PWTQ_pos	1.999	1.272	-0.469	-1.614	-0.490	-1.123	0.587
PEE	PEE_pos	1.818	0.705	0.027	-1.803	-0.388	-0.798	0.614
NVD	NVD_pos	1.350	1.185	0.360	-2.260	0.452	-0.548	0.936
PAE	PAE_pos	1.650	1.154	-0.289	-2.063	0.121	-0.961	0.776
VT;LS	VT;LS_pos	2.054	1.173	-0.374	-1.536	0.260	-0.760	0.411
VV	VV_pos	1.930	1.715	-0.592	-2.112	0.419	-0.398	0.467
PTT;DAL	PTT;DAL_pos	1.917	1.652	-0.148	-2.170	-0.226	-0.156	1.859
AV;LG;GL;VA	AV;LG;GL;VA_pos	1.927	1.903	-0.461	-1.804	0.326	-0.391	1.573
VM	VM_pos	1.391	1.701	-0.782	-2.242	0.124	-0.297	1.172
LV;VL	LV;VL_pos	0.706	1.484	-1.006	-2.005	0.236	-0.781	0.462
EV;DL;LD	EV;DL;LD_pos	1.070	1.095	-0.412	-2.017	-0.167	-1.361	-0.491
NVDE	NVDE_pos	0.681	1.283	-0.254	-1.922	-0.125	-1.544	-0.090
VNF	VNF_pos	1.991	2.064	-0.113	-2.635	-0.364	-0.435	0.482
PDA	PDA_pos	1.396	1.190	-0.073	-2.718	-0.343	-0.978	-0.072
VLG;VVA	VLG;VVA_pos	1.763	1.730	-0.100	-2.639	-0.372	-0.589	-0.509
EA	EA_pos	1.260	1.368	-0.221	-3.870	-1.073	-1.544	0.056
PE	PE_pos	0.877	0.772	0.006	-2.905	-0.387	-0.885	0.276
VDE;DEV	VDE;DEV_pos	1.137	1.245	-0.092	-3.405	-0.702	-1.056	0.756
RLLV	RLLV_pos	2.496	1.079	-0.014	-11.130	-2.993	-3.903	-0.270
HLD	HLD_pos	1.402	0.134	-0.315	-1.999	-0.677	-1.428	1.491
GDLS;SDGL	GDLS;SDGL_pos	1.253	0.513	-0.148	-1.787	-0.459	-2.366	0.515
STPDAVM	STPDAVM_pos	1.057	0.742	-0.020	-1.188	-0.832	-2.068	1.210
VGGEA	VGGEA_pos	0.776	0.213	0.501	-1.157	-1.402	-2.057	1.563
PVNF	PVNF_pos	1.396	1.374	-0.401	-0.737	0.295	-1.352	0.509
DALT	DALT_pos	0.913	1.392	-0.872	-0.659	-0.086	-0.785	0.212
DGLA	DGLA_pos	1.312	1.786	-0.898	-0.826	-0.426	-0.800	0.331
PAV	PAV_pos	2.090	1.806	-0.409	-0.565	-0.408	-0.616	0.812
PENF	PENF_pos	1.855	1.521	0.022	-0.530	-0.558	-0.294	0.950
DEVG;STPD	DEVG;STPD_pos	1.742	1.492	-0.489	-0.781	-0.585	-0.791	0.378
NVDEVG	NVDEVG_pos	1.066	1.494	-0.638	-0.011	-0.051	-0.429	0.876
YH	YH_pos	1.223	2.570	-0.349	-0.401	-0.565	-1.095	0.875

ESFG;AFSD	ESFG;AFSD_pos	0.732	0.702	-0.303	-0.232	-0.659	-1.040	0.472
VNVDEVGGEALG	VNVDEVGGEALG_pos	2.340	2.441	-0.776	-0.934	-1.018	-3.165	1.976
VLSPAD	VLSPAD_pos	0.608	1.172	-0.392	-0.033	-0.825	-0.413	-0.480
TFATL	TFATL_pos	1.289	1.270	-0.859	0.283	-0.987	-0.877	0.148
DGLA	DGLA_neg	3.058	3.594	-0.082	-0.716	-1.507	0.115	-0.471
FPH;PHF	FPH;PHF_pos	1.999	4.036		-1.032	2.445	-0.143	-2.279
TY	TY_pos	0.384	2.685	0.315	-0.234	1.123	-0.263	-2.205
FPH;PHF	FPH;PHF_neg	0.360	3.517	0.497	-1.227	1.160	-1.512	-2.052
TF;FT	TF;FT_neg	-0.506	3.363	-0.480	-2.037	0.183	-1.227	-0.520
PAE	PAE_neg	0.702	2.785	1.145	-2.470	0.556	-2.170	-1.287
TYFP	TYFP_neg	0.315	0.925	-1.003	-1.328	0.800	-0.683	-0.567
LSP	LSP_pos	1.345	3.872	0.378	-1.499	-0.447	0.103	-1.201
GKKV	GKKV_pos	0.998		0.054	-1.410	-0.762	-0.081	-0.966
EVGGEA	EVGGEA_pos	2.336	2.096	0.916	0.149	-0.125	-0.143	-0.719
MGNPK	MGNPK_pos	2.109	2.241	1.119	-0.020	0.136	-0.208	0.325
PENF	PENF_neg	4.744	4.381	1.925	-0.898	-0.132	-0.171	-0.146
PW	PW_neg	4.975	4.771	0.936	-0.644	1.787	-0.826	0.250
PWT	PWT_neg	5.601	4.978	0.938	-0.747	1.282	0.777	0.063
PDAVM	PDAVM_neg	3.356	4.191	0.856	-0.583	0.635	0.641	-1.353
PV	PV_neg	3.693	3.723	1.550	-2.061	2.323	-1.249	-1.892
TP	TP_neg	3.245	2.758	0.686	-0.412	1.013	-0.778	-1.680
VNVDEVGGEA	VNVDEVGGEA_neg	2.009	3.188	2.021	-0.561	0.327	-0.369	-1.509
PEEK	PEEK_neg	1.996	2.373	0.136	-1.535	0.560	-0.510	-1.900
NP	NP_neg	3.183	2.695	0.944	-1.805	0.431	-0.047	-1.449
VT;LS	VT;LS_neg	3.320	2.706	0.406	-2.171	0.145	0.284	-1.860
AV;LG;GL;VA	AV;LG;GL;VA_neg	3.572	3.423	0.396	-2.339	1.091	0.945	-0.918
SV;VS	SV;VS_neg	3.177	2.716	0.425	-2.131	0.911	0.600	-1.570
PADK	PADK_neg	1.854	1.779	0.755	-1.427	0.466	0.264	-0.690
PVQ	PVQ_neg	3.385	3.327	0.989	-2.123	1.110	-0.427	-0.998
VG;GV	VG;GV_neg	3.269	3.341	0.409	-3.327	0.834	-0.427	-1.530
PTT;DAL	PTT;DAL_neg	2.997	3.151	0.680	-2.505	0.810	-0.765	-1.140
PVN	PVN_neg	3.882	3.787	1.263	-3.022	-0.895	-0.748	-1.067
VN;NV;VGG	VN;NV;VGG_neg	3.080	2.248	0.337	-3.351	0.229	0.022	-1.899
PNA	PNA_neg	1.836	3.164	0.636	-3.595	-0.158	0.293	-1.481
PAEF	PAEF_neg	3.324	2.902	0.736	-3.504	0.847	0.617	0.028
KGHGK;GHGKK	KGHGK;GHGKK_pos	0.002	0.669	0.371	-1.723		-3.327	-0.592
LK;KL	LK;KL_pos	0.033	0.503	-0.001	-0.594	1.036	-1.038	-0.356
NVL	NVL_pos		1.797	2.381	-1.161	6.665	-3.584	-0.175
FK;KF	FK;KF_pos	1.955	0.628	1.534	-0.916	-0.149	-0.801	0.161
TPDA	TPDA_pos	1.797	0.447	1.856	-0.996	-0.324	-0.699	0.446
GKVGAHAG;AQVKGHG	GKVGAHAG;AQVKGHG_	4.545	2.851	3.962	-1.087	0.325	-2.298	1.867
TPEEK;LSTPDA	TPEEK;LSTPDA_pos	2.490	1.281	2.148	-0.620	0.711	-0.732	0.357
SPAD	SPAD_pos	1.529	-0.145	1.210	-0.591	-1.304	-0.940	0.247
PPVQ	PPVQ_pos	1.666	0.333	1.198	-1.584	-0.848	-1.882	-0.696
PPVQA	PPVQA_pos	2.012	0.876	1.116	-1.795	-0.731	-1.433	-0.292

TPEE	TPEE_pos	1.763	0.354	1.157	-0.632	-0.498	-1.822	0.263
DPVN	DPVN_pos	2.260	0.537	1.856	1.138	-0.861	-1.325	-0.566
TPAVH	TPAVH_neg	4.015	0.703	3.487	1.043	-0.067	-1.732	-2.283
NPKV	NPKV_pos	2.754	1.881	2.556	0.122	0.704	-1.131	-0.766
TPAVH	TPAVH_pos	2.996	2.766	2.214	1.083	0.229	-1.430	-0.937
DPVNF	DPVNF_neg	3.796	2.507	3.264	1.312	0.039	-2.124	-2.221
DPE	DPE_pos	0.942	-0.099	0.647	-0.059	-0.179	-0.987	-0.543
DPENF	DPENF_neg	5.319	3.712	4.931	1.524	-5.698	-6.260	-4.429
TPPVQ	TPPVQ_neg	3.669	2.625	3.600	0.312	-3.366	-3.872	-3.006
VKGHG	VKGHG_neg	2.035	2.343	4.042	0.378	-2.430	-2.903	-3.968
VAHVD	VAHVD_pos	0.086	0.600	0.208	-0.017	-0.297	-0.927	-0.367
DEVGGEA	DEVGGEA_neg	2.564	3.191	1.573	-1.079	-0.573	-6.346	-1.420
DPEN	DPEN_neg	1.009	0.950	1.687	0.039	-0.369	-2.846	-0.823
HVDPEN	HVDPEN_pos	1.684	3.276	0.825	-0.094	0.143	-2.266	-1.747
VDPVNF	VDPVNF_neg	0.984	2.208	0.942	-0.013	0.084	-1.877	-1.617
DNLK	DNLK_neg	2.059	2.258	0.266	-0.710	-0.424	-1.753	-1.799
TLSELHCDKL	TLSELHCDKL_neg	3.484	3.231	2.172	-1.579	0.261	-2.624	-2.893
TPEE	TPEE_neg	2.401	1.997	3.252	-3.849	-1.548	-3.059	-3.854
TPEEK;LSTPDA	TPEEK;LSTPDA_neg	1.067	1.368	4.006	-1.685	-1.255	-1.030	-1.545
LASVST	LASVST_neg	2.652	3.796	4.894	-3.495	-0.863	-3.188	-1.779
PAVH	PAVH_neg	0.554	1.950	1.464	-0.922	-1.203	-1.160	-1.287
PNALS	PNALS_pos	1.594	0.767	-0.738	-1.376	-0.826	-0.577	-0.382
CLLVTLA	CLLVTLA_pos	1.846	0.220	-1.048	-1.742	-4.021	-2.645	-1.964
PD;DP	PD;DP_pos	0.619	0.563	-0.368	-0.416	-0.708	-0.789	-0.839
GEA	GEA_pos	0.325	0.347	-0.005	-0.576	-0.690	-0.460	-0.489
KVGAHA	KVGAHA_pos	1.554	1.051	0.052	-1.135	-1.485	-1.599	-1.417
VDPEN	VDPEN_pos	1.942	1.593	0.904	-1.573	-1.542	-2.082	-2.352
TPEEKS	TPEEKS_pos	1.062	1.304	-0.031	-0.620	-0.744	-0.586	-0.335
NL;VQ	NL;VQ_neg	2.618	3.164	0.389	-2.088	-2.065	-2.175	-1.605
VMGNPKVK	VMGNPKVK_neg	2.246	3.793	-0.117	-2.992	-4.292	-3.573	0.283
TYFP	TYFP_pos	0.075	0.527	-0.600	-0.085	0.510	-0.554	-1.227
VTALWG	VTALWG_pos	0.046	0.714	-0.320	-1.315	0.541	-0.993	-4.236
FE;EF	FE;EF_pos	0.505	1.083	-1.101	-1.409	-0.327	-1.790	-1.404
SF;FS;AY	SF;FS;AY_pos	0.332	0.493	-1.211	-0.504	-0.345	-1.251	-1.428
VHL;LHV	VHL;LHV_pos	-0.189	0.057	-0.823	-0.668	-0.110	-1.297	-1.639
VHL;LHV	VHL;LHV_neg	-0.329	0.305	-1.965	-3.082	-0.889	-2.616	-2.940
VDD	VDD_neg	-1.374	1.001	0.002	-2.077	0.047	-2.365	-3.320
VDD	VDD_pos	0.802	0.487	-0.265	-1.888	-0.793	-1.394	-1.207
HL;LH	HL;LH_pos	0.840	0.351	-0.566	-2.072	-0.252	-1.196	-0.698
DLS	DLS_neg	0.588	1.035	-0.738	-2.993	0.181	-2.381	-2.118
EV;DL;LD	EV;DL;LD_neg	1.476	2.054	0.290	-3.906	-0.374	-3.180	-3.734
VDE;DEV	VDE;DEV_neg	1.452	1.897	0.509	-3.428	-0.538	-3.773	-3.958
PE	PE_neg	1.059	1.618	0.071	-3.844	-0.202	-1.899	-3.134
HLD	HLD_neg	1.194	0.240	-0.386	-2.502	-0.113	-2.301	-2.591
VDEVGGEALG	VDEVGGEALG_neg	1.408	0.541	0.250	-4.307	-0.749	-2.380	-4.993

AHLD	AHLD_neg	1.562	2.785	0.480	-1.413	-0.841	-0.643	-2.750
KTNV;TNVK	KTNV;TNVK_neg	0.904	2.686	0.018	-0.835	0.150	-0.628	-1.966
PWTQ	PWTQ_neg	1.747	2.537	-0.041	-2.071	-0.493	-1.568	-2.249
SA;GT	SA;GT_neg	1.054	1.303	-0.238	-1.794	0.091	-1.062	-1.536
VD	VD_neg	1.900	2.403	0.792	-1.849	0.076	-1.544	-2.533
PDA	PDA_neg	2.612	2.984	1.536	-2.928	-0.419	-1.840	-3.304
GDLS;SDGL	GDLS;SDGL_neg	2.066	2.674	1.284	-2.312	-0.091	-1.817	-2.559
PEE	PEE_neg	1.785	1.828	0.183	-1.989	0.270	-1.080	-2.458
PA	PA_neg	2.274	2.099	0.719	-2.746	0.260	-1.323	-2.274
PVQA	PVQA_neg	1.463	2.291	0.421	-2.538	-0.441	-1.771	-4.267
PNALS	PNALS_neg	2.809	2.906	0.687	-3.453	-1.216	-1.492	-4.473
LV;VL	LV;VL_neg	1.027	1.880	-0.646	-2.144	-0.557	-0.254	-1.522
VV	VV_neg	1.853	1.829	-1.002	-3.002	-0.498	-0.473	-1.845
VNF	VNF_neg	1.734	2.824	0.262	-4.727	-0.378	-1.131	-2.454
SFG	SFG_neg	0.435	1.409	-0.395	-0.108	0.109	-1.957	-1.657
ESFG;AFSD	ESFG;AFSD_neg	1.495	1.817	0.645	-0.170	-0.338	-2.725	-2.791
DEVGGEALG	DEVGGEALG_neg	3.235	3.218	2.327	-1.524	-1.793	-3.555	-5.334
GGEAL;GEALG	GGEAL;GEALG_neg	0.368	1.900	0.561	-0.829	-0.957	-2.599	-2.737
PPVQA	PPVQA_neg	0.900	1.613	1.443	-2.188	-0.793	-2.893	-3.338
PPVQ	PPVQ_neg	1.397	1.610	1.310	-2.054	-0.975	-3.036	-3.624
VNVDEVG;EVGGEALG	VNVDEVG;EVGGEALG_n	1.815	1.849	0.762	-1.554	-0.401	-3.178	-2.342
PHFD	PHFD_neg	2.678	3.508	1.504	-3.262	0.286	-4.853	-4.537
SFGD;FSDG	SFGD;FSDG_neg	1.191	1.893	-0.580	-1.581	-0.893	-2.145	-4.520
NVDEVG	NVDEVG_neg	0.534	0.924	-0.878	-1.149	0.081	-2.233	-3.012
PD;DP	PD;DP_neg	0.482	1.190	-0.193	-0.708	0.032	-1.711	-2.773
STPDAVM	STPDAVM_neg	1.078	1.354	0.176	-2.062	-0.731	-4.225	-5.754
HVDD	HVDD_neg	0.492	1.377	0.138	-1.142	-0.366	-2.865	-3.325
DALT	DALT_neg	0.834	1.475	-0.245	-2.170	-0.223	-2.133	-3.700
PVNF	PVNF_neg	0.865	1.377	-0.175	-1.500	-0.143	-2.389	-2.665
ADALT	ADALT_neg	0.853	0.524	-0.050	0.728	0.106	-2.365	-2.740
HFDLSHGSAQ	HFDLSHGSAQ_neg	1.475	1.590	0.094	0.647	-0.456	-4.491	-8.834
EL	EL_neg	-0.050	1.046	-0.011	0.337	-0.358	-0.376	-0.442
HFGKEFTPP	HFGKEFTPP_neg	-0.748	2.143	1.436	-0.285	-1.552	-1.344	-1.147
VDEVGG	VDEVGG_neg	-1.991	3.414	0.841	-1.928	-1.686	-2.244	-2.604
ESFGDLSTP	ESFGDLSTP_pos		-0.341	-0.167	0.085	0.408	0.247	-0.661
FFESFGDL	FFESFGDL_neg	5.698	1.139	-1.773	-3.501	1.350	-1.167	-5.430
DPVN	DPVN_neg	4.072	-1.009	-1.022	1.242	0.835	-1.647	-4.123

393 peptides were detected in either positive or negative mode. Blank spaces denote missing or undetectable values.
