# Supplementary material for: Piperaquine-resistant PfCRT mutations differentially impact drug transport, hemoglobin catabolism and parasite physiology in Plasmodium falciparum asexual blood stages
Source: PLoS Pathog. 2022 Oct 28;18(10):e1010926. doi: 10.1371/journal.ppat.1010926 (PMC9645663; doi:10.1371/journal.ppat.1010926)
Supplement: S4 Table — (PDF) [file ppat.1010926.s012.pdf]

S4 Table. List of peptides showing significantly different levels in Dd2^{F145Icrt} or Dd2^{G353Vcrt} lines compared with Dd2^{Dd2crt}. For each peptide, the length, number of isoforms, isoelectric point (IEP) and charge are shown.

Peptides	Length	No. of isoforms	IEP	Charge at pH 5.5	Charge at pH 7.4	P value	q value	Mean of log$_2$-transformed peak area of Dd2^{Dd2crt}	Mean of log2-transformed peak area of Dd2^{F145I}	Difference in log$_2$-transformed mean peak areas	SE of difference	Fold change relative to Dd2^{Dd2crt}
DPENF	5	1	3.52	-1.9	-2	<0.001	0.000	6.94	13.58	6.64	1.27	99.66
PW	2	1	5.99	0	0	<0.001	0.000	6.90	13.34	6.44	1.27	86.82
PWT	3	1	5.95	0	0	<0.001	0.000	6.56	12.72	6.16	1.27	71.41
PENF	4	1	3.82	-0.9	-1	<0.001	0.000	7.19	13.33	6.14	1.27	70.62
PV	2	1	5.94	0	0	<0.001	0.000	7.45	13.56	6.11	1.27	68.83
PAEF	4	1	3.86	-0.9	-1	<0.001	0.000	5.59	11.52	5.93	1.27	60.93
TPAVH	5	1	7.07	0.6	0	<0.001	0.000	5.99	11.88	5.89	1.27	59.22
NPKVK	5	1	8.18	2	1.9	<0.001	0.000	5.21	13.28	8.07	1.79	268.91
FPHFD	5	1	6.09	-0.4	-1	<0.001	0.001	1.01	6.57	5.55	1.27	46.95
VDEVG	5	1	3.55	-1.9	-2	<0.001	0.001	3.90	9.35	5.45	1.27	43.83
FFESFGDL	8	1	3.58	-1.9	-2	<0.001	0.001	4.67	9.94	5.28	1.27	38.83
TYF	3	1	5.82	0	0	<0.001	0.004	7.44	12.33	4.89	1.27	29.57
VAGVANAL	8	1	5.82	0	0	<0.001	0.005	12.66	7.29	-5.37	1.42	0.02
PAVH	4	1	7.16	0.6	0	<0.001	0.006	7.72	12.46	4.74	1.27	26.74
NL;VQ	2	2	5.821;5.886	0;0	0;0	<0.001	0.011	5.43	10.48	5.05	1.42	33.22
DGLA	4	1	3.63	-1	-1	<0.001	0.011	7.54	12.06	4.51	1.27	22.83
HFD	3	1	5.24	-0.4	-1	<0.001	0.012	8.47	12.91	4.44	1.27	21.72
PK	2	1	8.07	1	1	<0.001	0.012	7.24	12.19	4.95	1.42	30.97
VG;GV	2	2	5.827;5.823	0;0	0;0	<0.001	0.013	11.00	15.38	4.39	1.27	20.92
VCVLAH	6	1	7.24	0.6	-0.4	<0.001	0.015	3.80	9.91	6.12	1.79	69.41
DKTNV	5	1	5.97	0	0	<0.001	0.017	4.62	9.40	4.77	1.42	27.32
PVN	3	1	5.89	0	0	0.001	0.023	12.36	16.47	4.10	1.27	17.20
EVG;GDL;STP;DAV;DGL	3	5	3.764;3.676;5.795;3.631;3.633	-0.9;-1;0;-1;-1	-1;-1;0;-1;-1	0.001	0.024	7.90	11.98	4.08	1.27	16.92
FLSFP	5	1	5.56	0	0	0.002	0.029	8.24	14.19	5.95	1.90	61.91
PTT;DAL	3	2	5.945;3.64	0;-1	0;-1	0.002	0.029	8.65	12.61	3.97	1.27	15.64
HFDLSHGSAQ	10	1	6.21	0.2	-1	0.003	0.041	4.87	8.68	3.81	1.27	14.05
AHVD	4	1	6.40	-0.4	-1	0.003	0.041	7.84	11.63	3.79	1.27	13.83
KVKAH	5	1	8.16	2.6	2	0.003	0.041	13.69	9.45	-4.23	1.42	0.05
MP	2	1	5.90	0	0	0.003	0.046	8.22	11.95	3.73	1.27	13.22
FTPAV	5	1	5.84	0	0	0.003	0.046	15.19	8.76	-6.43	2.19	0.01
DPVN	4	1	3.59	-1	-1	0.005	0.058	7.23	10.83	3.60	1.27	12.12
PVNFKL	6	1	8.02	1	1	0.005	0.059	10.40	6.02	-4.38	1.55	0.05
DEVGGEALG	9	1	3.47	-2.8	-3	0.006	0.070	7.37	10.86	3.49	1.27	11.22
HVDPENF	7	1	4.22	-1.3	-2	0.006	0.070	3.82	7.31	3.49	1.27	11.21
TPE	3	1	3.93	-0.9	-1	0.006	0.070	10.06	13.54	3.48	1.27	11.13
LASVS	5	1	5.67	0	0	0.006	0.070	5.41	10.59	5.19	1.90	36.40
DPVNF	5	1	3.59	-1	-1	0.007	0.071	8.33	11.77	3.44	1.27	10.87
TPDAVMG	7	1	3.64	-1	-1	0.007	0.073	5.90	9.31	3.41	1.27	10.65
LASVST	6	1	5.70	0	0	0.007	0.073	8.07	11.48	3.41	1.27	10.64
NP	2	1	5.89	0	0	0.007	0.073	12.64	16.05	3.40	1.27	10.59
SP	2	1	5.86	0	0	0.008	0.079	8.58	12.34	3.76	1.42	13.55
NVDEVGGEALG	11	1	3.50	-2.8	-3	0.008	0.080	8.33	11.67	3.35	1.27	10.19
AV;LG;GL;VA	2	4	5.899;5.765;5.768;5.897	0;0;0;0	0;0;0;0	0.009	0.085	10.12	13.42	3.30	1.27	9.87
VT;LS	2	2	5.897;5.839	0;0	0;0	0.010	0.087	9.63	12.92	3.29	1.27	9.75
TPPV	4	1	5.88	0	0	0.010	0.088	11.13	7.12	-4.01	1.55	0.06
TLSELHCDKL	10	1	5.82	-0.3	-1.4	0.011	0.093	9.26	12.49	3.24	1.27	9.42
FTP	3	1	5.78	0	0	0.011	0.093	8.40	11.63	3.23	1.27	9.39
TPPVQ	5	1	5.91	0	0	0.012	0.098	6.50	9.70	3.20	1.27	9.20
HGKKV	5	1	8.16	2.6	2	0.013	0.103	9.17	13.90	4.73	1.90	26.58
HKLRVD	6	1	7.83	1.6	1	0.013	0.105	7.16	3.66	-3.50	1.42	0.09
LAHL	4	1	7.06	0.6	0	0.013	0.105	10.64	5.22	-5.42	2.19	0.02
PVQ	3	1	5.87	0	0	0.014	0.110	14.16	17.26	3.11	1.27	8.60
WTQ	3	1	5.86	0	0	0.015	0.112	8.26	11.35	3.09	1.27	8.50
PDAVM	5	1	3.64	-1	-1	0.017	0.129	10.17	13.18	3.02	1.27	8.08
DPEN	4	1	3.53	-1.9	-2	0.018	0.131	10.43	13.43	3.00	1.27	8.01
KGHGKK	6	1	8.21	3.6	2.9	0.018	0.131	13.00	9.66	-3.35	1.42	0.10
EVGGEA	6	1	3.59	-1.8	-2	0.019	0.133	8.35	11.33	2.98	1.27	7.89
PNALS	5	1	5.90	0	0	0.019	0.135	10.43	13.40	2.97	1.27	7.81
TPEE	4	1	3.67	-1.8	-2	0.020	0.136	10.62	13.58	2.96	1.27	7.76
TP	2	1	5.87	0	0	0.022	0.142	13.96	16.86	2.91	1.27	7.50
HK	2	1	7.99	1.6	1	0.023	0.146	9.24	12.13	2.89	1.27	7.41

Data were obtained were three independent experiments with technical triplicates. P values were calculated from unpaired t tests. q values represent the False Discovery Rate (FDR), as calculated using the two-stage step-up method of Benjamini, Krieger and Yekutieli.

Peptides	Length	No. of isoforms	IEP	Charge at pH 5.5	Charge at pH 7.4	P value	q value	Mean of log2-transformed peak area of Dd2^{Dd2crt}	Mean of log2-transformed peak area of Dd2^{G353V}	Difference in log_2-transformed mean peak areas	SE of difference	Fold change relative to Dd2^{Dd2crt}
PV	2	1	5.94	0	0	<0.001	0.028	7.45	13.84	6.39	1.62	83.75
PW	2	1	5.99	0	0	<0.001	0.028	6.90	13.22	6.32	1.62	79.84
PENF	4	1	3.82	-0.9	-1	<0.001	0.080	7.19	12.90	5.71	1.62	52.42
PAEF	4	1	3.86	-0.9	-1	<0.001	0.084	5.59	11.12	5.53	1.62	46.30
VMGNPKVK	8	1	8.16	2	1.9	<0.001	0.084	4.72	10.08	5.36	1.62	40.93
PWT	3	1	5.95	0	0	0.001	0.084	6.56	11.87	5.32	1.62	39.86
RMF	3	1	8.61	1	1	0.001	0.104	8.91	16.64	7.73	2.42	212.90

Gray highlighting indicates peptides with altered abundance in both Dd2^{F145Icrt} and Dd2^{G353V}, compared with Dd2^{Dd2crt}.

Peptides	Length	No. of isoforms	IEP	Charge at pH 5.5	Charge at pH 7.4	P value	q value	Mean log_{10} of peak area of Dd2^{Dd2crt}	Mean log_{10} of peak area of Dd2^{M343L}	log_{10} Difference	SE of difference	Fold change relative to Dd2^{Dd2crt}
No significant differences were observed.												
