# Supplementary material for: Piperaquine-resistant PfCRT mutations differentially impact drug transport, hemoglobin catabolism and parasite physiology in Plasmodium falciparum asexual blood stages
Source: PLoS Pathog. 2022 Oct 28;18(10):e1010926. doi: 10.1371/journal.ppat.1010926 (PMC9645663; doi:10.1371/journal.ppat.1010926)
Supplement: S5 Table — (PDF) [file ppat.1010926.s013.pdf]

S5 Table. Differentially expressed genes identified from one-way ANOVA of Dd2^{F145Icrt}, Dd2^{G353Vcrt}, Dd2^{M343Lcrt} and the combined set of Dd2^{Dd2crt} and Dd2 ($p<0.05$; with Bonferroni corrections and permutations).

Gene	Description	Dd2 repA	Dd2 repB	Dd2^{Dd2crt} repA	Dd2^{Dd2crt} repB	Dd2^{M343Lcrt} repA	Dd2^{M343Lcrt} repB	Dd2^{M343Lcrt} repC	Dd2^{F145Icrt} repA	Dd2^{F145Icrt} repB	Dd2^{F145Icrt} repC	Dd2^{G353Vcrt} repA	Dd2^{G353Vcrt} repB	Dd2^{G353Vcrt} repC
PF3D7_0418700	PF3D7_0418700::RNA-binding protein NOB1, putative	0.389	-0.121	0.179	-0.211	0.089	-0.421	-0.521	0.459	1.579	0.489	-0.651	-0.911	-0.351
PF3D7_1013500	PF3D7_1013500::phosphoinositide-specific phospholipase C	-0.156	-0.056	-0.086	-0.016	0.144	0.014	-0.026	0.144	0.374	0.254	-0.316	-0.146	-0.126
PF3D7_0818500	PF3D7_0818500::zinc finger protein, putative	-0.027	0.263	0.003	-0.217	-0.307	-0.427	-0.467	0.213	0.773	0.213	0.163	-0.007	-0.177
PF3D7_0403900	PF3D7_0403900::SET domain protein, putative	-0.088	-0.108	-0.208	-0.068	-0.188	-0.518	-0.208	0.282	0.442	0.532	0.102	0.182	-0.148
PF3D7_1350500	PF3D7_1350500::conserved Plasmodium protein, unknown function	-0.137	-0.197	-0.437	-0.257	0.023	-0.107	0.053	0.063	0.503	0.363	0.083	0.113	-0.067
PF3D7_1232500	PF3D7_1232500::CG2-related protein, putative	-0.032	-0.342	-0.802	-0.532	-0.212	-0.552	-0.162	0.718	1.578	0.538	0.068	0.108	-0.382
PF3D7_1467900	PF3D7_1467900::rab GTPase activator, putative	-0.279	-0.189	-0.379	-0.369	-0.019	-0.319	-0.229	0.421	1.181	0.471	0.041	-0.089	-0.239
PF3D7_0629900	PF3D7_0629900::sec14-like cytosolic factor or phosphatidylinositol/phosphatidylcholine transfer protein,	0.032	0.112	0.202	0.302	-0.048	0.012	0.092	-0.168	-0.508	-0.098	0.002	0.052	0.012
PF3D7_0425000	PF3D7_0425000::Plasmodium exported protein, unknown function, pseudogene	1.559	1.849	1.389	0.899	0.159	-0.291	-0.991	-1.061	-0.771	-1.591	-0.441	-0.241	-0.471
PF3D7_0610500	PF3D7_0610500::peptidyl-tRNA hydrolase PTRHD1, putative	-0.035	0.675	0.495	0.645	0.405	0.065	-0.045	-0.465	-0.905	-0.645	-0.055	-0.075	-0.055
PF3D7_0309600	PF3D7_0309600::60S acidic ribosomal protein P2	0.273	0.593	0.393	0.453	0.193	0.193	-0.107	-0.417	-0.577	-0.467	-0.327	-0.147	-0.057
PF3D7_1038200	PF3D7_1038200::conserved Plasmodium protein, unknown function	0.206	0.946	0.526	0.306	0.216	-0.024	-0.164	-0.304	-0.634	-0.494	-0.204	-0.244	-0.134
PF3D7_0308500	PF3D7_0308500::activator of Hsp90 ATPase, putative	0.998	0.608	0.798	0.408	0.298	0.158	-0.122	-0.592	-0.862	-0.722	-0.562	-0.292	-0.122
PF3D7_0310300	PF3D7_0310300::phosphoglycerate mutase, putative	0.289	0.249	0.249	0.139	0.049	0.019	-0.071	-0.301	-0.281	-0.271	-0.001	-0.121	0.049
PF3D7_1228900	PF3D7_1228900::conserved protein, unknown function	0.364	0.474	0.684	0.244	0.184	0.254	-0.066	-0.686	-0.776	-0.566	-0.096	-0.196	0.184
PF3D7_1126400	PF3D7_1126400::mediator of RNA polymerase II transcription subunit 21, putative	0.270	0.560	0.340	0.200	0.090	0.170	0.070	-0.560	-0.320	-0.500	-0.180	-0.200	0.060
PF3D7_0706400	PF3D7_0706400::60S ribosomal protein L37	0.308	0.688	0.178	0.128	0.158	0.398	0.058	-0.462	-0.602	-0.332	-0.332	-0.242	0.058
PF3D7_1122000	PF3D7_1122000::conserved protein, unknown function	0.208	0.908	0.388	0.398	0.338	0.758	0.308	-0.832	-0.952	-0.572	-0.532	-0.522	0.098
PF3D7_0313100	PF3D7_0313100::ubiquitin-protein ligase, putative	0.024	0.354	0.304	0.194	0.134	0.374	0.124	-0.296	-0.906	-0.356	-0.056	-0.066	0.174
PF3D7_0933600	PF3D7_0933600::mitochondrial-processing peptidase subunit beta, putative	0.238	0.328	0.428	0.228	0.048	0.438	0.108	-0.242	-1.082	-0.392	-0.052	-0.132	0.088
PF3D7_0807000	PF3D7_0807000::YEATS domain-containing protein, putative	0.143	0.363	0.163	0.333	0.243	0.603	0.193	-0.267	-0.937	-0.457	-0.197	-0.257	0.073
PF3D7_1308200	PF3D7_1308200::carbamoyl phosphate synthetase	0.374	0.424	0.354	0.274	0.244	0.344	0.194	-0.436	-0.846	-0.326	-0.396	-0.386	0.184
PF3D7_1132200	PF3D7_1132200::T-complex protein 1 subunit alpha	0.324	0.374	0.654	0.334	0.354	0.404	0.034	-0.546	-0.976	-0.486	-0.406	-0.276	0.214
PF3D7_1424100	PF3D7_1424100::60S ribosomal protein L5, putative	0.418	0.548	0.588	0.308	0.258	0.428	-0.072	-0.522	-1.052	-0.432	-0.392	-0.112	0.038
PF3D7_1323100	PF3D7_1323100::60S ribosomal protein L6, putative	0.111	0.291	0.421	0.361	0.151	0.401	-0.019	-0.259	-0.669	-0.459	-0.179	-0.169	0.021
PF3D7_1459700	PF3D7_1459700::pyridoxal 5'-phosphate synthase, putative	0.288	0.428	0.548	0.568	0.258	0.498	0.018	-0.502	-0.962	-0.492	-0.302	-0.302	-0.052
PF3D7_1226500	PF3D7_1226500::heptatricopeptide repeat and RAP domain-containing protein, putative	0.169	0.219	0.309	0.059	0.079	0.259	0.179	-0.291	-0.351	-0.371	-0.091	-0.191	0.019
PF3D7_0520600	PF3D7_0520600::bis(5'-nucleosyl)-tetraphosphatase [asymmetrical]	0.328	0.528	0.208	0.308	0.208	0.438	0.128	-0.712	-0.782	-0.672	-0.022	-0.112	0.148
PF3D7_1360400	PF3D7_1360400::conserved Plasmodium protein, unknown function	0.215	0.615	0.375	0.315	0.225	0.335	0.135	-0.475	-0.835	-0.645	-0.125	-0.255	0.115
PF3D7_1427800	PF3D7_1427800::exosome complex component RRP41	0.069	0.489	0.499	0.289	0.129	0.209	0.289	-0.721	-0.851	-0.631	0.089	-0.061	0.199
PF3D7_0605000	PF3D7_0605000::50S ribosomal protein L24, putative	0.160	0.260	0.570	0.460	0.050	0.100	0.110	-0.380	-0.610	-0.600	0.150	-0.250	-0.020
PF3D7_1364800	PF3D7_1364800::DNA-directed RNA polymerases I, II, and III subunit RPABC1, putative	0.396	0.306	0.936	0.626	0.136	0.146	0.186	-0.794	-0.934	-0.794	-0.354	-0.254	0.396
PF3D7_1205600	PF3D7_1205600::tetratricopeptide repeat protein, putative	0.029	0.259	0.479	0.199	0.279	0.079	0.099	-0.261	-1.011	-0.311	0.039	-0.071	0.189
PF3D7_0614500	PF3D7_0614500::60S ribosomal protein L19	0.097	0.667	0.947	0.367	0.137	-0.013	-0.013	-0.413	-1.083	-0.523	-0.033	-0.283	0.147
PF3D7_1309100	PF3D7_1309100::60S ribosomal protein L24, putative	0.138	0.558	0.828	0.388	0.248	-0.102	-0.162	-0.432	-0.802	-0.492	-0.112	-0.152	0.088
PF3D7_1445700	PF3D7_1445700::conserved Plasmodium protein, unknown function	0.124	0.234	0.884	0.324	0.154	0.084	-0.046	-0.506	-0.866	-0.376	-0.056	-0.186	0.234
PF3D7_1247100	PF3D7_1247100::conserved protein, unknown function	0.115	0.555	0.235	0.075	-0.135	-0.055	-0.045	-0.355	-0.515	-0.175	0.105	0.105	0.085
PF3D7_1346500	PF3D7_1346500::conserved Plasmodium protein, unknown function	0.119	0.899	0.459	0.309	-0.021	0.019	-0.101	-0.471	-0.911	-0.401	0.059	-0.121	0.159

Shown are the mean-centered log_2 ratios of each sample against a 3D7 reference pool.
