# Supplementary material for: Piperaquine-resistant PfCRT mutations differentially impact drug transport, hemoglobin catabolism and parasite physiology in Plasmodium falciparum asexual blood stages
Source: PLoS Pathog. 2022 Oct 28;18(10):e1010926. doi: 10.1371/journal.ppat.1010926 (PMC9645663; doi:10.1371/journal.ppat.1010926)
Supplement: S6 Table — (PDF) [file ppat.1010926.s014.pdf]

S6 Table. List of differentially expressed genes in Dd2^{F145Icrt}, Dd2^{G353Vcrt} and Dd2^{M343Lcrt}, compared with the combined set of Dd2^{Dd2crt} and Dd2 (p<0.05; based on Student's t-test with Bonferroni corrections and permutations).

Gene	Description	Dd2 repA	Dd2 repB	Dd2^{Dd2crt} repA	Dd2^{Dd2crt} repB	Dd2^{M343Lcrt} repA	Dd2^{M343Lcrt} repB	Dd2^{M343Lcrt} repC	Dd2^{G353Vcrt} repC	Dd2^{G353Vcrt} repB	Dd2^{G353Vcrt} repA	Dd2^{F145Icrt} repA	Dd2^{F145Icrt} repB	Dd2^{F145Icrt} repC	Averaged Dd2	Averaged Dd2^{M343Lcrt}	Averaged Dd2^{G353Vcrt}	Averaged Dd2^{F145Icrt}	Log_2 fold change of Dd2^{F145Icrt}/ Dd2^{Dd2crt}	Fold change of Dd2^{F145Icrt}/ Dd2^{Dd2crt}
PF3D7_020780(	PF3D7_0207800::serine repeat antigen 3	-0.502	-0.392	-0.042	-0.062	0.248	-0.372	0.058	0.158	0.558	-0.472	0.458	0.188	0.168	-0.249	-0.022	0.082	0.272	0.521	1.435
PF3D7_020410(	PF3D7_0204100::Sel1 repeat-containing protein, putative	-0.068	-0.268	-0.438	-0.218	-0.008	-0.178	0.112	0.002	0.392	-0.468	0.392	0.342	0.402	-0.248	-0.024	-0.024	0.379	0.627	1.544
PF3D7_020960(	PF3D7_0209600::transporter, putative	-0.079	-0.139	-0.429	-0.209	-0.069	-0.219	0.161	0.051	0.271	-0.349	0.441	0.351	0.221	-0.214	-0.043	-0.009	0.337	0.552	1.466
PF3D7_050810(	PF3D7_0508100::SET domain protein, putative	-0.103	-0.363	-0.593	-0.293	-0.073	-0.353	0.297	0.037	0.397	-0.413	0.557	0.427	0.477	-0.338	-0.043	0.007	0.487	0.825	1.772
PF3D7_102990(	PF3D7_1029900::ORC3 domain-containing protein, putative	-0.165	-0.215	-0.485	-0.335	-0.025	-0.295	0.315	0.005	0.385	-0.135	0.365	0.175	0.415	-0.300	-0.002	0.085	0.318	0.618	1.535
PF3D7_052940(	PF3D7_0529400::conserved Plasmodium protein, unknown function	-0.153	-0.033	-0.263	-0.123	-0.133	-0.123	0.037	-0.093	0.187	-0.083	0.197	0.247	0.337	-0.143	-0.073	0.004	0.260	0.403	1.323
PF3D7_041940(	PF3D7_0419400::conserved Plasmodium protein, unknown function	-0.425	-0.295	-0.515	-0.095	0.015	-0.135	0.215	0.095	0.445	-0.345	0.445	0.285	0.315	-0.333	0.031	0.065	0.348	0.681	1.603
PF3D7_061380(	PF3D7_0613800::AP2 domain transcription factor, putative	-0.592	-0.532	-0.382	-0.162	0.088	-0.212	0.308	0.158	0.448	-0.202	0.518	0.218	0.338	-0.417	0.062	0.135	0.358	0.775	1.711
PF3D7_113840(	PF3D7_1138400::guanylyl cyclase alpha	-0.483	-0.613	-0.363	-0.093	0.097	-0.213	0.127	0.197	0.317	-0.133	0.497	0.247	0.417	-0.388	0.004	0.127	0.387	0.775	1.711
PF3D7_080550(	PF3D7_0805500::conserved Plasmodium protein, unknown function	-0.208	-0.048	-0.388	-0.028	-0.018	-0.088	0.092	0.042	0.192	-0.098	0.152	0.102	0.302	-0.168	-0.005	0.045	0.185	0.353	1.278
PF3D7_091990(	PF3D7_0919900::regulator of chromosome condensation-PP1-interactin	-0.325	-0.045	-0.405	-0.125	-0.075	-0.195	0.145	0.005	0.295	0.005	0.235	0.235	0.255	-0.225	-0.042	0.101	0.241	0.467	1.382
PF3D7_060770(	PF3D7_0607700::conserved Plasmodium protein, unknown function	-0.552	-0.372	-0.572	-0.052	0.018	-0.492	0.278	0.128	0.578	-0.162	0.468	0.318	0.418	-0.387	-0.066	0.181	0.401	0.788	1.727
PF3D7_132540(	PF3D7_1325400::CRWN-like protein, putative	-0.451	-0.081	-0.341	-0.061	-0.061	-0.261	0.189	0.109	0.349	-0.051	0.269	0.119	0.269	-0.233	-0.044	0.136	0.219	0.453	1.368
PF3D7_061220(	PF3D7_0612200::leucine-rich repeat protein	-0.123	-0.413	-0.223	-0.123	-0.283	-0.153	0.137	0.007	0.257	0.207	0.377	0.027	0.307	-0.221	-0.100	0.157	0.237	0.458	1.373
PF3D7_060380(	PF3D7_0603800::centrosomal protein CEP76, putative	-0.168	-0.418	-0.188	-0.078	-0.118	-0.438	0.022	0.062	0.332	-0.148	0.422	0.382	0.342	-0.213	-0.178	0.082	0.382	0.595	1.510
PF3D7_114230(	PF3D7_1142300::conserved Plasmodium membrane protein, unknown fu	-0.100	-0.390	-0.100	-0.080	-0.100	-0.200	0.130	0.050	0.090	-0.170	0.250	0.340	0.280	-0.168	-0.057	-0.010	0.290	0.458	1.373
PF3D7_135300(	PF3D7_1353000::tryptophan-rich protein, pseudogene	-0.029	-0.349	-0.049	-0.239	-0.059	-0.209	0.041	-0.029	0.031	0.031	0.431	0.201	0.231	-0.167	-0.076	0.011	0.287	0.454	1.370
PF3D7_112020(	PF3D7_1120200::conserved Plasmodium protein, unknown function	-0.406	-0.516	-0.806	-0.336	-0.126	0.364	0.144	-0.106	0.244	0.204	0.054	0.774	0.514	-0.516	0.127	0.114	0.447	0.963	1.950
PF3D7_143930(	PF3D7_1439300::Sad1/UNC domain-containing protein, putative	-0.330	-0.160	-0.240	-0.080	-0.200	0.060	0.220	0.150	-0.020	-0.020	0.130	0.320	0.170	-0.203	0.027	0.037	0.207	0.409	1.328
PF3D7_061040(	PF3D7_0610400::histone H3	0.066	0.296	-1.324	0.096	-0.274	0.446	-0.274	-0.504	0.416	-1.694	0.296	1.556	0.896	-0.216	-0.034	-0.594	0.916	1.133	2.192
PF3D7_110500(	PF3D7_1105000::histone H4	0.167	0.457	-0.943	0.207	-0.813	-0.413	0.017	-0.173	0.387	-1.393	0.547	1.157	0.797	-0.028	-0.403	-0.393	0.834	0.862	1.817
PF3D7_031730(	PF3D7_0317300::conserved Plasmodium protein, unknown function	-0.200	-0.130	-0.090	-0.040	0.060	-0.130	0.010	0.080	-0.020	-0.180	0.210	0.200	0.230	-0.115	-0.020	-0.040	0.213	0.328	1.256
PF3D7_072380(	PF3D7_0723800::conserved Plasmodium protein, unknown function	-0.124	-0.254	-0.214	-0.114	0.126	-0.054	0.076	0.016	0.116	-0.364	0.246	0.266	0.276	-0.176	0.049	-0.077	0.263	0.439	1.356
PF3D7_080210(	PF3D7_0802100::AP2 domain transcription factor, putative	-0.062	-0.472	-0.422	-0.152	0.098	-0.092	0.318	-0.012	0.148	-0.622	0.378	0.498	0.398	-0.277	0.108	-0.162	0.424	0.702	1.626
PF3D7_060980(	PF3D7_0609800::palmitoyltransferase DHHC2, putative	-0.016	-0.536	-0.366	-0.086	0.174	-0.236	0.034	-0.026	0.214	-0.156	0.254	0.484	0.264	-0.251	-0.009	0.011	0.334	0.585	1.500
PF3D7_041080(	PF3D7_0410800::conserved Plasmodium protein, unknown function	-0.180	-0.280	-0.380	-0.120	0.080	-0.040	0.090	-0.050	0.140	-0.420	0.180	0.650	0.330	-0.240	0.043	-0.110	0.387	0.627	1.544
PF3D7_070550(	PF3D7_0705500::inositol-phosphate phosphatase, putative	-0.202	-0.552	-0.442	-0.092	0.028	-0.122	0.088	0.078	0.218	-0.382	0.328	0.568	0.478	-0.322	-0.002	-0.028	0.458	0.780	1.717
PF3D7_111010(	PF3D7_1110100::conserved Plasmodium protein, unknown function	-0.203	-0.483	-0.423	-0.013	0.077	-0.223	0.077	-0.013	0.117	-0.273	0.267	0.747	0.347	-0.281	-0.023	-0.056	0.454	0.734	1.663
PF3D7_132830(	PF3D7_1328300::conserved protein, unknown function	-0.375	-0.635	-0.445	-0.125	0.055	-0.135	0.055	-0.025	0.195	-0.365	0.355	0.965	0.485	-0.395	-0.009	-0.065	0.601	0.997	1.995
PF3D7_133940(	PF3D7_1339400::cytochrome c oxidase subunit ApiCOX14, putative	-0.102	-0.422	-0.582	-0.172	-0.072	-0.052	0.108	0.068	0.048	-0.072	0.418	0.518	0.318	-0.320	-0.006	0.014	0.418	0.738	1.667
PF3D7_102820(	PF3D7_1028200::RING zinc finger protein, putative	-0.125	-0.235	-0.295	-0.025	-0.115	0.105	0.135	-0.065	0.145	-0.305	0.215	0.345	0.215	-0.170	0.042	-0.075	0.259	0.428	1.346
PF3D7_124140(	PF3D7_1241400::RNA-binding protein, putative	-0.115	-0.375	-0.215	-0.145	-0.185	0.155	0.065	-0.305	0.265	-0.495	0.195	0.785	0.365	-0.212	0.012	-0.178	0.449	0.661	1.581
PF3D7_123680(	PF3D7_1236800::protein-S-isoprenylcysteine O-methyltransferase	-0.242	-0.102	-0.402	0.008	-0.012	-0.182	-0.252	-0.112	0.188	-0.292	0.438	0.738	0.228	-0.185	-0.149	-0.072	0.468	0.653	1.572
PF3D7_132000(	PF3D7_1320000::golgi protein 1	-0.252	-0.142	-0.292	-0.052	0.128	0.028	-0.112	0.048	0.098	-0.212	0.178	0.368	0.208	-0.184	0.015	-0.022	0.252	0.436	1.353
PF3D7_101350(	PF3D7_1013500::phosphoinositide-specific phospholipase C	-0.156	-0.056	-0.086	-0.016	0.144	0.014	-0.026	-0.126	-0.146	-0.316	0.144	0.374	0.254	-0.079	0.044	-0.196	0.257	0.336	1.262
PF3D7_090800(	PF3D7_0908000::plasma membrane protein 1, putative	0.026	-0.204	-0.494	-0.044	0.116	0.016	-0.024	0.026	0.086	-1.004	0.226	0.826	0.446	-0.179	0.036	-0.297	0.499	0.678	1.600
PF3D7_081160(	PF3D7_0811600::conserved protein, unknown function	-0.337	-0.487	-0.547	-0.047	0.183	-0.027	-0.047	-0.087	0.093	-1.127	0.583	1.203	0.643	-0.354	0.036	-0.374	0.810	1.164	2.241
PF3D7_111650(	PF3D7_1116500::folate transporter 2	-0.123	-0.303	-0.293	-0.063	0.127	0.117	0.007	0.047	-0.103	-0.543	0.197	0.647	0.287	-0.196	0.084	-0.200	0.377	0.573	1.487
PF3D7_112320(	PF3D7_1123200::leucine-rich repeat protein	-0.212	-0.082	-0.362	-0.022	0.008	0.018	0.188	-0.082	-0.172	-0.522	0.298	0.598	0.338	-0.169	0.072	-0.258	0.412	0.581	1.496
PF3D7_113650(	PF3D7_1136500::casein kinase 1	-0.050	0.000	-0.290	-0.210	0.180	-0.070	-0.090	-0.150	-0.010	-0.640	0.250	0.860	0.220	-0.138	0.007	-0.267	0.443	0.581	1.496
PF3D7_134210(	PF3D7_1342100::aconitate hydratase	0.022	-0.258	-0.258	-0.128	0.022	-0.338	0.012	-0.168	0.052	-0.988	0.542	0.882	0.612	-0.156	-0.102	-0.368	0.678	0.834	1.783
PF3D7_031130(	PF3D7_0311300::phosphatidylinositol 3- and 4-kinase, putative	-0.063	-0.063	-0.103	-0.023	-0.053	0.037	0.027	0.007	0.127	-0.313	0.107	0.077	0.237	-0.063	0.004	-0.060	0.140	0.203	1.151
PF3D7_020760(	PF3D7_0207600::serine repeat antigen 5	-0.212	-0.472	-0.542	-0.072	0.418	-0.292	0.168	0.128	0.808	-1.422	0.548	0.438	0.508	-0.325	0.098	-0.162	0.498	0.823	1.768
PF3D7_020800(	PF3D7_0208000::serine repeat antigen 1	-0.101	-0.141	-0.331	-0.061	0.079	-0.341	0.209	0.089	0.469	-0.641	0.409	0.229	0.129	-0.158	-0.017	-0.027	0.256	0.414	1.333
PF3D7_093580(	PF3D7_0935800::cytoadherence linked asexual protein 9	-0.025	-0.055	-0.625	-0.065	0.185	-0.325	0.155	0.005	0.425	-0.965	0.305	0.485	0.505	-0.193	0.005	-0.179	0.431	0.624	1.541
PF3D7_122760(	PF3D7_1227600::kinetochore protein SPC24, putative	-0.093	-0.533	-0.193	-0.053	0.267	0.197	0.427	0.037	0.157	-0.923	0.177	0.177	0.357	-0.218	0.297	-0.243	0.237	0.455	1.371
PF3D7_134640(	PF3D7_1346400::VPS13 domain-containing protein, putative	0.005	-0.375	-0.195	-0.145	0.115	0.145	0.215	0.085	0.065	-0.635	0.215	0.185	0.315	-0.177	0.159	-0.161	0.239	0.416	1.334
PF3D7_146810(	PF3D7_1468100::MORC family protein	-0.216	-0.306	-0.276	-0.106	0.304	0.124	0.304	0.104	0.094	-0.696	0.314	0.134	0.224	-0.226	0.244	-0.166	0.224	0.450	1.366
PF3D7_141440(	PF3D7_1414400::serine/threonine protein phosphatase PP1	-0.084	0.116	-0.494	0.056	0.256	0.216	0.186	-0.084	0.026	-0.894	0.136	0.326	0.236	-0.101	0.219	-0.317	0.233	0.334	1.261
PF3D7_145940(	PF3D7_1459400::conserved protein, unknown function	0.034	-0.066	-0.226	0.064	0.304	0.114	0.104	0.164	0.164	-1.076	0.174	0.114	0.134	-0.049	0.174	-0.249	0.141	0.189	1.140
PF3D7_082100(	PF3D7_0821000::conserved Plasmodium protein, unknown function	-0.200	-0.130	-0.450	-0.010	0.090	0.300	0.180	0.010	0.030	-0.460	0.110	0.340	0.190	-0.198	0.190	-0.140	0.213	0.411	1.329
PF3D7_120540(	PF3D7_1205400::kelch domain-containing protein, putative	-0.050	-0.150	-0.380	-0.020	0.030	0.220	0.150	0.110	0.090	-0.480	0.040	0.160	0.280	-0.150	0.133	-0.093	0.160	0.310	1.240
PF3D7_API042(	PF3D7_API04200::DNA-directed RNA polymerase subunit beta'', putative	-0.190		-1.310	-0.110	0.610	0.460	0.510	0.040	0.050	-0.940	0.230	0.220	0.430	-0.537	0.527	-0.283	0.293	0.830	1.778
PF3D7_API044(	PF3D7_API04400::DNA-directed RNA polymerase subunit beta, putative	-0.157	-0.177	-0.657	-0.197	0.063	0.183	0.123	0.113	0.413	-0.547	0.013	0.243	0.583	-0.297	0.123	-0.007	0.280	0.577	1.491
PF3D7_136400(	PF3D7_1364000::conserved protein, unknown function	-0.204	-0.044	-0.654	0.016	0.076	0.026	0.226	0.106	-0.014	-0.254	0.206	0.186	0.326	-0.221	0.109	-0.054	0.239	0.461	1.376
PF3D7_072790(	PF3D7_0727900::conserved Plasmodium protein, unknown function	-0.033	-0.293	-0.103	-0.023	0.167	0.157	0.027	0.137	-0.103	-0.333	0.027	0.267	0.107	-0.113	0.117	-0.100	0.134	0.247	1.186
PF3D7_120260(	PF3D7_1202600::nuclear export mediator factor NEMF, putative	-0.030	-0.460	-0.060	-0.150	0.090	-0.080	-0.060	-0.030	0.000	-0.320	0.350	0.440	0.310	-0.175	-0.017	-0.117	0.367	0.542	1.456
PF3D7_123740(	PF3D7_1237400::hydrolase, putative	0.037	-0.723	0.037	0.107	0.177	0.057	-0.143	-0.063	-0.123	-0.383	0.167	0.387	0.467	-0.136	0.030	-0.190	0.340	0.476	1.391
PF3D7_113850(	PF3D7_1138500::protein phosphatase PPM2	-0.188	-0.858	0.012	0.042	0.182	-0.058	0.092	0.122	0.112	-0.348	0.382	0.162	0.342	-0.248	0.072	-0.038	0.296	0.543	1.457
PF3D7_143690(	PF3D7_1436900::histidine triad protein, putative	0.022	-1.058	-0.038	0.142	-0.058	-0.218	0.112	0.062	0.262	-0.148	0.302	0.362	0.262	-0.233	-0.055	0.058	0.308	0.542	1.456
PF3D7_146620(	PF3D7_1466200::early gametocyte enriched phosphoprotein EGXP	0.052	-0.708	-0.078	-0.088	-0.258	0.262	0.342	-0.058	0.152	-0.528	0.242	0.472	0.202	-0.206	0.115	-0.145	0.305	0.511	1.425
PF3D7_021600(	PF3D7_0216000::DEAD/DEAH box helicase, putative	-0.147	-0.117	-0.237	-0.157	-0.137	0.063	0.003	-0.077	0.443	0.003	0.103	0.323	-0.067	-0.164	-0.024	0.123	0.120	0.284	1.218
PF3D7_050450(	PF3D7_0504500::MOLO1 domain-containing protein, putative	-1.130	0.010	-0.520	0.040	-0.450	-0.100	-0.550	0.260	0.950	0.180	0.430	0.840	0.040	-0.400	-0.367	0.463	0.437	0.837	1.786
PF3D7_070990(	PF3D7_0709900::hydrolase, putative	-0.178	-0.058	-0.008	-0.358	0.062	-0.418	-0.158	0.022	0.162	0.372	0.182	0.272	0.112	-0.151	-0.172	0.185	0.188	0.339	1.265
PF3D7_031140(	PF3D7_0311400::kinase-related protein PKRP, putative	-0.125	-0.855	-0.275	-0.525	-0.195	-0.785	-0.725	-0.225	0.235	1.355	0.305	0.895	0.915	-0.445	-0.568	0.455	0.705	1.150	2.219
PF3D7_072310(	PF3D7_0723100::protein CPH1, putative	-0.616	-0.816	-0.456	-0.326	-0.156	-1.106	-0.576	-0.236	0.254	1.904	0.224	1.034	0.874	-0.554	-0.613	0.641	0.711	1.264	2.402
PF3D7_111880(	PF3D7_1118800::actin-related protein 2/3 complex subunit 1, putative	-0.216	-0.156	-0.226	-0.196	-0.166	-0.246	-0.556	-0.356	0.094	0.804	-0.066	0.664	0.624	-0.199	-0.323	0.181	0.407	0.606	1.522
PF3D7_010400(	PF3D7_0104000::thrombospondin-related sporozoite protein	-0.314	0.076	-0.134	-0.074	-0.094	-1.444	-1.094	-0.204	0.226	1.456	0.306	0.866	0.426	-0.111	-0.877	0.493	0.533	0.644	1.563
PF3D7_101040(	PF3D7_1010400::MORN repeat protein, putative	-0.049	-0.059	-0.309	-0.459	-0.559	-0.999	-0.499	-0.349	0.501	1.191	0.351	0.931	0.311	-0.219	-0.686	0.447	0.531	0.750	1.682
PF3D7_082890(	PF3D7_0828900::conserved protein, unknown function	-0.187	-0.047	-0.467	-0.167	-0.147	-0.517	-0.367	-0.217	0.283	0.623	0.133	0.773	0.303	-0.217	-0.344	0.230	0.403	0.620	1.537
PF3D7_135980(	PF3D7_1359800::ADP-ribosylation factor, putative	-0.483	0.037	-0.593	-0.173	-0.353	-1.443	-0.913	-0.503	0.217	1.217	0.687	1.407	0.897	-0.303	-0.903	0.310	0.997	1.300	2.462
PF3D7_021320(	PF3D7_0213200::mitochondrial respiratory chain complex II subunit, puta	-0.782	-0.072	-0.172	-0.062	-0.362	-0.562	-0.422	-0.112	0.468	1.158	-0.002	0.668	0.248	-0.272	-0.448	0.505	0.305	0.577	1.491
PF3D7_042050(	PF3D7_0420500::conserved Plasmodium protein, unknown function	-1.109	-0.759	-0.069	-0.089	-0.369	-1.199	-0.749	-0.179	0.241	1.531	0.611	1.071	1.071	-0.507	-0.773	0.531	0.917	1.424	2.684
PF3D7_092750(	PF3D7_0927500::dynein light chain, putative	-0.976	-0.246	-0.516	-0.106	-0.346	-0.896	-0.926	-0.606	0.164	1.544	0.604	1.414	0.894	-0.461	-0.723	0.367	0.971	1.432	2.698
PF3D7_040600(	PF3D7_0406000::conserved protein, unknown function	-0.590	0.010	-0.230	-0.010	-0.350	-0.920	-0.620	-0.200	0.220	1.250	0.200	0.700	0.540	-0.205	-0.630	0.423	0.480	0.685	1.608
PF3D7_092740(	PF3D7_0927400::conserved Plasmodium protein, unknown function	-0.814	-0.294	-0.204	-0.184	-0.414	-0.944	-0.724	-0.244	0.306	1.536	0.116	1.046	0.816	-0.374	-0.694	0.533	0.659	1.033	2.047
PF3D7_135630(	PF3D7_1356300::ubiquitin-conjugating enzyme E2, putative	-0.582	0.048	-0.222	-0.032	-0.182	-0.782	-0.442	-0.222	0.278	0.808	0.208	0.698	0.428	-0.197	-0.469	0.288	0.444	0.642	1.560
PF3D7_061410(	PF3D7_0614100::filamin domain-containing protein, putative	-0.778	-0.138	-0.318	-0.138	-0.208	-0.768	-0.368	-0.048	0.342	0.892	0.472	0.592	0.462	-0.343	-0.448	0.396	0.509	0.852	1.805
PF3D7_123800(	PF3D7_1238000::COPI associated protein, putative	-0.597	-0.157	-0.447	-0.167	-0.337	-0.937	-0.257	-0.027	0.543	0.943	0.313	0.653	0.473	-0.342	-0.510	0.486	0.480	0.822	1.767
PF3D7_132750(	PF3D7_1327500::conserved protein, unknown function	-0.316	-0.206	-0.386	-0.206	-0.206	-0.616	0.004	-0.106	0.244	0.574	0.204	0.584	0.434	-0.279	-0.273	0.237	0.407	0.686	1.609
PF3D7_123670(	PF3D7_1236700::S-adenosyl-methyltransferase, putative	-0.438	-0.128	-0.318	-0.008	-0.088	-0.318	-0.268	-0.198	0.242	0.732	0.242	0.262	0.282	-0.223	-0.224	0.259	0.262	0.485	1.400
PF3D7_131670(	PF3D7_1316700::conserved protein, unknown function	-0.462	-0.022	-0.252	0.008	-0.042	-0.222	-0.102	-0.022	0.258	0.438	0.038	0.158	0.228	-0.182	-0.122	0.224	0.141	0.323	1.251
PF3D7_103800(	PF3D7_1038000::antigen UB05	-0.684	-0.254	-0.084	0.036	-0.004	-0.284	-0.514	-0.304	0.196	0.586	0.236	0.666	0.406	-0.246	-0.267	0.159	0.436	0.683	1.605
PF3D7_021980(	PF3D7_0219800::Plasmodium exported protein (PHISTc), unknown func	-0.868	-0.808	-0.238	-0.268	-0.188	-0.918	-0.228	-0.258	0.082	1.312	0.752	1.272	0.352	-0.545	-0.444	0.379	0.792	1.338	2.527
PF3D7_114590(	PF3D7_1145900::conserved Plasmodium protein, unknown function	-0.918	-0.128	-0.178	-0.328	-0.348	-0.678	-0.518	-0.388	0.292	1.282	0.522	1.252	0.142	-0.388	-0.515	0.395	0.638	1.027	2.037
PF3D7_071730(	PF3D7_0717300::transcription initiation factor IIE subunit alpha, putative	-0.595	-0.375	-0.095	-0.225	-0.345	-0.515	-0.535	-0.165	0.115	1.205	0.225	0.925	0.385	-0.323	-0.465	0.385	0.511	0.834	1.783
PF3D7_103780(	PF3D7_1037800::dynein intermediate chain, putative	-0.423	-0.343	-0.163	-0.373	-0.293	-0.393	-0.353	0.007	0.177	1.207	0.247	0.507	0.197	-0.326	-0.346	0.464	0.317	0.643	1.561
PF3D7_133840(	PF3D7_1338400::SprT-like domain-containing protein, putative	-0.329	-0.529	-0.159	-0.159	-0.399	-0.509	-0.339	-0.189	0.441	1.111	0.201	0.741	0.121	-0.294	-0.416	0.454	0.354	0.648	1.567
PF3D7_142290(	PF3D7_1422900::14-3-3 protein, putative	-0.154	-0.314	-0.214	-0.144	-0.444	-0.264	-0.434	-0.004	0.256	0.876	0.176	0.766	-0.104	-0.206	-0.381	0.376	0.279	0.486	1.400
PF3D7_071840(	PF3D7_0718400::ribosomal protein S8, mitochondrial, putative	-0.195	-0.385	-0.165	-0.155	-0.455	-0.255	-0.275	-0.145	0.155	1.665	-0.135	0.245	0.105	-0.225	-0.329	0.558	0.071	0.297	1.228
PF3D7_093130(	PF3D7_0931300::conserved Plasmodium protein, unknown function	-0.229	-0.399	-0.179	-0.469	-0.489	-0.469	0.101	0.071	0.491	1.481	0.161	-0.009	-0.059	-0.319	-0.286	0.681	0.031	0.350	1.275
PF3D7_131310(	PF3D7_1313100::conserved Plasmodium protein, unknown function	-0.295	-0.175	-0.235	-0.335	-0.025	-0.435	-0.045	0.005	0.175	1.325	0.015	-0.085	0.115	-0.260	-0.169	0.501	0.015	0.275	1.210
PF3D7_062640(	PF3D7_0626400::CRAL/TRIO domain-containing protein, putative	-0.505	-0.235	-0.215	-0.115	-0.295	-0.015	0.065	-0.045	0.085	0.575	0.105	0.265	0.335	-0.268	-0.082	0.205	0.235	0.503	1.417

PF3D7_092730(	PF3D7_0927300::fumarate hydratase	-0.393	-0.253	-0.313	-0.143	-0.243	0.007	0.097	-0.063	0.167	0.587	0.047	0.097	0.407	-0.276	-0.046	0.230	0.184	0.459	1.375
PF3D7_041600(	PF3D7_0416000::RNA-binding protein, putative	-0.356	-0.056	-0.106	-0.066	-0.126	-0.076	-0.006	-0.116	0.144	0.524	0.024	0.014	0.204	-0.146	-0.069	0.184	0.081	0.227	1.170
PF3D7_120230(	PF3D7_1202300::dynein heavy chain, putative	-0.402	-0.112	-0.212	-0.252	-0.302	-0.192	0.028	-0.092	0.248	0.898	0.098	0.078	0.218	-0.245	-0.156	0.351	0.131	0.376	1.298
PF3D7_092460(	PF3D7_0924600::conserved Plasmodium protein, unknown function	-0.872	-0.292	-0.242	-0.112	-0.242	-0.482	0.148	0.168	0.518	0.648	0.268	0.238	0.258	-0.380	-0.192	0.444	0.254	0.634	1.552
PF3D7_070510(	PF3D7_0705100::conserved Plasmodium protein, unknown function	-0.575	-0.155	-0.515	-0.005	-0.165	-0.535	0.105	0.035	0.435	0.485	0.295	0.225	0.375	-0.313	-0.199	0.318	0.298	0.611	1.527
PF3D7_122410(	PF3D7_1224100::conserved protein, unknown function	-0.455	-0.185	-0.395	-0.175	-0.405	-0.615	0.015	-0.005	0.695	0.715	0.235	0.195	0.385	-0.303	-0.335	0.468	0.271	0.574	1.489
PF3D7_021690(	PF3D7_0216900::conserved Plasmodium protein, unknown function	-0.653	-0.743	-0.073	-0.063	-0.183	-0.413	0.237	0.017	0.387	0.747	0.207	0.097	0.437	-0.383	-0.120	0.384	0.247	0.630	1.548
PF3D7_140460(	PF3D7_1404600::adenylyl cyclase alpha	-0.824	-0.694	-0.029	0.006	-0.544	-0.469	0.221	-0.084	0.401	1.131	0.476	0.246	0.166	-0.385	-0.264	0.482	0.296	0.681	1.604
PF3D7_142640(	PF3D7_1426400::MORN repeat protein, putative	-0.584	-0.814	-0.124	-0.084	-0.594	-0.554	-0.004	0.046	0.686	1.246	0.386	0.196	0.196	-0.401	-0.384	0.659	0.259	0.661	1.581
PF3D7_091980(	PF3D7_0919800::TLD domain-containing protein	-0.601	-0.121	-0.061	-0.081	-0.161	-0.291	-0.021	-0.001	0.249	0.599	0.199	0.009	0.279	-0.216	-0.157	0.283	0.163	0.378	1.300
PF3D7_111790(	PF3D7_1117900::conserved Plasmodium protein, unknown function	-0.489	-0.069	-0.009	-0.099	-0.229	-0.289	-0.019	0.061	0.231	0.601	0.111	0.051	0.151	-0.167	-0.179	0.297	0.104	0.271	1.207
PF3D7_147810(	PF3D7_1478100::Plasmodium exported protein (hyp13), unknown functi	-1.033	-0.263	-0.093	-0.053	-0.453	-0.793	-0.053	-0.133	0.437	1.507	0.367	0.297	0.267	-0.361	-0.433	0.604	0.310	0.671	1.592
PF3D7_121790(	PF3D7_1217900::PPPDE peptidase domain-containing protein, putative	-0.777	-0.177	-0.077	-0.007	-0.187	-0.757	-0.417	-0.027	0.223	1.133	0.333	0.193	0.543	-0.259	-0.454	0.443	0.356	0.616	1.532
PF3D7_113200(	PF3D7_1132000::ubiquitin-like protein, putative	-0.466	-0.306	0.054	0.044	-0.126	-0.316	-0.156	0.024	0.154	0.624	0.104	0.194	0.174	-0.169	-0.199	0.267	0.157	0.326	1.253
PF3D7_134320(	PF3D7_1343200::tRNA guanosine-2'-O-methyltransferase, putative	-0.737	-0.477	0.013	0.013	-0.127	-0.377	-0.127	0.073	0.153	0.893	0.133	0.103	0.463	-0.297	-0.210	0.373	0.233	0.530	1.444
PF3D7_032170(	PF3D7_0321700::conserved Plasmodium protein, unknown function	-0.495	-0.265	-0.125	-0.075	-0.195	-0.045	0.005	0.145	0.365	0.395	0.245	-0.005	0.055	-0.240	-0.079	0.301	0.098	0.338	1.264
PF3D7_131960(	PF3D7_1319600::ACDC domain-containing protein, putative	-0.442	-0.202	-0.222	-0.132	-0.402	-0.042	0.148	-0.062	0.548	0.368	0.218	0.238	-0.012	-0.250	-0.099	0.284	0.148	0.398	1.317
PF3D7_140380(	PF3D7_1403800::nuclear formin-like protein MISFIT, putative	-0.248	-0.088	-0.148	-0.078	-0.518	-0.298	-0.058	-0.078	0.382	0.532	0.212	0.372	0.012	-0.140	-0.291	0.279	0.199	0.339	1.265
PF3D7_050460(	PF3D7_0504600::2-oxoisovalerate dehydrogenase subunit beta, mitoch	-0.803	-0.753	-0.103	-0.033	-0.343	-0.123	0.067	0.127	0.477	0.387	0.467	0.157	0.477	-0.423	-0.133	0.330	0.367	0.790	1.729
PF3D7_071520(	PF3D7_0715200::conserved Plasmodium protein, unknown function	-0.540	-0.310	-0.230	-0.040	-0.140	-0.050	0.160	0.130	0.300	0.080	0.210	0.200	0.230	-0.280	-0.010	0.170	0.213	0.493	1.408
PF3D7_021680(	PF3D7_0216800::TMEM121 domain-containing protein, putative	-0.310	-0.570	-0.390	-0.150	-0.040	-0.100	0.150	0.020	0.280	0.220	0.150	0.400	0.340	-0.355	0.003	0.173	0.297	0.652	1.571
PF3D7_070450(	PF3D7_0704500::serine/threonine protein kinase, putative	-0.534	-0.494	-0.544	-0.234	-0.034	-0.194	0.286	0.066	0.476	0.196	0.336	0.326	0.346	-0.451	0.019	0.246	0.336	0.788	1.726
PF3D7_053030(	PF3D7_0530300::PhIL1-interacting candidate PIC6	-0.434	-0.654	-0.234	-0.054	-0.114	-0.214	0.106	0.076	0.256	0.306	0.326	0.346	0.286	-0.344	-0.074	0.213	0.319	0.663	1.584
PF3D7_080630(	PF3D7_0806300::ferlin-like protein, putative	-0.350	-0.680	-0.330	-0.080	-0.180	-0.190	0.210	0.130	0.210	0.270	0.300	0.260	0.430	-0.360	-0.053	0.203	0.330	0.690	1.613
PF3D7_130400(	PF3D7_1304000::condensin complex subunit 2, putative	-0.489	-0.469	-0.269	-0.069	-0.119	-0.199	0.071	0.031	0.161	0.361	0.281	0.311	0.401	-0.324	-0.083	0.184	0.331	0.655	1.575
PF3D7_092790(	PF3D7_0927900::phosphatidylserine decarboxylase	-0.662	-0.982	-0.452	-0.242	-0.292	-0.032	0.208	-0.012	0.258	0.718	0.228	0.488	0.778	-0.585	-0.039	0.321	0.498	1.083	2.118
PF3D7_010380(	PF3D7_0103800::actin-related protein ARP1, putative	-0.241	-0.401	-0.281	-0.151	-0.161	-0.361	-0.071	-0.031	0.459	0.519	0.099	0.439	0.179	-0.268	-0.197	0.316	0.239	0.508	1.422
PF3D7_052420(	PF3D7_0524200::conserved Plasmodium membrane protein, unknown fu	-0.272	-1.112	-0.262	-0.342	0.008	-0.432	-0.202	-0.112	0.268	0.818	0.488	0.958	0.198	-0.497	-0.209	0.324	0.548	1.045	2.063
PF3D7_144910(	PF3D7_1449100::CLASP domain-containing protein, putative	-0.163	-0.563	-0.423	-0.233	-0.183	-0.243	-0.063	-0.163	0.207	0.547	0.177	0.697	0.407	-0.346	-0.163	0.197	0.427	0.773	1.708
PF3D7_041800(	PF3D7_0418000::conserved Plasmodium protein, unknown function	-0.620	-0.550	-0.160	-0.070	-0.330	-0.450	0.090	-0.010	0.440	0.450	0.540	0.550	0.120	-0.350	-0.230	0.293	0.403	0.753	1.686
PF3D7_134800(	PF3D7_1348000::conserved Plasmodium protein, unknown function	-0.458	-0.468	-0.258	-0.068	-0.098	-0.448	-0.018	0.042	0.412	0.272	0.352	0.572	0.172	-0.313	-0.188	0.242	0.365	0.678	1.600
PF3D7_135990(	PF3D7_1359900::conserved Plasmodium membrane protein, unknown fu	-0.227	-0.237	-0.127	-0.057	-0.117	-0.077	0.103	0.033	0.073	0.153	0.233	0.203	0.043	-0.162	-0.030	0.086	0.160	0.322	1.250
PF3D7_073050(	PF3D7_0730500::conserved Plasmodium protein, unknown function	-0.856	-0.446	-0.076	0.044	-0.046	-0.316	-0.176	0.084	0.134	0.334	0.304	0.534	0.484	-0.334	-0.179	0.184	0.441	0.774	1.710
PF3D7_125110(	PF3D7_1251100::conserved Plasmodium protein, unknown function	-0.258	-0.168	-0.188	-0.048	-0.028	-0.208	0.032	0.012	0.092	0.312	0.152	0.212	0.092	-0.166	-0.068	0.138	0.152	0.318	1.246
PF3D7_062020(	PF3D7_0620200::conserved Plasmodium protein, unknown function	-1.092	-0.902	-0.592	-0.142	-0.112	-0.732	-0.282	-0.052	0.358	1.148	0.548	1.158	0.698	-0.682	-0.376	0.484	0.801	1.483	2.796
PF3D7_050720(	PF3D7_0507200::subtilisin-like protease 3	-0.772	-0.762	-0.372	0.018	-0.202	-0.822	-0.172	0.028	0.388	0.778	0.558	0.758	0.578	-0.472	-0.399	0.398	0.631	1.103	2.149
PF3D7_124390(	PF3D7_1243900::double C2-like domain-containing protein	-0.525	-0.595	-0.295	-0.175	-0.145	-0.675	-0.125	-0.025	0.325	0.655	0.375	0.655	0.555	-0.398	-0.315	0.318	0.528	0.926	1.900
PF3D7_144960(	PF3D7_1449600::conserved protein, unknown function	-0.657	-0.707	-0.547	-0.217	-0.297	-0.617	0.113	-0.247	0.373	0.813	0.393	0.963	0.633	-0.532	-0.267	0.313	0.663	1.195	2.289
PF3D7_146110(	PF3D7_1461100::conserved Plasmodium protein, unknown function	-0.557	-0.497	-0.157	-0.037	-0.157	-0.477	0.113	-0.037	0.183	0.563	0.243	0.363	0.453	-0.312	-0.174	0.236	0.353	0.665	1.586
PF3D7_133050(	PF3D7_1330500::CTLH domain-containing protein, putative	-0.808	-0.558	-0.038	-0.088	-0.258	-0.508	-0.128	0.022	0.352	0.662	0.392	0.622	0.332	-0.373	-0.298	0.346	0.449	0.822	1.767
PF3D7_144240(	PF3D7_1442400::protein KIC9	-0.545	-0.445	-0.165	-0.035	-0.235	-0.235	-0.055	0.035	0.145	0.445	0.235	0.495	0.355	-0.297	-0.175	0.209	0.362	0.659	1.579
PF3D7_147140(	PF3D7_1471400::diacylglycerol kinase, putative	-0.442	-0.412	-0.122	-0.042	-0.002	-0.272	0.018	0.008	0.128	0.368	0.058	0.438	0.278	-0.255	-0.086	0.168	0.258	0.513	1.427
PF3D7_053110(	PF3D7_0531100::conserved Plasmodium protein, unknown function	-0.545	0.055	-0.245	-0.125	-0.225	-0.555	-0.115	0.025	0.415	0.275	0.335	0.345	0.365	-0.215	-0.299	0.238	0.348	0.563	1.478
PF3D7_032110(	PF3D7_0321100::conserved Plasmodium protein, unknown function	-0.764	-0.254	-0.274	-0.134	0.016	-0.794	-0.274	0.086	0.336	0.556	0.446	0.476	0.576	-0.356	-0.351	0.326	0.499	0.856	1.810
PF3D7_053090(	PF3D7_0530900::formin 1	-0.728	-0.558	-0.248	0.012	-0.028	-0.798	-0.058	0.052	0.382	0.432	0.352	0.522	0.672	-0.381	-0.295	0.288	0.515	0.896	1.861
PF3D7_101960(	PF3D7_1019600::conserved Plasmodium protein, unknown function	-0.527	-0.267	-0.167	-0.087	-0.107	-0.477	-0.177	-0.007	0.183	0.243	0.463	0.483	0.443	-0.262	-0.254	0.140	0.463	0.725	1.653
PF3D7_111480(	PF3D7_1114800::glycerol-3-phosphate dehydrogenase, putative	-0.659	-0.259	-0.199	-0.189	-0.199	-0.789	-0.649	-0.089	0.501	0.391	0.541	0.851	0.751	-0.327	-0.546	0.267	0.714	1.041	2.057
PF3D7_050890(	PF3D7_0508900::protein AAP6	-0.555	-0.285	-0.385	-0.225	0.005	-0.515	-0.085	0.035	0.465	0.165	0.455	0.505	0.415	-0.362	-0.198	0.222	0.459	0.821	1.766
PF3D7_070410(	PF3D7_0704100::basal complex transmembrane protein 2	-0.542	-0.192	-0.462	-0.182	-0.072	-0.542	-0.032	0.068	0.408	0.188	0.398	0.488	0.478	-0.345	-0.216	0.221	0.454	0.799	1.740
PF3D7_111490(	PF3D7_1114900::filamin domain-containing protein, putative	-0.370	-0.200	-0.320	-0.050	-0.060	-0.490	0.030	0.000	0.240	0.040	0.380	0.410	0.390	-0.235	-0.173	0.093	0.393	0.628	1.546
PF3D7_113800(	PF3D7_1138000::conserved Plasmodium protein, unknown function	-0.515	-0.175	-0.575	-0.085	-0.005	-0.595	0.055	0.015	0.385	0.095	0.275	0.455	0.665	-0.337	-0.181	0.165	0.465	0.803	1.744
PF3D7_060410(	PF3D7_0604100::AP2 domain transcription factor	-0.468	-0.628	-0.578	-0.148	0.042	-0.748	0.082	0.052	0.522	0.102	0.572	0.572	0.622	-0.455	-0.208	0.226	0.589	1.044	2.062
PF3D7_144130(	PF3D7_1441300::serine/threonine protein kinase, putative	-0.268	-0.398	-0.318	-0.238	-0.098	-0.398	0.062	-0.018	0.372	0.232	0.312	0.382	0.372	-0.305	-0.144	0.196	0.356	0.661	1.581
PF3D7_090820(	PF3D7_0908200::conserved Plasmodium protein, unknown function	-0.422	-0.032	-0.242	-0.182	-0.122	-0.192	0.118	0.098	0.158	0.108	0.198	0.278	0.238	-0.220	-0.066	0.121	0.238	0.458	1.373
PF3D7_112260(	PF3D7_1122600::conserved Plasmodium protein, unknown function	-0.513	-0.133	-0.303	-0.243	-0.233	-0.293	-0.083	0.027	0.437	0.287	0.277	0.507	0.267	-0.298	-0.203	0.250	0.350	0.648	1.567
PF3D7_132260(	PF3D7_1322600::conserved Plasmodium protein, unknown function	-0.428	-0.048	-0.438	-0.298	-0.388	-0.358	0.042	-0.068	0.292	0.302	0.312	0.722	0.352	-0.303	-0.234	0.176	0.462	0.765	1.699
PF3D7_080620(	PF3D7_0806200::Dpy-19-like C-mannosyltransferase	-0.614	-0.264	-0.224	-0.084	-0.144	-0.464	-0.154	-0.014	0.346	0.016	0.586	0.716	0.296	-0.296	-0.254	0.116	0.533	0.829	1.777
PF3D7_135610(	PF3D7_1356100::conserved Plasmodium protein, unknown function	-0.506	-0.116	-0.136	-0.056	-0.086	-0.166	-0.116	-0.026	0.174	0.064	0.274	0.404	0.294	-0.204	-0.123	0.071	0.324	0.528	1.441
PF3D7_124890(	PF3D7_1248900::26S protease regulatory subunit 8, putative	-0.322	-0.022	-0.052	-0.022	-0.022	-0.152	-0.012	0.008	0.018	-0.022	0.178	0.208	0.218	-0.105	-0.062	0.001	0.201	0.306	1.236
PF3D7_112310(	PF3D7_1123100::calcium-dependent protein kinase 7	-0.392	-0.412	-0.132	-0.192	0.198	-0.562	-0.352	0.028	0.098	-0.122	0.528	0.578	0.738	-0.282	-0.239	0.001	0.614	0.897	1.862
PF3D7_144300(	PF3D7_1443000::serine/threonine protein kinase	-0.396	-0.406	-0.246	-0.146	-0.016	-0.216	-0.286	-0.086	0.224	0.094	0.284	0.704	0.494	-0.299	-0.173	0.077	0.494	0.793	1.732
PF3D7_145050(	PF3D7_1450500::conserved Plasmodium protein, unknown function	-0.570	-0.510	-0.130	-0.170	0.060	-0.220	-0.100	-0.030	0.190	0.010	0.420	0.580	0.470	-0.345	-0.087	0.057	0.490	0.835	1.784
PF3D7_147270(	PF3D7_1472700::DNA-directed RNA polymerase, alpha subunit, putative	-0.268	-0.388	-0.038	-0.078	-0.178	-0.098	-0.108	-0.008	-0.048	0.352	0.032	0.372	0.462	-0.193	-0.128	0.098	0.288	0.482	1.396
PF3D7_093250(	PF3D7_0932500::palmitoyltransferase DHHC6, putative	-0.092	-0.072	-0.422	-0.082	-0.192	-0.092	-0.202	-0.262	0.168	0.698	0.038	0.278	0.228	-0.167	-0.162	0.202	0.182	0.348	1.273
PF3D7_124750(	PF3D7_1247500::serine/threonine protein kinase, putative	-0.096	0.014	-0.386	-0.136	-0.176	-0.126	0.104	-0.186	0.054	0.324	0.164	0.324	0.124	-0.151	-0.066	0.064	0.204	0.355	1.279
PF3D7_100720(	PF3D7_1007200::rho GTPase-activating protein, putative	-0.023	-0.343	-0.353	-0.043	-0.273	-0.253	0.107	-0.243	0.267	0.237	0.187	0.417	0.317	-0.191	-0.140	0.087	0.307	0.498	1.412
PF3D7_142350(	PF3D7_1423500::conserved Plasmodium protein, unknown function	-0.083	-0.103	-0.323	-0.073	-0.223	-0.303	0.047	-0.123	0.367	0.177	0.227	0.337	0.077	-0.146	-0.160	0.140	0.214	0.359	1.283
PF3D7_050340(	PF3D7_0503400::actin-depolymerizing factor 1	-0.868	-0.518	-0.198	-0.098	-0.358	-0.268	-0.328	-0.078	0.132	0.362	0.412	1.552	0.262	-0.421	-0.318	0.138	0.742	1.163	2.238
PF3D7_021070(	PF3D7_0210700::syntaxin, Qa-SNARE family	-0.599	-1.049	-0.319	-0.179	-0.189	-0.599	-0.489	-0.649	-0.159	0.841	0.571	2.461	0.361	-0.537	-0.426	0.011	1.131	1.668	3.177
PF3D7_134530(	PF3D7_1345300::conserved Plasmodium protein, unknown function	-0.578	-0.388	-0.298	-0.218	-0.038	-0.168	-0.088	-0.418	0.002	0.482	0.442	1.042	0.222	-0.370	-0.098	0.022	0.569	0.939	1.917
PF3D7_120390(	PF3D7_1203900::ubiquitin-conjugating enzyme E2	-0.331	-0.451	-0.571	-0.201	-0.011	0.209	-0.261	-0.321	-0.001	-0.071	0.059	1.499	0.449	-0.388	-0.021	-0.131	0.669	1.058	2.081
PF3D7_123260(	PF3D7_1232600::conserved Plasmodium protein, unknown function	-0.367	-0.437	-0.577	-0.397	-0.097	-0.057	-0.217	-0.397	0.063	0.263	0.103	1.773	0.343	-0.444	-0.124	-0.024	0.740	1.184	2.272
PF3D7_123860(	PF3D7_1238600::sphingomyelin phosphodiesterase	-0.245	-0.325	-0.275	-0.285	0.065	0.075	-0.295	-0.355	0.375	0.005	0.375	0.585	0.295	-0.282	-0.051	0.009	0.419	0.701	1.625
PF3D7_061460(	PF3D7_0614600::conserved Plasmodium protein, unknown function	0.045	0.025	-0.125	-0.335	0.175	-0.895	-0.425	-0.315	0.125	0.245	0.135	0.725	0.615	-0.097	-0.381	0.019	0.492	0.589	1.504
PF3D7_020670(	PF3D7_0206700::adenylosuccinate lyase	-0.242	0.008	-0.182	-0.312	-0.132	-0.612	-0.572	-0.382	-0.262	0.618	0.178	1.238	0.648	-0.182	-0.438	-0.008	0.688	0.870	1.828
PF3D7_072980(	PF3D7_0729800::dynein light chain, putative	-0.248	-0.188	-0.298	-0.298	-0.128	-0.828	-0.688	-0.528	-0.088	0.422	0.052	1.572	1.252	-0.258	-0.548	-0.065	0.958	1.217	2.324
PF3D7_050860(	PF3D7_0508600::conserved protein, unknown function	-0.152	-0.282	-0.202	-0.262	-0.092	-0.482	-0.272	-0.242	0.098	0.498	0.138	0.678	0.568	-0.224	-0.282	0.118	0.462	0.686	1.609
PF3D7_131060(	PF3D7_1310600::ras-related protein Rab-5B	-0.257	-0.277	-0.227	-0.127	0.003	-0.787	-0.417	-0.167	0.213	0.433	0.053	0.813	0.743	-0.222	-0.400	0.160	0.536	0.758	1.692
PF3D7_091720(	PF3D7_0917200::conserved protein, unknown function	-0.243	-0.373	-0.153	-0.153	-0.133	-1.333	-0.603	0.247	0.427	0.627	0.207	0.677	0.807	-0.231	-0.690	0.434	0.564	0.794	1.734
PF3D7_072970(	PF3D7_0729700::conserved protein, unknown function	-0.580	-0.580	0.000	-0.080	0.020	-1.190	-0.740	-0.160	-0.010	1.010	0.480	1.090	0.740	-0.310	-0.637	0.280	0.770	1.080	2.114
PF3D7_101230(	PF3D7_1012300::cytochrome b-c1 complex subunit 7, putative	-0.254	-0.114	0.006	-0.024	-0.054	-0.534	-0.294	-0.064	0.066	0.336	0.056	0.566	0.306	-0.096	-0.294	0.113	0.309	0.406	1.325
PF3D7_103030(	PF3D7_1030300::conserved Plasmodium protein, unknown function	0.055	-0.305	-0.185	-0.015	-0.015	-1.095	-0.875	-0.055	-0.005	0.655	0.245	0.985	0.615	-0.113	-0.662	0.198	0.615	0.728	1.656
PF3D7_062070(	PF3D7_0620700::DnaJ protein, putative	-0.510	-0.190	-0.460	-0.150	-0.020	-0.310	-0.120	-0.140	0.130	0.370	0.340	0.720	0.340	-0.328	-0.150	0.120	0.467	0.794	1.734
PF3D7_082860(	PF3D7_0828600::folate transporter 1	-0.328	-0.258	-0.568	-0.078	-0.298	-0.408	-0.268	-0.158	0.252	0.642	0.372	0.722	0.382	-0.308	-0.325	0.245	0.492	0.800	1.741
PF3D7_010540(	PF3D7_0105400::conserved Plasmodium protein, unknown function	-0.395	-0.115	-0.605	-0.335	0.125	-0.655	-0.525	-0.425	0.125	0.575	0.395	1.425	0.405	-0.362	-0.351	0.092	0.742	1.104	2.150
PF3D7_100170(	PF3D7_1001700::Plasmodium exported protein (PHISTc), unknown func	-0.242	-0.192	-0.462	-0.162	-0.152	-0.652	-0.432	-0.252	0.328	0.388	0.368	1.128	0.328	-0.264	-0.412	0.155	0.608	0.873	1.831
PF3D7_093480(	PF3D7_0934800::cAMP-dependent protein kinase catalytic subunit	-0.268	-0.108	-0.828	-0.258	-0.118	-0.778	-0.368	-0.388	0.042	0.472	0.672	1.282	0.642	-0.365	-0.421	0.042	0.866	1.231	2.347
PF3D7_100300(	PF3D7_1003000::guanylate cyclase organizer UGO, putative	-0.194	0.036	-0.554	-0.374	0.006	-0.924	-0.364	-0.294	0.106	0.466	0.326	1.036	0.726	-0.271	-0.427	0.093	0.696	0.968	1.955
PF3D7_122770(	PF3D7_1227700::protein KIC2	-0.032	-0.032	-0.422	-0.302	-0.172	-0.692	-0.282	-0.232	0.288	0.198	0.288	0.858	0.528	-0.197	-0.382	0.085	0.558	0.755	1.688
PF3D7_061270(	PF3D7_0612700::6-cysteine protein P12	-0.462	0.018	-0.102	-0.062	-0.122	-0.562	-0.372	-0.142	0.018	0.338	0.308	0.778	0.368	-0.152	-0.352	0.071	0.484	0.637	1.555
PF3D7_114060(	PF3D7_1140600::conserved Plasmodium protein, unknown function	-0.391	-0.071	-0.351	-0.011	-0.021	-0.651	-0.311	-0.211	0.079	0.279	0.259	0.809	0.589	-0.206	-0.327	0.049	0.553	0.758	1.692
PF3D7_103510(	PF3D7_1035100::probable protein, unknown function	-0.428	-0.488	-0.248	-0.058	-0.148	-0.918	-0.148	-0.148	0.272	0.262	0.642	0.862	0.542	-0.305	-0.404	0.129	0.682	0.988	1.983
PF3D7_122930(	PF3D7_1229300::PhIL1-interacting candidate PIC1	-0.396	-0.376	-0.486	-0.146	-0.176	-0.776	-0.226	-0.176	0.394	0.184	0.514	1.014	0.654	-0.351	-0.393	0.134	0.727	1.078	2.112
PF3D7_072330(	PF3D7_0723300::conserved protein, unknown function	-0.285	-0.495	-0.405	-0.045	-0.395	-1.145	-0.605	-0.175	0.125	0.685	0.585	1.445	0.705	-0.307	-0.715	0.212	0.912	1.219	2.328
PF3D7_143020(	PF3D7_1430200::plasmepsin IX	-0.565	-0.615	-0.255	-0.085	-0.055	-0.785	-0.565	-0.305	0.315	0.555	0.675	1.195	0.485	-0.380	-0.468	0.189	0.785	1.165	2.242
PF3D7_021470(	PF3D7_0214700::conserved Plasmodium protein, unknown function	0.219	0.059	-0.741	-0.971	0.169	-1.441	-1.511	-1.341	-0.701	0.649	1.329	3.279	0.999	-0.358	-0.927	-0.464	1.869	2.228	4.683
PF3D7_040400(	PF3D7_0404000::conserved Plasmodium protein, unknown function	-0.348	-0.038	-0.628	-0.398	0.112	-1.208	-1.088	-0.818	-0.408	0.742	0.802	2.502	0.782	-0.353	-0.728	-0.162	1.362	1.715	3.283
PF3D7_131870(	PF3D7_1318700::protein AAP4	0.173	-0.067	-0.907	-0.987	-0.267	-0.937	-0.937	-1.057	-0.497	0.873	0.943	2.533	1.133	-0.447	-0.714	-0.227	1.536	1.983	3.954
PF3D7_123140(	PF3D7_1231400::membrane protein ICM1	-0.070	-0.090	-0.350	-0.430	-0.190	-0.270	-0.480	-0.400	-0.320	0.470	0.210	1.570	0.350	-0.235	-0.313	-0.083	0.710	0.945	1.925

PF3D7_050410(	PF3D7_0504100::AN1-type zinc finger protein, putative	-0.187	-0.477	-0.307	-0.237	-0.067	-0.857	-0.707	-0.397	-0.207	0.473	0.313	2.053	0.603	-0.302	-0.544	-0.044	0.990	1.292	2.448
PF3D7_112790(	PF3D7_1127900::conserved Plasmodium protein, unknown function	-0.215	-0.225	-0.075	-0.215	-0.195	-0.965	-0.695	-0.515	-0.245	0.535	0.525	1.915	0.375	-0.183	-0.619	-0.075	0.938	1.121	2.175
PF3D7_136140(	PF3D7_1361400::actin-depolymerizing factor 2	-0.645	-0.685	-0.475	-0.165	-0.025	-0.615	-0.865	-0.455	0.005	0.325	0.845	1.965	0.785	-0.492	-0.501	-0.041	1.199	1.691	3.228
PF3D7_146060(	PF3D7_1460600::inner membrane complex sub-compartment protein 3	-0.802	-0.402	-0.702	-0.352	-0.042	-1.222	-1.152	-0.822	-0.172	0.578	1.258	2.898	0.938	-0.565	-0.806	-0.139	1.698	2.263	4.798
PF3D7_102500(	PF3D7_1025000::Eps15-like protein	-0.026	0.004	-0.256	-0.126	-0.096	-0.276	-0.206	-0.176	-0.296	-0.016	0.234	0.754	0.484	-0.101	-0.193	-0.163	0.491	0.592	1.507
PF3D7_022060(	PF3D7_0220600::Plasmodium exported protein (hyp9), unknown functio	-0.104	-0.454	-0.434	-0.234	-0.004	-0.094	-0.324	-0.334	-0.184	0.166	0.586	0.886	0.526	-0.306	-0.141	-0.117	0.666	0.973	1.962
PF3D7_143080(	PF3D7_1430800::PhIL1 interacting protein PIP3	-0.077	-0.447	-0.537	-0.017	-0.297	-0.347	-0.227	-0.547	-0.047	0.143	0.633	1.123	0.643	-0.269	-0.290	-0.150	0.800	1.069	2.098
PF3D7_062500(	PF3D7_0625000::sphingomyelin synthase 1, putative	-0.081	-0.311	-0.511	-0.261	-0.181	-0.671	-0.421	-0.311	-0.171	0.309	0.579	1.449	0.579	-0.291	-0.424	-0.057	0.869	1.160	2.235
PF3D7_071750(	PF3D7_0717500::calcium-dependent protein kinase 4	-0.005	-0.325	-0.585	-0.485	-0.115	-0.815	-0.375	-0.385	-0.065	0.205	0.655	1.565	0.725	-0.350	-0.435	-0.081	0.982	1.332	2.517
PF3D7_141740(	PF3D7_1417400::rap guanine nucleotide exchange factor, putative, pse	-0.023	-0.133	-0.463	-0.393	-0.033	-0.643	-0.313	-0.533	-0.153	0.087	0.557	1.367	0.677	-0.253	-0.330	-0.200	0.867	1.120	2.173
PF3D7_146790(	PF3D7_1467900::rab GTPase activator, putative	-0.279	-0.189	-0.379	-0.369	-0.019	-0.319	-0.229	-0.239	-0.089	0.041	0.421	1.181	0.471	-0.304	-0.189	-0.096	0.691	0.995	1.993
PF3D7_123250(	PF3D7_1232500::CG2-related protein, putative	-0.032	-0.342	-0.802	-0.532	-0.212	-0.552	-0.162	-0.382	0.108	0.068	0.718	1.578	0.538	-0.427	-0.308	-0.068	0.945	1.372	2.588
PF3D7_142310(	PF3D7_1423100::ankyrin-repeat protein, putative	-0.237	-0.817	-0.507	-0.267	-0.187	-0.887	-0.187	-0.287	-0.157	0.273	0.593	1.683	0.983	-0.457	-0.420	-0.057	1.086	1.543	2.915
PF3D7_050690(	PF3D7_0506900::rhomboid protease ROM4	-0.126	0.014	-0.696	-0.396	-0.036	-0.536	-0.106	-0.246	0.244	-0.306	0.454	1.044	0.694	-0.301	-0.226	-0.103	0.731	1.032	2.044
PF3D7_052260(	PF3D7_0522600::magnesium transporter NIPA, putative	-0.145	0.115	-0.525	-0.205	-0.045	-0.575	-0.235	-0.215	0.235	-0.235	0.415	0.845	0.565	-0.190	-0.285	-0.071	0.609	0.798	1.739
PF3D7_112890(	PF3D7_1128900::conserved protein, unknown function	-0.301	-0.161	-0.701	-0.081	-0.101	-0.731	-0.001	-0.141	0.359	-0.271	0.369	0.979	0.779	-0.311	-0.277	-0.017	0.709	1.020	2.028
PF3D7_040990(	PF3D7_0409900::actin-like protein, putative	-0.323	-0.023	-0.633	-0.133	-0.043	-0.403	-0.073	-0.113	0.087	0.037	0.257	0.867	0.497	-0.278	-0.173	0.004	0.540	0.818	1.763
PF3D7_135970(	PF3D7_1359700::conserved Plasmodium protein, unknown function	-0.366	-0.186	-0.466	-0.286	-0.006	-0.426	-0.046	-0.016	0.264	0.084	0.304	0.704	0.444	-0.326	-0.159	0.111	0.484	0.810	1.753
PF3D7_081580(	PF3D7_0815800::vacuolar protein sorting-associated protein 9, putative	-0.155	-0.125	-0.315	-0.125	-0.105	-0.225	0.085	-0.085	0.165	-0.195	0.265	0.555	0.265	-0.180	-0.082	-0.039	0.361	0.542	1.456
PF3D7_144410(	PF3D7_1444100::conserved Plasmodium protein, unknown function	-0.295	-0.215	-0.335	-0.195	-0.075	-0.255	0.095	-0.205	0.055	-0.075	0.455	0.765	0.285	-0.260	-0.079	-0.075	0.501	0.762	1.695
PF3D7_081960(	PF3D7_0819600::ubiquitin-like protein, putative	-0.218	0.052	-0.338	-0.138	-0.258	-0.388	-0.208	-0.278	-0.028	-0.108	0.442	1.032	0.432	-0.160	-0.284	-0.138	0.636	0.796	1.736
PF3D7_102030(	PF3D7_1020300::cytoplasmic dynein intermediate chain, putative	0.067	-0.063	-0.883	-0.293	-0.293	-0.303	-0.273	-0.553	0.187	-0.203	0.547	1.457	0.607	-0.293	-0.290	-0.190	0.870	1.163	2.240
PF3D7_133370(	PF3D7_1333700::histone H3-like centromeric protein CSE4	-0.128	-0.058	-0.668	-0.238	-0.608	-0.258	0.132	-0.288	0.062	-0.158	0.252	1.202	0.752	-0.273	-0.244	-0.128	0.736	1.008	2.012
PF3D7_063040(	PF3D7_0630400::conserved Plasmodium protein, unknown function	-0.173	-0.053	-0.363	-0.133	-0.063	-0.293	-0.183	-0.213	0.477	-0.083	0.167	0.627	0.287	-0.181	-0.180	0.060	0.360	0.541	1.455
PF3D7_124780(	PF3D7_1247800::dipeptidyl aminopeptidase 2	0.052	-0.068	-0.518	-0.238	-0.208	-0.518	-0.308	-0.428	0.542	-0.228	0.542	1.142	0.242	-0.193	-0.345	-0.038	0.642	0.835	1.784
PF3D7_060290(	PF3D7_0602900::conserved Plasmodium protein, unknown function	-0.175	-0.205	-0.495	-0.125	-0.135	-0.145	0.055	-0.205	0.115	0.195	0.115	0.655	0.355	-0.250	-0.075	0.035	0.375	0.625	1.542
PF3D7_120520(	PF3D7_1205200::HAD domain ookinete protein, putative	-0.148	-0.378	-0.758	-0.058	-0.178	-0.368	-0.018	-0.218	0.072	0.222	0.392	0.892	0.542	-0.335	-0.188	0.026	0.609	0.944	1.924
PF3D7_091440(	PF3D7_0914400::protein KIC3	-0.206	-0.386	-1.016	-0.326	-0.116	-0.416	-0.146	-0.326	0.544	0.274	0.164	1.174	0.784	-0.484	-0.226	0.164	0.707	1.191	2.283
PF3D7_135050(	PF3D7_1350500::conserved Plasmodium protein, unknown function	-0.137	-0.197	-0.437	-0.257	0.023	-0.107	0.053	-0.067	0.113	0.083	0.063	0.503	0.363	-0.257	-0.010	0.043	0.310	0.567	1.481
PF3D7_030830(	PF3D7_0308300::PhIL1-interacting candidate PIC4	0.697	0.677	-0.953	-0.963	0.327	-1.613	-1.303	-1.393	-0.893	-0.343	1.197	3.077	1.487	-0.136	-0.863	-0.876	1.920	2.056	4.158
PF3D7_112670(	PF3D7_1126700::conserved Plasmodium protein, unknown function	0.615	0.565	-0.795	-0.965	0.255	-1.435	-1.135	-1.205	-0.675	-0.305	1.185	2.615	1.275	-0.145	-0.771	-0.728	1.692	1.837	3.572
PF3D7_120630(	PF3D7_1206300::conserved Plasmodium protein, unknown function	0.290	0.270	-0.400	-0.400	0.120	-0.890	-0.520	-0.450	-0.260	-0.160	0.460	1.220	0.720	-0.060	-0.430	-0.290	0.800	0.860	1.815
PF3D7_040780(	PF3D7_0407800::protein CINCH	-0.018	-0.058	-0.618	-0.408	0.052	-0.748	-0.198	-0.338	0.002	-0.368	0.552	1.362	0.782	-0.275	-0.298	-0.234	0.899	1.174	2.257
PF3D7_091600(	PF3D7_0916000::major facilitator superfamily domain-containing protein,	0.011	0.091	-0.709	-0.399	-0.059	-0.659	-0.159	-0.439	0.041	-0.279	0.481	1.361	0.721	-0.252	-0.293	-0.226	0.854	1.106	2.152
PF3D7_080280(	PF3D7_0802800::serine/threonine protein phosphatase 2B catalytic sub	0.063	0.113	-0.627	-0.327	-0.217	-0.467	-0.227	-0.257	0.053	-0.247	0.443	1.103	0.593	-0.194	-0.304	-0.150	0.713	0.908	1.876
PF3D7_100340(	PF3D7_1003400::conserved Plasmodium protein, unknown function	0.133	0.013	-0.617	-0.387	-0.117	-0.887	-0.327	-0.447	-0.087	-0.037	0.653	1.343	0.763	-0.214	-0.444	-0.190	0.920	1.134	2.195
PF3D7_130800(	PF3D7_1308000::conserved Plasmodium membrane protein, unknown fu	0.057	0.097	-0.323	-0.393	-0.063	-0.513	-0.313	-0.373	-0.013	-0.153	0.377	1.137	0.477	-0.141	-0.296	-0.180	0.664	0.804	1.746
PF3D7_040470(	PF3D7_0404700::dipeptidyl aminopeptidase 3	-0.083	-0.053	-0.883	-0.553	-0.073	-1.223	-0.643	-0.433	0.227	-0.163	0.637	2.077	1.167	-0.393	-0.646	-0.123	1.294	1.687	3.219
PF3D7_131800(	PF3D7_1318000::conserved protein, unknown function	0.088	0.068	-0.552	-0.172	-0.082	-0.782	-0.472	-0.312	0.158	-0.112	0.268	1.318	0.588	-0.142	-0.446	-0.089	0.724	0.867	1.823
PF3D7_032140(	PF3D7_0321400::pseudo protein kinase 1, putative	0.090	0.000	-0.930	-0.290	0.340	-1.370	-0.610	-0.730	-0.130	-0.810	0.880	2.430	1.130	-0.283	-0.547	-0.557	1.480	1.763	3.393
PF3D7_062030(	PF3D7_0620300::conserved Plasmodium protein, unknown function	0.214	-0.066	-0.546	-0.216	0.244	-1.096	-0.796	-0.506	-0.476	-0.546	0.824	1.954	1.014	-0.154	-0.549	-0.509	1.264	1.418	2.671
PF3D7_134150(	PF3D7_1341500::inner membrane complex suture component, putative	0.264	0.004	-0.566	-0.166	0.254	-1.006	-0.626	-0.516	-0.156	-0.606	0.644	1.524	0.954	-0.116	-0.459	-0.426	1.041	1.157	2.229
PF3D7_030410(	PF3D7_0304100::inner membrane complex protein 1e, putative	0.279	0.029	-0.871	-0.361	0.119	-1.161	-0.991	-0.841	-0.201	-0.501	0.949	2.249	1.299	-0.231	-0.677	-0.514	1.499	1.730	3.317
PF3D7_135680(	PF3D7_1356800::serine/threonine protein kinase ARK3, putative	0.342	0.262	-0.708	-0.588	0.192	-1.068	-0.698	-0.788	-0.428	-0.538	0.862	1.952	1.202	-0.173	-0.524	-0.584	1.339	1.512	2.851
PF3D7_080260(	PF3D7_0802600::adenylyl cyclase beta	0.000	-0.190	-0.550	-0.430	0.200	-0.960	-0.590	-0.330	0.040	-0.280	0.730	1.530	0.830	-0.293	-0.450	-0.190	1.030	1.323	2.501
PF3D7_072220(	PF3D7_0722200::rhoptry-associated leucine zipper-like protein 1	0.145	-0.095	-0.655	-0.405	0.235	-1.295	-0.815	-0.485	0.035	-0.495	0.825	1.885	1.125	-0.253	-0.625	-0.315	1.278	1.531	2.890
PF3D7_101220(	PF3D7_1012200::rhoptry associated adhesin	0.046	-0.224	-0.724	-0.264	0.356	-1.394	-0.824	-0.634	0.016	-0.584	0.926	2.086	1.216	-0.291	-0.621	-0.401	1.409	1.701	3.251
PF3D7_140160(	PF3D7_1401600::Plasmodium exported protein (PHISTb), unknown func	0.270	0.110	-0.470	-0.660	0.240	-1.320	-0.700	-0.590	-0.160	-0.500	0.930	1.680	1.170	-0.188	-0.593	-0.417	1.260	1.448	2.727
PF3D7_050150(	PF3D7_0501500::rhoptry-associated protein 3	0.148	0.098	-0.632	-0.192	0.368	-0.902	-0.632	-0.362	0.078	-0.862	0.608	1.568	0.718	-0.145	-0.389	-0.382	0.964	1.109	2.157
PF3D7_081770(	PF3D7_0817700::rhoptry neck protein 5	0.202	0.232	-0.608	-0.488	0.172	-1.008	-0.468	-0.408	0.232	-0.718	0.512	1.382	0.972	-0.166	-0.435	-0.298	0.955	1.121	2.175
PF3D7_114520(	PF3D7_1145200::serine/threonine protein kinase, putative	0.200	-0.010	-0.630	-0.510	0.200	-1.050	-0.410	-0.410	0.210	-0.490	0.630	1.430	0.840	-0.238	-0.420	-0.230	0.967	1.204	2.304
PF3D7_132110(	PF3D7_1321100::protein kinase domain-containing protein, putative	0.038	-0.102	-0.492	-0.302	0.258	-0.952	-0.392	-0.242	0.078	-0.732	0.508	1.328	0.998	-0.214	-0.362	-0.298	0.945	1.159	2.233
PF3D7_042340(	PF3D7_0423400::apical asparagine-rich protein AARP	-0.362	0.138	-0.382	-0.322	0.198	-1.162	-0.732	-0.472	-0.202	0.108	0.988	1.628	0.568	-0.232	-0.565	-0.188	1.062	1.293	2.451
PF3D7_091060(	PF3D7_0910600::SNARE protein	-0.019	-0.039	0.121	-0.139	0.081	-0.829	-0.539	-0.389	-0.309	-0.359	0.421	1.361	0.641	-0.019	-0.429	-0.353	0.807	0.827	1.774
PF3D7_100360(	PF3D7_1003600::inner membrane complex protein 1c, putative	0.622	0.292	-1.468	-1.208	0.622	-2.098	-1.748	-1.658	-0.928	-1.438	2.412	5.042	1.562	-0.441	-1.075	-1.342	3.005	3.446	10.897
PF3D7_092070(	PF3D7_0920700::CRAL/TRIO domain-containing protein, putative	-0.088	-0.228	-0.568	-0.368	0.222	-0.778	-0.738	-0.628	-0.448	-0.228	0.862	2.142	0.852	-0.313	-0.432	-0.435	1.285	1.598	3.028
PF3D7_102170(	PF3D7_1021700::VPS13 domain-containing protein, putative	-0.131	-0.041	-0.461	-0.511	0.259	-0.891	-0.611	-0.481	-0.611	-0.411	0.879	2.189	0.819	-0.286	-0.414	-0.501	1.296	1.582	2.993
PF3D7_110490(	PF3D7_1104900::calcium/calmodulin-dependent protein kinase, putative	0.058	-0.122	-0.742	-0.612	0.398	-1.272	-0.992	-1.012	-0.432	-0.302	1.318	2.538	1.168	-0.354	-0.622	-0.582	1.675	2.029	4.082
PF3D7_103540(	PF3D7_1035400::merozoite surface protein 3	-0.075	-0.205	-0.445	-0.345	0.215	-1.065	-0.625	-0.475	-0.155	-0.385	0.915	2.075	0.565	-0.267	-0.491	-0.338	1.185	1.453	2.737
PF3D7_103740(	PF3D7_1037400::conserved Plasmodium protein, unknown function	-0.156	-0.086	-0.216	-0.026	0.014	-0.466	-0.556	-0.306	-0.216	-0.066	0.274	1.184	0.624	-0.121	-0.336	-0.196	0.694	0.815	1.759
PF3D7_131660(	PF3D7_1316600::choline-phosphate cytidylyltransferase	-0.117	-0.167	-0.037	-0.177	0.043	-0.217	-0.307	-0.237	-0.107	-0.137	0.313	0.813	0.333	-0.124	-0.160	-0.160	0.486	0.611	1.527
PF3D7_124630(	PF3D7_1246300::protein KIC4	-0.261	-0.311	-0.091	-0.161	0.209	-0.901	-0.621	-0.261	-0.091	-0.271	0.679	1.289	0.789	-0.206	-0.437	-0.207	0.919	1.125	2.181
PF3D7_140940(	PF3D7_1409400::conserved protein, unknown function	-0.198	-0.328	-0.298	-0.068	0.242	-0.608	-0.468	-0.218	-0.058	-0.468	0.542	1.162	0.762	-0.223	-0.278	-0.248	0.822	1.045	2.063
PF3D7_050090(	PF3D7_0500900::serine/threonine protein kinase, FIKK family	-0.199	-0.529	-0.519	-0.109	0.411	-0.949	-0.389	-0.389	-0.129	0.031	0.821	1.151	0.801	-0.339	-0.309	-0.163	0.924	1.263	2.400
PF3D7_052240(	PF3D7_0522400::conserved Plasmodium protein, unknown function	-0.477	-0.387	-0.507	-0.397	0.143	-0.997	-0.347	-0.217	0.303	0.013	0.773	1.243	0.853	-0.442	-0.400	0.033	0.956	1.398	2.636
PF3D7_134380(	PF3D7_1343800::conserved Plasmodium protein, unknown function	-0.585	-0.305	-0.595	-0.345	0.175	-0.915	-0.355	-0.135	0.075	-0.085	0.815	1.395	0.865	-0.458	-0.365	-0.049	1.025	1.483	2.794
PF3D7_030520(	PF3D7_0305200::GRIP domain-containing protein, putative	-0.140	0.080	-0.090	-0.210	0.030	-0.650	-0.310	-0.150	0.040	-0.070	0.210	0.690	0.570	-0.090	-0.310	-0.060	0.490	0.580	1.495
PF3D7_042330(	PF3D7_0423300::conserved Plasmodium protein, unknown function	-0.055	0.145	-0.565	-0.315	0.135	-1.505	-0.945	-0.585	0.065	-0.065	0.795	1.755	1.145	-0.198	-0.772	-0.195	1.231	1.429	2.693
PF3D7_081310(	PF3D7_0813100::conserved Plasmodium protein, unknown function	-0.162	-0.112	-0.212	-0.282	-0.002	-0.852	-0.382	-0.382	0.148	0.038	0.508	1.108	0.588	-0.192	-0.412	-0.066	0.734	0.927	1.901
PF3D7_041970(	PF3D7_0419700::apical merozoite protein	-0.292	-0.242	-0.682	-0.332	0.048	-1.122	-0.682	-0.402	0.258	0.118	0.598	1.668	1.068	-0.387	-0.586	-0.009	1.111	1.498	2.825
PF3D7_141730(	PF3D7_1417300::cysteine protease ATG4, putative	-0.170	-0.090	-0.220	-0.230	0.030	-0.610	-0.260	-0.090	0.190	0.040	0.260	0.720	0.430	-0.178	-0.280	0.047	0.470	0.648	1.566
PF3D7_143840(	PF3D7_1438400::metacaspase-2	-0.341	-0.041	-0.221	-0.181	0.039	-0.771	-0.411	-0.211	0.059	-0.001	0.349	1.049	0.679	-0.196	-0.381	-0.051	0.693	0.888	1.851
PF3D7_102540(	PF3D7_1025400::conserved Plasmodium membrane protein, unknown fu	-0.103	-0.343	-0.393	-0.243	0.087	-0.803	-0.573	-0.243	0.267	-0.253	0.297	1.347	0.957	-0.271	-0.430	-0.076	0.867	1.138	2.200
PF3D7_101490(	PF3D7_1014900::protein KIC8	0.059	0.159	-0.451	-0.231	0.069	-0.931	-0.381	-0.231	0.269	-0.131	0.249	0.729	0.819	-0.116	-0.414	-0.031	0.599	0.715	1.641
PF3D7_103560(	PF3D7_1035600::merozoite surface protein	0.146	-0.024	-0.244	-0.284	-0.074	-0.994	-0.474	-0.254	0.126	0.096	0.526	0.916	0.536	-0.101	-0.514	-0.011	0.659	0.761	1.694
PF3D7_143730(	PF3D7_1437300::conserved Plasmodium protein, unknown function	-0.023	0.207	-0.553	-0.103	0.117	-1.153	-0.423	-0.273	0.307	-0.353	0.547	1.087	0.617	-0.118	-0.486	-0.106	0.750	0.868	1.826
PF3D7_144920(	PF3D7_1449200::conserved Plasmodium protein, unknown function	0.058	0.288	-0.812	-0.092	-0.112	-1.812	-0.692	-0.652	0.288	-0.092	0.948	1.638	1.048	-0.140	-0.872	-0.152	1.211	1.351	2.551
PF3D7_147630(	PF3D7_1476300::Plasmodium exported protein (PHISTb), unknown func	0.257	0.227	-0.413	-0.433	0.017	-1.003	-0.403	-0.223	0.167	-0.263	0.527	1.027	0.517	-0.091	-0.463	-0.106	0.690	0.781	1.718
PF3D7_130360(	PF3D7_1303600::conserved Plasmodium protein, unknown function	0.075	0.045	-0.245	0.005	0.145	-0.695	-0.515	-0.055	-0.005	-0.455	0.195	0.865	0.635	-0.030	-0.355	-0.171	0.565	0.595	1.510
PF3D7_071420(	PF3D7_0714200::conserved Plasmodium protein, unknown function	-0.080	-0.240	-0.270	-0.070	0.060	-0.040	-0.070	-0.030	-0.060	-0.200	0.170	0.630	0.200	-0.165	-0.017	-0.097	0.333	0.498	1.413
PF3D7_080250(	PF3D7_0802500::inositol 5-phosphatase, putative	-0.045	-0.265	-0.165	-0.135	0.095	-0.055	-0.095	0.025	-0.115	-0.075	0.205	0.405	0.215	-0.152	-0.018	-0.055	0.275	0.428	1.345
PF3D7_050850(	PF3D7_0508500::ND6 protein, putative	0.086	-0.354	-0.414	-0.284	0.106	-0.554	-0.174	-0.124	0.026	-0.564	0.476	1.086	0.686	-0.241	-0.207	-0.221	0.749	0.991	1.987
PF3D7_092940(	PF3D7_0929400::high molecular weight rhoptry protein 2	-0.128	-0.178	-0.928	-0.248	0.242	-0.638	-0.318	-0.278	0.232	-0.708	0.592	1.382	0.972	-0.370	-0.238	-0.251	0.982	1.353	2.554
PF3D7_125210(	PF3D7_1252100::rhoptry neck protein 3	-0.093	-0.313	-0.803	-0.203	0.317	-0.953	-0.173	-0.193	0.267	-1.143	0.737	1.487	1.067	-0.353	-0.270	-0.356	1.097	1.450	2.732
PF3D7_101820(	PF3D7_1018200::serine/threonine protein phosphatase 8, putative	0.035	-0.385	-0.575	-0.225	0.175	-0.865	-0.165	-0.155	0.355	-0.535	0.525	0.975	0.835	-0.287	-0.285	-0.111	0.779	1.066	2.093
PF3D7_114040(	PF3D7_1140400::conserved Plasmodium protein, unknown function	0.083	-0.447	-1.307	-0.247	0.143	-0.647	-0.427	-0.647	0.283	-1.267	0.723	2.473	1.283	-0.479	-0.310	-0.544	1.493	1.973	3.924
PF3D7_030310(	PF3D7_0303100::CLP1 P-loop domain-containing protein, putative	-0.148	-0.338	-0.178	-0.178	0.122	-0.228	-0.138	-0.138	0.012	-0.318	0.372	0.742	0.422	-0.211	-0.082	-0.148	0.512	0.723	1.650
PF3D7_141450(	PF3D7_1414500::atypical protein kinase, ABC-1 family, putative	-0.081	-0.351	-0.211	-0.281	0.109	-0.301	-0.231	-0.141	-0.011	-0.271	0.309	0.919	0.539	-0.231	-0.141	-0.141	0.589	0.820	1.765
PF3D7_113510(	PF3D7_1135100::protein phosphatase PPM8, putative	-0.017	-0.517	-0.267	-0.117	0.163	-0.337	-0.157	-0.287	0.083	-0.717	0.523	1.053	0.593	-0.229	-0.110	-0.307	0.723	0.953	1.935
PF3D7_102180(	PF3D7_1021800::schizont egress antigen-1	0.010	-0.490	-0.430	-0.230	-0.040	-0.280	0.050	-0.180	0.020	-0.210	0.460	0.830	0.490	-0.285	-0.090	-0.123	0.593	0.878	1.838
PF3D7_143660(	PF3D7_1436600::cGMP-dependent protein kinase	0.074	-0.576	-0.456	-0.306	-0.066	-0.466	-0.136	-0.296	0.044	-0.466	0.584	1.384	0.684	-0.316	-0.223	-0.239	0.884	1.200	2.297
PF3D7_040770(	PF3D7_0407700::conserved Plasmodium protein, unknown function	0.032	-0.198	-0.538	-0.288	0.112	-0.578	-0.278	-0.298	0.142	-0.098	0.382	1.072	0.542	-0.248	-0.248	-0.085	0.665	0.913	1.883
PF3D7_042350(	PF3D7_0423500::glideosome associated protein with multiple membrane	-0.232	-0.612	-0.922	-0.362	0.058	-0.742	-0.372	-0.442	0.198	-0.312	0.768	1.838	1.128	-0.532	-0.352	-0.185	1.245	1.777	3.426
PF3D7_134560(	PF3D7_1345600::inner membrane complex protein	-0.042	-0.252	-0.842	-0.262	0.108	-0.442	-0.372	-0.502	0.018	-0.272	0.608	1.498	0.758	-0.350	-0.236	-0.252	0.954	1.304	2.469
PF3D7_050160(	PF3D7_0501600::rhoptry-associated protein 2	-0.375	-1.015	-1.035	-0.135	0.445	-1.345	-0.795	-0.595	0.015	-0.415	1.275	2.755	1.225	-0.640	-0.565	-0.332	1.751	2.392	5.248
PF3D7_122310(	PF3D7_1223100::cAMP-dependent protein kinase regulatory subunit	-0.125	-0.605	-0.495	-0.155	-0.025	-0.645	-0.275	-0.325	0.005	-0.145	0.705	1.405	0.675	-0.345	-0.315	-0.155	0.929	1.273	2.417
PF3D7_146140(	PF3D7_1461400::MORN repeat protein, putative	0.106	-1.114	-0.804	-0.534	0.266	-1.524	-0.744	-0.584	-0.074	-0.384	1.356	2.936	1.096	-0.586	-0.667	-0.347	1.796	2.383	5.214
PF3D7_134430(	PF3D7_1344300::zinc finger protein, putative	-0.208	-0.728	-0.698	-0.428	0.302	-0.858	-0.508	-0.558	-0.218	-0.548	1.102	2.282	1.072	-0.516	-0.355	-0.442	1.485	2.001	4.002
PF3D7_133510(	PF3D7_1335100::merozoite surface protein 7	-0.062	-0.432	-0.612	-0.202	-0.022	-0.912	-0.312	-0.322	0.068	-0.642	0.878	2.018	0.558	-0.327	-0.416	-0.299	1.151	1.478	2.786

PF3D7_142020(	PF3D7_1420200::myosin-specific chaperone UNC, putative	-0.073	-0.253	-0.363	-0.133	-0.043	-0.303	-0.163	-0.253	-0.093	-0.163	0.287	1.187	0.367	-0.206	-0.170	-0.170	0.614	0.819	1.764
PF3D7_147260(	PF3D7_1472600::protein disulfide-isomerase	-0.159	-0.149	-0.589	-0.289	-0.009	-0.679	-0.439	-0.389	-0.139	-0.399	0.581	1.921	0.741	-0.297	-0.376	-0.309	1.081	1.378	2.598
PF3D7_114660(	PF3D7_1146600::oocyst rupture protein 1, putative	-0.185	-0.175	-0.665	-0.465	0.175	-0.325	-0.065	-0.395	-0.265	-0.285	0.405	1.575	0.675	-0.373	-0.072	-0.315	0.885	1.258	2.391
PF3D7_131280(	PF3D7_1312800::protein AAP2	0.092	0.002	-0.438	-0.368	-0.028	-0.228	0.032	-0.238	-0.078	-0.268	0.342	0.772	0.412	-0.178	-0.075	-0.195	0.508	0.687	1.610
PF3D7_040390(	PF3D7_0403900::SET domain protein, putative	-0.088	-0.108	-0.208	-0.068	-0.188	-0.518	-0.208	-0.148	0.182	0.102	0.282	0.442	0.532	-0.118	-0.305	0.045	0.418	0.537	1.451
PF3D7_111290(	PF3D7_1112900::conserved protein, unknown function	-0.305	-0.185	-0.485	-0.125	-0.405	-0.915	-0.545	-0.265	0.565	0.505	0.185	0.895	1.075	-0.275	-0.621	0.269	0.719	0.993	1.991
PF3D7_122130(	PF3D7_1221300::conserved Plasmodium protein, unknown function	-0.076	-0.136	-0.136	0.004	0.024	-0.816	-0.136	-0.166	0.304	0.014	0.214	0.404	0.504	-0.086	-0.309	0.051	0.374	0.460	1.376
PF3D7_080320(	PF3D7_0803200::filament assembling protein, putative	-0.037	-0.127	-0.227	-0.217	-0.097	-0.507	-0.077	-0.057	0.313	-0.117	0.263	0.463	0.423	-0.152	-0.227	0.046	0.383	0.535	1.449
PF3D7_111300(	PF3D7_1113000::conserved Plasmodium protein, unknown function	-0.129	-0.039	-0.289	-0.289	0.031	-0.729	-0.089	-0.099	0.321	-0.209	0.401	0.661	0.461	-0.187	-0.263	0.004	0.507	0.694	1.618
PF3D7_122860(	PF3D7_1228600::merozoite surface protein 9	-0.051	-0.471	-0.191	-0.311	-0.061	-0.991	-0.221	-0.061	0.529	-0.491	0.699	1.029	0.589	-0.256	-0.424	-0.007	0.773	1.028	2.040
PF3D7_134930(	PF3D7_1349300::tyrosine kinase-like protein	-0.083	-0.093	-0.113	-0.203	-0.073	-0.233	-0.033	0.067	0.187	-0.043	0.197	0.247	0.177	-0.123	-0.113	0.070	0.207	0.330	1.257
PF3D7_131360(	PF3D7_1313600::Clu domain-containing protein, putative	-0.347	0.023	-0.377	-0.107	-0.027	-0.877	-0.237	-0.127	0.323	-0.247	0.433	0.853	0.713	-0.202	-0.380	-0.017	0.666	0.868	1.826
PF3D7_132070(	PF3D7_1320700::conserved protein, unknown function	-0.529	0.091	-0.349	-0.089	-0.039	-0.649	-0.249	-0.059	0.371	-0.019	0.401	0.611	0.511	-0.219	-0.313	0.097	0.507	0.727	1.655
PF3D7_080490(	PF3D7_0804900::GTPase-activating protein, putative	-0.034	-0.414	0.006	-0.054	-0.144	-0.184	-0.104	-0.204	-0.284	-0.074	0.146	1.046	0.296	-0.124	-0.144	-0.187	0.496	0.620	1.537
PF3D7_110820(	PF3D7_1108200::D-tyrosyl-tRNA(Tyr) deacylase	0.147	-0.483	0.127	-0.103	0.127	-0.383	-0.093	-0.163	-0.463	-0.533	0.387	1.037	0.397	-0.078	-0.116	-0.386	0.607	0.685	1.608
PF3D7_135100(	PF3D7_1351000::phosphatidylinositol transfer protein, putative	0.008	-0.272	-0.062	-0.342	0.128	-0.402	-0.102	0.028	-0.092	-0.402	0.288	0.798	0.428	-0.167	-0.126	-0.156	0.504	0.672	1.593
PF3D7_082300(	PF3D7_0823000::serine/threonine protein kinase VPS15, putative	-0.357	-0.117	-0.037	0.033	0.053	-0.507	-0.397	-0.047	-0.177	0.223	0.423	0.653	0.253	-0.119	-0.284	0.000	0.443	0.563	1.477
PF3D7_131050(	PF3D7_1310500::ER membrane protein complex subunit 7, putative	-0.544	0.046	0.026	0.146	-0.074	-0.464	-0.274	-0.014	0.086	0.066	0.356	0.536	0.106	-0.081	-0.271	0.046	0.333	0.414	1.333
PF3D7_083270(	PF3D7_0832700::Plasmodium exported protein (PHISTa-like), unknown f	-0.170			-0.050	-0.410	-0.030	0.070	-0.120	-0.720	0.850	0.290		0.290	-0.110	-0.123	0.003	0.290	0.400	1.320
PF3D7_140290(	PF3D7_1402900::conserved Plasmodium protein, unknown function	-0.358	-0.848	-0.688	-0.108	-0.088	0.072	0.642	0.142	-0.578	0.592	0.392	0.272	0.562	-0.501	0.208	0.052	0.408	0.909	1.878
PF3D7_121110(	PF3D7_1211100::HSP20-like chaperone, putative	-0.172	-1.012	-0.282	-0.392	-0.062	-0.012	-0.012	-0.112	0.058	1.058	0.308	0.608	0.028	-0.465	-0.029	0.334	0.314	0.779	1.716
PF3D7_120590(	PF3D7_1205900::conserved protein, unknown function	0.003	-0.657	-0.117	-0.077	0.113	0.193	-0.027	-0.117	-0.057	0.073	0.113	0.283	0.273	-0.212	0.093	-0.034	0.223	0.435	1.352
PF3D7_131160(	PF3D7_1311600::conserved Plasmodium protein, unknown function	-0.099	-0.779	-0.059	-0.079	-0.109	0.251	0.221	-0.009	-0.109	0.271	-0.049	0.421	0.131	-0.254	0.121	0.051	0.167	0.422	1.339
PF3D7_123030(	PF3D7_1230300::subpellicular microtubule protein 2, putative	-0.320	-1.000	-0.140	-0.170	-0.060	0.180	0.080	-0.150	-0.090	0.520	0.090	0.630	0.430	-0.408	0.067	0.093	0.383	0.791	1.730
PF3D7_145870(	PF3D7_1458700::exonuclease V, mitochondrial, putative	-0.225	-0.575	-0.095	-0.105	-0.055	0.185	0.235	-0.185	-0.075	0.365	0.075	0.245	0.205	-0.250	0.122	0.035	0.175	0.425	1.343
PF3D7_133040(	PF3D7_1330400::ER lumen protein retaining receptor 1, putative	-0.220	-0.170	-0.530	-0.140	0.180	0.380	0.000	-0.040	0.290	0.150	-0.060	0.120	0.040	-0.265	0.187	0.133	0.033	0.298	1.230
PF3D7_032020(	PF3D7_0320200::CPW-WPC family protein	0.048	0.158	0.128	0.298	-0.032	0.118	-0.102	0.038	0.208	0.108	-0.142	-0.382	-0.452	0.158	-0.005	0.118	-0.325	-0.483	0.715
PF3D7_032360(	PF3D7_0323600::BSD domain-containing protein, putative	-0.137	-0.017	0.003	0.203	-0.017	0.123	-0.117	0.153	0.323	0.263	-0.247	-0.267	-0.267	0.013	-0.004	0.246	-0.260	-0.273	0.827
PF3D7_112830(	PF3D7_1128300::ATP-dependent 6-phosphofructokinase	0.308	0.078	-0.002	0.138	-0.272	0.198	0.228	0.298	0.418	0.268	-0.092	-0.932	-0.642	0.131	0.052	0.328	-0.555	-0.686	0.622
PF3D7_011450(	PF3D7_0114500::Plasmodium exported protein (hyp10), unknown functi	0.704	0.944	0.774	0.294	-0.586	-0.626	-0.376	-0.356	-0.136	0.244	-0.236	-0.236	-0.406	0.679	-0.529	-0.083	-0.293	-0.972	0.510
PF3D7_042500(	PF3D7_0425000::Plasmodium exported protein, unknown function, pseu	1.559	1.849	1.389	0.899	0.159	-0.291	-0.991	-0.471	-0.241	-0.441	-0.771	-1.061	-1.591	1.424	-0.374	-0.384	-1.141	-2.565	0.169
PF3D7_060220(	PF3D7_0602200::MYND-type zinc finger protein, putative	0.523	0.463	0.743	0.153	0.043	-0.227	-0.327	-0.187	-0.037	-0.107	-0.157	-0.367	-0.517	0.471	-0.170	-0.110	-0.347	-0.818	0.567
PF3D7_113610(	PF3D7_1136100::conserved Plasmodium protein, unknown function	0.237	0.237	0.417	0.207	0.007	-0.003	0.017	-0.433	-0.123	0.107	-0.213	-0.083	-0.373	0.274	0.007	-0.150	-0.223	-0.498	0.708
PF3D7_070660(	PF3D7_0706600::conserved Plasmodium protein, unknown function	0.207	0.247	0.467	0.197	0.027	-0.133	-0.413	0.147	0.137	0.187	0.037	-0.533	-0.573	0.279	-0.173	0.157	-0.356	-0.636	0.644
PF3D7_102800(	PF3D7_1028000::methyltransferase, putative	0.363	0.233	0.563	0.123	0.043	-0.197	-0.327	0.173	-0.047	0.393	-0.207	-0.477	-0.637	0.321	-0.160	0.173	-0.440	-0.761	0.590
PF3D7_092520(	PF3D7_0925200::ribosomal RNA-processing protein 8, putative	0.005	0.155	0.515	0.345	0.045	-0.095	-0.125	0.015	-0.005	0.185	-0.275	-0.305	-0.465	0.255	-0.058	0.065	-0.348	-0.603	0.658
PF3D7_072620(	PF3D7_0726200::serine/threonine protein kinase, FIKK family	0.248	0.368	0.168	0.148	-0.282	-0.022	-0.032	-0.102	0.158	0.018	-0.132	-0.202	-0.342	0.233	-0.112	0.025	-0.225	-0.458	0.728
PF3D7_021620(	PF3D7_0216200::Alba domain-containing protein, putative	0.438	1.188	0.078	0.058	-0.242	0.158	-0.282	0.038	0.198	-0.062	-0.282	-0.542	-0.742	0.440	-0.122	0.058	-0.522	-0.963	0.513
PF3D7_146310(	PF3D7_1463100::conserved Plasmodium protein, unknown function, pse	0.386	1.046	0.266	0.086	-0.274	-0.114	-0.434	0.186	0.346	-0.044	-0.244	-0.584	-0.624	0.446	-0.274	0.163	-0.484	-0.930	0.525
PF3D7_147600(	PF3D7_1476000::conserved Plasmodium protein, unknown function	0.560	0.750	0.220	0.040	-0.450	-0.450	-0.400	0.220	0.800	0.120	-0.170	-0.580	-0.660	0.393	-0.433	0.380	-0.470	-0.863	0.550
PF3D7_011410(	PF3D7_0114100::Pfmc-2TM Maurer's cleft two transmembrane protein	-0.024	0.246	0.666	0.226	0.226	-0.194	0.026	0.396	-0.704	0.326	-0.404	-0.284	-0.504	0.279	0.019	0.006	-0.397	-0.676	0.626
PF3D7_060120(	PF3D7_0601200::Pfmc-2TM Maurer's cleft two transmembrane protein	0.024	0.474	0.794	0.334	0.444	-0.396	0.044	0.594	-0.746	0.504	-0.696	-0.496	-0.876	0.406	0.031	0.117	-0.689	-1.096	0.468
PF3D7_070160(	PF3D7_0701600::Pfmc-2TM Maurer's cleft two transmembrane protein	0.070	0.230	0.630	0.300	0.180	-0.430	-0.040	0.440	-0.710	0.300	-0.210	-0.350	-0.410	0.308	-0.097	0.010	-0.323	-0.631	0.646
PF3D7_071310(	PF3D7_0713100::Pfmc-2TM Maurer's cleft two transmembrane protein	0.112	0.482	0.512	0.122	0.072	-0.168	-0.088	0.212	-0.498	0.272	-0.328	-0.248	-0.458	0.307	-0.061	-0.004	-0.344	-0.652	0.637
PF3D7_103970(	PF3D7_1039700::Pfmc-2TM Maurer's cleft two transmembrane protein	-0.144	-0.044	0.906	0.166	0.296	-0.534	0.066	0.576	-0.874	0.386	-0.284	-0.254	-0.264	0.221	-0.057	0.029	-0.267	-0.488	0.713
PF3D7_090290(	PF3D7_0902900::conserved Plasmodium protein, unknown function	0.216		0.066	0.116	0.266	-0.174	-0.074	0.096	-0.254	0.086	-0.094	-0.024	-0.224	0.132	0.006	-0.024	-0.114	-0.247	0.843
PF3D7_022150(	PF3D7_0221500::Pfmc-2TM Maurer's cleft two transmembrane protein, p	-0.096	0.584	1.234	0.444	-1.046	-1.696	-1.266	1.994	0.524	2.274	-1.106	-0.706	-1.136	0.541	-1.336	1.597	-0.983	-1.524	0.348
PF3D7_020180(	PF3D7_0201800::knob associated heat shock protein 40	0.083	0.533	0.093	0.123	-0.137	-0.357	-0.157	-0.067	-0.467	0.533	-0.287	0.023	0.083	0.208	-0.217	0.000	-0.060	-0.268	0.830
PF3D7_114890(	PF3D7_1148900::Plasmodium exported protein, unknown function	0.680	0.430	-0.020	-0.010	-0.430	-0.230	-0.170	-0.120	-0.340	0.840	-0.160	-0.290	-0.180	0.270	-0.277	0.127	-0.210	-0.480	0.717
PF3D7_020160(	PF3D7_0201600::PHISTb domain-containing RESA-like protein 1	0.161	0.631	0.101	0.051	-0.029	-0.229	-0.059	-0.089	-0.369	0.191	-0.269	-0.069	-0.019	0.236	-0.106	-0.089	-0.119	-0.355	0.782
PF3D7_102610(	PF3D7_1026100::conserved Plasmodium protein, unknown function	0.055	1.525	0.075	-0.115	-0.395	-0.305	-0.185	-0.475	-0.135	0.645	-0.155	-0.305	-0.235	0.385	-0.295	0.012	-0.231	-0.617	0.652
PF3D7_081920(	PF3D7_0819200::perforin-like protein 5	0.480	0.220	0.040	0.040	-0.100	0.180	-0.040	-0.030	-0.440	0.350	-0.150	-0.170	-0.380	0.195	0.013	-0.040	-0.233	-0.428	0.743
PF3D7_100500(	PF3D7_1005000::methionine--tRNA ligase, putative	0.102	0.172	0.042	0.042	-0.148	0.202	-0.058	0.002	-0.188	0.192	-0.088	-0.048	-0.228	0.090	-0.001	0.002	-0.121	-0.211	0.864
PF3D7_082270(	PF3D7_0822700::thrombospondin-related protein 1, putative	0.183	0.163	0.283	0.283	0.273	0.233	-0.147	-0.207	-0.547	0.643	-0.187	-0.547	-0.427	0.228	0.120	-0.037	-0.387	-0.615	0.653
PF3D7_093270(	PF3D7_0932700::conserved Plasmodium protein, unknown function	-0.007	0.083	0.203	0.203	-0.017	0.183	0.053	-0.067	-0.227	0.303	-0.197	-0.247	-0.267	0.121	0.073	0.003	-0.237	-0.358	0.781
PF3D7_082400(	PF3D7_0824000::conserved Plasmodium protein, unknown function	0.107	0.257	0.347	0.157	-0.163	0.147	-0.353	-0.033	-0.133	0.587	-0.193	-0.073	-0.653	0.217	-0.123	0.140	-0.306	-0.523	0.696
PF3D7_142520(	PF3D7_1425200::enoyl-CoA hydratase, putative	0.004	0.004	0.384	0.044	0.094	0.234	-0.086	-0.026	-0.066	0.364	-0.346	-0.096	-0.506	0.109	0.081	0.091	-0.316	-0.425	0.745
PF3D7_020500(	PF3D7_0205000::conserved Plasmodium protein, unknown function	-0.188	0.422	0.152	0.012	-0.418	0.302	0.052	-0.088	0.292	1.182	-0.568	-0.528	-0.628	0.100	-0.021	0.462	-0.574	-0.674	0.627
PF3D7_113130(	PF3D7_1131300::conserved Plasmodium protein, unknown function	-0.148	0.392	-0.048	-0.018	-0.298	0.112	-0.108	-0.038	0.232	0.652	-0.298	-0.178	-0.258	0.045	-0.098	0.282	-0.244	-0.289	0.818
PF3D7_030490(	PF3D7_0304900::conserved protein, unknown function	0.015	0.265	0.015	0.015	-0.235	0.195	0.245	0.105	-0.095	1.285	-0.915	-0.675	-0.215	0.077	0.068	0.431	-0.602	-0.679	0.625
PF3D7_101450(	PF3D7_1014500::conserved Plasmodium protein, unknown function	-0.108	0.342	0.002	0.112	-0.038	0.262	0.342	-0.108	0.002	0.662	-0.668	-0.418	-0.378	0.087	0.188	0.185	-0.488	-0.575	0.671
PF3D7_041780(	PF3D7_0417800::cdc2-related protein kinase 1	0.058	0.198	0.228	0.108	-0.222	0.348	0.288	0.028	-0.042	0.498	-0.912	-0.292	-0.282	0.148	0.138	0.161	-0.496	-0.643	0.640
PF3D7_145070(	PF3D7_1450700::conserved Plasmodium protein, unknown function	-0.035	0.185	0.325	-0.045	-0.395	0.215	0.225	0.085	0.035	0.735	-0.785	-0.195	-0.355	0.108	0.015	0.285	-0.445	-0.553	0.682
PF3D7_021840(	PF3D7_0218400::ATP-dependent RNA helicase DDX47, putative	-0.041	0.159	0.419	0.099	-0.171	0.089	0.049	0.129	-0.111	0.809	-0.441	-0.441	-0.551	0.159	-0.011	0.276	-0.477	-0.637	0.643
PF3D7_081020(	PF3D7_0810200::ABC1 family, putative	-0.096	-0.066	0.274	0.054	-0.206	0.394	0.214	0.044	-0.136	0.594	-0.316	-0.526	-0.226	0.041	0.134	0.167	-0.356	-0.398	0.759
PF3D7_145280(	PF3D7_1452800::conserved Plasmodium protein, unknown function	-0.022	0.068	0.368	0.058	-0.332	0.388	0.108	-0.012	-0.022	0.898	-0.732	-0.582	-0.182	0.118	0.054	0.288	-0.499	-0.617	0.652
PF3D7_091530(	PF3D7_0915300::heptatricopeptide repeat-containing protein, putative	0.455	0.115	0.235	-0.115	0.025	0.545	-0.085	-0.135	-0.145	1.095	-0.745	-0.725	-0.515	0.172	0.161	0.271	-0.662	-0.834	0.561
PF3D7_144730(	PF3D7_1447300::ribosomal protein S14, mitochondrial, putative	0.245	0.185	0.205	0.005	-0.005	0.365	0.245	0.095	-0.075	1.035	-0.935	-0.695	-0.675	0.160	0.202	0.352	-0.768	-0.928	0.525
PF3D7_131900(	PF3D7_1319000::conserved Plasmodium protein, unknown function	0.047	-0.033	-0.023	0.217	-0.263	0.227	0.317	-0.093	0.117	0.527	-0.413	-0.493	-0.133	0.052	0.094	0.184	-0.346	-0.398	0.759
PF3D7_011420(	PF3D7_0114200::exported protein family 3	0.313	1.223	2.143	1.503	-1.157	-1.597	-1.007	0.213	0.373	1.883	-1.427	-0.857	-1.607	1.296	-1.254	0.823	-1.297	-2.593	0.166
PF3D7_132300(	PF3D7_1323000::beta-hydroxyacyl-ACP dehydratase	0.391	0.931	0.241	-0.029	-0.449	-0.029	-0.159	-0.029	0.311	0.791	-0.469	-0.709	-0.789	0.383	-0.213	0.357	-0.656	-1.039	0.487
PF3D7_132440(	PF3D7_1324400::PRELI domain-containing protein, putative	0.158	0.688	0.048	-0.042	-0.242	-0.102	-0.122	-0.032	0.028	0.518	-0.312	-0.332	-0.262	0.213	-0.155	0.172	-0.302	-0.515	0.700
PF3D7_102950(	PF3D7_1029500::conserved Plasmodium protein, unknown function	0.023	0.563	0.383	0.093	-0.267	-0.417	-0.397	0.093	-0.117	0.783	-0.137	-0.417	-0.187	0.266	-0.360	0.253	-0.247	-0.513	0.701
PF3D7_147610(	PF3D7_1476100::early transcribed membrane protein 14.2	0.107	0.567	0.197	0.147	-0.243	-0.503	-0.143	0.117	-0.113	0.827	-0.193	-0.533	-0.233	0.254	-0.296	0.277	-0.320	-0.574	0.672
PF3D7_070740(	PF3D7_0707400::AAA family ATPase, putative	0.067	0.567	0.397	0.157	-0.033	0.097	-0.153	-0.013	-0.423	0.387	-0.433	-0.513	-0.103	0.297	-0.030	-0.016	-0.350	-0.647	0.639
PF3D7_082490(	PF3D7_0824900::conserved Plasmodium protein, unknown function	0.044	0.334	0.424	0.234	-0.046	-0.006	-0.216	0.144	-0.236	0.484	-0.506	-0.526	-0.126	0.259	-0.089	0.131	-0.386	-0.645	0.639
PF3D7_091560(	PF3D7_0915600::conserved Plasmodium protein, unknown function	0.098	0.458	0.648	0.128	-0.132	-0.002	-0.022	0.108	-0.312	0.458	-0.592	-0.492	-0.352	0.333	-0.052	0.085	-0.478	-0.812	0.570
PF3D7_112470(	PF3D7_1124700::GrpE protein homolog, mitochondrial, putative	0.075	0.605	0.535	0.175	0.115	-0.155	-0.215	0.015	-0.305	0.515	-0.435	-0.525	-0.405	0.348	-0.085	0.075	-0.455	-0.803	0.573
PF3D7_102260(	PF3D7_1022600::kelch protein K10	0.120	0.120	0.370	0.170	-0.060	-0.090	-0.140	0.050	-0.270	0.380	-0.190	-0.310	-0.150	0.195	-0.097	0.053	-0.217	-0.412	0.752
PF3D7_010640(	PF3D7_0106400::pre-rRNA-processing protein TSR2, putative	0.202	0.712	0.382	0.102	-0.098	-0.268	-0.188	-0.068	-0.188	1.032	-0.528	-0.708	-0.378	0.349	-0.185	0.258	-0.538	-0.888	0.541
PF3D7_072250(	PF3D7_0722500::pre-mRNA-splicing factor CWC15, putative	0.128	0.398	0.338	-0.012	-0.252	-0.062	-0.042	0.008	-0.092	0.538	-0.462	-0.232	-0.262	0.213	-0.118	0.152	-0.318	-0.532	0.692
PF3D7_124530(	PF3D7_1245300::NEDD8-conjugating enzyme UBC12, putative	0.344	0.324	0.384	0.054	-0.086	-0.246	-0.356	0.104	-0.226	0.894	-0.276	-0.416	-0.496	0.276	-0.229	0.257	-0.396	-0.673	0.627
PF3D7_122500(	PF3D7_1225000::conserved Plasmodium protein, unknown function	0.140	0.190	0.320	0.030	0.110	-0.160	-0.170	0.120	-0.080	0.290	-0.390	-0.210	-0.190	0.170	-0.073	0.110	-0.263	-0.433	0.741
PF3D7_133080(	PF3D7_1330800::RNA-binding protein, putative	0.172	0.392	0.362	0.062	0.092	-0.258	-0.278	0.062	-0.188	0.582	-0.438	-0.358	-0.208	0.247	-0.148	0.152	-0.334	-0.582	0.668
PF3D7_113740(	PF3D7_1137400::UVB-resistance protein UVR8 homologue	0.334	0.054	0.284	0.044	0.044	0.024	0.004	-0.026	-0.176	0.194	-0.356	-0.256	-0.166	0.179	0.024	-0.003	-0.259	-0.438	0.738
PF3D7_132210(	PF3D7_1322100::histone-lysine N-methyltransferase SET2	0.282	0.132	0.302	0.062	-0.058	-0.098	-0.018	0.052	-0.148	0.182	-0.178	-0.228	-0.288	0.195	-0.058	0.029	-0.231	-0.426	0.744
PF3D7_060190(	PF3D7_0601900::conserved Plasmodium protein, unknown function	0.094	0.454	0.204	0.104	0.024	-0.406	-0.266	-0.046	-0.296	0.904	-0.376	-0.136	-0.256	0.214	-0.216	0.187	-0.256	-0.470	0.722
PF3D7_090260(	PF3D7_0902600::serine/threonine protein kinase, FIKK family	0.205	0.145	0.125	0.055	-0.275	-0.335	-0.335	-0.195	-0.085	1.225	-0.115	-0.225	-0.185	0.132	-0.315	0.315	-0.175	-0.308	0.808
PF3D7_110920(	PF3D7_1109200::conserved Plasmodium protein, unknown function	0.209	0.089	0.249	0.069	-0.181	-0.061	-0.121	-0.161	-0.261	0.919	-0.341	-0.141	-0.271	0.154	-0.121	0.166	-0.251	-0.405	0.755
PF3D7_102790(	PF3D7_1027900::palmitoyltransferase DHHC10, putative	-0.028	0.242	0.132	0.442	0.102	-0.068	-0.298	-0.128	-0.088	0.972	-0.578	-0.378	-0.328	0.197	-0.088	0.252	-0.428	-0.625	0.648
PF3D7_103290(	PF3D7_1032900::RNA polymerase II-associated protein 1, putative	-0.035	-0.025	0.255	0.155	-0.035	-0.145	-0.145	0.015	-0.105	0.695	-0.205	-0.275	-0.145	0.087	-0.109	0.201	-0.209	-0.296	0.815
PF3D7_051710(	PF3D7_0517100::conserved protein, unknown function	0.248	-0.082	0.278	-0.002	-0.132	0.008	-0.112	0.118	-0.222	1.618	-0.582	-0.732	-0.412	0.111	-0.078	0.505	-0.575	-0.686	0.622
PF3D7_142460(	PF3D7_1424600::small nucleolar RNA snoR28	0.235	0.165	0.165	0.105	-0.125	0.035	-0.185	0.085	-0.145	1.125	-0.535	-0.495	-0.435	0.168	-0.091	0.355	-0.488	-0.656	0.635
PF3D7_010840(	PF3D7_0108400::mitochondrial carrier protein, putative	0.046	0.236	0.506	0.196	-0.144	0.036	-0.444	0.006	-0.024	1.206	-0.624	-0.504	-0.494	0.246	-0.184	0.396	-0.541	-0.787	0.580
PF3D7_142450(	PF3D7_1424500::small nucleolar RNA snoR27	-0.022	0.258	0.218	0.058	-0.042	-0.092	-0.302	0.268	-0.032	1.298	-0.672	-0.502	-0.432	0.128	-0.146	0.511	-0.536	-0.663	0.631
PF3D7_145360(	PF3D7_1453600::RAP protein, putative	-0.051	0.119	0.249	-0.031	0.159	-0.211	-0.341	0.189	-0.081	1.009	-0.501	-0.341	-0.171	0.072	-0.131	0.373	-0.337	-0.409	0.753
PF3D7_124490(	PF3D7_1244900::conserved Plasmodium protein, unknown function	0.072	0.512	0.012	-0.088	-0.158	-0.178	-0.258	-0.078	0.092	0.962	-0.428	-0.288	-0.178	0.127	-0.198	0.326	-0.298	-0.425	0.745
PF3D7_141910(	PF3D7_1419100::ATP-dependent RNA helicase DDX55	-0.082	0.418	0.178	-0.042	-0.252	-0.202	-0.032	0.028	-0.122	1.518	-0.672	-0.522	-0.222	0.118	-0.162	0.475	-0.472	-0.590	0.664
PF3D7_147570(	PF3D7_1475700::tubulin epsilon chain, putative	-0.148	0.752	0.042	0.212	-0.248	-0.688	-0.408	0.622	0.362	2.572	-0.758	-1.378	-0.928	0.214	-0.448	1.185	-1.022	-1.236	0.425

PF3D7_052230(	PF3D7_0522300::18S rRNA (guanine-N(7))-methyltransferase, putative	0.228	0.408	0.748	0.188	0.068	-0.362	-0.242	0.038	-0.362	-0.162	-0.342	-0.342	0.128	0.393	-0.178	-0.162	-0.185	-0.578	0.670
PF3D7_062850(	PF3D7_0628500::conserved Plasmodium protein, unknown function	0.808	0.348	0.228	0.098	0.328	0.298	-0.292	-0.232	-0.752	-0.392	-0.202	-0.202	-0.042	0.371	0.112	-0.458	-0.148	-0.519	0.698
PF3D7_062120(	PF3D7_0621200::pyridoxine biosynthesis protein PDX1	0.904	0.154	0.584	0.104	0.354	-0.006	-0.386	0.144	-0.296	-0.406	-0.306	-0.506	-0.336	0.436	-0.013	-0.186	-0.383	-0.819	0.567
PF3D7_070370(	PF3D7_0703700::conserved protein, unknown function	0.395	0.115	0.175	0.245	0.425	0.185	-0.125	0.225	-0.365	-0.395	-0.285	-0.375	-0.225	0.233	0.162	-0.178	-0.295	-0.528	0.694
PF3D7_083140(	PF3D7_0831400::Plasmodium exported protein, unknown function	0.725	0.405	0.595	0.355	0.935	0.375	-0.365	0.785	-0.755	-0.885	-0.555	-0.735	-0.885	0.520	0.315	-0.285	-0.725	-1.245	0.422
PF3D7_072920(	PF3D7_0729200::1-cys peroxiredoxin	0.236	0.126	0.256	0.276	0.106	0.076	-0.214	0.226	-0.114	-0.334	-0.154	-0.214	-0.274	0.224	-0.011	-0.074	-0.214	-0.438	0.738
PF3D7_121530(	PF3D7_1215300::10 kDa chaperonin	0.501	0.661	0.791	0.661	0.581	0.271	-0.389	0.121	-0.189	-0.849	-0.369	-0.929	-0.859	0.653	0.154	-0.306	-0.719	-1.373	0.386
PF3D7_102750(	PF3D7_1027500::conserved Plasmodium protein, unknown function	0.460	0.110	0.580	0.500	0.440	0.150	-0.440	0.080	-0.230	-0.470	-0.460	-0.450	-0.270	0.413	0.050	-0.207	-0.393	-0.806	0.572
PF3D7_102900(	PF3D7_1029000::conserved Plasmodium protein, unknown function, pse	0.145	0.165	0.335	0.285	0.325	-0.015	-0.245	-0.075	-0.365	-0.165	0.045	-0.355	-0.075	0.232	0.021	-0.202	-0.129	-0.361	0.779
PF3D7_071870(	PF3D7_0718700::conserved Plasmodium membrane protein, unknown fu	0.258	0.338	0.348	0.458	-0.072	0.208	-0.062	0.058	-0.722	-0.192	-0.042	-0.312	-0.262	0.350	0.024	-0.286	-0.206	-0.556	0.680
PF3D7_082410(	PF3D7_0824100::3'-5' exonuclease, putative	0.130	0.250	0.340	0.280	0.010	-0.030	0.010	0.060	-0.330	-0.150	-0.110	-0.400	-0.060	0.250	-0.003	-0.140	-0.190	-0.440	0.737
PF3D7_101850(	PF3D7_1018500::pre-mRNA-splicing factor RDS3, putative	0.368	0.148	0.498	0.128	0.318	-0.072	-0.022	-0.072	-0.302	-0.232	0.048	-0.442	-0.362	0.285	0.074	-0.202	-0.252	-0.538	0.689
PF3D7_103230(	PF3D7_1032300::conserved protein, unknown function	0.145	0.295	0.535	0.115	0.045	0.025	-0.115	0.125	-0.355	-0.095	-0.055	-0.345	-0.315	0.272	-0.015	-0.109	-0.239	-0.511	0.702
PF3D7_122780(	PF3D7_1227800::elongator complex protein 3, putative	0.333	0.243	0.603	0.103	0.163	0.063	-0.247	-0.017	-0.447	-0.097	0.013	-0.327	-0.387	0.321	-0.007	-0.187	-0.234	-0.554	0.681
PF3D7_120510(	PF3D7_1205100::O-phosphoseryl-tRNA(Sec) selenium transferase, puta	0.239	0.179	0.429	0.199	0.099	0.119	-0.151	-0.111	-0.191	0.119	-0.211	-0.441	-0.281	0.262	0.023	-0.061	-0.311	-0.573	0.672
PF3D7_142590(	PF3D7_1425900::conserved Plasmodium protein, unknown function	0.225	0.295	0.455	0.395	0.065	0.195	-0.085	0.075	-0.355	-0.045	-0.085	-0.555	-0.575	0.342	0.058	-0.109	-0.405	-0.748	0.596
PF3D7_143460(	PF3D7_1434600::methionine aminopeptidase 2	0.298	0.148	0.348	0.258	0.018	0.178	-0.202	0.048	-0.402	0.028	-0.092	-0.342	-0.282	0.263	-0.002	-0.109	-0.239	-0.502	0.706
PF3D7_092390(	PF3D7_0923900::polyadenylate-binding protein 2, putative	0.187	0.097	0.077	0.197	0.197	0.217	-0.053	0.067	-0.033	-0.063	-0.223	-0.393	-0.273	0.139	0.120	-0.010	-0.296	-0.436	0.739
PF3D7_135530(	PF3D7_1355300::histone-lysine N-methyltransferase, putative	0.276	0.106	0.176	0.176	0.136	0.026	-0.064	0.166	-0.264	0.056	-0.254	-0.504	-0.034	0.184	0.033	-0.014	-0.264	-0.448	0.733
PF3D7_143510(	PF3D7_1435100::ribonuclease H2 subunit B, putative	0.099	0.249	0.249	0.639	0.249	0.309	0.279	0.279	-0.071	0.109	-0.701	-1.671	-0.021	0.309	0.279	0.106	-0.797	-1.107	0.464
PF3D7_053210(	PF3D7_0532100::early transcribed membrane protein 5	0.278	0.798	0.808	0.198	-0.172	0.498	-0.102	0.268	-0.312	-1.522	-0.302	-0.032	-0.412	0.521	0.075	-0.522	-0.248	-0.769	0.587
PF3D7_080790(	PF3D7_0807900::tyrosine--tRNA ligase	0.157	0.127	0.127	0.207	0.327	0.327	-0.003	0.107	-0.183	-0.953	-0.223	-0.053	0.037	0.154	0.217	-0.343	-0.080	-0.234	0.850
PF3D7_093370(	PF3D7_0933700::conserved Plasmodium protein, unknown function	0.182	0.232	0.192	0.352	-0.018	0.292	0.042	-0.008	0.062	-0.988	-0.428	0.092	0.002	0.239	0.105	-0.312	-0.112	-0.351	0.784
PF3D7_052780(	PF3D7_0527800::tRNA Leucine	0.482	0.682	0.212	0.202	0.412	0.192	-0.338	-0.278	-0.128	-0.578	-0.408	-0.118	-0.338	0.395	0.089	-0.328	-0.288	-0.683	0.623
PF3D7_081750(	PF3D7_0817500::histidine triad nucleotide-binding protein 1	0.223	0.353	0.053	0.013	0.373	0.093	-0.087	0.053	0.213	-0.717	-0.297	-0.147	-0.127	0.161	0.126	-0.150	-0.190	-0.351	0.784
PF3D7_114420(	PF3D7_1144200::conserved Plasmodium protein, unknown function	0.488	0.218	0.148	0.438	0.118	0.028	-0.262	0.038	0.118	-0.742	-0.042	-0.172	-0.372	0.323	-0.039	-0.196	-0.196	-0.518	0.698
PF3D7_050580(	PF3D7_0505800::small ubiquitin-related modifier	0.373	0.423	0.043	0.063	0.233	0.523	0.183	0.183	0.163	-0.847	-0.747	-0.407	-0.187	0.226	0.313	-0.167	-0.447	-0.673	0.627
PF3D7_090930(	PF3D7_0909300::apoptosis-related protein	0.389	0.389	0.139	0.199	-0.001	0.529	0.339	0.219	0.189	-0.971	-0.661	-0.411	-0.351	0.279	0.289	-0.187	-0.474	-0.753	0.593
PF3D7_090480(	PF3D7_0904800::replication protein A1, small fragment	0.233	0.243	0.053	0.173	0.053	0.483	0.193	-0.047	0.173	-0.617	-0.557	-0.187	-0.197	0.176	0.243	-0.164	-0.314	-0.489	0.712
PF3D7_061300(	PF3D7_0613000::conserved Plasmodium protein, unknown function	0.156	0.586	0.036	0.586	0.566	0.696	0.256	0.106	-0.024	-1.034	-0.984	-0.484	-0.464	0.341	0.506	-0.317	-0.644	-0.985	0.505
PF3D7_062670(	PF3D7_0626700::ATPase	0.300	0.270	0.140	0.400	0.530	0.380	0.190	0.070	-0.080	-0.800	-0.790	-0.400	-0.210	0.278	0.367	-0.270	-0.467	-0.744	0.597
PF3D7_031760(	PF3D7_0317600::40S ribosomal protein S11, putative	0.372	0.542	0.042	0.472	0.362	0.572	-0.008	0.062	0.082	-0.948	-0.728	-0.448	-0.378	0.357	0.309	-0.268	-0.518	-0.875	0.545
PF3D7_135140(	PF3D7_1351400::60S ribosomal protein L17, putative	0.639	0.409	0.109	0.499	0.439	0.729	0.119	0.179	-0.091	-1.311	-0.751	-0.601	-0.371	0.414	0.429	-0.407	-0.574	-0.988	0.504
PF3D7_062684(	PF3D7_0626840::small nucleolar RNA snoR35	0.721	0.081	0.091	0.481	0.121	0.521	0.021	0.291	-0.069	-0.919	-0.789	-0.359	-0.189	0.343	0.221	-0.233	-0.446	-0.789	0.579
PF3D7_101940(	PF3D7_1019400::60S ribosomal protein L30e, putative	0.481	0.091	0.151	0.211	0.331	0.241	-0.059	0.141	-0.009	-0.519	-0.509	-0.329	-0.219	0.233	0.171	-0.129	-0.353	-0.586	0.666
PF3D7_102960(	PF3D7_1029600::adenosine deaminase	0.552	0.232	0.152	0.282	0.502	0.582	0.242	0.282	-0.118	-0.938	-1.178	-0.588	0.002	0.304	0.442	-0.258	-0.588	-0.893	0.539
PF3D7_052400(	PF3D7_0524000::karyopherin beta	0.614	0.164	-0.026	-0.006	0.434	0.734	0.374	0.124	-0.226	-1.356	-0.556	-0.206	-0.066	0.186	0.514	-0.486	-0.276	-0.463	0.726
PF3D7_010920(	PF3D7_0109200::cleavage and polyadenylation specificity factor subuni	0.535	0.455	-0.035	0.105	0.135	0.655	0.345	0.085	-0.185	-1.035	-0.505	-0.255	-0.295	0.265	0.378	-0.379	-0.352	-0.617	0.652
PF3D7_051230(	PF3D7_0512300::CDK-activating kinase assembly factor MAT1	0.641	0.291	-0.009	0.111	0.351	0.941	0.461	-0.089	-0.179	-1.259	-0.709	-0.369	-0.179	0.258	0.584	-0.509	-0.419	-0.678	0.625
PF3D7_051500(	PF3D7_0515000::pre-mRNA-splicing factor CWC2, putative	0.598	0.218	0.058	0.058	0.128	0.678	0.448	-0.002	-0.222	-0.982	-0.492	-0.242	-0.242	0.233	0.418	-0.402	-0.326	-0.558	0.679
PF3D7_070350(	PF3D7_0703500::erythrocyte membrane-associated antigen	0.677	0.317	-0.053	0.017	0.177	0.847	0.507	0.117	-0.103	-1.253	-0.753	-0.263	-0.233	0.239	0.510	-0.413	-0.416	-0.656	0.635
PF3D7_081270(	PF3D7_0812700::U1 small nuclear ribonucleoprotein C, putative	1.123	0.403	-0.057	-0.117	0.263	1.143	0.673	0.053	-0.227	-1.577	-0.897	-0.327	-0.457	0.338	0.693	-0.584	-0.560	-0.898	0.537
PF3D7_091080(	PF3D7_0910800::cytosolic Fe-S cluster assembly factor NBP35, putative	0.311	0.221	0.021	0.071	0.081	0.481	0.321	0.141	-0.269	-0.769	-0.379	-0.109	-0.119	0.156	0.294	-0.299	-0.203	-0.358	0.780
PF3D7_060450(	PF3D7_0604500::GYF domain-containing protein, putative	0.345	0.175	-0.055	-0.035	0.085	0.325	0.215	0.075	-0.105	-0.635	-0.155	-0.095	-0.145	0.108	0.209	-0.221	-0.131	-0.239	0.847
PF3D7_124680(	PF3D7_1246800::signal recognition particle receptor subunit beta, putati	0.564	0.244	-0.076	-0.056	0.194	0.744	0.314	-0.026	-0.216	-0.836	-0.236	-0.276	-0.336	0.169	0.417	-0.359	-0.283	-0.452	0.731
PF3D7_040540(	PF3D7_0405400::pre-mRNA-processing-splicing factor 8, putative	0.728	-0.042	0.098	-0.052	0.228	0.808	0.378	0.098	-0.112	-1.182	-0.672	-0.192	-0.082	0.183	0.471	-0.399	-0.316	-0.498	0.708
PF3D7_080200(	PF3D7_0802000::glutamate dehydrogenase, putative	0.546	0.006	0.126	0.036	0.156	0.556	0.236	0.136	-0.124	-0.854	-0.554	-0.074	-0.194	0.179	0.316	-0.281	-0.274	-0.453	0.731
PF3D7_091330(	PF3D7_0913300::conserved protein, unknown function	0.675	0.025	0.175	0.125	0.195	0.515	0.325	0.115	-0.075	-0.925	-0.735	-0.195	-0.225	0.250	0.345	-0.295	-0.385	-0.635	0.644
PF3D7_124170(	PF3D7_1241700::replication factor C subunit 4, putative	0.745	-0.035	0.145	0.045	0.335	0.635	0.325	0.215	0.025	-1.295	-0.605	-0.165	-0.375	0.225	0.432	-0.351	-0.381	-0.607	0.657
PF3D7_052680(	PF3D7_0526800::conserved Plasmodium protein, unknown function	0.623	0.123	0.023	0.123	0.373	0.813	0.373	0.203	-0.397	-0.897	-0.817	-0.247	-0.297	0.223	0.520	-0.364	-0.454	-0.677	0.626
PF3D7_111960(	PF3D7_1119600::ATP-dependent zinc metalloprotease FTSH, putative	0.775	0.065	-0.005	0.175	0.425	0.905	0.505	0.185	-0.375	-1.315	-0.835	-0.315	-0.185	0.252	0.611	-0.502	-0.445	-0.698	0.617
PF3D7_110730(	PF3D7_1107300::polyadenylate-binding protein-interacting protein 1, pu	0.368	-0.032	-0.002	-0.042	0.088	0.468	0.278	0.158	-0.162	-0.612	-0.292	-0.152	-0.062	0.073	0.278	-0.206	-0.169	-0.242	0.846
PF3D7_093500(	PF3D7_0935000::U2 small nuclear ribonucleoprotein B'', putative	0.592	0.142	-0.018	0.092	0.382	0.792	0.302	-0.228	-0.028	-1.168	-0.508	-0.058	-0.288	0.202	0.492	-0.475	-0.285	-0.487	0.714
PF3D7_010800(	PF3D7_0108000::proteasome subunit beta type-3, putative	0.378	0.018	0.128	0.108	0.278	0.588	0.218	0.018	-0.042	-0.772	-0.552	-0.042	-0.332	0.158	0.362	-0.265	-0.308	-0.467	0.724
PF3D7_041680(	PF3D7_0416800::small GTP-binding protein sar1	0.357	0.097	0.057	0.257	0.297	0.507	0.187	0.067	0.077	-0.803	-0.713	-0.163	-0.223	0.192	0.330	-0.220	-0.366	-0.558	0.679
PF3D7_092010(	PF3D7_0920100::chaperone, putative	0.707	0.197	0.177	0.287	0.407	0.547	0.267	0.027	-0.153	-1.243	-0.803	-0.153	-0.263	0.342	0.407	-0.456	-0.406	-0.748	0.595
PF3D7_092250(	PF3D7_0922500::phosphoglycerate kinase	0.497	0.347	0.307	0.167	0.637	0.577	0.077	0.197	-0.153	-1.173	-0.913	-0.353	-0.213	0.329	0.430	-0.376	-0.493	-0.823	0.565
PF3D7_145110(	PF3D7_1451100::elongation factor 2	0.504	0.174	0.374	0.254	0.564	0.774	0.164	0.214	-0.166	-1.296	-0.936	-0.306	-0.316	0.326	0.501	-0.416	-0.519	-0.846	0.556
PF3D7_062940(	PF3D7_0629400::polyadenylate-binding protein 3, putative	0.405	0.065	0.295	0.225	0.255	0.435	0.335	0.165	-0.145	-0.685	-0.765	-0.355	-0.235	0.248	0.342	-0.221	-0.451	-0.699	0.616
PF3D7_030890(	PF3D7_0308900::splicing factor 3B subunit 1, putative	0.419	0.059	0.149	0.069	0.169	0.579	0.419	0.139	-0.191	-0.761	-0.601	-0.221	-0.231	0.174	0.389	-0.271	-0.351	-0.525	0.695
PF3D7_143540(	PF3D7_1435400::ER membrane protein complex subunit 4, putative	0.488	0.058	0.188	0.018	0.278	0.488	0.478	0.108	-0.092	-0.712	-0.692	-0.392	-0.212	0.188	0.414	-0.232	-0.432	-0.620	0.651
PF3D7_030210(	PF3D7_0302100::serine/threonine protein kinase	0.297	0.147	0.317	0.087	0.177	0.437	0.237	0.047	-0.183	-0.633	-0.543	-0.213	-0.173	0.212	0.284	-0.256	-0.310	-0.522	0.697
PF3D7_141060(	PF3D7_1410600::eukaryotic translation initiation factor 2 subunit gamma	0.614	0.454	0.314	0.214	0.194	0.724	0.484	0.174	-0.276	-1.256	-0.976	-0.486	-0.176	0.399	0.467	-0.453	-0.546	-0.945	0.519
PF3D7_125270(	PF3D7_1252700::Plasmodium exported protein (PHISTb), unknown func	0.471	0.231	0.151	0.061	0.081	0.351	0.381	0.171	-0.029	-0.759	-0.739	-0.219	-0.149	0.228	0.271	-0.206	-0.369	-0.598	0.661
PF3D7_091830(	PF3D7_0918300::eukaryotic translation initiation factor 3 subunit F, puta	0.605	0.305	0.175	0.065	0.445	1.005	0.185	-0.005	-0.465	-0.965	-0.635	-0.395	-0.325	0.288	0.545	-0.478	-0.451	-0.739	0.599
PF3D7_081060(	PF3D7_0810600::ATP-dependent RNA helicase DBP1, putative	0.879	0.359	0.299	0.169	0.389	0.839	0.309	0.029	-0.321	-1.171	-0.701	-0.541	-0.541	0.427	0.513	-0.487	-0.594	-1.021	0.493
PF3D7_131080(	PF3D7_1310800::tetratricopeptide repeat protein, putative	0.401	0.261	0.311	0.121	0.251	0.451	0.211	0.101	-0.249	-0.769	-0.449	-0.259	-0.379	0.273	0.304	-0.306	-0.363	-0.636	0.644
PF3D7_041590(	PF3D7_0415900::60S ribosomal protein L15, putative	0.827	0.527	0.347	0.037	0.427	0.797	0.127	0.027	-0.093	-1.293	-0.773	-0.353	-0.603	0.434	0.450	-0.453	-0.576	-1.011	0.496
PF3D7_110540(	PF3D7_1105400::40S ribosomal protein S4, putative	0.734	0.364	0.074	0.174	0.384	0.644	0.164	0.124	-0.166	-0.976	-0.756	-0.436	-0.326	0.336	0.397	-0.339	-0.506	-0.843	0.558
PF3D7_132490(	PF3D7_1324900::L-lactate dehydrogenase	0.794	0.614	0.184	0.064	0.514	0.934	0.134	0.104	-0.236	-1.526	-0.756	-0.506	-0.316	0.414	0.527	-0.553	-0.526	-0.940	0.521
PF3D7_146870(	PF3D7_1468700::eukaryotic initiation factor 4A	0.910	0.510	0.190	0.150	0.670	0.800	0.200	0.090	-0.250	-1.520	-0.840	-0.460	-0.450	0.440	0.557	-0.560	-0.583	-1.023	0.492
PF3D7_141480(	PF3D7_1414800::small nuclear ribonucleoprotein-associated protein B, p	1.042	0.822	-0.058	0.052	0.412	0.982	0.182	-0.018	-0.258	-1.338	-0.888	-0.498	-0.438	0.465	0.526	-0.538	-0.608	-1.073	0.475
PF3D7_061080(	PF3D7_0610800::transketolase	0.980	-0.020	0.210	0.160	0.630	0.770	0.060	-0.110	-0.380	-1.280	-0.120	-0.560	-0.340	0.333	0.487	-0.590	-0.340	-0.673	0.627
PF3D7_133280(	PF3D7_1332800::eukaryotic translation initiation factor 6, putative	1.078	-0.042	0.398	0.068	0.718	0.918	0.158	-0.022	-0.442	-1.612	-0.442	-0.482	-0.302	0.376	0.598	-0.692	-0.408	-0.784	0.581
PF3D7_113930(	PF3D7_1139300::AP2 domain transcription factor, putative	0.341	-0.019	0.231	0.031	0.291	0.311	0.201	0.091	-0.289	-0.489	-0.309	-0.159	-0.229	0.146	0.267	-0.229	-0.233	-0.378	0.769
PF3D7_112700(	PF3D7_1127000::protein phosphatase, putative	0.515	0.185	0.105	0.105	0.425	0.375	0.185	0.195	-0.325	-0.825	-0.415	-0.255	-0.275	0.228	0.329	-0.318	-0.315	-0.543	0.687
PF3D7_123020(	PF3D7_1230200::vacuolar transporter chaperone, putative, pseudogene	0.555	0.135	0.255	0.255	0.395	0.555	0.085	0.125	-0.615	-0.915	-0.325	-0.295	-0.215	0.300	0.345	-0.468	-0.278	-0.578	0.670
PF3D7_092550(	PF3D7_0925500::thioredoxin-like protein 2	0.414	0.094	0.434	0.134	0.244	0.224	0.144	-0.096	-0.076	-0.666	-0.036	-0.336	-0.476	0.269	0.204	-0.279	-0.283	-0.552	0.682
PF3D7_070800(	PF3D7_0708000::cytoskeleton associated protein, putative	0.519	0.519	0.289	0.109	0.069	0.449	0.109	-0.181	0.009	-0.841	-0.281	-0.211	-0.561	0.359	0.209	-0.337	-0.351	-0.710	0.611
PF3D7_123570(	PF3D7_1235700::ATP synthase subunit beta, mitochondrial	0.617	0.247	0.327	0.117	0.187	0.637	0.167	0.047	0.007	-1.303	-0.393	-0.183	-0.473	0.327	0.330	-0.416	-0.350	-0.677	0.626
PF3D7_090770(	PF3D7_0907700::proteasome activator 28	0.612	0.312	0.202	0.192	0.112	0.302	0.332	-0.048	-0.418	-1.168	-0.248	-0.038	-0.138	0.329	0.248	-0.545	-0.142	-0.471	0.722
PF3D7_144620(	PF3D7_1446200::M17 leucyl aminopeptidase	0.878	0.578	0.308	0.128	0.348	0.188	0.048	-0.072	-0.142	-1.632	-0.232	-0.112	-0.282	0.473	0.194	-0.616	-0.209	-0.682	0.623
PF3D7_041820(	PF3D7_0418200::eukaryotic translation initiation factor 3 subunit M, puta	0.587	0.247	0.097	0.077	0.327	0.277	0.087	-0.053	-0.123	-0.333	-0.513	-0.273	-0.403	0.252	0.230	-0.170	-0.396	-0.648	0.638
PF3D7_051730(	PF3D7_0517300::serine/arginine-rich splicing factor 1	0.712	0.062	0.212	0.162	0.242	0.272	0.022	0.092	-0.158	-0.448	-0.518	-0.278	-0.368	0.287	0.178	-0.172	-0.388	-0.675	0.626
PF3D7_072340(	PF3D7_0723400::conserved Plasmodium protein, unknown function	0.747	0.397	0.297	-0.003	0.087	0.437	0.157	0.027	-0.293	-0.573	-0.483	-0.303	-0.493	0.359	0.227	-0.280	-0.426	-0.786	0.580
PF3D7_022070(	PF3D7_0220700::Plasmodium exported protein (hyp9), unknown functio	1.197	0.227	0.287	0.117	0.377	0.547	0.277	0.057	-0.503	-0.993	-0.413	-0.433	-0.743	0.457	0.400	-0.480	-0.530	-0.987	0.505
PF3D7_120660(	PF3D7_1206600::DNA-directed RNA polymerase III subunit RPC2, putat	0.822	0.202	0.222	-0.038	0.262	0.482	0.002	0.002	-0.398	-0.558	-0.348	-0.378	-0.268	0.302	0.248	-0.318	-0.332	-0.633	0.645
PF3D7_143470(	PF3D7_1434700::mitochondrial import inner membrane translocase subu	1.052	0.242	0.192	-0.078	0.372	0.812	0.062	-0.128	-0.268	-0.868	-0.328	-0.518	-0.538	0.352	0.415	-0.422	-0.462	-0.813	0.569
PF3D7_061700(	PF3D7_0617000::mitochondrial import receptor subunit TOM40, putative	0.732	0.282	0.362	-0.018	0.482	0.822	0.172	0.142	-0.658	-0.548	-0.548	-0.748	-0.478	0.340	0.492	-0.354	-0.591	-0.931	0.525
PF3D7_061290(	PF3D7_0612900::nucleolar GTP-binding protein 1, putative	0.649	0.439	0.569	0.079	0.299	0.479	0.069	0.049	-0.601	-0.451	-0.731	-0.481	-0.371	0.434	0.283	-0.334	-0.527	-0.962	0.513
PF3D7_101020(	PF3D7_1010200::DNA2/NAM7 helicase, putative	0.478	0.238	0.368	0.168	0.138	0.318	0.028	0.098	-0.332	-0.322	-0.362	-0.402	-0.412	0.313	0.161	-0.186	-0.392	-0.705	0.613
PF3D7_101530(	PF3D7_1015300::methionine aminopeptidase 1b, putative	0.716	0.566	0.566	0.356	0.466	0.576	-0.044	0.296	-0.714	-0.874	-0.774	-0.544	-0.594	0.551	0.333	-0.431	-0.637	-1.188	0.439
PF3D7_020370(	PF3D7_0203700::protein MAK16, putative	0.723	0.273	0.613	0.213	0.123	0.103	-0.127	0.173	-0.307	-0.497	-0.547	-0.357	-0.387	0.456	0.033	-0.210	-0.430	-0.886	0.541
PF3D7_030850(	PF3D7_0308500::activator of Hsp90 ATPase, putative	0.998	0.608	0.798	0.408	0.298	0.158	-0.122	-0.122	-0.292	-0.562	-0.862	-0.592	-0.722	0.703	0.112	-0.325	-0.725	-1.428	0.372
PF3D7_131930(	PF3D7_1319300::tRNA (guanine(26)-N(2))-dimethyltransferase, putative	0.411	0.221	0.331	0.121	0.171	0.071	-0.039	0.101	-0.169	-0.189	-0.489	-0.269	-0.269	0.271	0.067	-0.086	-0.343	-0.613	0.654
PF3D7_101560(	PF3D7_1015600::heat shock protein 60	0.479	0.549	0.709	0.089	0.319	0.209	-0.051	0.049	-0.401	-0.401	-0.821	-0.451	-0.281	0.457	0.159	-0.251	-0.517	-0.974	0.509
PF3D7_060210(	PF3D7_0602100::ATP-dependent RNA helicase MTR4	0.485	0.155	0.365	0.045	0.285	0.245	-0.015	-0.065	-0.275	-0.385	-0.265	-0.325	-0.255	0.263	0.172	-0.241	-0.281	-0.544	0.686
PF3D7_010960(	PF3D7_0109600::cold-shock protein, putative	0.899	0.519	0.559	0.119	0.389	0.239	-0.061	-0.241	-0.231	-0.701	-0.411	-0.561	-0.521	0.524	0.189	-0.391	-0.497	-1.022	0.493
PF3D7_121370(	PF3D7_1213700::DNA-directed RNA polymerases I, II, and III subunit RF	0.878	0.538	0.568	0.118	0.528	0.318	-0.022	-0.102	-0.382	-0.642	-0.642	-0.572	-0.592	0.526	0.275	-0.375	-0.602	-1.128	0.458

PF3D7_082920(	PF3D7_0829200::prohibitin 1, putative	0.635	0.365	0.435	0.005	0.295	0.445	-0.045	0.105	-0.295	-0.405	-0.535	-0.665	-0.335	0.360	0.231	-0.199	-0.512	-0.872	0.547
PF3D7_147100(	PF3D7_1471000::RNA 3'-terminal phosphate cyclase-like protein, putativ	0.757	0.297	0.477	0.077	0.267	0.197	-0.203	-0.003	-0.113	-0.393	-0.493	-0.563	-0.303	0.402	0.087	-0.170	-0.453	-0.855	0.553
PF3D7_021920(	PF3D7_0219200::40S ribosomal protein S30	0.624	1.154	0.104	0.364	0.264	0.584	0.034	0.174	-0.056	-1.056	-1.096	-0.726	-0.366	0.561	0.294	-0.313	-0.729	-1.291	0.409
PF3D7_030330(	PF3D7_0303300::DNA-directed RNA polymerases I, II, and III subunit RF	0.565	0.795	0.155	0.275	0.135	0.455	0.085	0.105	-0.215	-0.765	-0.725	-0.565	-0.305	0.448	0.225	-0.291	-0.531	-0.979	0.507
PF3D7_061170(	PF3D7_0611700::60S ribosomal protein L39	0.653	1.083	0.163	0.443	0.283	0.283	-0.047	-0.047	0.113	-1.207	-0.817	-0.467	-0.437	0.586	0.173	-0.380	-0.574	-1.159	0.448
PF3D7_070570(	PF3D7_0705700::40S ribosomal protein S29, putative	0.335	0.705	-0.005	0.255	0.205	0.455	0.025	0.005	0.145	-0.485	-0.775	-0.475	-0.385	0.322	0.228	-0.112	-0.545	-0.868	0.548
PF3D7_021340(	PF3D7_0213400::protein kinase 7	0.075	0.395	0.115	0.265	0.085	0.255	0.105	0.025	-0.115	-0.345	-0.395	-0.265	-0.195	0.212	0.148	-0.145	-0.285	-0.498	0.708
PF3D7_100490(	PF3D7_1004900::conserved protein, unknown function	0.337	0.837	0.337	0.277	0.007	0.337	0.017	-0.063	-0.113	-0.533	-0.623	-0.343	-0.473	0.447	0.120	-0.236	-0.480	-0.927	0.526
PF3D7_102330(	PF3D7_1023300::conserved protein, unknown function	0.122	0.632	0.112	0.352	0.052	0.172	-0.038	0.132	0.112	-0.528	-0.438	-0.268	-0.408	0.304	0.062	-0.095	-0.372	-0.676	0.626
PF3D7_040700(	PF3D7_0407000::conserved Plasmodium protein, unknown function	0.374	0.494	0.224	0.314	0.304	0.264	-0.046	-0.026	0.054	-0.566	-0.876	-0.536	0.024	0.351	0.174	-0.179	-0.463	-0.814	0.569
PF3D7_146770(	PF3D7_1467700::conserved Plasmodium protein, unknown function	0.310	0.460	0.280	0.260	0.210	0.340	0.070	0.020	0.130	-0.580	-0.700	-0.590	-0.210	0.328	0.207	-0.143	-0.500	-0.828	0.564
PF3D7_021420(	PF3D7_0214200::ribosomal protein L13, mitochondrial	0.658	0.968	0.248	0.138	0.388	0.658	0.038	-0.292	-0.182	-1.492	-0.752	-0.032	-0.342	0.503	0.361	-0.656	-0.376	-0.878	0.544
PF3D7_031680(	PF3D7_0316800::40S ribosomal protein S15A, putative	0.453	0.593	0.123	0.343	0.143	0.413	0.023	0.093	-0.017	-0.947	-0.657	-0.247	-0.317	0.378	0.193	-0.290	-0.407	-0.785	0.580
PF3D7_132500(	PF3D7_1325000::U6 snRNA-associated Sm-like protein LSm6, putative	0.704	0.714	0.134	0.204	0.264	0.574	0.314	0.044	-0.086	-1.346	-0.726	-0.256	-0.536	0.439	0.384	-0.463	-0.506	-0.945	0.519
PF3D7_061830(	PF3D7_0618300::60S ribosomal protein L27a, putative	0.757	0.987	0.587	0.327	0.477	0.497	0.037	-0.063	-0.143	-1.473	-0.963	-0.563	-0.463	0.664	0.337	-0.560	-0.663	-1.328	0.398
PF3D7_072160(	PF3D7_0721600::40S ribosomal protein S5, putative	0.555	0.755	0.545	0.145	0.425	0.315	0.035	-0.005	-0.235	-0.865	-0.875	-0.325	-0.465	0.500	0.258	-0.369	-0.555	-1.055	0.481
PF3D7_071960(	PF3D7_0719600::60S ribosomal protein L11a, putative	0.600	0.690	0.370	0.140	0.430	0.630	0.130	0.120	-0.200	-0.970	-0.910	-0.550	-0.480	0.450	0.397	-0.350	-0.647	-1.097	0.468
PF3D7_124270(	PF3D7_1242700::40S ribosomal protein S17, putative	0.652	0.822	0.462	0.142	0.122	0.352	0.082	0.092	-0.338	-0.958	-0.598	-0.228	-0.608	0.520	0.186	-0.401	-0.478	-0.998	0.501
PF3D7_082190(	PF3D7_0821900::conserved protein, unknown function	0.662	0.802	0.152	0.312	0.492	0.402	0.122	0.162	-0.588	-1.118	-0.758	-0.058	-0.578	0.482	0.338	-0.515	-0.465	-0.947	0.519
PF3D7_147410(	PF3D7_1474100::ribosomal protein L20, mitochondrial, putative	0.969	0.999	0.249	0.439	0.429	0.569	-0.111	-0.061	-0.451	-1.221	-1.001	-0.381	-0.431	0.664	0.296	-0.577	-0.604	-1.268	0.415
PF3D7_101790(	PF3D7_1017900::26S proteasome regulatory subunit p55, putative	0.242	0.402	0.202	0.112	0.052	0.082	0.042	0.032	-0.018	-0.548	-0.438	-0.108	-0.048	0.239	0.058	-0.178	-0.198	-0.438	0.738
PF3D7_132320(	PF3D7_1323200::V-type proton ATPase subunit G, putative	0.462	0.482	0.332	0.222	0.022	0.282	0.222	0.092	0.082	-0.908	-0.838	-0.138	-0.318	0.375	0.176	-0.244	-0.431	-0.806	0.572
PF3D7_021010(	PF3D7_0210100::60S ribosomal protein L37ae, putative	0.390	0.760	0.565	0.300	0.180	0.030	-0.205	0.125	-0.220	-0.500	-0.565	-0.280	-0.575	0.503	0.001	-0.199	-0.474	-0.977	0.508
PF3D7_030960(	PF3D7_0309600::60S acidic ribosomal protein P2	0.273	0.593	0.393	0.453	0.193	0.193	-0.107	-0.057	-0.147	-0.327	-0.577	-0.417	-0.467	0.428	0.093	-0.177	-0.487	-0.915	0.530
PF3D7_131780(	PF3D7_1317800::40S ribosomal protein S19	0.405	1.005	0.525	0.515	0.375	0.095	-0.105	0.105	-0.245	-0.955	-0.765	-0.475	-0.485	0.613	0.122	-0.365	-0.575	-1.188	0.439
PF3D7_146030(	PF3D7_1460300::60S ribosomal protein L29, putative	0.195	0.855	0.445	0.625	0.415	0.145	-0.215	0.095	-0.155	-0.795	-0.795	-0.415	-0.395	0.530	0.115	-0.285	-0.535	-1.065	0.478
PF3D7_146070(	PF3D7_1460700::60S ribosomal protein L27	0.567	1.017	0.517	0.517	0.357	0.087	-0.213	0.027	-0.103	-0.903	-1.063	-0.413	-0.393	0.654	0.077	-0.326	-0.623	-1.278	0.413
PF3D7_144330(	PF3D7_1443300::U6 snRNA-associated Sm-like protein LSm5, putative	0.363	0.733	0.283	0.303	0.253	0.113	0.013	0.143	-0.027	-0.747	-0.657	-0.267	-0.507	0.421	0.126	-0.210	-0.477	-0.898	0.537
PF3D7_146130(	PF3D7_1461300::40S ribosomal protein S28e, putative	0.323	0.703	0.603	0.373	0.183	0.023	0.013	0.113	-0.177	-0.917	-0.707	-0.217	-0.317	0.501	0.073	-0.327	-0.414	-0.914	0.531
PF3D7_103070(	PF3D7_1030700::RNA methyltransferase, putative	0.452	0.722	0.562	0.182	0.082	0.102	-0.068	0.262	-0.358	-0.598	-0.668	-0.368	-0.298	0.479	0.038	-0.232	-0.445	-0.924	0.527
PF3D7_142300(	PF3D7_1423000::nucleolar GTP-binding protein 2, putative	0.262	0.622	0.432	0.102	0.152	0.062	-0.158	0.132	-0.278	-0.578	-0.378	-0.288	-0.078	0.354	0.018	-0.242	-0.248	-0.603	0.659
PF3D7_030720(	PF3D7_0307200::60S ribosomal protein L7, putative	0.251	0.001	0.361	0.521	0.321	0.481	0.031	0.081	-0.199	-0.609	-0.519	-0.309	-0.409	0.283	0.277	-0.243	-0.413	-0.696	0.617
PF3D7_050790(	PF3D7_0507900::conserved Plasmodium protein, unknown function	0.365	0.275	0.405	0.405	0.425	0.475	0.125	-0.025	-0.225	-0.695	-0.665	-0.315	-0.555	0.363	0.342	-0.315	-0.511	-0.874	0.546
PF3D7_082440(	PF3D7_0824400::nucleoside transporter 2	0.130	0.100	0.320	0.380	0.310	0.490	0.140	0.170	-0.200	-0.880	-0.650	-0.010	-0.300	0.233	0.313	-0.303	-0.320	-0.553	0.682
PF3D7_080480(	PF3D7_0804800::peptidyl-prolyl cis-trans isomerase	0.112	0.332	0.492	0.652	0.352	0.492	0.082	0.062	-0.158	-0.788	-0.858	-0.198	-0.578	0.397	0.309	-0.294	-0.544	-0.942	0.521
PF3D7_132050(	PF3D7_1320500::SNARE protein, putative	0.062	0.242	0.372	0.572	0.242	0.352	0.142	0.322	0.002	-0.898	-0.658	-0.158	-0.588	0.312	0.245	-0.192	-0.468	-0.780	0.582
PF3D7_133340(	PF3D7_1333400::AMMECR1 domain-containing protein, putative	0.246	0.076	0.376	0.656	0.296	0.256	0.216	0.086	0.026	-1.134	-0.474	-0.154	-0.474	0.339	0.256	-0.341	-0.367	-0.706	0.613
PF3D7_051060(	PF3D7_0510600::tRNA Leucine	0.642	0.242	0.282	0.882	0.542	0.192	0.002	-0.078	-0.498	-1.068	-0.358	-0.278	-0.508	0.512	0.246	-0.548	-0.381	-0.893	0.538
PF3D7_050380(	PF3D7_0503800::60S ribosomal protein L31	0.495	0.645	0.495	0.575	0.455	0.205	-0.015	0.075	-0.235	-1.235	-0.585	-0.525	-0.355	0.553	0.215	-0.465	-0.488	-1.041	0.486
PF3D7_114400(	PF3D7_1144000::40S ribosomal protein S21	0.685	0.775	0.405	0.725	0.645	0.695	0.095	-0.025	-0.285	-1.545	-0.975	-0.525	-0.675	0.648	0.479	-0.618	-0.725	-1.373	0.386
PF3D7_081390(	PF3D7_0813900::40S ribosomal protein S16, putative	1.182	0.432	0.432	0.702	0.792	0.492	0.062	0.002	-0.348	-2.068	-0.658	-0.548	-0.468	0.687	0.448	-0.805	-0.558	-1.245	0.422
PF3D7_090390(	PF3D7_0903900::60S ribosomal protein L32	0.894	0.614	0.514	0.424	0.524	0.434	0.024	0.044	-0.296	-1.496	-0.716	-0.526	-0.436	0.611	0.327	-0.583	-0.559	-1.171	0.444
PF3D7_042240(	PF3D7_0422400::40S ribosomal protein S19	0.582	0.492	0.392	0.362	0.662	0.482	-0.008	0.022	-0.288	-1.558	-0.548	-0.258	-0.338	0.457	0.379	-0.608	-0.381	-0.838	0.559
PF3D7_131180(	PF3D7_1311800::M1-family alanyl aminopeptidase	0.472	0.342	0.202	0.262	0.492	0.392	0.032	0.332	-0.238	-1.248	-0.488	-0.278	-0.268	0.319	0.305	-0.385	-0.345	-0.664	0.631
PF3D7_102560(	PF3D7_1025600::cytochrome c oxidase copper chaperone, putative	0.229	0.479	0.339	0.409	0.619	0.229	0.079	0.159	-0.181	-1.461	-0.401	-0.111	-0.391	0.364	0.309	-0.494	-0.301	-0.665	0.631
PF3D7_101470(	PF3D7_1014700::prohibitin 2, putative	0.419	0.329	0.579	0.409	0.659	0.849	-0.021	0.069	-0.331	-1.331	-0.511	-0.681	-0.441	0.434	0.496	-0.531	-0.544	-0.978	0.508
PF3D7_140800(	PF3D7_1408000::plasmepsin II	0.458	0.138	0.458	0.488	0.478	0.918	0.158	0.218	-0.352	-1.422	-0.492	-0.602	-0.452	0.386	0.518	-0.518	-0.515	-0.901	0.536
PF3D7_021400(	PF3D7_0214000::T-complex protein 1 subunit theta	0.532	0.482	0.772	0.502	0.602	0.552	0.032	0.212	-0.418	-1.108	-0.888	-0.768	-0.498	0.572	0.395	-0.438	-0.718	-1.290	0.409
PF3D7_120860(	PF3D7_1208600::mitochondrial import inner membrane translocase subu	0.479	0.469	0.399	0.369	0.339	0.269	0.029	0.079	-0.251	-0.711	-0.571	-0.491	-0.411	0.429	0.213	-0.294	-0.491	-0.920	0.529
PF3D7_100350(	PF3D7_1003500::40S ribosomal protein S20e, putative	0.311	0.431	0.321	0.521	0.621	0.491	-0.009	0.141	-0.279	-0.819	-0.669	-0.709	-0.349	0.396	0.367	-0.319	-0.576	-0.972	0.510
PF3D7_134750(	PF3D7_1347500::DNA/RNA-binding protein Alba 4	0.519	0.459	0.319	0.129	0.389	0.299	-0.111	-0.041	-0.101	-0.641	-0.381	-0.511	-0.331	0.357	0.193	-0.261	-0.407	-0.764	0.589
PF3D7_143480(	PF3D7_1434800::mitochondrial acidic protein MAM33, putative	0.761	0.531	0.531	0.311	0.621	0.791	-0.059	0.141	-0.259	-1.309	-0.849	-0.869	-0.339	0.533	0.451	-0.476	-0.686	-1.219	0.430
PF3D7_112600(	PF3D7_1126000::threonine--tRNA ligase	0.152	0.222	0.162	0.132	0.192	0.242	0.022	0.072	-0.208	-0.468	-0.258	-0.228	-0.038	0.167	0.152	-0.201	-0.174	-0.342	0.789
PF3D7_145620(	PF3D7_1456200::conserved Plasmodium protein, unknown function	0.222	0.472	0.342	0.442	0.252	0.422	0.012	0.202	-0.538	-0.608	-0.558	-0.448	-0.218	0.370	0.229	-0.314	-0.408	-0.778	0.583
PF3D7_072360(	PF3D7_0723600::proteasome assembly chaperone 4, putative	0.107	0.627	0.407	0.547	0.477	0.187	-0.133	-0.033	-0.413	-0.553	-0.443	-0.413	-0.363	0.422	0.177	-0.333	-0.406	-0.828	0.563
PF3D7_110310(	PF3D7_1103100::60S acidic ribosomal protein P1, putative	0.392	0.392	0.632	0.602	0.652	0.162	-0.228	0.012	-0.218	-1.048	-0.658	-0.248	-0.448	0.505	0.196	-0.418	-0.451	-0.956	0.516
PF3D7_133420(	PF3D7_1334200::chaperone binding protein, putative	0.387	0.387	0.527	0.497	0.687	0.457	-0.313	-0.003	-0.403	-0.643	-0.963	-0.233	-0.383	0.449	0.277	-0.350	-0.526	-0.976	0.508
PF3D7_070560(	PF3D7_0705600::RNA helicase, putative	0.589	0.499	0.539	0.119	0.149	0.249	0.189	0.069	-0.391	-0.451	-0.851	-0.321	-0.391	0.437	0.196	-0.257	-0.521	-0.958	0.515
PF3D7_081250(	PF3D7_0812500::RNA-binding protein, putative	0.502	0.502	0.312	0.092	0.022	0.332	0.242	-0.028	-0.218	-0.278	-0.838	-0.328	-0.308	0.352	0.198	-0.175	-0.492	-0.843	0.557
PF3D7_112170(	PF3D7_1121700::protein GCN20	0.348	0.218	0.308	0.108	0.038	0.378	0.158	-0.042	-0.262	-0.232	-0.452	-0.322	-0.242	0.245	0.191	-0.179	-0.339	-0.584	0.667
PF3D7_140790(	PF3D7_1407900::plasmepsin I	0.685	0.355	0.265	0.225	0.075	0.185	0.215	0.205	-0.285	-0.505	-0.725	-0.455	-0.245	0.383	0.159	-0.195	-0.475	-0.858	0.552
PF3D7_062180(	PF3D7_0621800::nascent polypeptide-associated complex subunit alpha	0.728	0.768	0.388	0.238	0.528	0.598	-0.052	0.028	-0.362	-0.672	-0.902	-0.602	-0.692	0.531	0.358	-0.335	-0.732	-1.263	0.417
PF3D7_102780(	PF3D7_1027800::60S ribosomal protein L3	0.304	0.274	0.204	0.114	0.184	0.364	0.054	0.044	-0.046	-0.326	-0.486	-0.326	-0.356	0.224	0.201	-0.109	-0.389	-0.613	0.654
PF3D7_101060(	PF3D7_1010600::eukaryotic translation initiation factor 2 subunit beta	0.945	0.375	0.605	0.225	0.225	0.785	0.205	-0.055	-0.125	-0.805	-1.105	-0.505	-0.775	0.538	0.405	-0.328	-0.795	-1.333	0.397
PF3D7_091430(	PF3D7_0914300::met-10+ like protein, putative	0.481	0.431	0.241	0.281	0.091	0.541	-0.109	0.051	-0.249	-0.549	-0.569	-0.359	-0.279	0.358	0.174	-0.249	-0.403	-0.761	0.590
PF3D7_134720(	PF3D7_1347200::nucleoside transporter 1	0.455	0.565	0.355	0.385	0.485	0.865	-0.015	0.205	-0.365	-0.835	-1.155	-0.655	-0.285	0.440	0.445	-0.332	-0.699	-1.138	0.454
PF3D7_060700(	PF3D7_0607000::translation initiation factor IF-2, putative	0.370	0.350	0.460	0.090	0.090	0.420	0.220	0.090	-0.210	-0.440	-0.810	-0.230	-0.400	0.318	0.243	-0.187	-0.480	-0.798	0.575
PF3D7_062280(	PF3D7_0622800::leucine--tRNA ligase, putative	0.299	0.369	0.309	0.119	0.179	0.459	0.269	0.169	-0.231	-0.431	-0.931	-0.261	-0.321	0.274	0.303	-0.164	-0.504	-0.778	0.583
PF3D7_090340(	PF3D7_0903400::ATP-dependent RNA helicase DDX60, putative	0.254	0.224	0.234	0.104	0.094	0.354	0.084	0.164	-0.236	-0.286	-0.516	-0.306	-0.166	0.204	0.177	-0.119	-0.329	-0.533	0.691
PF3D7_120620(	PF3D7_1206200::eukaryotic translation initiation factor 3 subunit C, puta	0.346	0.426	0.476	0.186	0.176	0.346	0.126	0.186	-0.244	-0.414	-0.994	-0.344	-0.274	0.359	0.216	-0.157	-0.537	-0.896	0.537
PF3D7_130820(	PF3D7_1308200::carbamoyl phosphate synthetase	0.374	0.424	0.354	0.274	0.244	0.344	0.194	0.184	-0.386	-0.396	-0.846	-0.436	-0.326	0.356	0.261	-0.199	-0.536	-0.893	0.539
PF3D7_081360(	PF3D7_0813600::translation initiation factor SUI1, putative	0.500	0.300	0.710	0.360	0.370	0.640	0.350	0.170	-0.350	-0.800	-1.150	-0.450	-0.650	0.468	0.453	-0.327	-0.750	-1.218	0.430
PF3D7_081660(	PF3D7_0816600::chaperone protein ClpB1	0.303	0.173	0.533	0.333	0.403	0.513	0.163	0.223	-0.317	-0.447	-1.027	-0.527	-0.327	0.336	0.360	-0.180	-0.627	-0.963	0.513
PF3D7_144700(	PF3D7_1447000::40S ribosomal protein S5	0.310	0.140	0.380	0.250	0.290	0.470	0.090	0.170	-0.160	-0.430	-0.760	-0.420	-0.330	0.270	0.283	-0.140	-0.503	-0.773	0.585
PF3D7_146190(	PF3D7_1461900::valine--tRNA ligase, putative	0.408	0.158	0.658	0.298	0.318	0.388	0.118	0.168	-0.422	-0.592	-0.902	-0.362	-0.232	0.380	0.274	-0.282	-0.499	-0.879	0.544
PF3D7_071680(	PF3D7_0716800::eukaryotic translation initiation factor 3 subunit I, putat	0.424	0.324	0.234	0.284	0.524	0.514	0.104	0.114	-0.326	-0.356	-1.006	-0.336	-0.496	0.316	0.381	-0.189	-0.613	-0.929	0.525
PF3D7_121380(	PF3D7_1213800::proline--tRNA ligase	0.553	0.333	0.253	0.143	0.243	0.703	0.273	0.073	-0.247	-0.607	-0.967	-0.447	-0.307	0.321	0.406	-0.260	-0.574	-0.894	0.538
PF3D7_132780(	PF3D7_1327800::ribose-phosphate pyrophosphokinase, putative	0.396	0.226	0.086	0.196	0.176	0.466	0.136	-0.004	-0.184	-0.324	-0.674	-0.194	-0.304	0.226	0.259	-0.171	-0.391	-0.617	0.652
PF3D7_010290(	PF3D7_0102900::aspartate--tRNA ligase	0.252	0.452	0.512	0.242	0.222	0.102	0.162	0.242	-0.238	-0.638	-0.828	-0.228	-0.248	0.364	0.162	-0.212	-0.435	-0.799	0.575
PF3D7_062750(	PF3D7_0627500::protein DJ-1	0.526	0.436	0.486	0.546	0.786	0.566	-0.074	0.356	-0.244	-1.444	-0.954	-0.774	-0.214	0.499	0.426	-0.444	-0.647	-1.146	0.452
PF3D7_101590(	PF3D7_1015900::enolase	0.564	0.444	0.794	0.454	0.934	0.584	0.084	0.454	-0.366	-1.646	-1.396	-0.616	-0.286	0.564	0.534	-0.519	-0.766	-1.330	0.398
PF3D7_124910(	PF3D7_1249100::conserved protein, unknown function	0.252	0.052	0.472	0.392	0.522	0.412	0.182	0.172	-0.328	-0.758	-0.698	-0.418	-0.248	0.292	0.372	-0.305	-0.455	-0.747	0.596
PF3D7_032200(	PF3D7_0322000::peptidyl-prolyl cis-trans isomerase	0.298	0.328	0.588	0.438	0.538	0.398	-0.022	0.088	-0.272	-0.772	-0.932	-0.302	-0.372	0.413	0.304	-0.319	-0.536	-0.948	0.518
PF3D7_050710(	PF3D7_0507100::60S ribosomal protein L4	0.394	0.304	0.684	0.354	0.364	0.414	0.074	0.144	-0.176	-0.726	-1.086	-0.346	-0.396	0.434	0.284	-0.253	-0.609	-1.043	0.485
PF3D7_140860(	PF3D7_1408600::40S ribosomal protein S8e, putative	0.486	0.286	0.876	0.476	0.626	0.456	0.096	0.276	-0.244	-1.354	-1.204	-0.234	-0.544	0.531	0.393	-0.441	-0.661	-1.192	0.438
PF3D7_052440(	PF3D7_0524400::ribosome-interacting GTPase 1, putative	0.189	0.199	0.349	0.179	0.229	0.209	0.159	0.169	-0.181	-0.581	-0.591	-0.251	-0.081	0.229	0.199	-0.197	-0.307	-0.537	0.689
PF3D7_133290(	PF3D7_1332900::isoleucine--tRNA ligase, putative	0.237	0.287	0.447	0.267	0.417	0.487	0.127	0.227	-0.303	-0.723	-1.073	-0.233	-0.163	0.309	0.344	-0.266	-0.490	-0.799	0.575
PF3D7_135010(	PF3D7_1350100::lysine--tRNA ligase	0.545	0.195	0.585	0.235	0.385	0.585	0.115	0.235	-0.225	-1.025	-1.155	-0.255	-0.225	0.390	0.362	-0.338	-0.545	-0.935	0.523
PF3D7_051700(	PF3D7_0517000::60S ribosomal protein L12, putative	0.462	0.572	0.412	0.432	0.302	0.222	0.052	0.322	-0.058	-0.818	-0.948	-0.478	-0.468	0.469	0.192	-0.185	-0.632	-1.101	0.466
PF3D7_132340(	PF3D7_1323400::60S ribosomal protein L23	0.495	0.735	0.455	0.505	0.345	0.365	0.105	0.205	-0.205	-0.885	-1.065	-0.415	-0.635	0.547	0.271	-0.295	-0.705	-1.253	0.420
PF3D7_114250(	PF3D7_1142500::60S ribosomal protein L28	0.258	0.528	0.338	0.518	0.318	0.348	0.038	0.198	0.028	-0.632	-1.002	-0.542	-0.392	0.410	0.234	-0.136	-0.646	-1.056	0.481
PF3D7_021780(	PF3D7_0217800::40S ribosomal protein S26	0.429	0.519	0.409	0.779	0.589	0.369	-0.081	0.089	-0.101	-1.111	-0.981	-0.491	-0.421	0.534	0.293	-0.374	-0.631	-1.165	0.446
PF3D7_130280(	PF3D7_1302800::40S ribosomal protein S7, putative	0.308	0.718	0.478	0.698	0.618	0.358	0.038	0.278	-0.342	-1.092	-1.042	-0.422	-0.592	0.550	0.338	-0.386	-0.686	-1.236	0.425
PF3D7_114430(	PF3D7_1144300::60S ribosomal protein L41	0.339	0.709	0.559	0.689	0.699	0.369	-0.071	0.159	-0.091	-1.051	-1.051	-0.601	-0.661	0.574	0.333	-0.327	-0.771	-1.345	0.394
PF3D7_140250(	PF3D7_1402500::ribosomal protein S27a, putative	0.635	0.785	0.595	0.735	0.705	0.485	0.035	0.235	-0.235	-1.165	-1.215	-0.835	-0.755	0.687	0.408	-0.389	-0.935	-1.623	0.325
PF3D7_130830(	PF3D7_1308300::40S ribosomal protein S27	0.199	0.809	0.519	0.549	0.629	0.579	-0.021	0.129	0.039	-1.111	-0.981	-0.671	-0.671	0.519	0.396	-0.314	-0.774	-1.293	0.408
PF3D7_112010(	PF3D7_1120100::phosphoglycerate mutase, putative	0.158	0.418	0.508	0.328	0.598	0.328	-0.002	0.238	-0.092	-0.672	-1.092	-0.492	-0.232	0.353	0.308	-0.175	-0.605	-0.958	0.515
PF3D7_082670(	PF3D7_0826700::receptor for activated c kinase	0.164	0.544	0.464	0.564	0.564	0.554	0.054	0.204	-0.146	-0.866	-1.126	-0.436	-0.536	0.434	0.391	-0.269	-0.699	-1.133	0.456
PF3D7_142120(	PF3D7_1421200::40S ribosomal protein S25	0.398	0.308	0.388	0.468	0.568	0.428	0.178	0.238	-0.232	-0.812	-1.042	-0.472	-0.422	0.391	0.392	-0.268	-0.645	-1.036	0.488

PF3D7_031960(	PF3D7_0319600::elongation factor 1-delta, putative	0.247	0.497	0.617	0.507	0.387	0.457	-0.023	0.237	-0.123	-0.533	-1.293	-0.503	-0.473	0.467	0.274	-0.140	-0.756	-1.223	0.428
PF3D7_051770(	PF3D7_0517700::eukaryotic translation initiation factor 3 subunit B, puta	0.205	0.315	0.465	0.325	0.255	0.385	-0.005	0.185	-0.245	-0.455	-0.745	-0.405	-0.275	0.327	0.211	-0.172	-0.475	-0.803	0.573
PF3D7_113220(	PF3D7_1132200::T-complex protein 1 subunit alpha	0.324	0.374	0.654	0.334	0.354	0.404	0.034	0.214	-0.276	-0.406	-0.976	-0.546	-0.486	0.421	0.264	-0.156	-0.669	-1.091	0.469
PF3D7_060870(	PF3D7_0608700::T-complex protein 1 subunit zeta	0.279	0.559	0.699	0.269	0.369	0.289	-0.061	0.169	-0.291	-0.571	-0.851	-0.441	-0.421	0.452	0.199	-0.231	-0.571	-1.023	0.492
PF3D7_122950(	PF3D7_1229500::T-complex protein 1 subunit gamma	0.263	0.303	0.773	0.363	0.363	0.233	0.043	0.183	-0.227	-0.707	-0.757	-0.387	-0.447	0.426	0.213	-0.250	-0.530	-0.956	0.516
PF3D7_030690(	PF3D7_0306900::40S ribosomal protein S23, putative	0.158	0.418	0.638	0.348	0.388	0.138	-0.182	0.188	-0.122	-0.362	-0.802	-0.372	-0.442	0.391	0.115	-0.098	-0.538	-0.929	0.525
PF3D7_135880(	PF3D7_1358800::40S ribosomal protein S15	0.252	0.572	0.802	0.652	0.482	0.122	0.012	0.272	-0.078	-0.658	-1.118	-0.738	-0.568	0.569	0.205	-0.155	-0.808	-1.378	0.385
PF3D7_030630(	PF3D7_0306300::glutaredoxin 1	0.736	0.656	0.626	0.286	0.336	0.456	-0.084	-0.034	-0.144	-0.624	-1.144	-0.534	-0.534	0.576	0.236	-0.267	-0.737	-1.313	0.402
PF3D7_142410(	PF3D7_1424100::60S ribosomal protein L5, putative	0.418	0.548	0.588	0.308	0.258	0.428	-0.072	0.038	-0.112	-0.392	-1.052	-0.522	-0.432	0.465	0.204	-0.156	-0.669	-1.134	0.456
PF3D7_130540(	PF3D7_1305400::AAR2 protein, putative	0.420	0.300	0.480	0.420	0.140	0.390	0.050	0.050	-0.270	-0.470	-0.650	-0.370	-0.490	0.405	0.193	-0.230	-0.503	-0.908	0.533
PF3D7_102680(	PF3D7_1026800::40S ribosomal protein S2	0.237	0.437	0.527	0.447	0.217	0.327	-0.113	0.007	-0.143	-0.473	-0.713	-0.473	-0.283	0.412	0.144	-0.203	-0.490	-0.902	0.535
PF3D7_146590(	PF3D7_1465900::40S ribosomal protein S3	0.555	0.495	0.725	0.615	0.325	0.285	-0.055	0.035	-0.215	-0.745	-0.895	-0.575	-0.555	0.598	0.185	-0.308	-0.675	-1.273	0.414
PF3D7_132310(	PF3D7_1323100::60S ribosomal protein L6, putative	0.111	0.291	0.421	0.361	0.151	0.401	-0.019	0.021	-0.169	-0.179	-0.669	-0.259	-0.459	0.296	0.177	-0.109	-0.463	-0.758	0.591
PF3D7_145970(	PF3D7_1459700::pyridoxal 5'-phosphate synthase, putative	0.288	0.428	0.548	0.568	0.258	0.498	0.018	-0.052	-0.302	-0.302	-0.962	-0.502	-0.492	0.458	0.258	-0.218	-0.652	-1.110	0.463
PF3D7_051280(	PF3D7_0512800::conserved Plasmodium protein, unknown function	0.178	0.158	0.318	0.198	0.228	0.408	0.148	0.148	-0.152	-0.412	-0.482	-0.342	-0.392	0.213	0.261	-0.139	-0.406	-0.618	0.651
PF3D7_112530(	PF3D7_1125300::DNA-directed RNA polymerase	0.142	0.222	0.432	0.112	0.222	0.492	0.182	0.202	-0.128	-0.618	-0.588	-0.288	-0.378	0.227	0.298	-0.182	-0.418	-0.645	0.639
PF3D7_145440(	PF3D7_1454400::aminopeptidase P	0.075	0.075	0.505	0.175	0.385	0.435	-0.035	0.345	-0.205	-0.435	-0.465	-0.385	-0.475	0.208	0.262	-0.098	-0.441	-0.649	0.638
PF3D7_020460(	PF3D7_0204600::5'-3' exonuclease, putative	0.256	0.206	-0.064	-0.014	-0.044	0.646	0.316	0.026	-0.174	-0.164	-0.404	-0.354	-0.234	0.096	0.306	-0.104	-0.331	-0.427	0.744
PF3D7_121730(	PF3D7_1217300::GTP-binding protein EngA	0.286	0.166	0.016	-0.024	-0.134	0.456	0.236	0.026	-0.074	-0.044	-0.244	-0.394	-0.274	0.111	0.186	-0.031	-0.304	-0.415	0.750
PF3D7_110360(	PF3D7_1103600::actin-like protein, putative	0.317	0.287	0.047	0.137	-0.013	0.437	0.177	-0.063	-0.123	-0.503	-0.263	-0.223	-0.213	0.197	0.200	-0.230	-0.233	-0.430	0.742
PF3D7_111360(	PF3D7_1113600::conserved Plasmodium protein, unknown function	0.358	0.318	0.078	0.048	-0.202	0.378	0.228	0.038	-0.002	-0.442	-0.232	-0.312	-0.262	0.201	0.135	-0.135	-0.268	-0.469	0.722
PF3D7_090170(	PF3D7_0901700::Plasmodium exported protein (hyp5), unknown functio	0.912	0.382	-0.208	-0.098	-0.048	0.902	0.742	-0.128	-0.198	-0.668	-0.648	-0.548	-0.388	0.247	0.532	-0.332	-0.528	-0.775	0.584
PF3D7_123160(	PF3D7_1231600::pre-mRNA-splicing factor ATP-dependent RNA helicas	0.395	0.165	-0.025	0.015	-0.025	0.715	0.375	-0.045	-0.165	-0.415	-0.435	-0.155	-0.395	0.137	0.355	-0.209	-0.329	-0.466	0.724
PF3D7_140500(	PF3D7_1405000::conserved Plasmodium protein, unknown function	0.377	0.267	-0.123	-0.023	-0.043	0.777	0.267	-0.013	0.067	-0.513	-0.363	-0.333	-0.343	0.124	0.334	-0.153	-0.346	-0.471	0.722
PF3D7_131690(	PF3D7_1316900::conserved protein, unknown function	0.295	0.365	-0.015	-0.105	0.065	0.555	0.535	0.075	-0.115	-0.525	-0.535	-0.315	-0.285	0.135	0.385	-0.188	-0.378	-0.513	0.701
PF3D7_050570(	PF3D7_0505700::conserved Plasmodium membrane protein, unknown fu	0.345	0.045	0.185	0.065	-0.045	0.435	0.265	0.185	-0.055	-0.175	-0.845	-0.105	-0.305	0.160	0.219	-0.015	-0.418	-0.578	0.670
PF3D7_071930(	PF3D7_0719300::actin-related protein ARP6	0.268	0.038	0.438	0.158	-0.002	0.298	0.318	0.138	-0.092	-0.232	-0.782	-0.172	-0.382	0.226	0.205	-0.062	-0.445	-0.671	0.628
PF3D7_060810(	PF3D7_0608100::conserved Plasmodium protein, unknown function	0.105	0.045	0.125	0.125	-0.055	0.655	0.415	0.225	0.035	-0.275	-0.845	-0.105	-0.445	0.100	0.338	-0.005	-0.465	-0.565	0.676
PF3D7_102450(	PF3D7_1024500::conserved Plasmodium protein, unknown function	0.234	0.264	0.074	0.374	-0.146	0.454	0.424	0.094	-0.106	-0.226	-0.786	-0.186	-0.466	0.236	0.244	-0.079	-0.479	-0.716	0.609
PF3D7_080700(	PF3D7_0807000::YEATS domain-containing protein, putative	0.143	0.363	0.163	0.333	0.243	0.603	0.193	0.073	-0.257	-0.197	-0.937	-0.267	-0.457	0.251	0.346	-0.127	-0.554	-0.804	0.573
PF3D7_101700(	PF3D7_1017000::DNA polymerase delta catalytic subunit	0.105	0.235	0.195	0.225	0.045	0.585	0.275	0.115	-0.275	-0.235	-0.715	-0.285	-0.265	0.190	0.301	-0.132	-0.422	-0.612	0.654
PF3D7_135270(	PF3D7_1352700::intron-binding protein aquarius, putative	0.178	0.308	0.108	0.108	0.058	0.468	0.338	0.138	-0.142	-0.292	-0.742	-0.242	-0.292	0.176	0.288	-0.098	-0.425	-0.601	0.659
PF3D7_070930(	PF3D7_0709300::mediator of RNA polymerase II transcription subunit 14	0.024	0.194	0.114	0.044	0.084	0.214	0.154	0.154	-0.046	-0.166	-0.476	-0.106	-0.186	0.094	0.151	-0.019	-0.256	-0.350	0.785
PF3D7_130530(	PF3D7_1305300::translational activator GCN1, putative	0.065	0.265	0.225	0.165	0.165	0.435	0.325	0.295	-0.285	-0.165	-0.855	-0.345	-0.295	0.180	0.309	-0.051	-0.498	-0.678	0.625
PF3D7_122010(	PF3D7_1220100::pre-mRNA-processing factor 17, putative	0.096	0.176	0.276	0.176	0.046	0.406	0.256	0.166	-0.144	-0.304	-0.874	-0.144	-0.134	0.181	0.236	-0.094	-0.384	-0.565	0.676
PF3D7_052020(	PF3D7_0520200::mediator of RNA polymerase II transcription subunit 17	0.144	0.384	0.174	0.004	0.114	0.754	0.344	0.104	-0.376	-0.366	-0.666	-0.126	-0.486	0.176	0.404	-0.213	-0.426	-0.603	0.659
PF3D7_093400(	PF3D7_0934000::histidine--tRNA ligase, putative	0.171	0.291	0.061	0.021	0.121	0.611	0.231	-0.009	0.081	-0.259	-0.739	-0.179	-0.399	0.136	0.321	-0.063	-0.439	-0.575	0.671
PF3D7_134790(	PF3D7_1347900::conserved Plasmodium protein, unknown function	0.182	0.302	0.032	0.032	0.072	0.512	0.142	0.022	-0.088	-0.128	-0.728	-0.148	-0.198	0.137	0.242	-0.065	-0.358	-0.495	0.710
PF3D7_121690(	PF3D7_1216900::DNA-binding chaperone, putative	0.172	0.212	0.192	0.042	0.012	0.652	0.252	0.152	-0.228	-0.048	-0.768	-0.298	-0.338	0.154	0.305	-0.042	-0.468	-0.623	0.650
PF3D7_146750(	PF3D7_1467500::DNA/RNA-binding protein KIN17, putative	0.154	0.364	0.144	0.064	-0.116	0.694	0.414	-0.006	-0.186	-0.086	-0.836	-0.176	-0.426	0.181	0.331	-0.093	-0.479	-0.661	0.633
PF3D7_042250(	PF3D7_0422500::pre-mRNA-splicing helicase BRR2, putative	0.114	0.084	0.124	0.134	0.124	0.334	0.214	0.124	-0.056	-0.576	-0.506	-0.066	-0.046	0.114	0.224	-0.169	-0.206	-0.320	0.801
PF3D7_050830(	PF3D7_0508300::triose phosphate transporter	0.151	0.531	0.321	0.181	0.231	1.041	0.341	0.401	-0.239	-1.139	-1.209	-0.329	-0.279	0.296	0.537	-0.326	-0.606	-0.902	0.535
PF3D7_032210(	PF3D7_0322100::mRNA-capping enzyme subunit beta	0.069	0.019	0.329	0.079	0.119	0.539	0.309	0.259	-0.191	-0.421	-0.631	-0.161	-0.321	0.124	0.323	-0.117	-0.371	-0.495	0.710
PF3D7_122370(	PF3D7_1223700::vacuolar iron transporter	0.235	0.285	0.175	0.175	0.285	0.585	0.335	0.275	-0.035	-0.585	-1.105	-0.355	-0.265	0.217	0.401	-0.115	-0.575	-0.793	0.577
PF3D7_123360(	PF3D7_1233600::asparagine and aspartate rich protein 1	0.070	0.000	0.060	0.060	0.110	0.400	0.240	0.180	0.010	-0.270	-0.590	-0.150	-0.120	0.048	0.250	-0.027	-0.287	-0.334	0.793
PF3D7_141280(	PF3D7_1412800::glycylpeptide N-tetradecanoyltransferase	0.164	0.064	0.214	0.154	0.094	0.504	0.344	0.154	-0.016	-0.506	-0.796	-0.286	-0.086	0.149	0.314	-0.123	-0.389	-0.538	0.689
PF3D7_144390(	PF3D7_1443900::heat shock protein 90, putative	0.157	0.037	0.287	0.327	0.127	0.677	0.337	0.267	-0.093	-0.603	-0.973	-0.233	-0.313	0.202	0.380	-0.143	-0.506	-0.708	0.612
PF3D7_092130(	PF3D7_0921300::RAP protein, putative	0.328	0.128	0.158	0.338	0.168	0.668	0.328	0.098	-0.462	-0.382	-0.832	-0.342	-0.202	0.238	0.388	-0.248	-0.458	-0.697	0.617
PF3D7_133170(	PF3D7_1331700::glutamine--tRNA ligase, putative	0.128	0.018	0.278	0.238	0.058	0.388	0.208	0.078	-0.062	-0.132	-0.702	-0.372	-0.122	0.165	0.218	-0.039	-0.399	-0.564	0.676
PF3D7_135550(	PF3D7_1355500::serine/threonine protein phosphatase 5	0.230	0.180	0.130	0.330	0.110	0.810	0.310	0.200	-0.130	-0.360	-0.880	-0.660	-0.270	0.218	0.410	-0.097	-0.603	-0.821	0.566
PF3D7_103210(	PF3D7_1032100::mRNA-decapping enzyme subunit 1, putative	0.068	0.018	0.108	0.038	0.008	0.118	0.068	0.158	0.048	-0.122	-0.262	-0.132	-0.112	0.058	0.064	0.028	-0.169	-0.227	0.855
PF3D7_145880(	PF3D7_1458800::DNA-directed RNA polymerase III subunit RPC5, putat	0.151	0.141	0.001	0.051	0.111	0.241	0.251	0.281	-0.029	-0.139	-0.479	-0.309	-0.269	0.086	0.201	0.037	-0.353	-0.438	0.738
PF3D7_082200(	PF3D7_0822000::ribosomal protein L4, mitochondrial, putative	0.467	-0.023	0.147	0.087	0.007	0.587	0.307	-0.013	-0.103	-0.403	-0.613	-0.233	-0.213	0.169	0.300	-0.173	-0.353	-0.523	0.696
PF3D7_051950(	PF3D7_0519500::CCR4 domain-containing protein 1, putative	0.252	0.042	0.132	0.052	0.132	0.452	0.252	0.112	-0.158	-0.328	-0.478	-0.228	-0.228	0.119	0.278	-0.125	-0.312	-0.431	0.742
PF3D7_100250(	PF3D7_1002500::conserved protein, unknown function	0.490	0.060	0.160	0.210	0.180	0.700	0.300	0.130	-0.120	-0.500	-0.750	-0.460	-0.400	0.230	0.393	-0.163	-0.537	-0.767	0.588
PF3D7_142270(	PF3D7_1422700::conserved Plasmodium protein, unknown function	0.355	0.025	0.215	0.065	0.015	0.535	0.375	0.215	-0.255	-0.325	-0.685	-0.295	-0.245	0.165	0.309	-0.121	-0.408	-0.573	0.672
PF3D7_110750(	PF3D7_1107500::prefoldin, putative	0.377	0.317	0.207	0.237	0.097	0.627	0.317	0.137	-0.033	-0.483	-0.863	-0.353	-0.583	0.284	0.347	-0.126	-0.600	-0.884	0.542
PF3D7_136370(	PF3D7_1363700::protein AMR1	0.374	0.084	0.094	0.144	0.004	0.534	0.164	0.154	-0.036	-0.276	-0.536	-0.346	-0.356	0.174	0.234	-0.053	-0.413	-0.587	0.666
PF3D7_071620(	PF3D7_0716200::PDCD2 domain-containing protein, putative	0.834	-0.066	0.374	0.524	0.324	0.834	0.374	0.004	-0.256	-0.716	-0.826	-0.646	-0.756	0.416	0.511	-0.323	-0.743	-1.159	0.448
PF3D7_144030(	PF3D7_1440300::porphobilinogen synthase	0.558	-0.032	0.308	0.168	0.338	0.668	0.298	0.018	-0.232	-0.242	-0.872	-0.542	-0.432	0.250	0.434	-0.152	-0.616	-0.866	0.549
PF3D7_052690(	PF3D7_0526900::transmembrane emp24 domain-containing protein, put	0.401	0.081	0.061	0.331	-0.039	0.371	0.101	-0.059	-0.039	-0.319	-0.369	-0.239	-0.279	0.218	0.144	-0.139	-0.296	-0.514	0.700
PF3D7_146330(	PF3D7_1463300::DNA polymerase alpha subunit B, putative	0.455	-0.035	0.055	0.375	0.015	0.505	0.225	0.045	0.125	-0.415	-0.635	-0.335	-0.375	0.212	0.248	-0.082	-0.449	-0.661	0.633
PF3D7_147480(	PF3D7_1474800::proteasome subunit alpha type-1, putative	0.542	-0.028	0.062	0.332	0.182	0.662	0.292	0.092	0.032	-0.668	-0.768	-0.468	-0.268	0.227	0.379	-0.181	-0.501	-0.728	0.604
PF3D7_121390(	PF3D7_1213900::W2 domain-containing protein, putative	0.385	-0.025	0.045	0.075	0.035	0.245	0.095	0.035	-0.105	-0.075	-0.325	-0.195	-0.185	0.120	0.125	-0.049	-0.235	-0.355	0.782
PF3D7_090860(	PF3D7_0908600::ribosomal RNA methyltransferase, putative	0.679	0.089	0.289	-0.021	0.209	0.659	-0.061	0.079	-0.191	-0.341	-0.611	-0.431	-0.351	0.259	0.269	-0.151	-0.464	-0.723	0.606
PF3D7_120830(	PF3D7_1208300::acyl carrier protein, mitochondrial, putative	0.442	0.152	0.352	0.172	0.232	0.512	0.092	0.072	-0.018	-0.288	-0.588	-0.718	-0.408	0.279	0.278	-0.078	-0.572	-0.851	0.554
PF3D7_144590(	PF3D7_1445900::ATP-dependent RNA helicase DDX5, putative	0.525	0.225	0.155	0.145	0.125	0.655	0.125	-0.105	-0.135	-0.355	-0.395	-0.535	-0.425	0.262	0.301	-0.199	-0.452	-0.714	0.610
PF3D7_114770(	PF3D7_1147700::ATP synthase subunit delta, mitochondrial, putative	-0.005	0.175	0.265	0.295	0.045	0.455	0.195	-0.105	-0.065	-0.155	-0.445	-0.255	-0.405	0.183	0.232	-0.108	-0.368	-0.551	0.683
PF3D7_114240(	PF3D7_1142400::coproporphyrinogen-III oxidase	0.023	0.013	0.013	0.023	0.073	0.353	0.283	0.003	-0.107	-0.137	-0.347	-0.207	0.013	0.018	0.236	-0.080	-0.180	-0.198	0.872
PF3D7_136640(	PF3D7_1366400::rhoptry protein RHOP148	0.400	-0.050	-0.020	-0.120	0.130	0.610	0.440	-0.050	-0.230	-0.140	-0.550	-0.210	-0.210	0.053	0.393	-0.140	-0.323	-0.376	0.771
PF3D7_121060(	PF3D7_1210600::kelch protein, putative	0.060	0.240	0.100	0.240	-0.210	0.270	0.380	0.140	-0.150	-0.290	-0.310	-0.330	-0.140	0.160	0.147	-0.100	-0.260	-0.420	0.747
PF3D7_091340(	PF3D7_0913400::conserved Plasmodium protein, unknown function	-0.084	0.166	0.566	0.596	0.366	0.266	-0.144	0.176	0.046	-0.404	-0.564	-0.444	-0.544	0.311	0.163	-0.061	-0.517	-0.828	0.563
PF3D7_100400(	PF3D7_1004000::60S ribosomal protein L13, putative	0.029	-0.021	0.619	0.649	0.239	0.199	-0.261	-0.011	0.049	-0.361	-0.561	-0.211	-0.361	0.319	0.059	-0.107	-0.377	-0.697	0.617
PF3D7_032290(	PF3D7_0322900::40S ribosomal protein S3A, putative	0.064	-0.016	0.274	0.684	0.424	0.364	0.014	0.044	-0.026	-0.386	-0.786	-0.386	-0.266	0.251	0.267	-0.123	-0.479	-0.731	0.603
PF3D7_081560(	PF3D7_0815600::eukaryotic translation initiation factor 3 subunit G, puta	0.073	-0.017	0.703	0.743	0.443	0.693	0.003	0.093	-0.127	-0.667	-1.117	-0.437	-0.387	0.376	0.380	-0.234	-0.647	-1.023	0.492
PF3D7_052610(	PF3D7_0526100::conserved Plasmodium membrane protein, unknown fu	-0.059	0.011	0.441	0.501	0.271	0.211	0.181	0.371	-0.069	-0.459	-0.629	-0.429	-0.339	0.223	0.221	-0.053	-0.466	-0.689	0.620
PF3D7_130960(	PF3D7_1309600::ribosomal RNA methyltransferase, putative	-0.045	-0.105	0.455	0.595	0.265	0.335	0.135	0.215	-0.005	-0.375	-0.535	-0.445	-0.485	0.225	0.245	-0.055	-0.489	-0.713	0.610
PF3D7_136000(	PF3D7_1360000::ATP synthase-associated protein, putative	-0.103	-0.043	0.527	0.617	0.027	0.227	0.137	0.187	0.087	-0.263	-0.653	-0.343	-0.403	0.249	0.130	0.004	-0.466	-0.716	0.609
PF3D7_102340(	PF3D7_1023400::HORMA domain protein, putative	-0.035	0.005	0.045	0.495	0.015	0.405	0.365	0.095	0.145	-0.355	-0.675	-0.105	-0.405	0.128	0.262	-0.038	-0.395	-0.523	0.696
PF3D7_072430(	PF3D7_0724300::ubiquinone biosynthesis O-methyltransferase, putative	0.031	0.031	0.071	0.371	0.151	0.351	0.481	0.271	0.121	-0.689	-0.819	-0.019	-0.349	0.126	0.327	-0.099	-0.396	-0.522	0.697
PF3D7_133330(	PF3D7_1333300::transmembrane emp24 domain-containing protein, put	0.155	0.005	0.175	0.385	0.125	0.515	0.305	0.175	0.145	-0.805	-0.625	-0.195	-0.355	0.180	0.315	-0.162	-0.392	-0.572	0.673
PF3D7_133010(	PF3D7_1330100::conserved Plasmodium protein, unknown function	0.023	0.293	0.043	0.113	0.073	0.273	0.373	0.103	0.113	-0.617	-0.567	-0.057	-0.167	0.118	0.240	-0.134	-0.264	-0.382	0.768
PF3D7_111620(	PF3D7_1116200::pyridoxine biosynthesis protein PDX2	0.064	0.154	0.214	0.174	0.034	0.134	0.204	0.134	0.154	-0.396	-0.496	-0.146	-0.226	0.151	0.124	-0.036	-0.289	-0.441	0.737
PF3D7_144710(	PF3D7_1447100::conserved Plasmodium protein, unknown function	0.237	0.297	0.267	0.307	0.067	0.177	0.047	0.107	0.097	-0.453	-0.603	-0.173	-0.373	0.277	0.097	-0.083	-0.383	-0.660	0.633
PF3D7_145330(	PF3D7_1453300::Yos1-like protein, putative	0.100	0.100	-0.040	0.400	0.110	0.100	0.040	0.190	0.160	-0.320	-0.360	-0.200	-0.280	0.140	0.083	0.010	-0.280	-0.420	0.747
PF3D7_021720(	PF3D7_0217200::conserved Plasmodium protein, unknown function	-0.015	0.385	0.005	0.075	0.165	0.475	0.255	0.075	-0.195	-0.165	-0.405	-0.475	-0.175	0.112	0.298	-0.095	-0.352	-0.464	0.725
PF3D7_053150(	PF3D7_0531500::non-coding RNA	-0.104	0.856	-0.074	0.506	0.206	0.476	0.026	0.266	-0.134	-0.454	-0.804	-0.554	-0.214	0.296	0.236	-0.107	-0.524	-0.820	0.566
PF3D7_091650(	PF3D7_0916500::ubiquitin fusion degradation protein 1	-0.085	0.385	-0.055	0.465	0.155	0.625	0.295	0.185	-0.095	-0.805	-0.515	-0.285	-0.275	0.178	0.359	-0.238	-0.358	-0.536	0.690
PF3D7_145230(	PF3D7_1452300::DER1-like protein	-0.126		0.014	0.324	0.634	1.294	0.394	0.304	-0.116	-1.556		-0.606	-0.556	0.070	0.774	-0.456	-0.581	-0.652	0.637
PF3D7_133180(	PF3D7_1331800::60S ribosomal protein L23, putative	-0.084	0.226	-0.094	0.366	0.476	0.636	0.086	0.216	0.046	-0.504	-0.644	-0.544	-0.184	0.104	0.399	-0.081	-0.457	-0.561	0.678
PF3D7_133020(	PF3D7_1330200::conserved Plasmodium protein, unknown function	-0.096	0.274	-0.086	0.084	0.034	0.544	0.314	-0.076	0.314	-0.306	-0.546	-0.236	-0.216	0.044	0.297	-0.023	-0.333	-0.377	0.770
PF3D7_101120(	PF3D7_1011200::BET1-like protein, putative	0.096	0.276	0.126	0.416	-0.004	0.316	0.076	0.066	-0.184	-0.564	-0.144	-0.204	-0.274	0.229	0.129	-0.227	-0.207	-0.436	0.739
PF3D7_133250(	PF3D7_1332500::SAM-dependent RNA methyltransferase, putative	0.189	0.179	0.159	0.369	0.089	0.349	-0.031	0.099	-0.131	-0.591	0.119	-0.551	-0.251	0.224	0.136	-0.207	-0.227	-0.452	0.731
PF3D7_082600(	PF3D7_0826000::conserved Plasmodium protein, unknown function	0.072	0.062	0.392	0.152	0.172	0.422	0.152	0.142	-0.488	-0.188	-0.178	-0.328	-0.378	0.169	0.248	-0.178	-0.295	-0.464	0.725
PF3D7_111100(	PF3D7_1111000::tRNA m5C-methyltransferase, putative	0.165	0.035	0.315	0.165	0.105	0.375	0.135	0.135	-0.285	-0.395	-0.175	-0.155	-0.425	0.170	0.205	-0.181	-0.251	-0.422	0.747
PF3D7_143450(	PF3D7_1434500::dynein-related AAA-type ATPase, putative	0.221	0.121	0.191	0.011	0.151	0.191	0.101	0.201	-0.249	-0.309	-0.119	-0.209	-0.299	0.136	0.147	-0.119	-0.209	-0.345	0.787
PF3D7_125170(	PF3D7_1251700::tryptophan--tRNA ligase	0.078	0.278	0.228	0.178	0.038	0.558	0.118	0.008	-0.282	-0.262	-0.082	-0.412	-0.452	0.191	0.238	-0.178	-0.315	-0.506	0.704
PF3D7_130490(	PF3D7_1304900::DNA-directed RNA polymerase II subunit RPB11, puta	0.258	0.348	0.198	0.158	0.088	0.398	-0.042	0.048	-0.262	-0.282	-0.122	-0.332	-0.452	0.240	0.148	-0.166	-0.302	-0.543	0.687
PF3D7_143640(	PF3D7_1436400::conserved Plasmodium protein, unknown function	0.188	0.568	0.148	0.048	0.078	0.328	-0.152	0.208	-0.152	-0.402	-0.062	-0.312	-0.482	0.238	0.084	-0.116	-0.286	-0.523	0.696

PF3D7_080380(	PF3D7_0803800::proteasome subunit beta type-4	0.184	0.684	0.094	0.034	-0.026	-0.136	-0.076	-0.066	0.104	-0.426	-0.306	0.034	-0.096	0.249	-0.079	-0.129	-0.123	-0.372	0.773
PF3D7_030650(	PF3D7_0306500::cytochrome c oxidase subunit ApiCOX35, putative	0.196	0.456	0.056	0.016	-0.284	0.106	0.096	-0.064	-0.064	-0.254	-0.124	-0.064	-0.074	0.181	-0.027	-0.127	-0.087	-0.268	0.830
PF3D7_100750(	PF3D7_1007500::conserved Plasmodium protein, unknown function	0.278	0.868	0.118	0.118	0.038	0.138	0.138	-0.112	-0.062	-0.672	-0.312	-0.232	-0.302	0.345	0.104	-0.282	-0.282	-0.628	0.647
PF3D7_111980(	PF3D7_1119800::alternative splicing factor ASF-1, putative	0.342	0.922	0.022	0.082	-0.108	0.242	-0.018	-0.048	-0.088	-0.668	-0.138	-0.278	-0.258	0.342	0.038	-0.268	-0.225	-0.567	0.675
PF3D7_022210(	PF3D7_0222100::Pfmc-2TM Maurer's cleft two transmembrane protein	0.416	2.516	0.346	-0.104	-0.064	-0.394	-0.184	-0.014	-0.644	-0.264	-0.454	-0.594	-0.564	0.794	-0.214	-0.307	-0.537	-1.331	0.398
PF3D7_062150(	PF3D7_0621500::ribonuclease P/MRP protein subunit RPP1, putative	0.219	0.709	0.209	0.129	-0.171	-0.011	-0.151	-0.201	-0.031	0.179	-0.441	-0.341	-0.101	0.317	-0.111	-0.017	-0.294	-0.611	0.655
PF3D7_042040(	PF3D7_0420400::ribosome-recycling factor	0.394	0.934	0.194	0.034	0.014	0.224	-0.036	-0.116	-0.086	-0.106	-0.576	-0.396	-0.476	0.389	0.067	-0.103	-0.483	-0.872	0.547
PF3D7_133820(	PF3D7_1338200::60S ribosomal protein L6, putative	0.292	0.852	0.182	-0.008	0.062	0.192	-0.108	-0.178	-0.138	-0.078	-0.428	-0.418	-0.228	0.330	0.049	-0.131	-0.358	-0.688	0.621
PF3D7_102210(	PF3D7_1022100::methyltransferase, putative	0.238	0.818	0.098	-0.032	-0.142	0.128	-0.052	-0.162	-0.032	0.048	-0.292	-0.352	-0.262	0.280	-0.022	-0.049	-0.302	-0.583	0.668
PF3D7_073130(	PF3D7_0731300::Plasmodium exported protein (PHISTb), unknown func	0.368	0.788	0.228	0.018	0.008	0.128	0.028	0.088	-0.172	-0.262	-0.702	-0.192	-0.322	0.350	0.054	-0.116	-0.406	-0.756	0.592
PF3D7_121630(	PF3D7_1216300::signal recognition particle subunit SRP19	0.180	0.970	0.120	0.060	-0.180	0.060	0.070	0.030	-0.070	-0.250	-0.600	-0.230	-0.160	0.333	-0.017	-0.097	-0.330	-0.663	0.632
PF3D7_072830(	PF3D7_0728300::conserved protein, unknown function	0.125	0.765	0.025	-0.015	-0.215	0.175	-0.005	-0.205	0.175	-0.125	-0.125	-0.275	-0.305	0.225	-0.015	-0.051	-0.235	-0.460	0.727
PF3D7_142200(	PF3D7_1422000::cytochrome c oxidase assembly protein COX14, putati	0.054	0.874	0.114	0.154	-0.036	0.174	-0.076	-0.046	0.244	-0.286	-0.406	-0.516	-0.246	0.299	0.021	-0.029	-0.389	-0.688	0.621
PF3D7_111000(	PF3D7_1110000::conserved Plasmodium protein, unknown function	0.396	0.656	0.136	0.266	0.006	-0.094	0.156	0.136	0.066	-0.204	-0.764	-0.174	-0.584	0.364	0.023	-0.001	-0.507	-0.871	0.547
PF3D7_134980(	PF3D7_1349800::GPN-loop GTPase, putative	0.127	0.357	0.057	-0.013	0.017	-0.073	0.097	0.007	0.047	-0.073	-0.123	-0.193	-0.233	0.132	0.014	-0.006	-0.183	-0.315	0.804
PF3D7_080460(	PF3D7_0804600::tRNA pseudouridine synthase, putative	-0.036	0.484	0.254	0.094	-0.276	0.134	0.144	0.104	-0.056	-0.126	-0.246	-0.186	-0.286	0.199	0.001	-0.026	-0.239	-0.438	0.738
PF3D7_040350(	PF3D7_0403500::ubiquitin specific protease, putative	0.023	0.473	-0.017	0.183	-0.067	0.313	0.083	-0.027	-0.017	-0.147	-0.137	-0.337	-0.327	0.166	0.110	-0.064	-0.267	-0.433	0.741
PF3D7_132030(	PF3D7_1320300::conserved protein, unknown function	-0.118	0.502	-0.148	0.142	-0.068	0.412	0.222	0.002	0.032	-0.088	-0.328	-0.178	-0.378	0.094	0.188	-0.018	-0.295	-0.389	0.764
PF3D7_112740(	PF3D7_1127400::conserved Plasmodium protein, unknown function	-0.002	0.508	-0.042	0.298	-0.062	0.198	0.048	0.078	-0.032	0.058	-0.412	-0.232	-0.412	0.191	0.062	0.035	-0.352	-0.543	0.687
PF3D7_060400(	PF3D7_0604000::conserved Plasmodium protein, unknown function	0.350	0.270	0.000	0.010	0.100	0.200	-0.110	0.000	-0.010	-0.030	-0.070	-0.280	-0.430	0.158	0.063	-0.013	-0.260	-0.418	0.749
PF3D7_031280(	PF3D7_0312800::60S ribosomal protein L26, putative	0.459	0.679	0.019	0.219	-0.141	0.099	-0.011	0.069	-0.031	-0.281	-0.371	-0.421	-0.291	0.344	-0.017	-0.081	-0.361	-0.705	0.613
PF3D7_040930(	PF3D7_0409300::methyltransferase, putative	0.457	0.447	0.127	0.027	-0.103	0.157	-0.043	0.107	0.007	-0.203	-0.293	-0.273	-0.413	0.264	0.004	-0.030	-0.326	-0.591	0.664
PF3D7_060490(	PF3D7_0604900::conserved Plasmodium protein, unknown function	0.602	0.812	0.162	0.222	0.122	-0.078	-0.248	-0.028	0.142	-0.428	-0.298	-0.448	-0.528	0.449	-0.068	-0.105	-0.425	-0.874	0.546
PF3D7_060820(	PF3D7_0608200::conserved Plasmodium protein, unknown function	0.347	0.837	0.267	0.217	-0.063	-0.043	-0.123	-0.073	-0.093	-0.373	-0.103	-0.303	-0.493	0.417	-0.076	-0.180	-0.300	-0.717	0.609
PF3D7_030860(	PF3D7_0308600::pre-mRNA-processing factor 19, putative	1.204	0.774	-0.026	-0.026	0.114	0.624	0.074	-0.186	-0.256	-1.006	-0.386	-0.406	-0.496	0.481	0.271	-0.483	-0.429	-0.911	0.532
PF3D7_112650(	PF3D7_1126500::WD repeat-containing protein, putative	0.642	0.662	0.152	0.062	0.142	0.452	0.072	-0.078	-0.398	-0.598	-0.138	-0.448	-0.518	0.379	0.222	-0.358	-0.368	-0.748	0.596
PF3D7_021080(	PF3D7_0210800::conserved Plasmodium protein, unknown function	0.822	0.722	0.052	0.012	-0.068	0.272	0.002	-0.268	-0.248	-0.308	-0.058	-0.398	-0.538	0.402	0.069	-0.274	-0.331	-0.733	0.602
PF3D7_113570(	PF3D7_1135700::conserved Plasmodium protein, unknown function	0.425	0.525	0.075	0.085	-0.005	0.195	0.015	-0.075	-0.115	-0.215	-0.205	-0.305	-0.405	0.278	0.069	-0.135	-0.305	-0.583	0.668
PF3D7_120980(	PF3D7_1209800::ATP synthase mitochondrial F1 complex assembly fact	0.790	0.980	0.100	0.040	0.090	0.360	-0.130	-0.130	-0.130	-0.500	-0.320	-0.560	-0.590	0.478	0.107	-0.253	-0.490	-0.968	0.511
PF3D7_100190(	PF3D7_1001900::Plasmodium exported protein (hyp16), unknown functi	0.791	0.681	0.061	-0.019	0.271	0.171	-0.169	-0.029	-0.189	-0.489	-0.349	-0.419	-0.309	0.378	0.091	-0.236	-0.359	-0.738	0.600
PF3D7_137220(	PF3D7_1372200::histidine-rich protein III	1.792	0.992	0.742	0.292	-0.438	0.722	0.062	-0.118	-0.528	-1.148	-0.448	-0.878	-1.038	0.954	0.115	-0.598	-0.788	-1.743	0.299
PF3D7_100140(	PF3D7_1001400::exported lipase 1	0.830	0.350	0.040	-0.030	-0.050	-0.010	0.010	0.040	-0.270	-0.230	-0.260	-0.260	-0.160	0.298	-0.017	-0.153	-0.227	-0.524	0.695
PF3D7_092150(	PF3D7_0921500::conserved Plasmodium protein, unknown function	0.326	1.156	-0.014	0.366	0.506	0.506	0.046	0.326	-0.864	-0.804	-0.714	-0.524	-0.314	0.459	0.353	-0.447	-0.517	-0.976	0.508
PF3D7_137190(	PF3D7_1371900::Plasmodium exported protein, unknown function	1.103	1.213	0.193	0.463	0.823	0.623	0.033	0.143	-1.047	-0.497	-0.937	-1.237	-0.877	0.743	0.493	-0.467	-1.017	-1.760	0.295
PF3D7_144790(	PF3D7_1447900::multidrug resistance protein 2	0.432	0.422	0.062	-0.038	0.212	0.242	-0.028	-0.048	-0.298	-0.168	-0.308	-0.258	-0.228	0.220	0.142	-0.171	-0.264	-0.484	0.715
PF3D7_135900(	PF3D7_1359000::conserved Plasmodium protein, unknown function	0.202	0.322	0.042	0.122	0.112	-0.028	-0.078	0.112	-0.138	-0.168	-0.068	-0.288	-0.148	0.172	0.002	-0.064	-0.168	-0.340	0.790
PF3D7_060480(	PF3D7_0604800::RAP protein, putative	0.265	0.855	0.255	-0.045	0.415	0.295	0.205	-0.075	-0.265	-0.245	-0.425	-0.535	-0.695	0.332	0.305	-0.195	-0.552	-0.884	0.542
PF3D7_114860(	PF3D7_1148600::18S ribosomal RNA	0.656	1.866	0.326	-0.024	1.046	1.546	-0.174	-0.434	-0.054	-1.314	-0.844	-1.164	-1.434	0.706	0.806	-0.601	-1.147	-1.853	0.277
PF3D7_147890(	PF3D7_1478900::non-coding RNA	1.178	1.638	-0.432	-0.562	0.568	1.218	0.898	0.008	0.388	-0.852	-0.862	-1.392	-1.792	0.455	0.894	-0.152	-1.349	-1.804	0.286
PF3D7_052560(	PF3D7_0525600::RNA methyltransferase, putative	0.172	0.522	-0.018	0.062	-0.128	0.682	0.092	-0.128	-0.188	0.142	-0.218	-0.658	-0.328	0.184	0.215	-0.058	-0.402	-0.586	0.666
PF3D7_072230(	PF3D7_0722300::ubiquitin carboxyl-terminal hydrolase, putative	0.191	0.241	0.081	-0.009	-0.029	0.301	0.101	0.011	-0.199	0.171	-0.169	-0.399	-0.289	0.126	0.124	-0.006	-0.286	-0.412	0.752
PF3D7_082360(	PF3D7_0823600::lipoate-protein ligase B	0.814	1.234	0.114	-0.216	0.114	1.094	0.384	-0.066	-0.306	0.104	-0.966	-1.356	-0.946	0.486	0.531	-0.089	-1.089	-1.576	0.335
PF3D7_061070(	PF3D7_0610700::conserved Plasmodium protein, unknown function	0.559	0.609	0.219	0.069	0.219	0.189	-0.041	0.019	-0.221	0.179	-0.221	-1.061	-0.521	0.364	0.123	-0.007	-0.601	-0.965	0.512
PF3D7_102930(	PF3D7_1029300::conserved protein, unknown function	0.038	0.558	0.098	0.198	0.038	0.238	-0.112	0.018	-0.122	0.208	-0.202	-0.522	-0.432	0.223	0.054	0.034	-0.386	-0.608	0.656
PF3D7_051190(	PF3D7_0511900::conserved Plasmodium protein, unknown function	0.171	0.501	-0.049	0.121	0.321	0.321	0.061	-0.089	-0.419	0.411	-0.609	-0.399	-0.339	0.186	0.234	-0.033	-0.449	-0.635	0.644
PF3D7_122150(	PF3D7_1221500::heptatricopeptide repeat-containing protein, putative	0.122	0.602	0.212	0.092	0.302	0.632	0.122	-0.078	-0.648	0.182	-0.928	-0.388	-0.228	0.257	0.352	-0.181	-0.514	-0.772	0.586
PF3D7_072000(	PF3D7_0720000::exosome complex component CSL4, putative	0.414	0.274	0.064	0.004	0.014	0.604	-0.026	-0.206	-0.226	0.254	-0.546	-0.496	-0.126	0.189	0.197	-0.059	-0.389	-0.578	0.670
PF3D7_090200(	PF3D7_0902000::serine/threonine protein kinase, FIKK family	0.442	0.422	0.142	0.152	0.162	0.432	0.152	0.022	-0.568	0.112	-0.518	-0.678	-0.278	0.290	0.249	-0.144	-0.491	-0.781	0.582
PF3D7_070500(	PF3D7_0705000::mRNA cap guanine-N7 methyltransferase, putative	0.348	0.198	0.218	0.058	0.028	0.448	0.068	0.088	-0.182	0.018	-0.662	-0.412	-0.212	0.205	0.181	-0.026	-0.429	-0.634	0.644
PF3D7_110480(	PF3D7_1104800::metabolite/drug transporter, putative	0.235	0.155	0.125	-0.005	0.145	0.375	0.055	0.195	-0.215	0.205	-0.575	-0.485	-0.215	0.128	0.192	0.062	-0.425	-0.553	0.682
PF3D7_082370(	PF3D7_0823700::mitochondrial import receptor subunit TOM7, putative	0.520	0.560	0.030	0.010	0.070	0.250	-0.130	0.010	-0.050	0.110	-0.530	-0.390	-0.460	0.280	0.063	0.023	-0.460	-0.740	0.599
PF3D7_101310(	PF3D7_1013100::U3 small nucleolar RNA-associated protein 13, putative	0.601	0.671	0.131	0.011	-0.069	0.411	0.131	-0.009	-0.359	-0.129	-0.669	-0.419	-0.299	0.353	0.157	-0.166	-0.463	-0.816	0.568
PF3D7_121720(	PF3D7_1217200::multiple RNA-binding domain-containing protein 1, puta	0.715	0.535	0.185	-0.035	-0.105	0.415	0.025	0.055	-0.305	-0.005	-0.555	-0.445	-0.485	0.350	0.112	-0.085	-0.495	-0.845	0.557
PF3D7_144510(	PF3D7_1445100::histidine--tRNA ligase, putative	0.387	0.487	0.127	0.177	-0.043	0.277	0.117	-0.053	-0.223	0.017	-0.713	-0.323	-0.233	0.294	0.117	-0.086	-0.423	-0.718	0.608
PF3D7_051490(	PF3D7_0514900::conserved Plasmodium protein, unknown function	-0.028	0.562	-0.108	0.202	-0.288	0.442	0.372	0.152	0.212	0.042	-0.858	-0.298	-0.408	0.157	0.176	0.136	-0.521	-0.678	0.625
PF3D7_082820(	PF3D7_0828200::leucine--tRNA ligase, putative	0.000	0.360	0.150	0.060	-0.150	0.430	0.230	0.150	-0.010	-0.150	-0.610	-0.160	-0.300	0.143	0.170	-0.003	-0.357	-0.499	0.708
PF3D7_136920(	PF3D7_1369200::conserved Plasmodium protein, unknown function	0.034	0.364	-0.036	-0.006	-0.096	0.434	0.414	0.134	0.074	-0.316	-0.596	-0.146	-0.256	0.089	0.251	-0.036	-0.333	-0.422	0.747
PF3D7_061000(	PF3D7_0610000::ribosomal protein L19, mitochondrial, putative	0.240	0.510	0.300	0.060	-0.090	0.170	0.430	0.070	-0.060	-0.340	-0.730	-0.270	-0.290	0.278	0.170	-0.110	-0.430	-0.708	0.612
PF3D7_062980(	PF3D7_0629800::cullin-2, putative	0.132	0.322	0.062	0.012	-0.018	0.102	0.152	0.072	0.072	-0.108	-0.538	-0.128	-0.128	0.132	0.078	0.012	-0.265	-0.397	0.760
PF3D7_135940(	PF3D7_1359400::CUGBP Elav-like family member 1	0.222	0.582	0.072	-0.028	-0.018	0.312	0.332	0.052	0.052	-0.118	-0.798	-0.228	-0.438	0.212	0.209	-0.004	-0.488	-0.700	0.616
PF3D7_072960(	PF3D7_0729600::COX assembly mitochondrial protein, putative	0.436	0.606	0.166	0.046	-0.164	0.286	0.206	-0.034	0.056	-0.344	-0.724	-0.184	-0.354	0.314	0.109	-0.107	-0.421	-0.734	0.601
PF3D7_141000(	PF3D7_1410000::ER membrane protein complex subunit 2, putative	0.355	0.565	0.165	0.155	-0.175	0.485	0.255	0.105	0.055	-0.525	-0.715	-0.225	-0.495	0.310	0.188	-0.122	-0.479	-0.788	0.579
PF3D7_060320(	PF3D7_0603200::mitochondrial chaperone BCS1, putative	0.202	0.532	0.292	0.172	0.212	0.412	0.012	-0.068	-0.238	-0.048	-0.828	-0.328	-0.328	0.300	0.212	-0.118	-0.494	-0.794	0.577
PF3D7_082700(	PF3D7_0827000::ATP-dependent RNA helicase DBP10, putative	0.392	0.372	0.372	0.152	0.162	0.352	0.082	0.052	-0.308	-0.108	-0.658	-0.508	-0.348	0.322	0.198	-0.122	-0.505	-0.827	0.564
PF3D7_131270(	PF3D7_1312700::conserved Plasmodium protein, unknown function	0.336	0.696	0.716	0.246	0.106	0.436	0.126	0.186	-0.464	-0.154	-1.084	-0.614	-0.534	0.499	0.223	-0.144	-0.744	-1.243	0.423
PF3D7_021630(	PF3D7_0216300::conserved Plasmodium protein, unknown function	0.305	0.725	0.115	0.115	0.235	0.395	0.235	-0.005	-0.295	-0.105	-0.695	-0.485	-0.545	0.315	0.289	-0.135	-0.575	-0.890	0.540
PF3D7_070640(	PF3D7_0706400::60S ribosomal protein L37	0.308	0.688	0.178	0.128	0.158	0.398	0.058	0.058	-0.242	-0.332	-0.602	-0.462	-0.332	0.325	0.204	-0.172	-0.466	-0.791	0.578
PF3D7_112200(	PF3D7_1122000::conserved protein, unknown function	0.208	0.908	0.388	0.398	0.338	0.758	0.308	0.098	-0.522	-0.532	-0.952	-0.832	-0.572	0.476	0.468	-0.318	-0.785	-1.261	0.417
PF3D7_124670(	PF3D7_1246700::conserved Plasmodium protein, unknown function	0.275	0.985	0.375	0.215	0.085	0.465	0.135	-0.025	-0.395	-0.195	-0.905	-0.505	-0.505	0.462	0.228	-0.205	-0.639	-1.101	0.466
PF3D7_130780(	PF3D7_1307800::FHA domain-containing protein, putative	0.171	0.681	0.431	0.101	0.141	0.361	0.211	-0.029	-0.379	-0.319	-0.719	-0.289	-0.359	0.346	0.237	-0.243	-0.456	-0.802	0.574
PF3D7_071550(	PF3D7_0715500::ATP synthase subunit epsilon, mitochondrial, putative	0.337	0.567	0.247	0.247	-0.023	0.567	0.177	0.027	-0.173	-0.303	-0.873	-0.513	-0.283	0.349	0.240	-0.150	-0.556	-0.906	0.534
PF3D7_090600(	PF3D7_0906000::exoribonuclease II	0.108	0.268	0.258	0.138	0.058	0.248	0.098	0.158	-0.082	-0.222	-0.492	-0.362	-0.182	0.193	0.135	-0.048	-0.345	-0.538	0.689
PF3D7_143800(	PF3D7_1438000::eukaryotic translation initiation factor eIF2A, putative	0.108	0.308	0.278	0.128	0.088	0.318	0.148	0.138	-0.112	-0.202	-0.642	-0.422	-0.142	0.206	0.185	-0.058	-0.402	-0.608	0.656
PF3D7_082170(	PF3D7_0821700::60S ribosomal protein L22, putative	0.182	0.872	0.532	0.252	0.222	0.232	0.142	0.172	-0.088	-0.448	-0.978	-0.568	-0.518	0.459	0.198	-0.122	-0.688	-1.148	0.451
PF3D7_103770(	PF3D7_1037700::enhancer of rudimentary homolog, putative	0.045	0.605	0.295	0.295	0.065	0.245	0.135	0.165	0.085	-0.195	-0.715	-0.345	-0.685	0.310	0.149	0.019	-0.581	-0.892	0.539
PF3D7_052060(	PF3D7_0520600::bis(5'-nucleosyl)-tetraphosphatase [asymmetrical]	0.328	0.528	0.208	0.308	0.208	0.438	0.128	0.148	-0.112	-0.022	-0.782	-0.712	-0.672	0.343	0.258	0.005	-0.722	-1.065	0.478
PF3D7_136040(	PF3D7_1360400::conserved Plasmodium protein, unknown function	0.215	0.615	0.375	0.315	0.225	0.335	0.135	0.115	-0.255	-0.125	-0.835	-0.475	-0.645	0.380	0.232	-0.088	-0.651	-1.032	0.489
PF3D7_142780(	PF3D7_1427800::exosome complex component RRP41	0.069	0.489	0.499	0.289	0.129	0.209	0.289	0.199	-0.061	0.089	-0.851	-0.721	-0.631	0.337	0.209	0.076	-0.734	-1.071	0.476
PF3D7_042020(	PF3D7_0420200::holo-[acyl-carrier-protein] synthase, putative	0.284	0.594	0.214	0.444	0.024	0.574	0.224	0.044	-0.546	-0.356	-0.806	-0.366	-0.326	0.384	0.274	-0.286	-0.499	-0.883	0.542
PF3D7_136450(	PF3D7_1364500::exosome complex component RRP45, putative	0.011	0.371	0.131	0.261	-0.019	0.431	0.071	0.121	-0.219	-0.169	-0.469	-0.299	-0.219	0.193	0.161	-0.089	-0.329	-0.523	0.696
PF3D7_134130(	PF3D7_1341300::60S ribosomal protein L18-2, putative	0.144	0.674	0.134	0.234	-0.186	0.544	0.074	0.124	-0.256	-0.476	-0.446	-0.276	-0.286	0.296	0.144	-0.203	-0.336	-0.633	0.645
PF3D7_062880(	PF3D7_0628800::glutamyl-tRNA(Gln) amidotransferase subunit B	-0.009	0.651	0.231	0.111	0.201	0.451	0.051	0.001	-0.089	-0.189	-0.779	-0.149	-0.479	0.246	0.234	-0.093	-0.469	-0.715	0.609
PF3D7_120180(	PF3D7_1201800::cytochrome c oxidase assembly protein COX19, putati	0.203	0.293	0.093	0.183	0.043	0.223	0.133	0.163	-0.147	-0.277	-0.577	-0.007	-0.327	0.193	0.133	-0.087	-0.304	-0.497	0.709
PF3D7_090691(	PF3D7_0906910::tubulin-specific chaperone, putative	0.234	0.644	0.304	0.114	0.204	0.524	0.154	0.144	-0.236	-0.456	-0.836	-0.236	-0.556	0.324	0.294	-0.183	-0.543	-0.867	0.548
PF3D7_145730(	PF3D7_1457300::MA3 domain-containing protein, putative	0.399	0.679	0.309	0.149	0.119	0.489	0.079	0.209	-0.201	-0.531	-1.001	-0.381	-0.321	0.384	0.229	-0.174	-0.567	-0.952	0.517
PF3D7_131410(	PF3D7_1314100::conserved Plasmodium protein, unknown function	0.202	0.762	0.172	0.212	0.312	0.672	0.242	0.122	-0.398	-0.568	-1.128	-0.338	-0.258	0.337	0.408	-0.282	-0.575	-0.912	0.532
PF3D7_131490(	PF3D7_1314900::general transcription factor IIH subunit 2	0.371	0.881	0.131	0.191	0.161	0.441	0.171	0.061	-0.499	-0.589	-0.789	-0.199	-0.329	0.393	0.257	-0.343	-0.439	-0.833	0.562
PF3D7_052450(	PF3D7_0524500::conserved Plasmodium protein, unknown function	0.122	0.792	0.082	-0.018	-0.158	0.442	0.122	0.082	-0.158	-0.088	-0.558	-0.528	-0.138	0.245	0.136	-0.054	-0.408	-0.653	0.636
PF3D7_070790(	PF3D7_0707900::ribosomal protein S8e, putative	0.140	0.460	0.160	0.140	-0.040	0.420	-0.070	0.130	-0.110	-0.140	-0.370	-0.420	-0.300	0.225	0.103	-0.040	-0.363	-0.588	0.665
PF3D7_110940(	PF3D7_1109400::essential nuclear protein 1, putative	0.533	1.083	0.023	-0.087	-0.057	0.243	0.213	0.103	-0.187	-0.177	-0.637	-0.457	-0.597	0.388	0.133	-0.087	-0.564	-0.952	0.517
PF3D7_093330(	PF3D7_0933300::conserved Plasmodium protein, unknown function	0.126	0.506	0.056	0.066	-0.114	0.266	0.066	0.026	-0.074	-0.034	-0.374	-0.234	-0.284	0.189	0.073	-0.027	-0.297	-0.486	0.714
PF3D7_052870(	PF3D7_0528700::peptidyl-prolyl cis-trans isomerase	0.330	0.810	0.030	0.210	-0.190	0.380	0.360	0.130	-0.030	-0.270	-0.820	-0.430	-0.510	0.345	0.183	-0.057	-0.587	-0.932	0.524
PF3D7_145400(	PF3D7_1454000::RNA-binding protein, putative	0.252	0.522	0.162	0.092	-0.128	0.322	0.132	0.052	-0.068	-0.278	-0.458	-0.298	-0.298	0.257	0.108	-0.098	-0.352	-0.608	0.656
PF3D7_141940(	PF3D7_1419400::conserved Plasmodium membrane protein, unknown fu	0.131	0.381	0.081	0.021	-0.089	0.271	0.201	0.141	-0.149	-0.079	-0.289	-0.269	-0.349	0.153	0.127	-0.029	-0.303	-0.456	0.729
PF3D7_113230(	PF3D7_1132300::nucleic acid binding protein, putative	0.036	0.526	0.016	0.016	-0.054	0.426	0.056	-0.094	0.046	-0.154	-0.424	-0.224	-0.174	0.149	0.143	-0.067	-0.274	-0.423	0.746
PF3D7_122160(	PF3D7_1221600::protein TSSC1, putative	0.151	0.571	0.011	0.051	-0.069	0.581	0.231	-0.019	-0.179	-0.239	-0.679	-0.249	-0.159	0.196	0.247	-0.146	-0.363	-0.558	0.679
PF3D7_103010(	PF3D7_1030100::pre-mRNA-splicing factor ATP-dependent RNA helicase	0.267	0.427	0.087	-0.013	-0.013	0.527	0.087	0.007	-0.093	-0.353	-0.453	-0.353	-0.123	0.192	0.200	-0.146	-0.310	-0.502	0.706
PF3D7_113000(	PF3D7_1130000::phosphoacetylglucosamine mutase, putative	0.478	0.698	0.008	-0.002	0.038	0.558	0.178	-0.042	-0.102	-0.292	-0.722	-0.392	-0.402	0.295	0.258	-0.146	-0.506	-0.801	0.574
PF3D7_133980(	PF3D7_1339800::mitotic-spindle organizing protein 1, putative	0.322	1.062	0.152	0.072	0.062	0.772	0.252	-0.018	-0.338	-0.558	-0.718	-0.498	-0.568	0.402	0.362	-0.304	-0.594	-0.997	0.501

PF3D7_144530(	PF3D7_1445300::ribosomal protein S29, mitochondrial, putative	0.322	0.682	0.032	0.112	-0.068	0.402	-0.038	-0.138	-0.118	-0.218	-0.398	-0.218	-0.348	0.287	0.098	-0.158	-0.322	-0.608	0.656
PF3D7_080310(	PF3D7_0803100::U3 small nucleolar RNA-associated protein 14, putative	0.405	0.205	0.445	0.005	-0.055	0.265	0.165	0.115	-0.345	-0.205	-0.415	-0.285	-0.295	0.265	0.125	-0.145	-0.332	-0.597	0.661
PF3D7_100560(	PF3D7_1005600::DnaJ protein, putative	0.234	0.244	0.404	0.034	-0.046	0.154	0.044	0.084	-0.266	-0.106	-0.326	-0.196	-0.256	0.229	0.051	-0.096	-0.259	-0.488	0.713
PF3D7_072130(	PF3D7_0721300::ATP-dependent DNA helicase DDX31	0.338	0.218	0.448	0.008	0.118	0.328	0.048	0.148	-0.172	-0.152	-0.522	-0.362	-0.452	0.253	0.165	-0.058	-0.445	-0.698	0.616
PF3D7_122650(	PF3D7_1226500::heptatricopeptide repeat and RAP domain-containing	0.169	0.219	0.309	0.059	0.079	0.259	0.179	0.019	-0.191	-0.091	-0.351	-0.291	-0.371	0.189	0.173	-0.087	-0.337	-0.527	0.694
PF3D7_140760(	PF3D7_1407600::conserved Plasmodium protein, unknown function	0.468	0.418	0.428	0.178	-0.142	0.308	0.108	-0.092	-0.342	-0.112	-0.432	-0.152	-0.632	0.373	0.091	-0.182	-0.406	-0.778	0.583
PF3D7_081500(	PF3D7_0815000::selenoprotein	0.272	0.532	0.582	0.392	0.252	0.562	0.332	0.182	-0.318	-0.688	-0.698	-0.778	-0.628	0.445	0.382	-0.274	-0.701	-1.146	0.452
PF3D7_102620(	PF3D7_1026200::conserved protein, unknown function	0.150	0.640	0.450	0.300	0.160	0.350	0.100	0.230	-0.250	-0.370	-0.550	-0.590	-0.620	0.385	0.203	-0.130	-0.587	-0.972	0.510
PF3D7_102040(	PF3D7_1020400::rRNA (cytosine-C(5))-methyltransferase, putative	0.028	0.458	0.378	0.168	0.048	0.128	0.048	0.098	-0.222	-0.242	-0.352	-0.402	-0.142	0.258	0.075	-0.122	-0.298	-0.557	0.680
PF3D7_140210(	PF3D7_1402100::pseudouridine synthase, putative	0.179	0.639	0.529	0.119	0.219	0.329	0.109	0.129	-0.571	-0.351	-0.281	-0.591	-0.461	0.367	0.219	-0.264	-0.444	-0.811	0.570
PF3D7_125000(	PF3D7_1250000::rRNA-processing protein FCF2, putative	0.155	0.405	0.655	0.285	0.185	0.315	0.325	-0.025	-0.305	-0.685	-0.605	-0.335	-0.375	0.375	0.275	-0.338	-0.438	-0.813	0.569
PF3D7_140750(	PF3D7_1407500::multifunctional methyltransferase subunit TRM112, put	0.100	0.650	0.680	0.240	0.070	0.200	0.030	-0.070	-0.190	-0.590	-0.410	-0.290	-0.420	0.418	0.100	-0.283	-0.373	-0.791	0.578
PF3D7_030440(	PF3D7_0304400::60S ribosomal protein L44	-0.045	0.705	0.285	0.175	0.205	0.055	-0.105	-0.095	-0.145	-0.025	-0.365	-0.365	-0.275	0.280	0.051	-0.089	-0.335	-0.615	0.653
PF3D7_092110(	PF3D7_0921100::conserved Plasmodium protein, unknown function	0.125	0.795	0.575	0.335	0.425	0.085	-0.145	-0.045	-0.255	-0.205	-0.315	-0.765	-0.615	0.458	0.122	-0.168	-0.565	-1.023	0.492
PF3D7_113010(	PF3D7_1130100::60S ribosomal protein L38	0.097	0.477	0.277	0.107	0.157	0.277	-0.163	-0.083	0.017	-0.053	-0.463	-0.393	-0.253	0.239	0.090	-0.040	-0.370	-0.609	0.656
PF3D7_021390(	PF3D7_0213900::RTR1 domain-containing protein, putative	0.222	0.332	0.402	0.072	-0.028	-0.048	-0.048	0.122	-0.268	0.102	-0.328	-0.228	-0.308	0.257	-0.041	-0.014	-0.288	-0.545	0.685
PF3D7_072930(	PF3D7_0729300::60S ribosomal export protein NMD3, putative	0.339	0.469	0.489	0.099	0.039	0.059	-0.211	0.029	-0.271	0.109	-0.421	-0.361	-0.371	0.349	-0.037	-0.044	-0.384	-0.733	0.602
PF3D7_052880(	PF3D7_0528800::nuclear GTP-binding protein, putative	0.222	0.812	0.382	0.012	-0.028	-0.108	-0.188	0.022	-0.358	0.232	-0.348	-0.298	-0.358	0.357	-0.108	-0.034	-0.334	-0.692	0.619
PF3D7_071610(	PF3D7_0716100::protein SDA1, putative	0.359	0.789	0.399	-0.011	-0.021	-0.031	-0.071	0.079	-0.261	0.099	-0.431	-0.441	-0.461	0.384	-0.041	-0.027	-0.444	-0.828	0.563
PF3D7_140590(	PF3D7_1405900::RNA-binding protein, putative	0.325	0.495	0.285	0.045	-0.125	0.025	-0.095	0.035	-0.215	0.065	-0.335	-0.235	-0.265	0.287	-0.065	-0.039	-0.279	-0.566	0.676
PF3D7_050610(	PF3D7_0506100::60S ribosomal subunit protein L24, putative	0.165	0.645	0.435	0.215	0.045	0.075	-0.215	0.035	-0.145	0.205	-0.545	-0.445	-0.475	0.365	-0.031	0.032	-0.488	-0.853	0.554
PF3D7_146930(	PF3D7_1469300::pre-rRNA-processing protein PNO1, putative	0.155	0.765	0.425	0.145	-0.085	0.055	-0.215	0.015	-0.265	0.185	-0.495	-0.335	-0.345	0.372	-0.082	-0.022	-0.392	-0.764	0.589
PF3D7_114600(	PF3D7_1146000::ribosome assembly protein 4, putative	0.358	0.678	0.548	0.108	0.148	-0.192	-0.332	0.068	-0.312	-0.192	-0.332	-0.252	-0.302	0.423	-0.125	-0.145	-0.295	-0.718	0.608
PF3D7_102840(	PF3D7_1028400::ribosome biogenesis protein RPF2, putative	0.205	0.395	0.345	0.145	0.055	0.005	0.005	0.095	-0.265	-0.125	-0.435	-0.195	-0.225	0.272	0.021	-0.099	-0.285	-0.558	0.679
PF3D7_112000(	PF3D7_1120000::conserved protein, unknown function	0.202	0.512	0.432	0.162	0.152	0.032	-0.128	0.112	-0.248	-0.128	-0.438	-0.278	-0.388	0.327	0.019	-0.088	-0.368	-0.695	0.618
PF3D7_122600(	PF3D7_1226000::conserved protein, unknown function	0.297	0.577	0.387	0.217	0.087	0.047	-0.143	0.197	-0.213	-0.103	-0.573	-0.413	-0.363	0.369	-0.003	-0.040	-0.450	-0.819	0.567
PF3D7_103820(	PF3D7_1038200::conserved Plasmodium protein, unknown function	0.206	0.946	0.526	0.306	0.216	-0.024	-0.164	-0.134	-0.244	-0.204	-0.634	-0.304	-0.494	0.496	0.009	-0.194	-0.477	-0.973	0.509
PF3D7_092320(	PF3D7_0923200::nitric oxide synthase, putative	0.191	0.711	0.661	0.091	0.261	-0.059	-0.009	-0.039	-0.329	0.031	-0.529	-0.339	-0.639	0.413	0.064	-0.113	-0.503	-0.916	0.530
PF3D7_147870(	PF3D7_1478700::Plasmodium exported protein, unknown function, pseu	0.501	1.361	1.011	0.191	0.461	0.041	-0.209	-0.129	-0.639	-0.239	-0.859	-0.539	-0.949	0.766	0.097	-0.336	-0.783	-1.548	0.342
PF3D7_070080(	PF3D7_0700800::Pfmc-2TM Maurer's cleft two transmembrane protein	0.274	0.844	0.314	0.064	0.174	-0.166	-0.006	0.264	-0.436	-0.036	-0.386	-0.286	-0.616	0.374	0.001	-0.069	-0.429	-0.803	0.573
PF3D7_110080(	PF3D7_1100800::Pfmc-2TM Maurer's cleft two transmembrane protein	0.258	1.018	0.308	0.018	0.188	-0.292	-0.032	0.198	-0.412	0.048	-0.402	-0.432	-0.472	0.401	-0.045	-0.055	-0.435	-0.836	0.560
PF3D7_142140(	PF3D7_1421400::DNA-directed RNA polymerase III subunit RPC6, putat	0.195	1.125	0.445	0.025	0.155	0.085	0.035	0.215	-0.265	-0.135	-0.715	-0.555	-0.605	0.447	0.091	-0.062	-0.625	-1.073	0.475
PF3D7_114940(	PF3D7_1149400::Plasmodium exported protein, unknown function	0.252	1.112	0.402	-0.058	0.062	0.002	0.082	0.152	-0.498	0.372	-0.908	-0.358	-0.608	0.427	0.048	0.008	-0.625	-1.052	0.482
PF3D7_050930(	PF3D7_0509300::conserved Plasmodium protein, unknown function	0.542	0.662	0.722	0.192	0.042	0.152	0.062	0.012	-0.438	-0.368	-0.418	-0.558	-0.598	0.529	0.085	-0.265	-0.525	-1.054	0.482
PF3D7_112390(	PF3D7_1123900::13 kDa ribonucleoprotein-associated protein, putative	0.485	0.765	0.625	0.175	0.195	0.105	0.055	0.045	-0.365	-0.515	-0.545	-0.615	-0.415	0.513	0.119	-0.278	-0.525	-1.038	0.487
PF3D7_112640(	PF3D7_1126400::mediator of RNA polymerase II transcription subunit 21	0.270	0.560	0.340	0.200	0.090	0.170	0.070	0.060	-0.200	-0.180	-0.320	-0.560	-0.500	0.343	0.110	-0.107	-0.460	-0.803	0.573
PF3D7_120710(	PF3D7_1207100::pre-rRNA-processing protein ESF1, putative	0.245	0.345	0.195	0.065	-0.025	0.065	-0.045	0.095	-0.085	-0.215	-0.165	-0.245	-0.235	0.213	-0.001	-0.068	-0.215	-0.428	0.744
PF3D7_092190(	PF3D7_0921900::conserved Plasmodium protein, unknown function	0.335	0.735	0.435	0.085	-0.125	0.125	-0.055	-0.025	-0.225	-0.085	-0.525	-0.335	-0.335	0.397	-0.019	-0.112	-0.399	-0.796	0.576
PF3D7_103330(	PF3D7_1033300::conserved protein, unknown function	0.672	1.512	0.662	0.282	0.122	0.182	-0.128	0.012	-0.448	-0.588	-0.898	-0.698	-0.678	0.782	0.058	-0.342	-0.758	-1.540	0.344
PF3D7_110770(	PF3D7_1107700::pescadillo homolog	0.423	0.893	0.623	0.113	-0.047	0.233	-0.117	-0.017	-0.387	-0.347	-0.537	-0.437	-0.397	0.513	0.023	-0.250	-0.457	-0.970	0.511
PF3D7_083070(	PF3D7_0830700::Plasmodium exported protein (hyp9), unknown functio	0.653	0.983	0.293	0.043	0.313	0.183	-0.007	0.153	-0.557	-0.257	-0.697	-0.537	-0.567	0.493	0.163	-0.220	-0.600	-1.093	0.469
PF3D7_102830(	PF3D7_1028300::rRNA-processing protein EBP2, putative	0.578	0.908	0.428	0.078	0.098	0.168	-0.112	-0.042	-0.382	-0.182	-0.542	-0.412	-0.592	0.498	0.052	-0.202	-0.515	-1.013	0.495
PF3D7_112540(	PF3D7_1125400::mitochondrial import inner membrane translocase subu	0.599	1.029	0.519	0.119	0.489	0.379	-0.091	0.169	-0.441	-0.641	-0.741	-0.771	-0.621	0.567	0.259	-0.304	-0.711	-1.278	0.413
PF3D7_142610(	PF3D7_1426100::transcription factor BTF3, putative	0.554	0.894	0.424	0.264	0.334	0.204	-0.056	0.014	-0.286	-0.576	-0.776	-0.506	-0.486	0.534	0.161	-0.283	-0.589	-1.123	0.459
PF3D7_143400(	PF3D7_1434000::CCR4-associated factor 16, putative	0.569	0.789	0.429	0.249	0.229	0.289	-0.111	-0.011	-0.311	-0.401	-0.771	-0.451	-0.501	0.509	0.136	-0.241	-0.574	-1.083	0.472
PF3D7_071900(	PF3D7_0719000::conserved protein, unknown function	0.175	0.575	0.375	0.005	0.205	0.195	-0.075	0.045	-0.245	-0.165	-0.425	-0.415	-0.245	0.282	0.108	-0.122	-0.362	-0.644	0.640
PF3D7_130710(	PF3D7_1307100::U3 small nucleolar RNA-associated protein 6, putative	0.236	0.506	0.356	0.076	0.086	0.216	-0.124	0.146	-0.404	-0.074	-0.444	-0.384	-0.194	0.294	0.059	-0.111	-0.341	-0.634	0.644
PF3D7_124180(	PF3D7_1241800::ATP-dependent RNA helicase DBP9, putative	0.274	0.484	0.264	0.024	0.024	0.154	0.094	0.114	-0.416	-0.156	-0.356	-0.326	-0.176	0.261	0.091	-0.153	-0.286	-0.548	0.684
PF3D7_082550(	PF3D7_0825500::protein KRI1, putative	0.418	0.658	0.378	0.018	0.028	0.088	0.058	0.008	-0.292	-0.092	-0.482	-0.432	-0.352	0.368	0.058	-0.126	-0.422	-0.790	0.578
PF3D7_100900(	PF3D7_1009000::diphthine methyl ester synthase, putative	0.517	0.847	0.317	0.027	-0.033	0.227	0.007	-0.023	-0.303	0.027	-0.583	-0.593	-0.433	0.427	0.067	-0.100	-0.536	-0.963	0.513
PF3D7_080800(	PF3D7_0808000::conserved Plasmodium protein, unknown function	0.471	0.671	0.241	0.021	0.111	0.321	0.151	-0.019	-0.239	-0.069	-0.609	-0.619	-0.429	0.351	0.194	-0.109	-0.553	-0.903	0.535
PF3D7_144520(	PF3D7_1445200::ATP-dependent RNA helicase MAK5, putative	0.335	0.565	0.295	-0.015	-0.055	0.195	0.205	0.075	-0.245	-0.055	-0.565	-0.415	-0.315	0.295	0.115	-0.075	-0.432	-0.727	0.604
PF3D7_141150(	PF3D7_1411500::conserved Plasmodium protein, unknown function	0.339	0.829	0.239	0.029	0.029	0.169	0.039	0.049	-0.211	0.199	-0.581	-0.601	-0.531	0.359	0.079	0.013	-0.571	-0.930	0.525
PF3D7_063090(	PF3D7_0630900::ATP-dependent RNA helicase HAS1	0.289	0.599	0.479	0.169	-0.051	0.289	-0.071	0.079	-0.321	-0.141	-0.441	-0.451	-0.431	0.384	0.056	-0.127	-0.441	-0.825	0.564
PF3D7_146340(	PF3D7_1463400::DNA-directed RNA polymerase III subunit RPC4, putat	0.333	0.693	0.533	0.113	-0.017	0.233	-0.027	0.153	-0.397	0.163	-0.627	-0.607	-0.547	0.418	0.063	-0.027	-0.594	-1.012	0.496
PF3D7_133960(	PF3D7_1339600::conserved Plasmodium protein, unknown function	0.145	0.635	0.345	0.275	0.135	0.235	-0.045	-0.005	-0.285	0.065	-0.535	-0.655	-0.305	0.350	0.108	-0.075	-0.499	-0.848	0.555
PF3D7_072760(	PF3D7_0727600::conserved Plasmodium protein, unknown function	0.365	0.555	0.395	0.015	0.305	0.355	0.155	0.165	-0.365	-0.215	-0.595	-0.475	-0.655	0.332	0.271	-0.139	-0.575	-0.908	0.533
PF3D7_134030(	PF3D7_1340300::nucleolar complex protein 2, putative	0.358	0.468	0.338	0.018	0.108	0.228	-0.032	0.178	-0.302	-0.132	-0.372	-0.412	-0.452	0.296	0.102	-0.085	-0.412	-0.708	0.612
PF3D7_135770(	PF3D7_1357700::U3 small nucleolar RNA-associated protein 21, putative	0.409	0.519	0.289	0.019	0.129	0.239	0.079	0.159	-0.321	-0.221	-0.471	-0.431	-0.401	0.309	0.149	-0.127	-0.434	-0.743	0.597
PF3D7_120150(	PF3D7_1201500::GPN-loop GTPase, putative	0.462	0.462	0.402	0.072	-0.028	0.252	0.002	0.032	-0.268	-0.058	-0.368	-0.578	-0.388	0.350	0.076	-0.098	-0.444	-0.794	0.577
PF3D7_142950(	PF3D7_1429500::diphthamide biosynthesis protein 2, putative	0.385	0.335	0.285	0.015	0.025	0.245	0.145	0.175	-0.225	-0.025	-0.325	-0.555	-0.485	0.255	0.139	-0.025	-0.455	-0.710	0.611
PF3D7_031030(	PF3D7_0310300::phosphoglycerate mutase, putative	0.289	0.249	0.249	0.139	0.049	0.019	-0.071	0.049	-0.121	-0.001	-0.281	-0.301	-0.271	0.232	-0.001	-0.024	-0.284	-0.516	0.699
PF3D7_131240(	PF3D7_1312400::translation initiation factor IF-2, putative	0.397	0.367	0.347	0.087	0.027	0.117	-0.063	0.187	-0.153	-0.003	-0.543	-0.443	-0.323	0.299	0.027	0.010	-0.436	-0.736	0.600
PF3D7_145420(	PF3D7_1454200::conserved Plasmodium protein, unknown function	0.248	0.338	0.298	0.028	0.188	0.008	-0.092	0.168	-0.212	0.028	-0.392	-0.302	-0.312	0.228	0.035	-0.005	-0.335	-0.563	0.677
PF3D7_122890(	PF3D7_1228900::conserved protein, unknown function	0.364	0.474	0.684	0.244	0.184	0.254	-0.066	0.184	-0.196	-0.096	-0.776	-0.686	-0.566	0.441	0.124	-0.036	-0.676	-1.118	0.461
PF3D7_147060(	PF3D7_1470600::RAP protein, putative	0.210	0.300	0.470	0.210	0.120	0.190	0.000	0.220	-0.230	0.130	-0.620	-0.590	-0.410	0.298	0.103	0.040	-0.540	-0.838	0.560
PF3D7_141190(	PF3D7_1411900::p1/s1 nuclease, putative	0.218	0.348	0.198	0.038	0.178	0.138	-0.202	0.138	-0.212	0.118	-0.252	-0.382	-0.322	0.200	0.038	0.014	-0.319	-0.519	0.698
PF3D7_147750(	PF3D7_1477500::Plasmodium exported protein (PHISTb), unknown func	0.514	0.524	0.444	0.154	0.064	-0.046	0.014	0.004	-0.616	0.024	-0.436	-0.386	-0.256	0.409	0.011	-0.196	-0.359	-0.768	0.587
PF3D7_092270(	PF3D7_0922700::pre-mRNA-splicing factor 18, putative	-0.006	0.054	0.044	0.274	-0.106	0.074	0.004	0.034	0.074	0.044	-0.256	-0.056	-0.176	0.091	-0.009	0.051	-0.163	-0.254	0.838
PF3D7_031330(	PF3D7_0313300::YGGT family protein, putative	0.182	0.282	0.272	0.132	-0.068	0.322	0.072	-0.008	0.302	-0.118	-0.418	-0.368	-0.588	0.217	0.109	0.059	-0.458	-0.675	0.626
PF3D7_143970(	PF3D7_1439700::leucine carboxyl methyltransferase, putative	0.289	0.249	0.099	0.189	-0.041	0.189	0.069	0.019	0.249	-0.101	-0.611	-0.131	-0.471	0.207	0.073	0.056	-0.404	-0.611	0.655
PF3D7_072730(	PF3D7_0727300::DNA (cytosine-5)-methyltransferase	0.300	0.330	0.060	0.260	-0.080	-0.030	-0.180	0.150	0.200	-0.070	-0.510	-0.270	-0.160	0.238	-0.097	0.093	-0.313	-0.551	0.683
PF3D7_143590(	PF3D7_1435900::conserved Plasmodium protein, unknown function	0.245	0.435	0.115	0.605	0.105	-0.035	0.025	0.005	-0.115	-0.195	-0.645	-0.395	-0.155	0.350	0.032	-0.101	-0.398	-0.748	0.595
PF3D7_031510(	PF3D7_0315100::eukaryotic translation initiation factor 4E	-0.008	0.022	0.052	0.432	0.102	0.512	0.132	0.222	-0.218	-0.068	-0.658	-0.478	-0.048	0.125	0.249	-0.021	-0.394	-0.519	0.698
PF3D7_020920(	PF3D7_0209200::exosome complex component MTR3, putative	-0.175	-0.155	0.175	0.365	0.145	0.585	0.205	0.285	0.015	-0.135	-0.555	-0.395	-0.365	0.053	0.312	0.055	-0.438	-0.491	0.712
PF3D7_102490(	PF3D7_1024900::conserved Plasmodium protein, unknown function	-0.071	0.049	0.049	0.439	0.129	0.579	0.229	0.269	-0.031	-0.181	-0.811	-0.351	-0.301	0.117	0.313	0.019	-0.487	-0.604	0.658
PF3D7_090980(	PF3D7_0909800::small nuclear ribonucleoprotein Sm D3, putative	-0.019	0.041	-0.019	0.181	0.121	0.501	0.071	0.201	0.051	0.101	-0.429	-0.459	-0.339	0.046	0.231	0.117	-0.409	-0.455	0.730
PF3D7_061020(	PF3D7_0610200::RNA-binding protein 25, putative	0.004	0.214	0.484	0.084	0.174	0.234	0.304	-0.006	-0.486	0.044	-0.556	-0.126	-0.366	0.196	0.237	-0.149	-0.349	-0.546	0.685
PF3D7_101760(	PF3D7_1017600::conserved Plasmodium protein, unknown function	0.068	0.008	0.288	0.018	0.028	0.288	0.288	0.228	-0.312	0.038	-0.572	-0.032	-0.332	0.095	0.201	-0.016	-0.312	-0.408	0.754
PF3D7_031790(	PF3D7_0317900::conserved protein, unknown function	0.106	0.736	0.316	0.126	-0.214	0.236	0.236	0.366	-0.234	0.076	-0.924	-0.364	-0.464	0.321	0.086	0.069	-0.584	-0.905	0.534
PF3D7_031920(	PF3D7_0319200::CCR4 domain-containing protein 4, putative	-0.040	0.690	0.280	0.140	-0.030	0.310	0.210	0.180	-0.240	0.000	-0.800	-0.250	-0.450	0.268	0.163	-0.020	-0.500	-0.768	0.587
PF3D7_071150(	PF3D7_0711500::regulator of chromosome condensation, putative	0.134	0.314	0.264	0.184	-0.106	0.274	0.354	0.124	0.004	0.064	-1.066	-0.336	-0.206	0.224	0.174	0.064	-0.536	-0.760	0.590
PF3D7_112050(	PF3D7_1120500::tRNA nucleotidyltransferase, putative	0.077	0.367	0.077	0.237	-0.083	0.377	0.257	0.207	-0.103	-0.013	-0.863	-0.273	-0.263	0.189	0.184	0.030	-0.466	-0.656	0.635
PF3D7_122170(	PF3D7_1221700::FbpA domain protein, putative	0.064	0.474	0.054	0.214	-0.236	0.294	0.254	0.234	-0.106	0.124	-0.856	-0.266	-0.246	0.201	0.104	0.084	-0.456	-0.658	0.634
PF3D7_112820(	PF3D7_1128200::multiprotein-bridging factor 1, putative	0.102	0.482	0.232	0.202	-0.088	0.052	0.132	0.212	-0.078	0.142	-0.898	-0.298	-0.188	0.254	0.032	0.092	-0.462	-0.716	0.609
PF3D7_080170(	PF3D7_0801700::sentrin-specific protease 2, putative	0.153	0.243	0.183	0.063	-0.107	0.383	0.183	0.153	-0.057	0.193	-0.727	-0.337	-0.327	0.161	0.153	0.096	-0.464	-0.624	0.649
PF3D7_082450(	PF3D7_0824500::conserved protein, unknown function	0.167	0.327	0.437	0.327	0.077	0.607	0.497	0.257	-0.143	0.357	-1.453	-0.793	-0.663	0.314	0.394	0.157	-0.970	-1.284	0.411
PF3D7_091620(	PF3D7_0916200::conserved Plasmodium protein, unknown function	0.154	0.434	0.274	0.094	0.064	0.354	0.144	0.114	-0.066	0.004	-0.776	-0.506	-0.286	0.239	0.187	0.017	-0.523	-0.762	0.590
PF3D7_091280(	PF3D7_0912800::tRNA (adenine(58)-N(1))-methyltransferase non-catalyt	-0.055	0.315	0.195	0.165	0.005	0.325	0.155	0.255	-0.145	0.125	-0.645	-0.435	-0.255	0.155	0.161	0.078	-0.445	-0.600	0.660
PF3D7_140970(	PF3D7_1409700::conserved protein, unknown function	0.025	0.425	0.235	0.285	0.045	0.515	0.265	0.145	-0.145	0.095	-0.935	-0.585	-0.365	0.242	0.275	0.031	-0.629	-0.871	0.547
PF3D7_141920(	PF3D7_1419200::thioredoxin-like protein, putative	0.031	0.571	0.251	0.271	0.191	0.651	0.431	0.331	-0.269	-0.089	-1.159	-0.689	-0.519	0.281	0.424	-0.009	-0.789	-1.070	0.476
PF3D7_093410(	PF3D7_0934100::TFIIH basal transcription factor complex helicase XPD	0.058	0.058	0.178	0.078	-0.032	0.498	0.008	0.068	-0.062	0.208	-0.702	-0.252	-0.102	0.093	0.158	0.071	-0.352	-0.445	0.735
PF3D7_092210(	PF3D7_0922100::ubiquitin-like protein, putative	-0.049	0.001	0.301	0.161	0.081	0.361	0.181	0.231	-0.179	0.111	-0.699	-0.279	-0.219	0.103	0.207	0.054	-0.399	-0.503	0.706
PF3D7_103360(	PF3D7_1033600::pre-mRNA-splicing factor CEF1, putative	0.039	0.089	0.369	0.129	-0.011	0.389	0.159	0.109	-0.111	0.159	-0.831	-0.231	-0.261	0.157	0.179	0.053	-0.441	-0.598	0.661
PF3D7_135340(	PF3D7_1353400::Ran-binding protein, putative	0.067	0.067	0.157	0.077	0.007	0.237	0.177	0.147	-0.173	0.017	-0.573	-0.153	-0.053	0.092	0.140	-0.003	-0.260	-0.352	0.784
PF3D7_041090(	PF3D7_0410900::conserved Plasmodium protein, unknown function	0.092	0.212	0.392	0.112	0.002	0.252	-0.008	0.042	-0.118	0.292	-0.728	-0.268	-0.278	0.202	0.082	0.072	-0.424	-0.627	0.648
PF3D7_041530(	PF3D7_0415300::cdc2-related protein kinase 3	0.135	0.185	0.195	-0.005	0.025	0.085	0.075	0.105	-0.045	0.265	-0.585	-0.245	-0.195	0.128	0.062	0.109	-0.341	-0.469	0.722
PF3D7_133350(	PF3D7_1333500::conserved Plasmodium protein, unknown function	0.026	0.376	0.416	0.166	-0.084	0.286	0.136	0.256	-0.114	0.416	-1.094	-0.534	-0.254	0.246	0.113	0.186	-0.627	-0.873	0.546
PF3D7_061820(	PF3D7_0618200::conserved protein, unknown function	0.079	0.359	0.529	0.399	0.039	0.279	0.189	0.209	0.019	0.289	-1.351	-0.561	-0.481	0.342	0.169	0.173	-0.797	-1.139	0.454

PF3D7_132510(	PF3D7_1325100::phosphoribosylpyrophosphate synthetase	0.059	0.109	0.409	0.239	0.159	0.259	0.129	0.149	-0.101	0.169	-1.041	-0.301	-0.241	0.204	0.183	0.073	-0.527	-0.732	0.602
PF3D7_120560(	PF3D7_1205600::tetratricopeptide repeat protein, putative	0.029	0.259	0.479	0.199	0.279	0.079	0.099	0.189	-0.071	0.039	-1.011	-0.261	-0.311	0.242	0.153	0.053	-0.527	-0.769	0.587
PF3D7_140120(	PF3D7_1401200::Plasmodium exported protein, unknown function	0.085	0.235	0.775	0.265	0.155	0.065	0.115	0.105	-0.155	0.315	-1.345	-0.305	-0.315	0.340	0.112	0.089	-0.655	-0.995	0.502
PF3D7_144970(	PF3D7_1449700::exosome complex exonuclease RRP6	0.048	0.038	0.198	0.078	-0.042	0.258	0.198	0.238	0.118	0.308	-0.942	-0.262	-0.232	0.090	0.138	0.221	-0.479	-0.569	0.674
PF3D7_031310(	PF3D7_0313100::ubiquitin-protein ligase, putative	0.024	0.354	0.304	0.194	0.134	0.374	0.124	0.174	-0.066	-0.056	-0.906	-0.296	-0.356	0.219	0.211	0.017	-0.519	-0.738	0.599
PF3D7_060240(	PF3D7_0602400::elongation factor G	0.325	0.325	0.465	0.225	0.045	0.475	0.205	0.165	-0.215	-0.215	-1.125	-0.295	-0.375	0.335	0.241	-0.089	-0.599	-0.933	0.524
PF3D7_093360(	PF3D7_0933600::mitochondrial-processing peptidase subunit beta, puta	0.238	0.328	0.428	0.228	0.048	0.438	0.108	0.088	-0.132	-0.052	-1.082	-0.242	-0.392	0.305	0.198	-0.032	-0.572	-0.878	0.544
PF3D7_101520(	PF3D7_1015200::cysteine--tRNA ligase	0.147	0.237	0.487	0.407	0.217	0.447	0.097	0.117	-0.413	0.027	-1.063	-0.403	-0.303	0.319	0.254	-0.090	-0.590	-0.909	0.532
PF3D7_132640(	PF3D7_1326400::translation initiation factor eIF-2B subunit gamma, puta	0.220	0.450	0.460	0.340	0.230	0.570	0.130	0.210	-0.500	-0.210	-1.160	-0.380	-0.360	0.368	0.310	-0.167	-0.633	-1.001	0.500
PF3D7_123830(	PF3D7_1238300::pre-mRNA-splicing factor CWC22, putative	0.342	0.182	0.472	0.202	-0.038	0.342	0.202	0.172	-0.388	-0.078	-0.878	-0.148	-0.388	0.300	0.169	-0.098	-0.471	-0.771	0.586
PF3D7_070880(	PF3D7_0708800::heat shock protein 110	0.158	0.128	0.308	0.278	0.198	0.218	0.088	0.218	-0.192	-0.212	-0.972	-0.082	-0.142	0.218	0.168	-0.062	-0.398	-0.617	0.652
PF3D7_123700(	PF3D7_1237000::SUMO-activating enzyme subunit 2	0.129	0.129	0.389	0.119	0.029	0.189	0.109	0.149	-0.071	-0.011	-0.881	-0.131	-0.151	0.192	0.109	0.023	-0.387	-0.579	0.669
PF3D7_010980(	PF3D7_0109800::phenylalanine--tRNA ligase alpha subunit	0.045	0.195	0.245	0.425	-0.005	0.405	0.015	0.195	-0.085	-0.085	-0.885	-0.235	-0.235	0.228	0.139	0.009	-0.451	-0.679	0.625
PF3D7_102470(	PF3D7_1024700::conserved protein, unknown function	0.231	0.201	0.401	0.381	0.021	0.331	0.101	0.111	-0.029	-0.259	-0.969	-0.169	-0.349	0.303	0.151	-0.059	-0.496	-0.799	0.575
PF3D7_146160(	PF3D7_1461600::splicing factor 3B subunit 2, putative	0.127	0.107	0.417	0.277	0.007	0.297	0.067	0.147	-0.013	-0.283	-0.813	-0.133	-0.203	0.232	0.124	-0.050	-0.383	-0.615	0.653
PF3D7_040890(	PF3D7_0408900::tRNA N6-adenosine threonylcarbamoyltransferase	0.086	0.646	0.326	0.266	-0.044	0.536	0.046	0.056	-0.074	-0.004	-1.184	-0.284	-0.374	0.331	0.179	-0.007	-0.614	-0.945	0.519
PF3D7_141790(	PF3D7_1417900::ATP synthase-associated protein, putative	0.038	0.438	0.448	0.448	-0.102	0.378	0.028	0.058	-0.042	0.048	-0.942	-0.432	-0.362	0.343	0.101	0.021	-0.579	-0.922	0.528
PF3D7_135690(	PF3D7_1356900::protein kinase 5	0.149	0.599	0.369	0.529	-0.011	0.209	-0.031	0.119	0.089	-0.291	-1.161	-0.221	-0.351	0.412	0.056	-0.027	-0.577	-0.989	0.504
PF3D7_124310(	PF3D7_1243100::zinc finger protein, putative	0.435	0.685	0.725	0.515	-0.165	0.585	0.195	0.085	-0.175	-0.415	-1.265	-0.525	-0.675	0.590	0.205	-0.169	-0.822	-1.412	0.376
PF3D7_131570(	PF3D7_1315700::tRNA (adenine(58)-N(1))-methyltransferase catalytic su	0.092	0.242	0.392	0.172	-0.098	0.252	0.072	-0.098	0.042	-0.158	-0.508	-0.168	-0.238	0.225	0.076	-0.071	-0.304	-0.529	0.693
PF3D7_040730(	PF3D7_0407300::transcription factor, putative	0.251	0.261	0.401	0.031	-0.379	0.401	0.241	0.351	-0.379	0.221	-0.629	-0.449	-0.319	0.236	0.087	0.064	-0.466	-0.702	0.615
PF3D7_052430(	PF3D7_0524300::conserved Plasmodium protein, unknown function	0.096	0.286	0.206	0.146	-0.304	0.426	0.246	0.136	-0.164	0.006	-0.574	-0.264	-0.244	0.184	0.123	-0.007	-0.361	-0.544	0.686
PF3D7_080600(	PF3D7_0806000::AAA family ATPase, putative	0.198	0.088	0.138	-0.002	-0.032	0.188	0.118	0.088	-0.152	0.188	-0.312	-0.112	-0.392	0.105	0.091	0.041	-0.272	-0.378	0.770
PF3D7_060340(	PF3D7_0603400::trophozoite exported protein 1	0.145	-0.015	0.175	0.035	0.015	0.475	0.255	0.055	-0.155	0.135	-0.605	-0.345	-0.165	0.085	0.248	0.011	-0.372	-0.457	0.729
PF3D7_133730(	PF3D7_1337300::exoribonuclease, putative	0.162	-0.038	0.112	0.082	0.032	0.682	0.282	0.162	-0.178	0.052	-0.538	-0.418	-0.398	0.080	0.332	0.012	-0.451	-0.531	0.692
PF3D7_145060(	PF3D7_1450600::S-adenosylmethionine-dependent methyltransferase,		0.275	-0.025	0.235	0.245	0.065	0.605	0.355	0.145	-0.175	0.115	-0.815	-0.425	-0.595	0.182	0.341	0.028	-0.612	-0.794	0.577
PF3D7_134400(	PF3D7_1344000::aminomethyltransferase, putative	0.194	0.034	0.254	0.014	0.014	0.404	0.184	0.004	-0.156	0.194	-0.316	-0.406	-0.416	0.124	0.201	0.014	-0.379	-0.503	0.705
PF3D7_113260(	PF3D7_1132600::pre-mRNA-splicing factor 38A, putative	-0.034	-0.044	0.226	0.116	-0.294	0.526	0.296	0.136	0.026	0.066	-0.494	-0.324	-0.204	0.066	0.176	0.076	-0.341	-0.407	0.754
PF3D7_147200(	PF3D7_1472000::pre-mRNA-splicing factor ISY1, putative	0.088	-0.042	0.428	0.248	-0.262	0.238	0.318	0.178	0.198	0.008	-0.532	-0.472	-0.392	0.180	0.098	0.128	-0.466	-0.646	0.639
PF3D7_010970(	PF3D7_0109700::rRNA biogenesis protein RRP36, putative	-0.010	0.450	0.270	0.150	-0.020	0.020	-0.140	0.100	0.000	0.280	-0.460	-0.330	-0.310	0.215	-0.047	0.127	-0.367	-0.582	0.668
PF3D7_062870(	PF3D7_0628700::conserved Plasmodium protein, unknown function	-0.042	0.678	0.208	0.258	-0.062	0.378	-0.212	0.118	0.028	0.388	-0.852	-0.512	-0.382	0.276	0.035	0.178	-0.582	-0.858	0.552
PF3D7_090680(	PF3D7_0906800::conserved Plasmodium protein, unknown function	-0.069	0.601	0.131	0.281	0.191	0.281	-0.079	0.121	-0.229	0.241	-0.769	-0.329	-0.369	0.236	0.131	0.044	-0.489	-0.725	0.605
PF3D7_112730(	PF3D7_1127300::tRNA (guanine-N(7)-)-methyltransferase, putative	0.259	0.719	0.419	0.279	-0.011	0.299	-0.101	-0.161	-0.241	0.269	-0.871	-0.341	-0.521	0.419	0.063	-0.044	-0.577	-0.997	0.501
PF3D7_111500(	PF3D7_1115000::conserved protein, unknown function	0.168	0.718	0.318	0.348	0.068	0.138	-0.122	-0.032	-0.012	0.328	-0.952	-0.542	-0.422	0.388	0.028	0.094	-0.639	-1.027	0.491
PF3D7_122400(	PF3D7_1224000::GTP cyclohydrolase 1	0.142	0.732	0.292	0.232	0.082	0.102	-0.098	0.112	-0.208	0.152	-0.838	-0.368	-0.328	0.349	0.028	0.018	-0.512	-0.861	0.551
PF3D7_134650(	PF3D7_1346500::conserved Plasmodium protein, unknown function	0.119	0.899	0.459	0.309	-0.021	0.019	-0.101	0.159	-0.121	0.059	-0.911	-0.471	-0.401	0.447	-0.034	0.033	-0.594	-1.041	0.486
PF3D7_062320(	PF3D7_0623200::ferredoxin--NADP reductase	0.032	0.412	0.132	0.002	-0.018	0.122	-0.088	-0.028	-0.018	0.142	-0.378	-0.208	-0.108	0.145	0.006	0.032	-0.231	-0.376	0.771
PF3D7_093680(	PF3D7_0936800::Plasmodium exported protein (PHISTc), unknown func	0.252	0.642	0.182	-0.038	0.032	0.112	0.062	0.002	-0.018	0.252	-0.758	-0.388	-0.328	0.259	0.068	0.078	-0.492	-0.751	0.594
PF3D7_141270(	PF3D7_1412700::AAA family ATPase, putative	0.194	0.674	0.264	-0.106	0.014	0.384	0.044	-0.076	-0.076	0.374	-0.736	-0.406	-0.546	0.256	0.147	0.074	-0.563	-0.819	0.567
PF3D7_090630(	PF3D7_0906300::Maf-like protein, putative	0.112	0.562	0.482	0.152	-0.008	0.042	0.132	0.092	-0.158	-0.068	-0.798	-0.518	-0.018	0.327	0.055	-0.045	-0.445	-0.772	0.586
PF3D7_102150(	PF3D7_1021500::ATP-dependent RNA helicase ROK1, putative	0.121	0.491	0.481	0.041	-0.079	0.251	0.121	0.071	-0.029	-0.009	-0.629	-0.309	-0.519	0.283	0.097	0.011	-0.486	-0.769	0.587
PF3D7_062240(	PF3D7_0622400::conserved protein, unknown function	0.173	0.553	0.443	0.113	-0.067	-0.007	0.053	-0.027	-0.117	0.193	-0.597	-0.377	-0.337	0.321	-0.007	0.016	-0.437	-0.758	0.592
PF3D7_124260(	PF3D7_1242600::protein farnesyltransferase subunit alpha	0.285	0.435	0.425	0.055	0.015	0.145	0.065	0.015	-0.125	0.065	-0.605	-0.405	-0.375	0.300	0.075	-0.015	-0.461	-0.762	0.590
PF3D7_124150(	PF3D7_1241500::conserved Plasmodium protein, unknown function	0.409	0.769	0.649	0.219	-0.151	0.149	0.159	0.129	-0.191	-0.131	-1.131	-0.441	-0.441	0.512	0.053	-0.064	-0.671	-1.183	0.441
PF3D7_040740(	PF3D7_0407400::conserved Plasmodium protein, unknown function	-0.091	0.729	0.399	-0.021	-0.101	0.239	0.249	0.239	-0.111	0.839	-0.871	-0.731	-0.771	0.254	0.129	0.323	-0.791	-1.045	0.485
PF3D7_111920(	PF3D7_1119200::conserved protein, unknown function	-0.267	0.653	0.403	-0.037	-0.207	0.163	0.173	0.143	0.033	1.133	-0.847	-0.647	-0.697	0.188	0.043	0.436	-0.730	-0.918	0.529
PF3D7_136510(	PF3D7_1365100::ribosomal protein S17, mitochondrial	-0.035	0.715	0.205	0.025	-0.065	0.095	0.045	0.255	0.165	0.725	-0.925	-0.725	-0.485	0.228	0.025	0.382	-0.711	-0.939	0.522
PF3D7_082010(	PF3D7_0820100::RNA-binding protein, putative	-0.035	0.455	0.275	0.255	-0.015	0.225	0.125	0.065	0.015	0.485	-0.755	-0.565	-0.525	0.237	0.111	0.188	-0.615	-0.853	0.554
PF3D7_111910(	PF3D7_1119100::tRNA m(1)G methyltransferase, putative	-0.046	0.404	0.304	0.264	-0.096	0.034	0.084	0.244	-0.076	0.364	-0.706	-0.366	-0.406	0.231	0.007	0.177	-0.493	-0.724	0.605
PF3D7_112980(	PF3D7_1129800::conserved Plasmodium protein, unknown function	-0.112	0.648	0.328	0.068	-0.142	0.208	0.108	0.038	0.048	0.608	-0.932	-0.472	-0.392	0.233	0.058	0.231	-0.599	-0.832	0.562
PF3D7_141820(	PF3D7_1418200::conserved Plasmodium protein, unknown function	0.074	0.654	0.294	0.024	-0.196	0.304	0.084	0.074	-0.146	0.504	-0.976	-0.386	-0.306	0.261	0.064	0.144	-0.556	-0.818	0.567
PF3D7_141220(	PF3D7_1412200::conserved Plasmodium protein, unknown function	0.149	0.439	0.319	-0.121	-0.111	0.429	0.149	0.069	-0.041	0.449	-0.621	-0.631	-0.481	0.197	0.156	0.159	-0.577	-0.774	0.585
PF3D7_145910(	PF3D7_1459100::GTP-binding protein, putative	0.132	0.422	0.372	0.022	-0.028	0.182	0.022	0.062	-0.178	0.412	-0.518	-0.748	-0.148	0.237	0.058	0.098	-0.472	-0.708	0.612
PF3D7_122640(	PF3D7_1226400::regulator of MON1-CCZ1 complex, putative	0.108	0.618	0.158	0.178	-0.152	0.318	0.038	0.108	0.128	0.178	-0.582	-0.732	-0.372	0.266	0.068	0.138	-0.562	-0.828	0.564
PF3D7_135020(	PF3D7_1350200::small nuclear ribonucleoprotein E, putative	-0.150	0.400	0.080	0.310	0.060	0.240	-0.030	0.250	0.130	0.350	-0.650	-0.560	-0.430	0.160	0.090	0.243	-0.547	-0.707	0.613
PF3D7_136620(	PF3D7_1366200::conserved protein, unknown function	0.243	0.283	0.183	0.133	-0.077	0.033	0.083	-0.077	-0.027	0.313	-0.407	-0.297	-0.387	0.211	0.013	0.070	-0.364	-0.574	0.672
PF3D7_082480(	PF3D7_0824800::conserved Plasmodium membrane protein, unknown fu	-0.112	0.448	0.008	0.008	-0.162	0.278	0.158	0.158	-0.072	0.278	-0.532	-0.262	-0.192	0.088	0.091	0.121	-0.329	-0.417	0.749
PF3D7_100470(	PF3D7_1004700::conserved protein, unknown function	0.118	0.458	-0.092	0.078	-0.212	0.518	0.198	0.028	-0.072	0.238	-0.602	-0.452	-0.202	0.140	0.168	0.064	-0.419	-0.559	0.679
PF3D7_141830(	PF3D7_1418300::calmodulin, putative	0.094	0.554	0.054	0.114	-0.266	0.504	0.104	-0.016	-0.036	0.404	-0.736	-0.446	-0.326	0.204	0.114	0.117	-0.503	-0.707	0.613
PF3D7_134820(	PF3D7_1348200::step II splicing factor, putative	0.118	1.018	-0.002	0.218	-0.342	0.268	0.058	-0.052	-0.022	0.118	-0.642	-0.362	-0.382	0.338	-0.005	0.015	-0.462	-0.800	0.574
PF3D7_147280(	PF3D7_1472800::HSP20-like chaperone, putative	0.198	0.528	0.108	0.088	-0.242	0.158	0.018	-0.102	0.208	0.168	-0.552	-0.252	-0.332	0.231	-0.022	0.092	-0.378	-0.609	0.656
PF3D7_072080(	PF3D7_0720800::Ham1-like protein, putative	0.086	0.586	0.186	0.186	-0.014	-0.094	-0.094	0.036	0.046	-0.044	-0.294	-0.444	-0.144	0.261	-0.067	0.013	-0.294	-0.555	0.681
PF3D7_124710(	PF3D7_1247100::conserved protein, unknown function	0.115	0.555	0.235	0.075	-0.135	-0.055	-0.045	0.085	0.105	0.105	-0.515	-0.355	-0.175	0.245	-0.078	0.099	-0.348	-0.593	0.663
PF3D7_147020(	PF3D7_1470200::conserved Plasmodium protein, unknown function	-0.012	0.508	0.428	0.168	-0.142	0.028	-0.112	0.078	0.048	0.128	-0.322	-0.352	-0.452	0.273	-0.075	0.085	-0.375	-0.648	0.638
PF3D7_031950(	PF3D7_0319500::RNA-binding protein, putative	0.525	0.205	0.515	0.165	0.135	0.155	0.255	0.135	-0.345	-0.685	-0.775	0.025	-0.315	0.353	0.182	-0.298	-0.355	-0.708	0.612
PF3D7_053070(	PF3D7_0530700::conserved Plasmodium protein, unknown function	0.258	0.058	0.608	0.298	0.078	0.188	0.108	0.128	-0.402	-0.252	-0.532	-0.322	-0.222	0.306	0.125	-0.175	-0.358	-0.664	0.631
PF3D7_120350(	PF3D7_1203500::threonylcarbamoyl-AMP synthase, putative	0.285	0.115	0.385	0.225	0.035	0.085	0.085	0.015	-0.255	-0.255	-0.455	-0.105	-0.165	0.253	0.069	-0.165	-0.241	-0.494	0.710
PF3D7_060330(	PF3D7_0603300::dihydroorotate dehydrogenase	0.299	0.129	0.469	0.289	0.179	0.079	-0.021	0.209	-0.581	-0.261	-0.341	-0.281	-0.171	0.297	0.079	-0.211	-0.264	-0.561	0.678
PF3D7_134490(	PF3D7_1344900::conserved Plasmodium protein, unknown function	0.281	0.161	0.221	0.131	0.131	0.211	0.051	0.121	-0.419	-0.339	-0.219	-0.249	-0.079	0.198	0.131	-0.213	-0.183	-0.381	0.768
PF3D7_011300(	PF3D7_0113000::glutamic acid-rich protein GARP	0.445	0.055	1.165	0.435	0.805	0.105	-0.205	0.595	-0.675	-0.465	-0.975	-0.665	-0.615	0.525	0.235	-0.182	-0.752	-1.277	0.413
PF3D7_052750(	PF3D7_0527500::Hsc70-interacting protein	0.398	0.168	0.968	0.238	0.448	0.008	-0.112	0.168	-0.472	-0.492	-0.582	-0.242	-0.492	0.443	0.114	-0.266	-0.439	-0.882	0.543
PF3D7_040940(	PF3D7_0409400::chaperone protein DnaJ	0.485	0.315	0.565	0.085	0.235	0.165	-0.095	0.105	-0.385	-0.235	-0.345	-0.485	-0.405	0.362	0.101	-0.172	-0.412	-0.774	0.585
PF3D7_072940(	PF3D7_0729400::ribosome biogenesis protein BRX1, putative	0.409	0.039	0.539	0.209	0.209	0.129	-0.181	0.079	-0.431	-0.121	-0.291	-0.291	-0.301	0.299	0.053	-0.157	-0.294	-0.593	0.663
PF3D7_082310(	PF3D7_0823100::RWD domain-containing protein, putative	0.448	0.208	0.578	0.528	0.398	-0.012	-0.082	0.088	-0.482	-0.142	-0.512	-0.442	-0.572	0.440	0.101	-0.179	-0.509	-0.949	0.518
PF3D7_011070(	PF3D7_0110700::chromatin assembly factor 1 subunit C, putative	0.228	0.398	0.598	0.218	0.358	0.118	-0.202	-0.042	-0.392	-0.182	-0.432	-0.252	-0.412	0.360	0.091	-0.206	-0.366	-0.726	0.605
PF3D7_134630(	PF3D7_1346300::DNA/RNA-binding protein Alba 2	0.262	0.162	0.342	0.142	0.102	0.142	-0.128	0.032	-0.248	-0.048	-0.338	-0.118	-0.308	0.227	0.039	-0.088	-0.254	-0.482	0.716
PF3D7_092120(	PF3D7_0921200::conserved Plasmodium membrane protein, unknown fu	0.211	0.121	0.331	0.081	0.241	0.191	-0.009	0.271	-0.289	-0.109	-0.399	-0.309	-0.329	0.186	0.141	-0.043	-0.346	-0.532	0.692
PF3D7_144800(	PF3D7_1448000::U3 small nucleolar RNA-associated protein 12, putative	0.246	0.006	0.326	0.306	0.116	0.086	0.106	0.326	-0.294	-0.124	-0.484	-0.344	-0.274	0.221	0.103	-0.031	-0.367	-0.588	0.665
PF3D7_135810(	PF3D7_1358100::Sas10 domain-containing protein, putative	0.447	-0.063	0.557	0.127	0.037	0.267	0.107	0.177	-0.413	0.027	-0.483	-0.413	-0.373	0.267	0.137	-0.070	-0.423	-0.690	0.620
PF3D7_062220(	PF3D7_0622200::radical SAM protein, putative	0.088	-0.012	0.468	0.158	0.058	0.118	-0.002	0.158	-0.222	0.038	-0.502	-0.062	-0.292	0.176	0.058	-0.008	-0.285	-0.461	0.727
PF3D7_071790(	PF3D7_0717900::thioredoxin-like protein	-0.002	0.168	0.618	0.308	0.038	0.378	0.018	0.118	-0.332	0.268	-0.612	-0.472	-0.502	0.273	0.145	0.018	-0.528	-0.802	0.574
PF3D7_052150(	PF3D7_0521500::ribosomal large subunit pseudouridylate synthase, put	-0.030	0.190	0.540	0.410	0.050	0.380	0.110	0.240	-0.230	0.140	-0.970	-0.340	-0.490	0.278	0.180	0.050	-0.600	-0.878	0.544
PF3D7_123080(	PF3D7_1230800::pre-mRNA-splicing regulator WTAP, putative	0.036	0.266	0.286	0.216	0.056	0.216	-0.024	0.226	-0.154	0.126	-0.634	-0.324	-0.294	0.201	0.083	0.066	-0.417	-0.618	0.651
PF3D7_060500(	PF3D7_0605000::50S ribosomal protein L24, putative	0.160	0.260	0.570	0.460	0.050	0.100	0.110	-0.020	-0.250	0.150	-0.610	-0.380	-0.600	0.363	0.087	-0.040	-0.530	-0.893	0.539
PF3D7_061450(	PF3D7_0614500::60S ribosomal protein L19	0.097	0.667	0.947	0.367	0.137	-0.013	-0.013	0.147	-0.283	-0.033	-1.083	-0.413	-0.523	0.519	0.037	-0.056	-0.673	-1.193	0.438
PF3D7_130700(	PF3D7_1307000::exosome complex component RRP40, putative	0.044	0.614	0.684	0.204	0.084	0.014	-0.066	0.104	-0.296	0.194	-0.856	-0.326	-0.396	0.386	0.011	0.001	-0.526	-0.913	0.531
PF3D7_131790(	PF3D7_1317900::nucleolar complex protein 4, putative	0.090	0.430	0.440	0.220	0.100	0.040	-0.040	0.170	-0.200	0.020	-0.590	-0.400	-0.280	0.295	0.033	-0.003	-0.423	-0.718	0.608
PF3D7_124480(	PF3D7_1244800::translation machinery-associated protein 46, putative	0.279	0.289	0.589	0.209	0.139	0.079	-0.051	0.229	-0.251	0.099	-0.951	-0.341	-0.321	0.342	0.056	0.026	-0.537	-0.879	0.544
PF3D7_052100(	PF3D7_0521000::conserved Plasmodium protein, unknown function	0.104	0.304	0.874	0.404	0.004	0.254	0.124	0.254	-0.226	-0.106	-0.876	-0.446	-0.666	0.421	0.127	-0.026	-0.663	-1.084	0.472
PF3D7_120760(	PF3D7_1207600::tRNA dimethylallyltransferase, putative	0.070	0.330	0.560	0.310	0.100	0.060	0.110	0.210	-0.300	0.040	-0.710	-0.330	-0.450	0.318	0.090	-0.017	-0.497	-0.814	0.569
PF3D7_124660(	PF3D7_1246600::pre-mRNA-splicing factor CWC26, putative	-0.024	0.166	0.756	0.276	0.006	0.126	0.156	0.226	-0.244	0.146	-0.814	-0.344	-0.434	0.294	0.096	0.043	-0.531	-0.824	0.565
PF3D7_144570(	PF3D7_1445700::conserved Plasmodium protein, unknown function	0.124	0.234	0.884	0.324	0.154	0.084	-0.046	0.234	-0.186	-0.056	-0.866	-0.506	-0.376	0.391	0.064	-0.003	-0.583	-0.974	0.509
PF3D7_020820(	PF3D7_0208200::KRR1 small subunit processome component, putative	0.039	0.469	0.779	0.219	0.189	-0.201	-0.171	0.109	-0.271	0.179	-0.501	-0.381	-0.461	0.377	-0.061	0.006	-0.447	-0.824	0.565
PF3D7_133710(	PF3D7_1337100::protein kinase 6	0.157	0.417	0.667	0.387	0.247	-0.203	-0.063	0.107	-0.353	0.057	-0.583	-0.363	-0.473	0.407	-0.006	-0.063	-0.473	-0.880	0.543
PF3D7_112940(	PF3D7_1129400::rRNA (cytosine-C(5))-methyltransferase, putative	0.141	0.621	0.731	0.251	0.271	0.051	-0.149	0.211	-0.269	-0.299	-0.599	-0.609	-0.349	0.436	0.057	-0.119	-0.519	-0.955	0.516
PF3D7_130910(	PF3D7_1309100::60S ribosomal protein L24, putative	0.138	0.558	0.828	0.388	0.248	-0.102	-0.162	0.088	-0.152	-0.112	-0.802	-0.432	-0.492	0.478	-0.005	-0.058	-0.575	-1.053	0.482
PF3D7_143170(	PF3D7_1431700::60S ribosomal protein L14, putative	0.158	0.438	0.678	0.348	0.228	0.018	-0.142	0.178	-0.002	-0.282	-0.632	-0.492	-0.492	0.405	0.034	-0.036	-0.539	-0.944	0.520
PF3D7_072720(	PF3D7_0727200::cysteine desulfurase IscS, putative	0.232	0.082	0.752	0.102	0.162	0.432	-0.168	0.382	-0.118	-0.468	-0.818	-0.278	-0.298	0.292	0.142	-0.068	-0.464	-0.757	0.592
PF3D7_072240(	PF3D7_0722400::Obg-like ATPase 1, putative	0.144	0.084	0.614	0.454	0.404	0.284	0.114	0.274	-0.156	-0.516	-0.946	-0.276	-0.476	0.324	0.267	-0.133	-0.566	-0.890	0.540

PF3D7_081080(	PF3D7_0810800::hydroxymethyldihydropterin pyrophosphokinase-dihydr	0.138	0.158	0.478	0.588	0.508	0.098	0.138	0.288	-0.302	-0.482	-0.912	-0.372	-0.322	0.340	0.248	-0.166	-0.536	-0.876	0.545
PF3D7_120430(	PF3D7_1204300::eukaryotic translation initiation factor 5A	-0.052	0.138	0.898	0.518	0.348	0.208	0.058	0.248	-0.242	-0.302	-1.012	-0.352	-0.452	0.375	0.204	-0.099	-0.606	-0.981	0.507
PF3D7_143600(	PF3D7_1436000::glucose-6-phosphate isomerase	0.087	0.277	0.637	0.407	0.347	0.227	0.017	0.347	-0.313	-0.233	-1.113	-0.463	-0.223	0.352	0.197	-0.066	-0.600	-0.952	0.517
PF3D7_031550(	PF3D7_0315500::ribosomal protein L47, mitochondrial, putative	0.162	0.102	0.372	0.222	0.162	0.232	0.112	0.032	-0.118	-0.238	-0.638	-0.118	-0.278	0.214	0.168	-0.108	-0.345	-0.559	0.679
PF3D7_135890(	PF3D7_1358900::GTP-binding protein, putative	0.249	0.079	0.609	0.279	0.359	0.419	0.209	0.219	-0.251	-0.341	-1.001	-0.361	-0.471	0.304	0.329	-0.124	-0.611	-0.915	0.530
PF3D7_142040(	PF3D7_1420400::glycine--tRNA ligase	0.149	0.089	0.339	0.189	0.179	0.329	0.029	0.159	-0.161	-0.101	-0.711	-0.181	-0.311	0.192	0.179	-0.034	-0.401	-0.593	0.663
PF3D7_143790(	PF3D7_1437900::HSP40, subfamily A	0.291	0.091	0.681	0.171	0.361	0.241	0.001	0.251	-0.299	-0.249	-0.979	-0.209	-0.349	0.308	0.201	-0.099	-0.513	-0.821	0.566
PF3D7_101260(	PF3D7_1012600::GMP synthase [glutamine-hydrolyzing]	0.246	0.186	0.906	0.476	0.456	0.226	0.126	0.196	-0.414	-0.104	-1.114	-0.714	-0.474	0.454	0.269	-0.107	-0.767	-1.221	0.429
PF3D7_142830(	PF3D7_1428300::proliferation-associated protein 2g4, putative	0.159	0.179	0.599	0.359	0.269	0.329	-0.021	0.109	-0.261	-0.101	-0.721	-0.621	-0.281	0.324	0.193	-0.084	-0.541	-0.865	0.549
PF3D7_132550(	PF3D7_1325500::conserved Plasmodium protein, unknown function	0.316	-0.004	0.556	0.376	0.236	0.476	0.206	0.066	-0.314	-0.254	-0.694	-0.624	-0.344	0.311	0.306	-0.167	-0.554	-0.865	0.549
PF3D7_142760(	PF3D7_1427600::CorA-like Mg2+ transporter protein, putative	0.007	0.287	0.517	0.247	0.227	0.437	0.177	-0.063	-0.323	-0.063	-0.713	-0.473	-0.263	0.264	0.280	-0.150	-0.483	-0.748	0.596
PF3D7_133680(	PF3D7_1336800::nuclear movement protein, putative	0.228	0.268	0.698	0.128	0.428	0.348	-0.132	0.008	-0.142	-0.132	-0.732	-0.292	-0.672	0.330	0.214	-0.089	-0.566	-0.896	0.537
PF3D7_136480(	PF3D7_1364800::DNA-directed RNA polymerases I, II, and III subunit RI	0.396	0.306	0.936	0.626	0.136	0.146	0.186	0.396	-0.254	-0.354	-0.934	-0.794	-0.794	0.566	0.156	-0.071	-0.841	-1.407	0.377
PF3D7_140200(	PF3D7_1402000::conserved Plasmodium protein, unknown function	0.145	0.265	0.625	0.345	0.425	0.075	0.085	0.215	-0.285	-0.185	-0.495	-0.685	-0.535	0.345	0.195	-0.085	-0.571	-0.917	0.530
PF3D7_141260(	PF3D7_1412600::deoxyhypusine synthase	-0.046	-0.086	0.894	0.444	0.494	0.134	0.014	0.324	-0.236	-0.276	-0.586	-0.526	-0.546	0.301	0.214	-0.063	-0.553	-0.854	0.553
PF3D7_061720(	PF3D7_0617200::BFR1 domain-containing protein, putative	0.079	0.179	0.539	0.139	-0.101	-0.151	0.089	0.149	-0.091	0.049	-0.581	-0.161	-0.141	0.234	-0.054	0.036	-0.294	-0.528	0.693
PF3D7_091710(	PF3D7_0917100::N-glycosylase/DNA lyase, putative	0.040	0.260	0.520	0.080	0.010	-0.230	-0.070	0.110	-0.150	0.110	-0.440	-0.150	-0.090	0.225	-0.097	0.023	-0.227	-0.452	0.731
PF3D7_093690(	PF3D7_0936900::Plasmodium exported protein (PHISTb), unknown func	0.776	0.906	1.176	0.156	0.276	-0.114	-0.314	0.286	-0.504	0.566	-1.694	-1.174	-0.344	0.754	-0.051	0.116	-1.071	-1.824	0.282
PF3D7_145840(	PF3D7_1458400::aminodeoxychorismate lyase	0.172	0.252	0.592	0.142	0.172	0.122	-0.188	0.062	-0.348	0.152	-0.738	-0.358	-0.038	0.290	0.036	-0.044	-0.378	-0.668	0.630
PF3D7_146530(	PF3D7_1465300::ribonuclease Z, putative	0.275	0.215	0.565	0.055	0.195	0.005	-0.305	0.015	-0.305	0.045	-0.425	-0.195	-0.135	0.277	-0.035	-0.082	-0.252	-0.529	0.693
PF3D7_133470(	PF3D7_1334700::MSP7-like protein	0.132	0.472	0.512	0.292	0.382	-0.188	-0.058	0.082	-0.198	0.312	-1.008	-0.188	-0.548	0.352	0.046	0.066	-0.581	-0.933	0.524
PF3D7_133690(	PF3D7_1336900::tryptophan--tRNA ligase	0.267	0.287	0.217	0.237	0.087	-0.043	-0.283	0.147	-0.093	0.137	-0.723	-0.093	-0.143	0.252	-0.080	0.064	-0.320	-0.572	0.673
PF3D7_140240(	PF3D7_1402400::zinc finger protein, putative	0.052	0.162	0.172	0.152	-0.038	-0.088	-0.058	0.172	0.052	0.112	-0.478	-0.068	-0.148	0.135	-0.061	0.112	-0.231	-0.366	0.776
PF3D7_062230(	PF3D7_0622300::vacuolar transporter chaperone, putative	0.077	0.177	0.517	0.087	-0.073	0.127	-0.063	0.077	0.037	0.257	-0.543	-0.253	-0.423	0.214	-0.003	0.124	-0.406	-0.621	0.650
PF3D7_010710(	PF3D7_0107100::conserved protein, unknown function	-0.045	0.125	0.635	0.445	0.015	0.125	-0.025	0.235	-0.125	0.305	-0.865	-0.505	-0.325	0.290	0.039	0.139	-0.565	-0.855	0.553
PF3D7_136550(	PF3D7_1365500::aminomethyltransferase, mitochondrial, putative	-0.062	-0.062	0.598	0.298	-0.042	0.128	0.048	0.088	-0.132	0.458	-0.772	-0.332	-0.212	0.193	0.044	0.138	-0.439	-0.632	0.645
PF3D7_130850(	PF3D7_1308500::conserved Plasmodium protein, unknown function	-0.075	-0.105	0.465	0.265	-0.125	0.145	0.025	0.125	-0.045	0.305	-0.405	-0.235	-0.345	0.138	0.015	0.129	-0.328	-0.466	0.724
PF3D7_090730(	PF3D7_0907300::prefoldin-like protein, putative	0.030	-0.140	0.640	0.540	0.210	0.330	0.030	0.250	-0.190	0.230	-0.770	-0.670	-0.490	0.268	0.190	0.097	-0.643	-0.911	0.532
PF3D7_143180(	PF3D7_1431800::apyrase, putative	-0.239	-0.259	0.541	0.561	0.381	0.121	0.051	0.221	-0.149	0.231	-0.639	-0.389	-0.429	0.151	0.184	0.101	-0.486	-0.637	0.643
PF3D7_131390(	PF3D7_1313900::ankyrin-repeat protein, putative	-0.093	-0.073	0.227	0.487	0.197	0.217	0.077	0.317	-0.183	0.107	-0.673	-0.423	-0.183	0.137	0.164	0.080	-0.426	-0.563	0.677
PF3D7_031660(	PF3D7_0316600::formate-nitrite transporter	0.032	0.072	0.032	0.032	0.052	0.322	0.162	0.092	-0.018	0.352	-0.388	-0.488	-0.248	0.042	0.178	0.142	-0.375	-0.417	0.749
PF3D7_123340(	PF3D7_1233400::conserved Plasmodium membrane protein, unknown fu	-0.092	0.008	0.208	0.228	0.058	0.348	0.088	0.088	-0.052	0.528	-0.572	-0.382	-0.452	0.088	0.164	0.188	-0.469	-0.557	0.680
PF3D7_102200(	PF3D7_1022000::RNA-binding protein UIS12, putative	0.010	0.190	0.260	0.110	0.190	0.440	0.190	0.150	-0.200	0.390	-0.880	-0.550	-0.300	0.143	0.273	0.113	-0.577	-0.719	0.607
PF3D7_143850(	PF3D7_1438500::cleavage and polyadenylation specificity factor subunit	-0.095	0.025	0.285	0.225	0.125	0.375	0.135	0.075	-0.165	0.435	-0.695	-0.465	-0.255	0.110	0.211	0.115	-0.472	-0.582	0.668
PF3D7_060730(	PF3D7_0607300::uroporphyrinogen III decarboxylase	0.066	0.146	0.316	0.186	0.156	0.006	-0.074	-0.124	-0.054	0.516	-0.454	-0.394	-0.294	0.179	0.029	0.113	-0.381	-0.559	0.679
PF3D7_141690(	PF3D7_1416900::prefoldin subunit 2, putative	0.108	0.268	0.438	0.428	0.228	0.148	-0.022	-0.042	-0.092	0.368	-0.852	-0.572	-0.402	0.310	0.118	0.078	-0.609	-0.919	0.529
PF3D7_140270(	PF3D7_1402700::U2 snRNP-associated SURP motif-containing protein,	0.017	0.107	0.207	0.107	0.287	0.157	-0.173	0.107	0.087	0.297	-0.573	-0.403	-0.223	0.109	0.090	0.164	-0.400	-0.509	0.703
PF3D7_021910(	PF3D7_0219100::saccharopine dehydrogenase, putative	0.178	0.098	0.378	0.138	0.278	0.038	-0.492	0.328	-0.102	0.688	-0.512	-0.752	-0.272	0.198	-0.058	0.305	-0.512	-0.710	0.611
PF3D7_136540(	PF3D7_1365400::adenylyltransferase and sulfurtransferase UBA4, putat	-0.100	-0.010	0.200	0.200	0.120	0.140	-0.270	0.380	-0.080	0.620	-0.460	-0.540	-0.200	0.073	-0.003	0.307	-0.400	-0.473	0.721
PF3D7_100660(	PF3D7_1006600::phosducin-like protein 3, putative	-0.070	0.130	0.570	0.410	-0.020	-0.010	-0.330	0.060	0.090	0.780	-0.560	-0.700	-0.350	0.260	-0.120	0.310	-0.537	-0.797	0.576
PF3D7_020230(	PF3D7_0202300::Plasmodium exported protein (hyp11), unknown functi	0.049	0.199	0.709	0.199	0.839	-0.111	-0.321	0.229	-0.521	0.279	-0.421	-0.581	-0.551	0.289	0.136	-0.004	-0.517	-0.807	0.572
PF3D7_145950(	PF3D7_1459500::conserved Plasmodium protein, unknown function	0.015	0.025	0.495	0.385	0.465	0.285	-0.455	0.045	-0.195	0.455	-0.505	-0.495	-0.515	0.230	0.098	0.101	-0.505	-0.735	0.601
PF3D7_113420(	PF3D7_1134200::conserved Plasmodium protein, unknown function	0.011	0.161	0.341	0.301	0.151	-0.069	-0.009	0.221	-0.249	0.171	-0.469	-0.369	-0.189	0.203	0.024	0.047	-0.343	-0.546	0.685
PF3D7_135790(	PF3D7_1357900::pyrroline-5-carboxylate reductase, putative	-0.024	0.266	0.756	0.426	0.236	-0.274	-0.374	0.386	-0.144	0.376	-0.744	-0.524	-0.364	0.356	-0.137	0.206	-0.544	-0.900	0.536
PF3D7_140820(	PF3D7_1408200::AP2 domain transcription factor AP2-G2	-0.045	0.005	0.245	0.015	0.205	-0.065	-0.025	0.145	-0.135	0.225	-0.295	-0.185	-0.095	0.055	0.039	0.079	-0.191	-0.247	0.843
PF3D7_061990(	PF3D7_0619900::splicing factor 3A subunit 2, putative	0.410	0.500	0.080	0.100	-0.080	0.370	0.140	-0.290	-0.480	-0.340	-0.080	-0.190	-0.140	0.273	0.143	-0.370	-0.137	-0.409	0.753
PF3D7_113160(	PF3D7_1131600::kelch domain-containing protein, putative	0.028	0.718	0.188	0.008	0.058	0.428	0.398	-0.602	-0.262	-0.652	-0.182	0.008	-0.142	0.236	0.295	-0.505	-0.105	-0.341	0.790
PF3D7_130860(	PF3D7_1308600::conserved Plasmodium protein, unknown function	0.114	0.324	0.024	0.004	0.054	0.324	-0.186	-0.166	-0.116	0.024	-0.186	-0.116	-0.096	0.116	0.064	-0.086	-0.133	-0.249	0.841
PF3D7_134620(	PF3D7_1346200::nuclear import protein MOG1, putative	0.270	1.430	-0.270	0.210	0.020	0.720	-0.010	-0.610	-0.390	0.700	-0.870	-0.570	-0.630	0.410	0.243	-0.100	-0.690	-1.100	0.467
PF3D7_112290(	PF3D7_1122900::dynein heavy chain, putative	0.401	0.121	0.011	0.011	0.061	0.121	0.281	-0.169	-0.109	0.041	-0.249	-0.199	-0.319	0.136	0.154	-0.079	-0.256	-0.392	0.762
PF3D7_114470(	PF3D7_1144700::apicoplast import protein Tic20, putative	0.082	-0.078	-0.038	0.002	0.152	0.542	0.282	-0.178	-0.068	0.352	-0.488	-0.178	-0.378	-0.008	0.325	0.035	-0.348	-0.340	0.790
PF3D7_121040(	PF3D7_1210400::general transcription factor 3C polypeptide 5, putative	0.329	0.029	0.039	0.089	-0.001	0.469	-0.461	0.029	-0.031	0.029	-0.241	-0.041	-0.241	0.122	0.003	0.009	-0.174	-0.296	0.815
PF3D7_140440(	PF3D7_1404400::ribosomal protein L16, mitochondrial, putative	0.339	0.009	0.159	-0.001	-0.291	0.139	0.079	-0.031	-0.141	-0.021	-0.071	-0.111	-0.061	0.127	-0.024	-0.064	-0.081	-0.208	0.866

Shown are the mean-centered log_2 ratios of each sample against a 3D7 reference pool, the averaged log_2 ratios for each parasite line grouped by PfCRT haplotype, and the fold change for $Dd2^{F145Icrt}$ vs Dd2 lines.

Gene	Description	Dd2 repA	Dd2 repB	$Dd2^{Dd2crt}$ repA	$Dd2^{Dd2crt}$ repB	$Dd2^{M343Lcrt}$ repA	$Dd2^{M343Lcrt}$ repB	$Dd2^{M343Lcrt}$ repC	$Dd2^{G353Vcrt}$ repC	$Dd2^{G353Vcrt}$ repB	$Dd2^{G353Vcrt}$ repA	$Dd2^{F145Icrt}$ repA	$Dd2^{F145Icrt}$ repB	$Dd2^{F145Icrt}$ repC	Averaged Dd2	Averaged $Dd2^{M343Lcrt}$	Averaged $Dd2^{G353Vcrt}$	Averaged $Dd2^{F145Icrt}$	Log_2 fold change of $Dd2^{G353Vcrt}$/ $Dd2^{Dd2crt}$	Fold change of $Dd2^{G353Vcrt}$/ $Dd2^{Dd2crt}$
PF3D7_031190(	PF3D7_0311900::heptatricopeptide repeat-containing protein, putative	0.006	-0.064	0.026	-0.054	0.226	0.306	0.036	0.106	0.246	0.386	-0.534	-0.744	0.056	-0.021	0.189	0.246	-0.407	0.268	1.204
PF3D7_072390(	PF3D7_0723900::RNA-binding protein, putative	-0.183	-0.233	-0.013	-0.003	0.017	0.207	0.107	0.147	0.207	0.277	0.017	-0.413	-0.133	-0.108	0.110	0.210	-0.176	0.318	1.247
PF3D7_082500(	PF3D7_0825000::conserved Plasmodium protein, unknown function	-0.198	-0.228	-0.018	-0.048	-0.018	0.232	0.222	0.062	0.092	0.082	0.032	-0.268	0.052	-0.123	0.146	0.079	-0.061	0.202	1.150
PF3D7_123920(	PF3D7_1239200::AP2 domain transcription factor, putative	-0.255	-0.335	0.015	0.025	0.015	0.355	0.195	0.165	0.185	0.075	0.155	-0.715	0.115	-0.137	0.189	0.142	-0.148	0.279	1.213
PF3D7_090490(	PF3D7_0904900::copper-transporting ATPase	-0.046	-0.026	0.044	-0.006	-0.126	0.354	0.184	0.114	0.184	0.144	0.004	-0.696	-0.126	-0.009	0.137	0.147	-0.273	0.156	1.114
PF3D7_113320(	PF3D7_1133200::conserved Plasmodium protein, unknown function	0.016	0.006	-0.064	-0.024	-0.074	0.366	0.336	0.086	0.036	0.066	-0.014	-0.564	-0.174	-0.016	0.209	0.063	-0.251	0.079	1.056
PF3D7_114240(	PF3D7_1142400::coproporphyrinogen-III oxidase	0.023	0.013	0.013	0.023	0.073	0.353	0.283	0.003	-0.107	-0.137	-0.207	-0.347	0.013	0.018	0.236	-0.080	-0.180	-0.098	0.934
PF3D7_011280(	PF3D7_0112800::Plasmodium exported protein (hyp11), unknown functi	-0.445	-0.245	-0.015	-0.005	0.175	-0.415	0.705	0.315	0.495	0.855	-0.305	-0.395	-0.725	-0.177	0.155	0.555	-0.475	0.733	1.662
PF3D7_040430(	PF3D7_0404300::conserved Plasmodium protein, unknown function	-0.196	-0.366	-0.016	-0.046	-0.096	0.124	0.134	0.154	0.134	0.194	0.134	-0.246	0.094	-0.156	0.054	0.161	-0.006	0.317	1.245
PF3D7_100420(	PF3D7_1004200::WD repeat-containing protein, putative	-0.305	-0.455	0.025	0.025	0.055	0.025	0.225	0.115	0.255	0.085	0.155	-0.315	0.115	-0.178	0.101	0.151	-0.015	0.329	1.256
PF3D7_101930(	PF3D7_1019300::zinc finger protein, putative	-0.339	-0.569	-0.089	-0.169	0.011	-0.079	0.241	0.051	0.311	0.261	0.221	-0.089	0.241	-0.292	0.057	0.207	0.124	0.499	1.413
PF3D7_120840(	PF3D7_1208400::amino acid transporter, putative	-0.358	-0.298	0.002	-0.048	-0.248	-0.138	0.162	0.172	0.272	0.352	0.352	-0.268	0.042	-0.175	-0.074	0.266	0.042	0.441	1.357
PF3D7_032170(	PF3D7_0321700::conserved Plasmodium protein, unknown function	-0.495	-0.265	-0.125	-0.075	-0.195	-0.045	0.005	0.145	0.365	0.395	0.245	-0.005	0.055	-0.240	-0.079	0.301	0.098	0.542	1.456
PF3D7_050460(	PF3D7_0504600::2-oxoisovalerate dehydrogenase subunit beta, mitoch	-0.803	-0.753	-0.103	-0.033	-0.343	-0.123	0.067	0.127	0.477	0.387	0.467	0.157	0.477	-0.423	-0.133	0.330	0.367	0.753	1.686
PF3D7_092460(	PF3D7_0924600::conserved Plasmodium protein, unknown function	-0.872	-0.292	-0.242	-0.112	-0.482	-0.482	0.148	0.168	0.518	0.648	0.268	0.238	0.258	-0.380	-0.192	0.444	0.254	0.824	1.771
PF3D7_091980(	PF3D7_0919800::TLD domain-containing protein	-0.601	-0.121	-0.061	-0.081	-0.161	-0.291	-0.021	-0.001	0.249	0.599	0.199	0.009	0.279	-0.216	-0.157	0.283	0.163	0.498	1.413
PF3D7_111790(	PF3D7_1117900::conserved Plasmodium protein, unknown function	-0.489	-0.069	-0.009	-0.099	-0.229	-0.289	-0.019	0.061	0.231	0.601	0.111	0.051	0.151	-0.167	-0.179	0.297	0.104	0.464	1.380
PF3D7_021690(	PF3D7_0216900::conserved Plasmodium protein, unknown function	-0.653	-0.743	-0.073	-0.063	-0.183	-0.413	0.237	0.017	0.387	0.747	0.207	0.097	0.437	-0.383	-0.120	0.384	0.247	0.767	1.701
PF3D7_142640(	PF3D7_1426400::MORN repeat protein, putative	-0.584	-0.814	-0.124	-0.084	-0.594	-0.554	-0.004	0.046	0.686	1.246	0.386	0.196	0.196	-0.401	-0.384	0.659	0.259	1.061	2.086
PF3D7_050320(	PF3D7_0503200::cell division control protein 6, putative	-1.118	-0.488	0.192	0.182	-0.018	-0.468	0.172	0.352	0.292	0.632	0.302	-0.238	0.202	-0.308	-0.104	0.426	0.089	0.733	1.662
PF3D7_041990(	PF3D7_0419900::phosphatidylinositol 4-kinase, putative	-0.602	-0.222	-0.202	-0.032	0.078	-0.142	0.138	0.198	0.178	0.258	0.208	-0.052	0.198	-0.265	0.024	0.211	0.118	0.476	1.391
PF3D7_144850(	PF3D7_1448500::conserved Plasmodium protein, unknown function	-0.523	-0.163	-0.103	-0.013	-0.033	-0.003	0.127	0.137	0.207	0.207	0.117	-0.053	0.097	-0.201	0.030	0.184	0.054	0.384	1.305
PF3D7_142630(	PF3D7_1426300::dynein intermediate chain, putative	-1.292	-0.392	-0.142	0.098	-0.292	-0.482	0.448	0.318	0.608	0.398	0.398	0.008	0.318	-0.432	-0.108	0.442	0.242	0.873	1.832
PF3D7_031360(	PF3D7_0313600::conserved Plasmodium protein, unknown function	-0.498	-0.318	0.212	0.152	-0.268	-0.038	0.282	0.422	0.452	0.422	-0.028	-0.528	-0.268	-0.113	-0.008	0.432	-0.274	0.545	1.459
PF3D7_030640(	PF3D7_0306400::FAD-dependent glycerol-3-phosphate dehydrogenase	-0.617	-0.067	-0.167	0.063	0.013	0.133	0.303	0.163	0.263	0.293	-0.037	-0.497	0.153	-0.197	0.150	0.240	-0.127	0.437	1.353
PF3D7_040900(	PF3D7_0409000::conserved Plasmodium protein, unknown function	-0.622	0.008	-0.282	0.028	-0.122	0.138	0.308	0.258	0.138	0.358	0.168	-0.482	0.108	-0.217	0.108	0.251	-0.069	0.468	1.384
PF3D7_072810(	PF3D7_0728100::anaphase-promoting complex subunit 1, putative	-0.882	-0.292	-0.062	0.088	0.058	0.068	0.198	0.338	0.258	0.418	0.208	-0.492	0.098	-0.287	0.108	0.338	-0.062	0.625	1.542
PF3D7_143380(	PF3D7_1433800::conserved Plasmodium protein, unknown function	-0.675	-0.315	-0.055	0.065	-0.005	0.005	0.305	0.295	0.235	0.265	0.215	-0.395	0.065	-0.245	0.101	0.265	-0.039	0.510	1.424
PF3D7_010690(	PF3D7_0106900::2-C-methyl-D-erythritol 4-phosphate cytidylyltransferase	-0.499	-0.029	-0.059	0.051	-0.269	0.261	0.191	0.161	0.211	0.751	-0.119	-0.549	-0.099	-0.154	0.061	0.374	-0.256	0.508	1.422
PF3D7_072500(	PF3D7_0725000::exonuclease I, putative	-0.220	0.010	0.010	0.000	-0.170	0.080	0.110	0.020	0.090	0.360	0.050	-0.260	-0.080	-0.050	0.007	0.157	-0.097	0.207	1.154
PF3D7_131940(	PF3D7_1319400::conserved protein, unknown function	-0.202	-0.062	-0.012	-0.062	-0.182	0.168	0.158	0.098	0.088	0.378	0.058	-0.432	0.008	-0.085	0.048	0.188	-0.122	0.273	1.208
PF3D7_133970(	PF3D7_1339700::conserved Plasmodium protein, unknown function	-0.195	-0.065	-0.125	-0.055	-0.165	0.195	0.285	0.055	0.175	0.345	0.065	-0.455	-0.055	-0.110	0.105	0.191	-0.149	0.302	1.233
PF3D7_134340(	PF3D7_1343400::DNA repair protein RAD5, putative	-0.252	-0.232	-0.032	-0.122	-0.192	0.258	0.108	0.188	0.318	0.678	-0.082	-0.702	0.058	-0.159	0.058	0.395	-0.242	0.554	1.468
PF3D7_071020(	PF3D7_0710200::conserved Plasmodium protein, unknown function	-0.188	0.032	0.022	0.002	-0.078	0.022	0.142	0.102	0.052	0.192	0.072	-0.338	-0.038	-0.033	0.029	0.116	-0.101	0.148	1.108
PF3D7_145740(	PF3D7_1457400::conserved Plasmodium protein, unknown function	-0.582	0.068	-0.162	0.128	-0.152	0.048	0.218	0.318	0.168	0.568	0.008	-0.702	0.078	-0.137	0.038	0.351	-0.206	0.488	1.403
PF3D7_060750(	PF3D7_0607500::4-hydroxybenzoate polyprenyltransferase, putative	-0.415	-0.315	-0.065	0.045	0.025	-0.165	-0.065	0.225	0.285	0.495	0.025	-0.335	0.265	-0.188	-0.069	0.335	-0.015	0.523	1.436
PF3D7_082020(	PF3D7_0820200::phosphatidylglycerophosphate synthase	-0.370	0.030	-0.060	-0.040	0.030	-0.110	0.140	0.110	0.110	0.350	0.010	-0.350	0.150	-0.110	0.020	0.190	-0.063	0.300	1.231
PF3D7_081320(	PF3D7_0813200::CS domain protein, putative	-0.316	0.074	0.104	0.014	-0.126	-0.226	0.074	0.144	0.194	0.394	0.024	-0.556	0.204	-0.031	-0.093	0.244	-0.109	0.275	1.210
PF3D7_122750(	PF3D7_1227500::protein SOC2, putative	-0.439	-0.079	0.101	0.081	-0.079	-0.169	-0.039	0.191	0.221	0.401	0.131	-0.479	0.161	-0.084	-0.096	0.271	-0.063	0.355	1.279

PF3D7_020840(	PF3D7_0208400::conserved Plasmodium protein, unknown function	-0.585	0.075	-0.165	-0.055	-0.125	-0.105	0.075	0.185	0.275	0.515	0.055	-0.275	0.135	-0.183	-0.052	0.325	-0.029	0.508	1.422
PF3D7_130370(	PF3D7_1303700::tetratricopeptide repeat protein, putative	-0.992	0.048	-0.192	0.088	-0.212	-0.402	0.148	0.158	0.388	0.768	0.198	-0.142	0.138	-0.262	-0.155	0.438	0.065	0.700	1.625
PF3D7_145810(	PF3D7_1458100::protein PET117, putative	-0.885	0.145	-0.035	0.015	-0.055	-0.365	0.085	0.195	0.395	0.985	0.105	-0.485	-0.105	-0.190	-0.111	0.525	-0.161	0.715	1.641
PF3D7_143390(	PF3D7_1433900::protein kinase, putative	-0.848	-0.058	-0.268	-0.068	-0.158	0.032	0.302	0.072	0.212	0.692	0.312	-0.188	-0.028	-0.311	0.058	0.325	0.032	0.636	1.554
PF3D7_123630(	PF3D7_1236300::conserved protein, unknown function	-0.666	0.004	-0.116	-0.006	-0.046	-0.066	-0.156	0.034	0.164	0.724	0.024	-0.246	0.354	-0.196	-0.089	0.307	0.044	0.503	1.417
PF3D7_131210(	PF3D7_1312100::GYF domain-containing protein, putative	-0.212	-0.012	-0.142	-0.052	-0.272	-0.002	0.188	0.058	0.238	0.248	0.018	-0.142	0.088	-0.105	-0.029	0.181	-0.012	0.286	1.219
PF3D7_132590(	PF3D7_1325900::conserved Plasmodium protein, unknown function	-0.327	-0.097	-0.307	-0.077	-0.197	0.253	0.333	0.073	0.153	0.213	0.193	-0.187	-0.027	-0.202	0.130	0.146	-0.007	0.348	1.273
PF3D7_092650(	PF3D7_0926500::tetratricopeptide repeat protein, putative	-0.155	-0.395	-0.235	-0.255	0.045	0.255	0.315	0.045	0.275	-0.065	0.205	-0.225	0.185	-0.260	0.205	0.085	0.055	0.345	1.270
PF3D7_133040(	PF3D7_1330400::ER lumen protein retaining receptor 1, putative	-0.220	-0.170	-0.530	-0.140	0.180	0.380	0.000	-0.040	0.290	0.150	-0.060	0.120	0.040	-0.265	0.187	0.133	0.033	0.398	1.318
PF3D7_040440(	PF3D7_0404400::6-cysteine protein P36	0.005	-0.755	0.035	-0.075	-0.015	-0.415	-0.205	0.185	0.155	0.565	-0.005	0.145	0.375	-0.197	-0.211	0.302	0.172	0.499	1.413
PF3D7_091000(	PF3D7_0910000::histone-lysine N-methyltransferase SET4	-0.042	-0.182	-0.012	-0.142	-0.092	-0.382	-0.312	0.088	0.258	0.388	-0.072	0.428	0.078	-0.095	-0.262	0.244	0.144	0.339	1.265
PF3D7_091720(	PF3D7_0917200::conserved protein, unknown function	-0.243	-0.373	-0.153	-0.153	-0.133	-1.333	-0.603	0.247	0.427	0.627	0.207	0.677	0.807	-0.231	-0.690	0.434	0.564	0.664	1.585
PF3D7_052420(	PF3D7_0524200::conserved Plasmodium membrane protein, unknown fu	-0.272	-1.112	-0.262	-0.342	0.008	-0.432	-0.202	-0.112	0.268	0.818	0.488	0.958	0.198	-0.497	-0.209	0.324	0.548	0.822	1.767
PF3D7_142290(	PF3D7_1422900::14-3-3 protein, putative	-0.154	-0.314	-0.214	-0.144	-0.444	-0.264	-0.434	-0.004	0.256	0.876	0.176	0.766	-0.104	-0.206	-0.381	0.376	0.279	0.583	1.497
PF3D7_050820(	PF3D7_0508200::longevity-assurance (LAG1) protein, putative	-0.082	-0.112	-0.242	-0.302	-0.122	-0.082	-0.082	-0.012	0.208	0.538	-0.082	0.128	0.248	-0.185	-0.096	0.244	0.098	0.429	1.346
PF3D7_114100(	PF3D7_1141000::conserved Plasmodium protein, unknown function	-0.152	-0.382	-0.412	-0.212	-0.312	-0.882	-0.212	0.258	0.458	1.418	-0.552	0.168	0.808	-0.289	-0.468	0.712	0.142	1.001	2.001
PF3D7_093130(	PF3D7_0931300::conserved Plasmodium protein, unknown function	-0.229	-0.399	-0.179	-0.469	-0.489	-0.469	0.101	0.071	0.491	1.481	0.161	-0.009	-0.059	-0.319	-0.286	0.681	0.031	1.000	2.000
PF3D7_131310(	PF3D7_1313100::conserved Plasmodium protein, unknown function	-0.295	-0.175	-0.235	-0.335	-0.025	-0.435	-0.045	0.005	0.175	1.325	0.015	-0.085	0.115	-0.260	-0.169	0.501	0.015	0.762	1.695
PF3D7_050450(	PF3D7_0504500::MOLO1 domain-containing protein, putative	-1.130	0.010	-0.520	0.040	-0.450	-0.100	-0.550	0.260	0.950	0.180	0.430	0.840	0.040	-0.400	-0.367	0.463	0.437	0.863	1.819
PF3D7_061220(	PF3D7_0612200::leucine-rich repeat protein	-0.123	-0.413	-0.223	-0.123	-0.283	-0.153	0.137	0.007	0.257	0.207	0.377	0.027	0.307	-0.221	-0.100	0.157	0.237	0.378	1.299
PF3D7_021680(	PF3D7_0216800::TMEM121 domain-containing protein, putative	-0.310	-0.570	-0.390	-0.150	-0.040	-0.100	0.150	0.020	0.280	0.220	0.150	0.400	0.340	-0.355	0.003	0.173	0.297	0.528	1.442
PF3D7_080630(	PF3D7_0806300::ferlin-like protein, putative	-0.350	-0.680	-0.330	-0.080	-0.180	-0.190	0.210	0.130	0.210	0.270	0.300	0.260	0.430	-0.360	-0.053	0.203	0.330	0.563	1.478
PF3D7_092790(	PF3D7_0927900::phosphatidylserine decarboxylase	-0.662	-0.982	-0.452	-0.242	-0.292	-0.032	0.208	-0.012	0.258	0.718	0.228	0.488	0.778	-0.585	-0.039	0.321	0.498	0.906	1.874
PF3D7_070450(	PF3D7_0704500::serine/threonine protein kinase, putative	-0.534	-0.494	-0.544	-0.234	-0.034	-0.194	0.286	0.066	0.476	0.196	0.336	0.326	0.346	-0.451	0.019	0.246	0.336	0.698	1.622
PF3D7_135990(	PF3D7_1359900::conserved Plasmodium membrane protein, unknown fu	-0.227	-0.237	-0.127	-0.057	-0.117	-0.077	0.103	0.033	0.073	0.153	0.233	0.203	0.043	-0.162	-0.030	0.086	0.160	0.248	1.188
PF3D7_112020(	PF3D7_1120200::conserved Plasmodium protein, unknown function	-0.406	-0.516	-0.806	-0.336	-0.126	0.364	0.144	-0.106	0.244	0.204	0.054	0.774	0.514	-0.516	0.127	0.114	0.447	0.630	1.548
PF3D7_135050(	PF3D7_1350500::conserved Plasmodium protein, unknown function	-0.137	-0.197	-0.437	-0.257	0.023	-0.107	0.053	-0.067	0.113	0.083	0.063	0.503	0.363	-0.257	-0.010	0.043	0.310	0.300	1.231
PF3D7_010380(	PF3D7_0103800::actin-related protein ARP1, putative	-0.241	-0.401	-0.281	-0.151	-0.161	-0.361	-0.071	-0.031	0.459	0.519	0.099	0.439	0.179	-0.268	-0.197	0.316	0.239	0.584	1.499
PF3D7_122410(	PF3D7_1224100::conserved protein, unknown function	-0.455	-0.185	-0.395	-0.175	-0.405	-0.615	0.015	-0.005	0.695	0.715	0.235	0.195	0.385	-0.303	-0.335	0.468	0.271	0.771	1.706
PF3D7_050890(	PF3D7_0508900::protein AAP6	-0.555	-0.285	-0.385	-0.225	0.005	-0.515	-0.085	0.035	0.465	0.165	0.455	0.505	0.415	-0.362	-0.198	0.222	0.459	0.584	1.499
PF3D7_070410(	PF3D7_0704100::basal complex transmembrane protein 2	-0.542	-0.192	-0.462	-0.182	-0.072	-0.542	-0.032	0.068	0.408	0.188	0.398	0.488	0.478	-0.345	-0.216	0.221	0.454	0.566	1.480
PF3D7_144130(	PF3D7_1441300::serine/threonine protein kinase, putative	-0.268	-0.398	-0.318	-0.238	-0.098	-0.398	0.062	-0.018	0.372	0.232	0.312	0.382	0.372	-0.305	-0.144	0.196	0.356	0.501	1.415
PF3D7_112260(	PF3D7_1122600::conserved Plasmodium protein, unknown function	-0.513	-0.133	-0.303	-0.243	-0.233	-0.293	-0.083	0.027	0.437	0.287	0.277	0.507	0.267	-0.298	-0.203	0.250	0.350	0.548	1.462
PF3D7_032110(	PF3D7_0321100::conserved Plasmodium protein, unknown function	-0.764	-0.254	-0.274	-0.134	0.016	-0.794	-0.274	0.086	0.336	0.556	0.446	0.476	0.576	-0.356	-0.351	0.326	0.499	0.683	1.605
PF3D7_101960(	PF3D7_1019600::conserved Plasmodium protein, unknown function	-0.527	-0.267	-0.167	-0.087	-0.107	-0.477	-0.177	-0.007	0.183	0.243	0.463	0.483	0.443	-0.262	-0.254	0.140	0.463	0.402	1.321
PF3D7_111480(	PF3D7_1114800::glycerol-3-phosphate dehydrogenase, putative	-0.659	-0.259	-0.199	-0.189	-0.199	-0.789	-0.649	-0.089	0.501	0.391	0.541	0.851	0.751	-0.327	-0.546	0.267	0.714	0.594	1.510
PF3D7_061410(	PF3D7_0614100::filamin domain-containing protein, putative	-0.778	-0.138	-0.318	-0.138	-0.208	-0.768	-0.368	-0.048	0.342	0.892	0.472	0.592	0.462	-0.343	-0.448	0.396	0.509	0.738	1.668
PF3D7_123800(	PF3D7_1238000::COPI associated protein, putative	-0.597	-0.157	-0.447	-0.167	-0.337	-0.937	-0.257	-0.027	0.543	0.943	0.313	0.653	0.473	-0.342	-0.510	0.486	0.480	0.828	1.776
PF3D7_124390(	PF3D7_1243900::double C2-like domain-containing protein	-0.525	-0.595	-0.295	-0.175	-0.145	-0.675	-0.125	-0.025	0.325	0.655	0.375	0.655	0.555	-0.398	-0.315	0.318	0.528	0.716	1.642
PF3D7_132750(	PF3D7_1327500::conserved protein, unknown function	-0.316	-0.206	-0.386	-0.206	-0.206	-0.616	0.004	-0.106	0.244	0.574	0.204	0.584	0.434	-0.279	-0.273	0.237	0.407	0.516	1.430
PF3D7_052240(	PF3D7_0522400::conserved Plasmodium protein, unknown function	-0.477	-0.387	-0.507	-0.397	0.143	-0.997	-0.347	-0.217	0.303	0.013	0.773	1.243	0.853	-0.442	-0.400	0.033	0.956	0.475	1.390
PF3D7_134380(	PF3D7_1343800::conserved Plasmodium protein, unknown function	-0.585	-0.305	-0.595	-0.345	0.175	-0.915	-0.355	-0.135	0.075	-0.085	0.815	1.395	0.865	-0.458	-0.365	-0.049	1.025	0.409	1.328
PF3D7_135970(	PF3D7_1359700::conserved Plasmodium protein, unknown function	-0.366	-0.186	-0.466	-0.286	-0.006	-0.426	-0.046	-0.016	0.264	0.084	0.304	0.704	0.444	-0.326	-0.159	0.111	0.484	0.437	1.353
PF3D7_134930(	PF3D7_1349300::tyrosine kinase-like protein	-0.083	-0.093	-0.113	-0.203	-0.073	-0.233	-0.033	0.067	0.187	-0.043	0.197	0.247	0.177	-0.123	-0.113	0.070	0.207	0.193	1.143
PF3D7_124890(	PF3D7_1248900::26S protease regulatory subunit 8, putative	-0.322	-0.022	-0.052	-0.022	-0.022	-0.152	-0.012	0.008	0.018	-0.022	0.178	0.208	0.218	-0.105	-0.062	0.001	0.201	0.106	1.076
PF3D7_135610(	PF3D7_1356100::conserved Plasmodium protein, unknown function	-0.506	-0.116	-0.136	-0.056	-0.086	-0.166	-0.116	-0.026	0.174	0.064	0.274	0.404	0.294	-0.204	-0.123	0.071	0.324	0.274	1.209
PF3D7_145050(	PF3D7_1450500::conserved Plasmodium protein, unknown function	-0.570	-0.510	-0.130	-0.170	0.060	-0.220	-0.100	-0.030	0.190	0.010	0.420	0.580	0.470	-0.345	-0.087	0.057	0.490	0.402	1.321
PF3D7_062430(	PF3D7_0624300::CPW-WPC family protein	-0.026	-0.276	0.054	0.024	-0.246	0.014	-0.006	0.074	0.124	0.124	0.024	0.234	-0.116	-0.056	-0.079	0.107	0.047	0.163	1.120
PF3D7_120370(	PF3D7_1203700::nucleosome assembly protein	-0.042	-0.392	-0.032	-0.022	-0.112	0.128	-0.002	-0.002	0.038	0.318	-0.312	0.398	0.038	-0.122	0.004	0.118	0.041	0.240	1.181
PF3D7_031800(	PF3D7_0318000::conserved Plasmodium protein, unknown function	-0.617	-0.347	0.173	0.103	-0.597	-0.077	0.303	0.253	0.553	0.783	-0.327	0.113	-0.317	-0.172	-0.124	0.530	-0.177	0.702	1.626
PF3D7_135480(	PF3D7_1354800::metacaspase-1	-0.405	-0.275	-0.255	-0.035	-0.125	0.115	0.135	0.145	0.395	0.385	-0.225	0.245	-0.105	-0.242	0.042	0.309	-0.028	0.551	1.465
PF3D7_021700(	PF3D7_0217000::conserved Plasmodium membrane protein, unknown fu	-0.418	-0.058	-0.008	-0.008	-0.478	-0.048	-0.278	0.072	0.382	0.672	-0.148	-0.008	0.332	-0.123	-0.268	0.375	0.058	0.498	1.413
PF3D7_131960(	PF3D7_1319600::ACDC domain-containing protein, putative	-0.442	-0.202	-0.222	-0.132	-0.402	-0.042	0.148	-0.062	0.548	0.368	0.218	0.238	-0.012	-0.250	-0.099	0.284	0.148	0.534	1.448
PF3D7_131110(	PF3D7_1311100::meiosis-specific nuclear structural protein 1, putative	-0.239	-0.069	-0.049	-0.079	-0.839	-0.209	-0.209	-0.049	0.691	1.181	-0.109	0.201	-0.219	-0.109	-0.419	0.607	-0.043	0.717	1.643
PF3D7_140380(	PF3D7_1403800::nuclear formin-like protein MISFIT, putative	-0.248	-0.088	-0.148	-0.078	-0.518	-0.298	-0.058	-0.078	0.382	0.532	0.212	0.372	0.012	-0.140	-0.291	0.279	0.199	0.419	1.337
PF3D7_142560(	PF3D7_1425600::zinc finger protein, putative	-0.292	-0.102	-0.082	-0.002	-0.602	-0.262	0.178	0.088	0.328	0.568	0.148	0.148	-0.122	-0.119	-0.228	0.328	0.058	0.448	1.364
PF3D7_011150(	PF3D7_0111500::UMP-CMP kinase, putative	0.302	0.192	0.012	-0.018	0.132	0.062	-0.208	-0.298	-0.248	-0.438	-0.018	0.312	0.222	0.122	-0.005	-0.328	0.172	-0.450	0.732
PF3D7_121620(	PF3D7_1216200::glycerol-3-phosphate dehydrogenase [NAD(+)], putativ	0.899	0.289	-0.081	-0.091	0.249	0.209	-0.421	-0.201	-0.611	-0.671	-0.351	1.039	-0.261	0.254	0.013	-0.494	0.143	-0.748	0.595
PF3D7_032350(	PF3D7_0323500::survival motor neuron-like protein	0.222	-0.188	-0.038	-0.158	0.092	-0.368	-0.408	-0.328	-0.498	-0.218	0.162	1.572	0.162	-0.041	-0.228	-0.348	0.632	-0.308	0.808
PF3D7_041870(	PF3D7_0418700::RNA-binding protein NOB1, putative	0.389	-0.121	0.179	-0.211	0.089	-0.421	-0.521	-0.351	-0.911	-0.651	0.459	1.579	0.489	0.059	-0.284	-0.637	0.843	-0.697	0.617
PF3D7_021530(	PF3D7_0215300::acyl-CoA synthetase	0.775	0.005	-0.325	-0.355	-0.075	-0.355	-0.215	-0.355	-0.585	-0.425	0.445	1.515	-0.045	0.025	-0.215	-0.455	0.638	-0.480	0.717
PF3D7_040790(	PF3D7_0407900::AAA family ATPase, putative	0.674	-0.266	-0.286	-0.356	0.214	-0.126	-0.286	-0.406	-0.506	-0.686	0.074	1.764	0.194	-0.059	-0.066	-0.533	0.677	-0.474	0.720
PF3D7_101730(	PF3D7_1017300::golgi re-assembly stacking protein 1	0.380	-0.020	-0.190	-0.260	-0.050	-0.150	-0.240	-0.390	-0.340	-0.670	0.170	1.370	0.390	-0.023	-0.147	-0.467	0.643	-0.444	0.735
PF3D7_031610(	PF3D7_0316100::ribosomal protein L27, mitochondrial, putative	0.508	-0.042	-0.102	-0.062	0.178	0.038	-0.332	-0.502	-0.452	-0.472	-0.042	1.318	-0.032	0.075	-0.039	-0.476	0.414	-0.551	0.683
PF3D7_132760(	PF3D7_1327600::nicotinamide/nicotinic acid mononucleotide adenylyltra	0.312	-0.028	-0.198	-0.128	0.332	-0.008	-0.328	-0.358	-0.728	-0.788	0.222	1.732	-0.028	-0.011	-0.002	-0.625	0.642	-0.614	0.653
PF3D7_101410(	PF3D7_1014100::merozoite surface protein MSA180	1.055	0.135	-0.825	-0.755	0.655	-1.375	-1.135	-1.265	-1.065	-1.165	1.465	3.075	1.195	-0.097	-0.618	-1.165	1.912	-1.068	0.477
PF3D7_140470(	PF3D7_1404700::cysteine-rich small secreted protein CSS, putative	1.240	0.600	-0.650	-0.680	0.530	-0.930	-0.760	-1.400	-0.800	-1.120	0.750	2.490	0.730	0.128	-0.387	-1.107	1.323	-1.234	0.425
PF3D7_091060(	PF3D7_0910600::SNARE protein	-0.019	-0.039	0.121	-0.139	0.081	-0.829	-0.539	-0.389	-0.309	-0.359	0.421	1.361	0.641	-0.019	-0.429	-0.353	0.807	-0.333	0.794
PF3D7_062960(	PF3D7_0629600::ribosomal RNA-processing protein 7, putative	0.188	0.318	0.058	0.028	0.028	-0.192	-0.242	-0.192	-0.132	-0.132	-0.282	0.988	-0.432	0.148	-0.136	-0.152	0.091	-0.300	0.812
PF3D7_110580(	PF3D7_1105800::conserved Apicomplexan protein, unknown function	0.043	0.143	-0.007	0.113	-0.107	0.213	-0.197	-0.207	-0.227	-0.327	-0.007	0.673	-0.107	0.073	-0.030	-0.254	0.186	-0.327	0.797
PF3D7_136590(	PF3D7_1365900::ubiquitin-60S ribosomal protein L40	0.291	0.131	0.051	0.231	-0.019	-0.179	-0.289	-0.189	-0.139	-0.569	-0.289	0.891	0.081	0.176	-0.163	-0.299	0.227	-0.475	0.719
PF3D7_050620(	PF3D7_0506200::TATA-box-binding protein	0.228	0.028	0.088	0.188	-0.022	0.298	-0.272	-0.192	-0.102	-0.112	-0.272	-0.102	0.238	0.133	0.002	-0.135	-0.045	-0.268	0.830
PF3D7_062850(	PF3D7_0628500::conserved Plasmodium protein, unknown function	0.808	0.348	0.228	0.098	0.328	0.298	-0.292	-0.232	-0.752	-0.392	-0.202	-0.202	-0.042	0.371	0.112	-0.458	-0.148	-0.829	0.563
PF3D7_133380(	PF3D7_1333800::Voldacs domain-containing protein, putative	0.419	-0.071	0.209	0.159	0.289	0.359	-0.191	-0.401	-0.461	-0.421	-0.311	0.249	0.169	0.179	0.153	-0.427	0.036	-0.607	0.657
PF3D7_103760(	PF3D7_1037600::TFIIH basal transcription factor complex helicase XPB	0.175	0.315	-0.015	-0.005	-0.105	0.365	0.085	-0.085	-0.295	-0.495	-0.145	0.125	0.085	0.117	0.115	-0.292	0.021	-0.409	0.753
PF3D7_113160(	PF3D7_1131600::kelch domain-containing protein, putative	0.028	0.718	0.188	0.008	0.058	0.428	0.398	-0.602	-0.262	-0.652	0.008	-0.182	-0.142	0.236	0.295	-0.505	-0.105	-0.741	0.598
PF3D7_131340(	PF3D7_1313400::DEAD box helicase, putative	-0.015	0.345	-0.025	-0.035	-0.045	0.205	-0.095	-0.185	-0.045	-0.225	-0.285	0.175	0.235	0.067	0.021	-0.152	0.041	-0.219	0.859
PF3D7_070660(	PF3D7_0706600::conserved Plasmodium protein, unknown function	0.207	0.247	0.467	0.197	0.027	-0.133	-0.413	0.147	0.137	0.187	-0.533	0.037	-0.573	0.279	-0.173	0.157	-0.356	-0.123	0.919
PF3D7_113610(	PF3D7_1136100::conserved Plasmodium protein, unknown function	0.237	0.237	0.417	0.207	0.007	-0.003	0.017	-0.433	-0.123	0.107	-0.083	-0.213	-0.373	0.274	0.007	-0.150	-0.223	-0.424	0.745
PF3D7_052230(	PF3D7_0522300::18S rRNA (guanine-N(7))-methyltransferase, putative	0.228	0.408	0.748	0.188	0.068	-0.362	-0.242	0.038	-0.362	-0.162	-0.342	-0.342	0.128	0.393	-0.178	-0.162	-0.185	-0.555	0.681
PF3D7_133690(	PF3D7_1336900::tryptophan--tRNA ligase	0.267	0.287	0.217	0.237	0.087	-0.043	-0.283	0.147	-0.093	0.137	-0.093	-0.723	-0.143	0.252	-0.080	0.064	-0.320	-0.188	0.878
PF3D7_031160(	PF3D7_0311600::dolichyl-diphosphooligosaccharide--protein glycosyltrar	0.223	0.663	0.223	0.033	0.173	0.303	0.013	-0.277	-0.367	-0.057	-0.537	0.073	-0.467	0.286	0.163	-0.234	-0.310	-0.519	0.698
PF3D7_091400(	PF3D7_0914000::pseudouridylate synthase, putative	0.202	0.462	0.282	0.092	0.062	0.162	-0.138	-0.008	-0.548	-0.368	-0.308	0.422	-0.308	0.259	0.028	-0.308	-0.065	-0.568	0.675
PF3D7_111750(	PF3D7_1117500::tyrosine--tRNA ligase	0.160	0.210	0.150	0.090	0.040	0.210	-0.030	-0.060	-0.270	-0.160	-0.350	0.250	-0.240	0.183	0.073	-0.163	-0.113	-0.316	0.803
PF3D7_112160(	PF3D7_1121600::exported protein 1	0.585	0.595	0.475	0.205	0.025	0.365	-0.425	-0.135	-0.295	-0.815	-0.455	0.175	-0.305	0.465	-0.011	-0.415	-0.195	-0.880	0.543
PF3D7_133250(	PF3D7_1332500::SAM-dependent RNA methyltransferase, putative	0.189	0.179	0.159	0.369	0.089	0.349	-0.031	0.099	-0.131	-0.591	-0.551	0.119	-0.251	0.224	0.136	-0.207	-0.227	-0.432	0.742
PF3D7_100670(	PF3D7_1006700::conserved Plasmodium protein, unknown function	0.256	0.326	0.566	0.106	0.176	0.006	-0.124	-0.024	-0.344	-0.414	-0.364	0.066	-0.234	0.314	0.019	-0.261	-0.177	-0.574	0.672
PF3D7_101850(	PF3D7_1018500::pre-mRNA-splicing factor RDS3, putative	0.368	0.148	0.498	0.128	0.318	-0.072	-0.022	-0.072	-0.302	-0.232	-0.442	0.048	-0.362	0.285	0.074	-0.202	-0.252	-0.488	0.713
PF3D7_102900(	PF3D7_1029000::conserved Plasmodium protein, unknown function, pse	0.145	0.165	0.335	0.285	0.325	-0.015	-0.245	-0.075	-0.365	-0.165	-0.355	0.045	-0.075	0.232	0.021	-0.202	-0.129	-0.434	0.740
PF3D7_071870(	PF3D7_0718700::conserved Plasmodium membrane protein, unknown fu	0.258	0.338	0.348	0.458	-0.072	0.208	-0.062	0.058	-0.722	-0.192	-0.312	-0.042	-0.262	0.350	0.024	-0.286	-0.206	-0.636	0.644
PF3D7_120510(	PF3D7_1205100::O-phosphoseryl-tRNA(Sec) selenium transferase, puta	0.239	0.179	0.429	0.199	0.099	0.119	-0.151	-0.111	-0.191	0.119	-0.441	-0.211	-0.281	0.262	0.023	-0.061	-0.311	-0.323	0.800
PF3D7_142590(	PF3D7_1425900::conserved Plasmodium protein, unknown function	0.225	0.295	0.455	0.395	0.065	0.195	-0.085	0.075	-0.355	-0.045	-0.555	-0.085	-0.575	0.342	0.058	-0.109	-0.405	-0.451	0.732
PF3D7_042500(	PF3D7_0425000::Plasmodium exported protein, unknown function, pseu	1.559	1.849	1.389	0.899	0.159	-0.291	-0.991	-0.471	-0.241	-0.441	-1.061	-0.771	-1.591	1.424	-0.374	-0.384	-1.141	-1.808	0.286
PF3D7_060820(	PF3D7_0608200::conserved Plasmodium protein, unknown function	0.347	0.837	0.267	0.217	-0.063	-0.043	-0.123	-0.073	-0.093	-0.373	-0.303	-0.103	-0.493	0.417	-0.076	-0.180	-0.300	-0.597	0.661
PF3D7_072080(	PF3D7_0720800::Ham1-like protein, putative	0.086	0.586	0.186	0.186	-0.014	-0.094	-0.094	0.036	0.046	-0.044	-0.444	-0.294	-0.144	0.261	-0.067	0.013	-0.294	-0.248	0.842
PF3D7_030650(	PF3D7_0306500::cytochrome c oxidase subunit ApiCOX35, putative	0.196	0.456	0.056	0.016	-0.284	0.106	0.096	-0.064	-0.064	-0.254	-0.064	-0.124	-0.074	0.181	-0.027	-0.127	-0.087	-0.308	0.808
PF3D7_100750(	PF3D7_1007500::conserved Plasmodium protein, unknown function	0.278	0.868	0.118	0.118	0.038	0.138	0.138	-0.112	-0.062	-0.672	-0.232	-0.312	-0.302	0.345	0.104	-0.282	-0.282	-0.628	0.647
PF3D7_121630(	PF3D7_1216300::signal recognition particle subunit SRP19	0.180	0.970	0.120	0.060	-0.180	0.060	0.070	0.030	-0.070	-0.250	-0.230	-0.600	-0.160	0.333	-0.017	-0.097	-0.330	-0.429	0.743
PF3D7_052780(	PF3D7_0527800::tRNA Leucine	0.482	0.682	0.212	0.202	0.412	0.192	-0.338	-0.278	-0.128	-0.578	-0.118	-0.408	-0.338	0.395	0.089	-0.328	-0.288	-0.723	0.606

PF3D7_061990(	PF3D7_0619900::splicing factor 3A subunit 2, putative	0.410	0.500	0.080	0.100	-0.080	0.370	0.140	-0.290	-0.480	-0.340	-0.190	-0.080	-0.140	0.273	0.143	-0.370	-0.137	-0.643	0.641
PF3D7_114750(	PF3D7_1147500::protein farnesyltransferase subunit beta	0.532	0.702	0.102	0.222	-0.098	0.332	0.072	-0.128	-0.228	-0.668	-0.648	-0.188	0.002	0.389	0.102	-0.342	-0.278	-0.731	0.603
PF3D7_021080(	PF3D7_0210800::conserved Plasmodium protein, unknown function	0.822	0.722	0.052	0.012	-0.068	0.272	0.002	-0.268	-0.248	-0.308	-0.398	-0.058	-0.538	0.402	0.069	-0.274	-0.331	-0.677	0.626
PF3D7_113570(	PF3D7_1135700::conserved Plasmodium protein, unknown function	0.425	0.525	0.075	0.085	-0.005	0.195	0.015	-0.075	-0.115	-0.215	-0.305	-0.205	-0.405	0.278	0.069	-0.135	-0.305	-0.413	0.751
PF3D7_112650(	PF3D7_1126500::WD repeat-containing protein, putative	0.642	0.662	0.152	0.062	0.142	0.452	0.072	-0.078	-0.398	-0.598	-0.448	-0.138	-0.518	0.379	0.222	-0.358	-0.368	-0.738	0.600
PF3D7_137220(	PF3D7_1372200::histidine-rich protein III	1.792	0.992	0.742	0.292	-0.438	0.722	0.062	-0.118	-0.528	-1.148	-0.878	-0.448	-1.038	0.954	0.115	-0.598	-0.788	-1.553	0.341
PF3D7_090770(	PF3D7_0907700::proteasome activator 28	0.612	0.312	0.202	0.192	0.112	0.302	0.332	-0.048	-0.418	-1.168	-0.038	-0.248	-0.138	0.329	0.248	-0.545	-0.142	-0.874	0.546
PF3D7_080790(	PF3D7_0807900::tyrosine--tRNA ligase	0.157	0.127	0.127	0.207	0.327	0.327	-0.003	0.107	-0.183	-0.953	-0.053	-0.223	0.037	0.154	0.217	-0.343	-0.080	-0.498	0.708
PF3D7_103890(	PF3D7_1038900::esterase, putative	0.526	0.056	0.166	0.146	0.556	0.526	0.186	-0.084	-0.444	-1.294	0.286	-0.544	-0.084	0.224	0.423	-0.607	-0.114	-0.831	0.562
PF3D7_051060(	PF3D7_0510600::tRNA Leucine	0.642	0.242	0.282	0.882	0.542	0.192	0.002	-0.078	-0.498	-1.068	-0.278	-0.358	-0.508	0.512	0.246	-0.548	-0.381	-1.060	0.480
PF3D7_022070(	PF3D7_0220700::Plasmodium exported protein (hyp9), unknown functio	1.197	0.227	0.287	0.117	0.377	0.547	0.277	0.057	-0.503	-0.993	-0.433	-0.413	-0.743	0.457	0.400	-0.480	-0.530	-0.937	0.522
PF3D7_123020(	PF3D7_1230200::vacuolar transporter chaperone, putative, pseudogene	0.555	0.135	0.255	0.255	0.395	0.555	0.085	0.125	-0.615	-0.915	-0.295	-0.325	-0.215	0.300	0.345	-0.468	-0.278	-0.768	0.587
PF3D7_134490(	PF3D7_1344900::conserved Plasmodium protein, unknown function	0.281	0.161	0.221	0.131	0.131	0.211	0.051	0.121	-0.419	-0.339	-0.249	-0.219	-0.079	0.198	0.131	-0.213	-0.183	-0.411	0.752
PF3D7_041820(	PF3D7_0418200::eukaryotic translation initiation factor 3 subunit M, puta	0.587	0.247	0.097	0.077	0.327	0.277	0.087	-0.053	-0.123	-0.333	-0.273	-0.513	-0.403	0.252	0.230	-0.170	-0.396	-0.422	0.747
PF3D7_062180(	PF3D7_0621800::nascent polypeptide-associated complex subunit alpha	0.728	0.768	0.388	0.238	0.528	0.598	-0.052	0.028	-0.362	-0.672	-0.602	-0.902	-0.692	0.531	0.358	-0.335	-0.732	-0.866	0.549
PF3D7_030850(	PF3D7_0308500::activator of Hsp90 ATPase, putative	0.998	0.608	0.798	0.408	0.298	0.158	-0.122	-0.122	-0.292	-0.562	-0.592	-0.862	-0.722	0.703	0.112	-0.325	-0.725	-1.028	0.490
PF3D7_060210(	PF3D7_0602100::ATP-dependent RNA helicase MTR4	0.485	0.155	0.365	0.045	0.285	0.245	-0.015	-0.065	-0.275	-0.385	-0.325	-0.265	-0.255	0.263	0.172	-0.241	-0.281	-0.504	0.705
PF3D7_101020(	PF3D7_1010200::DNA2/NAM7 helicase, putative	0.478	0.238	0.368	0.168	0.138	0.318	0.028	0.098	-0.332	-0.322	-0.402	-0.362	-0.412	0.313	0.161	-0.186	-0.392	-0.498	0.708
PF3D7_101530(	PF3D7_1015300::methionine aminopeptidase 1b, putative	0.716	0.566	0.566	0.356	0.466	0.576	-0.044	0.296	-0.714	-0.874	-0.544	-0.774	-0.594	0.551	0.333	-0.431	-0.637	-0.982	0.506
PF3D7_112170(	PF3D7_1121700::protein GCN20	0.348	0.218	0.308	0.108	0.038	0.378	0.158	-0.042	-0.262	-0.232	-0.322	-0.452	-0.242	0.245	0.191	-0.179	-0.339	-0.424	0.745
PF3D7_130820(	PF3D7_1308200::carbamoyl phosphate synthetase	0.374	0.424	0.354	0.274	0.244	0.344	0.194	0.184	-0.386	-0.396	-0.436	-0.846	-0.326	0.356	0.261	-0.199	-0.536	-0.556	0.680
PF3D7_140790(	PF3D7_1407900::plasmepsin I	0.685	0.355	0.265	0.225	0.075	0.185	0.215	0.205	-0.285	-0.505	-0.455	-0.725	-0.245	0.383	0.159	-0.195	-0.475	-0.578	0.670
PF3D7_042240(	PF3D7_0422400::40S ribosomal protein S19	0.582	0.492	0.392	0.362	0.662	0.482	-0.008	0.022	-0.288	-1.558	-0.258	-0.548	-0.338	0.457	0.379	-0.608	-0.381	-1.065	0.478
PF3D7_081390(	PF3D7_0813900::40S ribosomal protein S16, putative	1.182	0.432	0.432	0.702	0.792	0.492	0.062	0.002	-0.348	-2.068	-0.548	-0.658	-0.468	0.687	0.448	-0.805	-0.558	-1.492	0.356
PF3D7_090390(	PF3D7_0903900::60S ribosomal protein L32	0.894	0.614	0.514	0.424	0.524	0.434	0.024	0.044	-0.296	-1.496	-0.526	-0.716	-0.436	0.611	0.327	-0.583	-0.559	-1.194	0.437
PF3D7_050380(	PF3D7_0503800::60S ribosomal protein L31	0.495	0.645	0.495	0.575	0.455	0.205	-0.015	0.075	-0.235	-1.235	-0.525	-0.585	-0.355	0.553	0.215	-0.465	-0.488	-1.018	0.494
PF3D7_021780(	PF3D7_0217800::40S ribosomal protein S26	0.429	0.519	0.409	0.779	0.589	0.369	-0.081	0.089	-0.101	-1.111	-0.491	-0.981	-0.421	0.534	0.293	-0.374	-0.631	-0.908	0.533
PF3D7_114400(	PF3D7_1144000::40S ribosomal protein S21	0.685	0.775	0.405	0.725	0.645	0.695	0.095	-0.025	-0.285	-1.545	-0.525	-0.975	-0.675	0.648	0.479	-0.618	-0.725	-1.266	0.416
PF3D7_100350(	PF3D7_1003500::40S ribosomal protein S20e, putative	0.311	0.431	0.321	0.521	0.621	0.491	-0.009	0.141	-0.279	-0.819	-0.709	-0.669	-0.349	0.396	0.367	-0.319	-0.576	-0.715	0.609
PF3D7_021400(	PF3D7_0214000::T-complex protein 1 subunit theta	0.532	0.482	0.772	0.502	0.602	0.552	0.032	0.212	-0.418	-1.108	-0.768	-0.888	-0.498	0.572	0.395	-0.438	-0.718	-1.010	0.497
PF3D7_120860(	PF3D7_1208600::mitochondrial import inner membrane translocase subu	0.479	0.469	0.399	0.369	0.339	0.269	0.029	0.079	-0.251	-0.711	-0.491	-0.571	-0.411	0.429	0.213	-0.294	-0.491	-0.723	0.606
PF3D7_140250(	PF3D7_1402500::ribosomal protein S27a, putative	0.635	0.785	0.595	0.735	0.705	0.485	0.035	0.235	-0.235	-1.165	-0.835	-1.215	-0.755	0.687	0.408	-0.389	-0.935	-1.076	0.474
PF3D7_062750(	PF3D7_0627500::protein DJ-1	0.526	0.436	0.486	0.546	0.786	0.566	-0.074	0.356	-0.244	-1.444	-0.774	-0.954	-0.214	0.499	0.426	-0.444	-0.647	-0.943	0.520
PF3D7_112600(	PF3D7_1126000::threonine--tRNA ligase	0.152	0.222	0.162	0.132	0.192	0.242	0.022	0.072	-0.208	-0.468	-0.228	-0.258	-0.038	0.167	0.152	-0.201	-0.174	-0.368	0.775
PF3D7_101470(	PF3D7_1014700::prohibitin 2, putative	0.419	0.329	0.579	0.409	0.659	0.849	-0.021	0.069	-0.331	-1.331	-0.681	-0.511	-0.441	0.434	0.496	-0.531	-0.544	-0.965	0.512
PF3D7_110310(	PF3D7_1103100::60S acidic ribosomal protein P1, putative	0.392	0.392	0.632	0.602	0.652	0.162	-0.228	0.012	-0.218	-1.048	-0.248	-0.658	-0.448	0.505	0.196	-0.418	-0.451	-0.923	0.528
PF3D7_133420(	PF3D7_1334200::chaperone binding protein, putative	0.387	0.387	0.527	0.497	0.687	0.457	-0.313	-0.003	-0.403	-0.643	-0.233	-0.963	-0.383	0.449	0.277	-0.350	-0.526	-0.799	0.575
PF3D7_050790(	PF3D7_0507900::conserved Plasmodium protein, unknown function	0.365	0.275	0.405	0.405	0.425	0.475	0.125	-0.025	-0.225	-0.695	-0.315	-0.665	-0.555	0.363	0.342	-0.315	-0.511	-0.678	0.625
PF3D7_051280(	PF3D7_0512800::conserved Plasmodium protein, unknown function	0.178	0.158	0.318	0.198	0.228	0.408	0.148	0.148	-0.152	-0.412	-0.342	-0.482	-0.392	0.213	0.261	-0.139	-0.406	-0.352	0.784
PF3D7_081360(	PF3D7_0813600::translation initiation factor SUI1, putative	0.500	0.300	0.710	0.360	0.370	0.640	0.350	0.170	-0.350	-0.800	-0.450	-1.150	-0.650	0.468	0.453	-0.327	-0.750	-0.794	0.577
PF3D7_032200(	PF3D7_0322000::peptidyl-prolyl cis-trans isomerase	0.298	0.328	0.588	0.438	0.538	0.398	-0.022	0.088	-0.272	-0.772	-0.302	-0.932	-0.372	0.413	0.304	-0.319	-0.536	-0.732	0.602
PF3D7_050710(	PF3D7_0507100::60S ribosomal protein L4	0.394	0.304	0.684	0.354	0.364	0.414	0.074	0.144	-0.176	-0.726	-0.346	-1.086	-0.396	0.434	0.284	-0.253	-0.609	-0.687	0.621
PF3D7_142120(	PF3D7_1421200::40S ribosomal protein S25	0.398	0.308	0.388	0.468	0.568	0.428	0.178	0.238	-0.232	-0.812	-0.472	-1.042	-0.422	0.391	0.392	-0.268	-0.645	-0.659	0.633
PF3D7_052440(	PF3D7_0524400::ribosome-interacting GTPase 1, putative	0.189	0.199	0.349	0.179	0.229	0.209	0.159	0.169	-0.181	-0.581	-0.251	-0.591	-0.081	0.229	0.199	-0.197	-0.307	-0.427	0.744
PF3D7_133290(	PF3D7_1332900::isoleucine--tRNA ligase, putative	0.237	0.287	0.447	0.267	0.417	0.487	0.127	0.227	-0.303	-0.723	-0.233	-1.073	-0.163	0.309	0.344	-0.266	-0.490	-0.576	0.671
PF3D7_122950(	PF3D7_1229500::T-complex protein 1 subunit gamma	0.263	0.303	0.773	0.363	0.363	0.233	0.043	0.183	-0.227	-0.707	-0.387	-0.757	-0.447	0.426	0.213	-0.250	-0.530	-0.676	0.626
PF3D7_125000(	PF3D7_1250000::rRNA-processing protein FCF2, putative	0.155	0.405	0.655	0.285	0.185	0.315	0.325	-0.025	-0.305	-0.685	-0.335	-0.605	-0.375	0.375	0.275	-0.338	-0.438	-0.713	0.610
PF3D7_060870(	PF3D7_0608700::T-complex protein 1 subunit zeta	0.279	0.559	0.699	0.269	0.369	0.289	-0.061	0.169	-0.291	-0.571	-0.441	-0.851	-0.421	0.452	0.199	-0.231	-0.571	-0.683	0.623
PF3D7_113220(	PF3D7_1132200::T-complex protein 1 subunit alpha	0.324	0.374	0.654	0.334	0.354	0.404	0.034	0.214	-0.276	-0.406	-0.546	-0.976	-0.486	0.421	0.264	-0.156	-0.669	-0.578	0.670
PF3D7_142410(	PF3D7_1424100::60S ribosomal protein L5, putative	0.418	0.548	0.588	0.308	0.258	0.428	-0.072	0.038	-0.112	-0.392	-0.522	-1.052	-0.432	0.465	0.204	-0.156	-0.669	-0.621	0.650
PF3D7_130540(	PF3D7_1305400::AAR2 protein, putative	0.420	0.300	0.480	0.420	0.140	0.390	0.050	0.050	-0.270	-0.470	-0.370	-0.650	-0.490	0.405	0.193	-0.230	-0.503	-0.635	0.644
PF3D7_102680(	PF3D7_1026800::40S ribosomal protein S2	0.237	0.437	0.527	0.447	0.217	0.327	-0.113	0.007	-0.143	-0.473	-0.473	-0.713	-0.283	0.412	0.144	-0.203	-0.490	-0.615	0.653
PF3D7_146590(	PF3D7_1465900::40S ribosomal protein S3	0.555	0.495	0.725	0.615	0.325	0.285	-0.055	0.035	-0.215	-0.745	-0.575	-0.895	-0.555	0.598	0.185	-0.308	-0.675	-0.906	0.534
PF3D7_145970(	PF3D7_1459700::pyridoxal 5'-phosphate synthase, putative	0.288	0.428	0.548	0.568	0.258	0.498	0.018	-0.052	-0.302	-0.302	-0.502	-0.962	-0.492	0.458	0.258	-0.218	-0.652	-0.677	0.626
PF3D7_091430(	PF3D7_0914300::met-10+ like protein, putative	0.481	0.431	0.241	0.281	0.091	0.541	-0.109	0.051	-0.249	-0.549	-0.359	-0.569	-0.279	0.358	0.174	-0.249	-0.403	-0.608	0.656
PF3D7_134720(	PF3D7_1347200::nucleoside transporter 1	0.455	0.565	0.355	0.385	0.485	0.865	-0.015	0.205	-0.365	-0.835	-0.655	-1.155	-0.285	0.440	0.445	-0.332	-0.699	-0.772	0.586
PF3D7_146770(	PF3D7_1467700::conserved Plasmodium protein, unknown function	0.310	0.460	0.280	0.260	0.210	0.340	0.070	0.020	0.130	-0.580	-0.590	-0.700	-0.210	0.328	0.207	-0.143	-0.500	-0.471	0.722
PF3D7_021010(	PF3D7_0210100::60S ribosomal protein L37ae, putative	0.390	0.760	0.565	0.300	0.180	0.030	-0.205	0.125	-0.220	-0.500	-0.280	-0.565	-0.575	0.503	0.001	-0.199	-0.474	-0.702	0.615
PF3D7_112000(	PF3D7_1120000::conserved protein, unknown function	0.202	0.512	0.432	0.162	0.152	0.032	-0.128	0.112	-0.248	-0.128	-0.278	-0.438	-0.388	0.327	0.019	-0.088	-0.368	-0.415	0.750
PF3D7_103820(	PF3D7_1038200::conserved Plasmodium protein, unknown function	0.206	0.946	0.526	0.306	0.216	-0.024	-0.164	-0.134	-0.244	-0.204	-0.304	-0.634	-0.494	0.496	0.009	-0.194	-0.477	-0.690	0.620
PF3D7_030960(	PF3D7_0309600::60S acidic ribosomal protein P2	0.273	0.593	0.393	0.453	0.193	0.193	-0.107	-0.057	-0.147	-0.327	-0.417	-0.577	-0.467	0.428	0.093	-0.177	-0.487	-0.605	0.657
PF3D7_100490(	PF3D7_1004900::conserved protein, unknown function	0.337	0.837	0.337	0.277	0.007	0.337	0.017	-0.063	-0.113	-0.533	-0.343	-0.623	-0.473	0.447	0.120	-0.236	-0.480	-0.683	0.623
PF3D7_103330(	PF3D7_1033300::conserved protein, unknown function	0.672	1.512	0.662	0.282	0.122	0.182	-0.128	0.012	-0.448	-0.588	-0.698	-0.898	-0.678	0.782	0.058	-0.342	-0.758	-1.123	0.459
PF3D7_142610(	PF3D7_1426100::transcription factor BTF3, putative	0.554	0.894	0.424	0.264	0.334	0.204	-0.056	0.014	-0.286	-0.576	-0.506	-0.776	-0.486	0.534	0.161	-0.283	-0.589	-0.817	0.568
PF3D7_143400(	PF3D7_1434000::CCR4-associated factor 16, putative	0.569	0.789	0.429	0.249	0.229	0.289	-0.111	-0.011	-0.311	-0.401	-0.451	-0.771	-0.501	0.509	0.136	-0.241	-0.574	-0.750	0.595
PF3D7_051700(	PF3D7_0517000::60S ribosomal protein L12, putative	0.462	0.572	0.412	0.432	0.302	0.222	0.052	0.322	-0.058	-0.818	-0.478	-0.948	-0.468	0.469	0.192	-0.185	-0.632	-0.654	0.635
PF3D7_132340(	PF3D7_1323400::60S ribosomal protein L23	0.495	0.735	0.455	0.505	0.345	0.365	0.105	0.205	-0.205	-0.885	-0.415	-1.065	-0.635	0.547	0.271	-0.295	-0.705	-0.843	0.558
PF3D7_144710(	PF3D7_1447100::conserved Plasmodium protein, unknown function	0.237	0.297	0.267	0.307	0.067	0.177	0.047	0.107	0.097	-0.453	-0.173	-0.603	-0.373	0.277	0.097	-0.083	-0.383	-0.360	0.779
PF3D7_131780(	PF3D7_1317800::40S ribosomal protein S19	0.405	1.005	0.525	0.515	0.375	0.095	-0.105	0.105	-0.245	-0.955	-0.475	-0.765	-0.485	0.613	0.122	-0.365	-0.575	-0.978	0.508
PF3D7_146070(	PF3D7_1460700::60S ribosomal protein L27	0.567	1.017	0.517	0.517	0.357	0.087	-0.213	0.027	-0.103	-0.903	-0.413	-1.063	-0.393	0.654	0.077	-0.326	-0.623	-0.981	0.507
PF3D7_144330(	PF3D7_1443300::U6 snRNA-associated Sm-like protein LSm5, putative	0.363	0.733	0.283	0.303	0.253	0.113	0.013	0.143	-0.027	-0.747	-0.267	-0.657	-0.507	0.421	0.126	-0.210	-0.477	-0.631	0.646
PF3D7_146130(	PF3D7_1461300::40S ribosomal protein S28e, putative	0.323	0.703	0.603	0.373	0.183	0.023	0.013	0.113	-0.177	-0.917	-0.217	-0.707	-0.317	0.501	0.073	-0.327	-0.414	-0.828	0.564
PF3D7_121530(	PF3D7_1215300::10 kDa chaperonin	0.501	0.661	0.791	0.661	0.581	0.271	-0.389	0.121	-0.189	-0.849	-0.929	-0.369	-0.859	0.653	0.154	-0.306	-0.719	-0.959	0.514
PF3D7_052750(	PF3D7_0527500::Hsc70-interacting protein	0.398	0.168	0.968	0.238	0.448	0.008	-0.112	0.168	-0.472	-0.492	-0.242	-0.582	-0.492	0.443	0.114	-0.266	-0.439	-0.708	0.612
PF3D7_011070(	PF3D7_0110700::chromatin assembly factor 1 subunit C, putative	0.228	0.398	0.598	0.218	0.358	0.118	-0.202	-0.042	-0.392	-0.182	-0.252	-0.432	-0.412	0.360	0.091	-0.206	-0.366	-0.566	0.676
PF3D7_134630(	PF3D7_1346300::DNA/RNA-binding protein Alba 2	0.262	0.162	0.342	0.142	0.102	0.142	-0.128	0.032	-0.248	-0.048	-0.118	-0.338	-0.308	0.227	0.039	-0.088	-0.254	-0.315	0.804
PF3D7_133680(	PF3D7_1336800::nuclear movement protein, putative	0.228	0.268	0.698	0.128	0.428	0.348	-0.132	0.008	-0.142	-0.132	-0.292	-0.732	-0.672	0.330	0.214	-0.089	-0.566	-0.419	0.748
PF3D7_142830(	PF3D7_1428300::proliferation-associated protein 2g4, putative	0.159	0.179	0.599	0.359	0.269	0.329	-0.021	0.109	-0.261	-0.101	-0.621	-0.721	-0.281	0.324	0.193	-0.084	-0.541	-0.408	0.753
PF3D7_120350(	PF3D7_1203500::threonylcarbamoyl-AMP synthase, putative	0.285	0.115	0.385	0.225	0.035	0.085	0.085	0.015	-0.255	-0.255	-0.105	-0.455	-0.165	0.253	0.069	-0.165	-0.241	-0.418	0.749
PF3D7_031550(	PF3D7_0315500::ribosomal protein L47, mitochondrial, putative	0.162	0.102	0.372	0.222	0.162	0.232	0.112	0.032	-0.118	-0.238	-0.118	-0.638	-0.278	0.214	0.168	-0.108	-0.345	-0.323	0.800
PF3D7_060240(	PF3D7_0602400::elongation factor G	0.325	0.325	0.465	0.225	0.045	0.475	0.205	0.165	-0.215	-0.215	-0.295	-1.125	-0.375	0.335	0.241	-0.089	-0.599	-0.423	0.746
PF3D7_093360(	PF3D7_0933600::mitochondrial-processing peptidase subunit beta, puta	0.238	0.328	0.428	0.228	0.048	0.438	0.108	0.088	-0.132	-0.052	-0.242	-1.082	-0.392	0.305	0.198	-0.032	-0.572	-0.338	0.791
PF3D7_102470(	PF3D7_1024700::conserved protein, unknown function	0.231	0.201	0.401	0.381	0.021	0.331	0.101	0.111	-0.029	-0.259	-0.169	-0.969	-0.349	0.303	0.151	-0.059	-0.496	-0.363	0.778
PF3D7_092130(	PF3D7_0921300::RAP protein, putative	0.328	0.128	0.158	0.338	0.168	0.668	0.328	0.098	-0.462	-0.382	-0.342	-0.832	-0.202	0.238	0.388	-0.248	-0.458	-0.487	0.714
PF3D7_080700(	PF3D7_0807000::YEATS domain-containing protein, putative	0.143	0.363	0.163	0.333	0.243	0.603	0.193	0.073	-0.257	-0.197	-0.267	-0.937	-0.457	0.251	0.346	-0.127	-0.554	-0.378	0.770
PF3D7_071680(	PF3D7_0716800::eukaryotic translation initiation factor 3 subunit I, putat	0.424	0.324	0.234	0.284	0.524	0.514	0.104	0.114	-0.326	-0.356	-0.336	-1.006	-0.496	0.316	0.381	-0.189	-0.613	-0.506	0.704
PF3D7_132780(	PF3D7_1327800::ribose-phosphate pyrophosphokinase, putative	0.396	0.226	0.086	0.196	0.176	0.466	0.136	-0.004	-0.184	-0.324	-0.194	-0.674	-0.304	0.226	0.259	-0.171	-0.391	-0.397	0.760
PF3D7_122160(	PF3D7_1221600::protein TSSC1, putative	0.151	0.571	0.011	0.051	-0.069	0.581	0.231	-0.019	-0.179	-0.239	-0.249	-0.679	-0.159	0.196	0.247	-0.146	-0.363	-0.342	0.789
PF3D7_134790(	PF3D7_1347900::conserved Plasmodium protein, unknown function	0.182	0.302	0.032	0.032	0.072	0.512	0.142	0.022	-0.088	-0.128	-0.148	-0.728	-0.198	0.137	0.242	-0.065	-0.358	-0.202	0.870
PF3D7_146750(	PF3D7_1467500::DNA/RNA-binding protein KIN17, putative	0.154	0.364	0.144	0.064	-0.116	0.694	0.414	-0.006	-0.186	-0.086	-0.176	-0.836	-0.426	0.181	0.331	-0.093	-0.479	-0.274	0.827
PF3D7_021630(	PF3D7_0216300::conserved Plasmodium protein, unknown function	0.305	0.725	0.115	0.115	0.235	0.395	0.235	-0.005	-0.295	-0.105	-0.485	-0.695	-0.545	0.315	0.289	-0.135	-0.575	-0.450	0.732
PF3D7_070640(	PF3D7_0706400::60S ribosomal protein L37	0.308	0.688	0.178	0.128	0.158	0.398	0.058	0.058	-0.242	-0.332	-0.462	-0.602	-0.332	0.325	0.204	-0.172	-0.466	-0.498	0.708
PF3D7_112200(	PF3D7_1122000::conserved protein, unknown function	0.208	0.908	0.388	0.398	0.338	0.758	0.308	0.098	-0.522	-0.532	-0.832	-0.952	-0.572	0.476	0.468	-0.318	-0.785	-0.794	0.577
PF3D7_124670(	PF3D7_1246700::conserved Plasmodium protein, unknown function	0.275	0.985	0.375	0.215	0.085	0.465	0.135	-0.025	-0.395	-0.195	-0.505	-0.905	-0.505	0.462	0.228	-0.205	-0.639	-0.668	0.630
PF3D7_130780(	PF3D7_1307800::FHA domain-containing protein, putative	0.171	0.681	0.431	0.101	0.141	0.361	0.211	-0.029	-0.379	-0.319	-0.289	-0.719	-0.359	0.346	0.237	-0.243	-0.456	-0.588	0.665
PF3D7_042020(	PF3D7_0420200::holo-[acyl-carrier-protein] synthase, putative	0.284	0.594	0.214	0.444	0.024	0.574	0.224	0.044	-0.546	-0.356	-0.366	-0.806	-0.326	0.384	0.274	-0.286	-0.499	-0.670	0.629
PF3D7_131410(	PF3D7_1314100::conserved Plasmodium protein, unknown function	0.202	0.762	0.172	0.212	0.312	0.672	0.242	0.122	-0.398	-0.568	-0.338	-1.128	-0.258	0.337	0.408	-0.282	-0.575	-0.618	0.651
PF3D7_131490(	PF3D7_1314900::general transcription factor IIH subunit 2	0.371	0.881	0.131	0.191	0.161	0.441	0.171	0.061	-0.499	-0.589	-0.199	-0.789	-0.329	0.393	0.257	-0.343	-0.439	-0.736	0.600
PF3D7_071550(	PF3D7_0715500::ATP synthase subunit epsilon, mitochondrial, putative	0.337	0.567	0.247	0.247	-0.023	0.567	0.177	0.027	-0.173	-0.303	-0.513	-0.873	-0.283	0.349	0.240	-0.150	-0.556	-0.499	0.708

PF3D7_124310(	PF3D7_1243100::zinc finger protein, putative	0.435	0.685	0.725	0.515	-0.165	0.585	0.195	0.085	-0.175	-0.415	-0.525	-1.265	-0.675	0.590	0.205	-0.169	-0.822	-0.758	0.591
PF3D7_060320(	PF3D7_0603200::mitochondrial chaperone BCS1, putative	0.202	0.532	0.292	0.172	0.212	0.412	0.012	-0.068	-0.238	-0.048	-0.328	-0.828	-0.328	0.300	0.212	-0.118	-0.494	-0.418	0.749
PF3D7_144510(	PF3D7_1445100::histidine--tRNA ligase, putative	0.387	0.487	0.127	0.177	-0.043	0.277	0.117	-0.053	-0.223	0.017	-0.323	-0.713	-0.233	0.294	0.117	-0.086	-0.423	-0.381	0.768
PF3D7_052060(	PF3D7_0520600::bis(5'-nucleosyl)-tetraphosphatase [asymmetrical]	0.328	0.528	0.208	0.308	0.208	0.438	0.128	0.148	-0.112	-0.022	-0.712	-0.782	-0.672	0.343	0.258	0.005	-0.722	-0.338	0.791
PF3D7_136040(	PF3D7_1360400::conserved Plasmodium protein, unknown function	0.215	0.615	0.375	0.315	0.225	0.335	0.135	0.115	-0.255	-0.125	-0.475	-0.835	-0.645	0.380	0.232	-0.088	-0.651	-0.468	0.723
PF3D7_112640(	PF3D7_1126400::mediator of RNA polymerase II transcription subunit 21	0.270	0.560	0.340	0.200	0.090	0.170	0.070	0.060	-0.200	-0.180	-0.560	-0.320	-0.500	0.343	0.110	-0.107	-0.460	-0.449	0.732
PF3D7_070790(	PF3D7_0707900::ribosomal protein S8e, putative	0.140	0.460	0.160	0.140	-0.040	0.420	-0.070	0.130	-0.110	-0.140	-0.420	-0.370	-0.300	0.225	0.103	-0.040	-0.363	-0.265	0.832
PF3D7_093330(	PF3D7_0933300::conserved Plasmodium protein, unknown function	0.126	0.506	0.056	0.066	-0.114	0.266	0.066	0.026	-0.074	-0.034	-0.234	-0.374	-0.284	0.189	0.073	-0.027	-0.297	-0.216	0.861
PF3D7_144530(	PF3D7_1445300::ribosomal protein S29, mitochondrial, putative	0.322	0.682	0.032	0.112	-0.068	0.402	-0.038	-0.138	-0.118	-0.218	-0.218	-0.398	-0.348	0.287	0.098	-0.158	-0.322	-0.445	0.735
PF3D7_133980(	PF3D7_1339800::mitotic-spindle organizing protein 1, putative	0.322	1.062	0.152	0.072	0.062	0.772	0.252	-0.018	-0.338	-0.558	-0.498	-0.718	-0.568	0.402	0.362	-0.304	-0.594	-0.707	0.613
PF3D7_134130(	PF3D7_1341300::60S ribosomal protein L18-2, putative	0.144	0.674	0.134	0.234	-0.186	0.544	0.074	0.124	-0.256	-0.476	-0.276	-0.446	-0.286	0.296	0.144	-0.203	-0.336	-0.499	0.708
PF3D7_113010(	PF3D7_1130100::60S ribosomal protein L38	0.097	0.477	0.277	0.107	0.157	0.277	-0.163	-0.083	0.017	-0.053	-0.393	-0.463	-0.253	0.239	0.090	-0.040	-0.370	-0.279	0.824
PF3D7_140760(	PF3D7_1407600::conserved Plasmodium protein, unknown function	0.468	0.418	0.428	0.178	-0.142	0.308	0.108	-0.092	-0.342	-0.112	-0.152	-0.432	-0.632	0.373	0.091	-0.182	-0.406	-0.555	0.681
PF3D7_052690(	PF3D7_0526900::transmembrane emp24 domain-containing protein, put	0.401	0.081	0.061	0.331	-0.039	0.371	0.101	-0.059	-0.039	-0.319	-0.239	-0.369	-0.279	0.218	0.144	-0.139	-0.296	-0.358	0.781
PF3D7_120830(	PF3D7_1208300::acyl carrier protein, mitochondrial, putative	0.442	0.152	0.352	0.172	0.232	0.512	0.092	0.072	-0.018	-0.288	-0.718	-0.588	-0.408	0.279	0.278	-0.078	-0.572	-0.358	0.781
PF3D7_144590(	PF3D7_1445900::ATP-dependent RNA helicase DDX5, putative	0.525	0.225	0.155	0.145	0.125	0.655	0.125	-0.105	-0.135	-0.355	-0.535	-0.395	-0.425	0.262	0.301	-0.199	-0.452	-0.461	0.727
PF3D7_125170(	PF3D7_1251700::tryptophan--tRNA ligase	0.078	0.278	0.228	0.178	0.038	0.558	0.118	0.008	-0.282	-0.262	-0.412	-0.082	-0.452	0.191	0.238	-0.178	-0.315	-0.369	0.774
PF3D7_130490(	PF3D7_1304900::DNA-directed RNA polymerase II subunit RPB11, puta	0.258	0.348	0.198	0.158	0.088	0.398	-0.042	0.048	-0.262	-0.282	-0.332	-0.122	-0.452	0.240	0.148	-0.166	-0.302	-0.406	0.755
PF3D7_093300(	PF3D7_0933000::CSTF domain-containing protein, putative	0.250	0.210	0.060	0.030	-0.180	0.080	0.070	-0.020	-0.180	-0.110	-0.010	0.060	-0.260	0.138	-0.010	-0.103	-0.070	-0.241	0.846
PF3D7_120190(	PF3D7_1201900::conserved protein, unknown function	0.294	0.144	0.074	0.084	-0.126	0.234	0.094	-0.036	-0.166	0.074	-0.306	0.124	-0.486	0.149	0.067	-0.043	-0.223	-0.192	0.876
PF3D7_141250(	PF3D7_1412500::actin II	0.368	0.018	0.018	0.038	-0.022	0.038	-0.002	-0.032	-0.042	-0.152	-0.172	0.048	-0.102	0.110	0.004	-0.076	-0.076	-0.186	0.879
PF3D7_121040(	PF3D7_1210400::general transcription factor 3C polypeptide 5, putative	0.329	0.029	0.039	0.089	-0.001	0.469	-0.461	0.029	-0.031	0.029	-0.041	-0.241	-0.241	0.122	0.003	0.009	-0.174	-0.113	0.925

Shown are the mean-centered $\log_2$ ratios of each sample against a 3D7 reference pool, the averaged $\log_2$ ratios for each parasite line grouped by PfCRT haplotype, and the fold change for $Dd2^{G353Vcrt}$ vs Dd2 lines.

Gene	Description	Dd2 repA	Dd2 repB	$Dd2^{Dd2crt}$ repA	$Dd2^{Dd2crt}$ repB	$Dd2^{M343Lcrt}$ repA	$Dd2^{M343Lcrt}$ repB	$Dd2^{M343Lcrt}$ repC	$Dd2^{G353Vcrt}$ repC	$Dd2^{G353Vcrt}$ repB	$Dd2^{G353Vcrt}$ repA	$Dd2^{F145Icrt}$ repA	$Dd2^{F145Icrt}$ repB	$Dd2^{F145Icrt}$ repC	Averaged Dd2	Averaged $Dd2^{M343Lcrt}$	Averaged $Dd2^{G353Vcrt}$	Averaged $Dd2^{F145Icrt}$	Log_2 fold change of $Dd2^{M343Lcrt}$/ $Dd2^{Dd2crt}$	Fold change of $Dd2^{M343Lcrt}$/ $Dd2^{Dd2crt}$
PF3D7_144680(	PF3D7_1446800::heme detoxification protein	0.050	-1.820	-0.200	0.180	0.260	0.550	0.260	0.170	0.060	0.150	0.190	0.010	0.140	-0.448	0.357	0.127	0.113	0.804	1.746
PF3D7_145870(	PF3D7_1458700::exonuclease V, mitochondrial, putative	-0.225	-0.575	-0.095	-0.105	-0.055	0.185	0.235	-0.185	-0.075	0.365	0.075	0.245	0.205	-0.250	0.122	0.035	0.175	0.372	1.294
PF3D7_123030(	PF3D7_1230300::subpellicular microtubule protein 2, putative	-0.320	-1.000	-0.140	-0.170	-0.060	0.180	0.080	-0.150	-0.090	0.520	0.090	0.630	0.430	-0.408	0.067	0.093	0.383	0.474	1.389
PF3D7_121110(	PF3D7_1211100::HSP20-like chaperone, putative	-0.172	-1.012	-0.282	-0.392	-0.062	-0.012	-0.012	-0.112	0.058	1.058	0.308	0.608	0.028	-0.465	-0.029	0.334	0.314	0.436	1.353
PF3D7_133040(	PF3D7_1330400::ER lumen protein retaining receptor 1, putative	-0.220	-0.170	-0.530	-0.140	0.180	0.380	0.000	-0.040	0.290	0.150	-0.060	0.120	0.040	-0.265	0.187	0.133	0.033	0.452	1.368
PF3D7_135050(	PF3D7_1350500::conserved Plasmodium protein, unknown function	-0.137	-0.197	-0.437	-0.257	0.023	-0.107	0.053	-0.067	0.113	0.083	0.063	0.503	0.363	-0.257	-0.010	0.043	0.310	0.247	1.186
PF3D7_112020(	PF3D7_1120200::conserved Plasmodium protein, unknown function	-0.406	-0.516	-0.806	-0.336	-0.126	0.364	0.144	-0.106	0.244	0.204	0.054	0.774	0.514	-0.516	0.127	0.114	0.447	0.643	1.562
PF3D7_123260(	PF3D7_1232600::conserved Plasmodium protein, unknown function	-0.367	-0.437	-0.577	-0.397	-0.097	-0.057	-0.217	-0.397	0.063	0.263	0.103	1.773	0.343	-0.444	-0.124	-0.024	0.740	0.321	1.249
PF3D7_082140(	PF3D7_0821400::conserved Plasmodium protein, unknown function	-0.242	-0.312	-0.692	-0.142	-0.122	0.148	0.018	-0.192	-0.022	0.088	-0.132	1.368	0.228	-0.347	0.015	-0.042	0.488	0.362	1.285
PF3D7_111190(	PF3D7_1111900::Josephin domain-containing protein, putative	-0.365	-0.605	-0.155	-0.105	0.215	0.635	0.005	-0.215	0.095	-0.315	0.015	0.785	0.005	-0.307	0.285	-0.145	0.269	0.593	1.508
PF3D7_140520(	PF3D7_1405200::trafficking protein particle complex subunit 1, putative	-0.356	-0.046	-0.276	0.014	0.294	0.474	0.094	0.084	0.144	-0.566	0.124	-0.066	0.084	-0.166	0.287	-0.113	0.047	0.453	1.369
PF3D7_141440(	PF3D7_1414400::serine/threonine protein phosphatase PP1	-0.084	0.116	-0.494	0.056	0.256	0.216	0.186	-0.084	0.026	-0.894	0.136	0.326	0.236	-0.101	0.219	-0.317	0.233	0.321	1.249
PF3D7_122450(	PF3D7_1224500::histone chaperone ASF1	-0.048	0.162	-0.378	0.072	0.342	0.462	0.302	-0.018	0.022	-1.098	0.102	0.012	0.062	-0.048	0.369	-0.364	0.059	0.417	1.335
PF3D7_146810(	PF3D7_1468100::MORC family protein	-0.216	-0.306	-0.276	-0.106	0.304	0.124	0.304	0.104	0.094	-0.696	0.314	0.134	0.224	-0.226	0.244	-0.166	0.224	0.470	1.385
PF3D7_122760(	PF3D7_1227600::kinetochore protein SPC24, putative	-0.093	-0.533	-0.193	-0.053	0.267	0.197	0.427	0.037	0.157	-0.923	0.177	0.177	0.357	-0.218	0.297	-0.243	0.237	0.515	1.429
PF3D7_API044(	PF3D7_API04400::DNA-directed RNA polymerase subunit beta, putative	-0.157	-0.177	-0.657	-0.197	0.063	0.183	0.123	0.113	0.413	-0.547	0.013	0.243	0.583	-0.297	0.123	-0.007	0.280	0.420	1.338
PF3D7_120540(	PF3D7_1205400::kelch domain-containing protein, putative	-0.050	-0.150	-0.380	-0.020	0.030	0.220	0.150	0.110	0.090	-0.480	0.040	0.160	0.280	-0.150	0.133	-0.093	0.160	0.283	1.217
PF3D7_082100(	PF3D7_0821000::conserved Plasmodium protein, unknown function	-0.200	-0.130	-0.450	-0.010	0.090	0.300	0.180	0.010	0.030	-0.460	0.110	0.340	0.190	-0.198	0.190	-0.140	0.213	0.388	1.308
PF3D7_111650(	PF3D7_1116500::folate transporter 2	-0.123	-0.303	-0.293	-0.063	0.127	0.117	0.007	0.047	-0.103	-0.543	0.197	0.647	0.287	-0.196	0.084	-0.200	0.377	0.279	1.213
PF3D7_041080(	PF3D7_0410800::conserved Plasmodium protein, unknown function	-0.180	-0.280	-0.380	-0.120	0.080	-0.040	0.090	-0.050	0.140	-0.420	0.180	0.650	0.330	-0.240	0.043	-0.110	0.387	0.283	1.217
PF3D7_072380(	PF3D7_0723800::conserved Plasmodium protein, unknown function	-0.124	-0.254	-0.214	-0.114	0.126	-0.054	0.076	0.016	0.116	-0.364	0.246	0.266	0.276	-0.176	0.049	-0.077	0.263	0.226	1.169
PF3D7_072790(	PF3D7_0727900::conserved Plasmodium protein, unknown function	-0.033	-0.293	-0.103	-0.023	0.167	0.157	0.027	0.137	-0.103	-0.333	0.027	0.267	0.107	-0.113	0.117	-0.100	0.134	0.230	1.173
PF3D7_124980(	PF3D7_1249800::THO complex subunit 2, putative	-0.238	-0.078	-0.068	-0.018	0.002	0.182	0.162	0.102	0.022	-0.038	-0.028	0.032	-0.028	-0.101	0.115	0.028	-0.008	0.216	1.161
PF3D7_080500(	PF3D7_0805000::alpha/beta hydrolase, putative	-0.786	-0.246	-0.266	0.024	0.344	0.354	0.324	0.094	0.104	-0.006	0.114	-0.216	0.164	-0.319	0.341	0.064	0.021	0.659	1.579
PF3D7_130890(	PF3D7_1308900::mRNA-decapping enzyme 2, putative	0.047	-0.353	0.087	-0.043	0.127	0.517	0.447	0.147	-0.063	0.047	-0.063	-0.703	-0.193	-0.066	0.364	0.044	-0.320	0.429	1.346
PF3D7_124120(	PF3D7_1241200::conserved Plasmodium protein, unknown function	-0.110	-0.060	-0.070	0.000	0.050	0.270	0.260	0.160	-0.080	0.130	0.010	-0.450	-0.110	-0.060	0.193	0.070	-0.183	0.253	1.192
PF3D7_122240(	PF3D7_1222400::AP2 domain transcription factor	-0.134	0.036	0.036	-0.134	0.096	0.486	0.426	-0.004	-0.004	0.136	-0.084	-0.544	-0.314	-0.049	0.336	0.043	-0.314	0.385	1.306
PF3D7_114240(	PF3D7_1142400::coproporphyrinogen-III oxidase	0.023	0.013	0.013	0.023	0.073	0.353	0.283	0.003	-0.107	-0.137	-0.207	-0.347	0.013	0.018	0.236	-0.080	-0.180	0.218	1.163
PF3D7_123200(	PF3D7_1232000::phenylalanine--tRNA ligase	-0.322	0.038	0.098	0.108	0.138	0.408	0.378	0.318	-0.022	-0.282	-0.092	-0.612	-0.152	-0.020	0.308	0.004	-0.286	0.328	1.255
PF3D7_123360(	PF3D7_1233600::asparagine and aspartate rich protein 1	0.070	0.000	0.060	0.060	0.110	0.400	0.240	0.180	0.010	-0.270	-0.150	-0.590	-0.120	0.048	0.250	-0.027	-0.287	0.203	1.151
PF3D7_081120(	PF3D7_0811200::ER membrane protein complex subunit 1, putative	-0.193	-0.003	-0.003	0.067	0.307	0.577	0.207	0.367	0.087	-0.423	-0.253	-0.653	-0.083	-0.033	0.364	0.010	-0.330	0.397	1.316
PF3D7_145960(	PF3D7_1459600::conserved Plasmodium protein, unknown function	-0.123	-0.293	-0.183	-0.023	0.037	0.527	0.447	0.087	0.117	-0.173	-0.173	-0.253	0.007	-0.156	0.337	0.010	-0.140	0.493	1.407
PF3D7_082840(	PF3D7_0828400::mitochondrial inner membrane protein OXA1, putative	0.011	-0.209	0.021	-0.019	0.071	0.351	0.461	0.071	0.181	-0.199	-0.089	-0.469	-0.179	-0.049	0.294	0.017	-0.246	0.343	1.269
PF3D7_071390(	PF3D7_0713900::conserved Plasmodium protein, unknown function	0.018	-0.172	-0.142	-0.072	0.098	0.288	0.318	0.198	0.068	-0.152	0.008	-0.512	0.048	-0.092	0.235	0.038	-0.152	0.327	1.254
PF3D7_141630(	PF3D7_1416300::N-acyl-phosphatidylethanolamine-hydrolyzing phosphc	0.188	0.188	-0.052	0.038	0.268	0.468	0.258	0.008	0.028	-0.842	-0.322	-0.362	0.138	0.090	0.331	-0.269	-0.182	0.241	1.182
PF3D7_103310(	PF3D7_1033100::S-adenosylmethionine decarboxylase/ornithine decarb	0.138	-0.142	0.108	0.068	0.318	0.568	0.158	0.038	-0.062	-1.042	-0.072	-0.002	-0.082	0.043	0.348	-0.355	-0.052	0.305	1.235
PF3D7_131700(	PF3D7_1317000::U4/U6.U5 tri-snRNP-associated protein 2, putative	0.151	-0.129	0.081	0.131	0.361	0.581	0.261	0.091	-0.149	-0.529	-0.249	-0.379	-0.219	0.058	0.401	-0.196	-0.283	0.343	1.268
PF3D7_052910(	PF3D7_0529100::thioredoxin-like protein, putative	0.237	-0.043	0.107	0.177	0.577	0.667	0.327	0.107	-0.013	-1.103	-0.153	-0.533	-0.353	0.119	0.524	-0.336	-0.346	0.404	1.323
PF3D7_146320(	PF3D7_1463200::replication factor C subunit 3, putative	0.372	-0.548	0.392	0.342	0.412	0.552	0.462	0.182	-0.078	-1.388	-0.008	-0.648	-0.048	0.140	0.476	-0.428	-0.234	0.336	1.262
PF3D7_141780(	PF3D7_1417800::DNA replication licensing factor MCM2	0.234	-0.596	0.134	0.194	0.244	0.414	0.424	0.194	0.014	-1.206	0.304	-0.506	0.154	-0.009	0.361	-0.333	-0.016	0.369	1.292
PF3D7_030150(	PF3D7_0301500::Plasmodium exported protein, unknown function	-0.064	-0.634	0.136	0.076	0.206	0.156	0.406	0.256	0.176	-0.904	0.226	-0.234	0.196	-0.121	0.256	-0.157	0.063	0.378	1.299
PF3D7_121590(	PF3D7_1215900::serpentine receptor 10	-0.114	0.026	-0.344	0.136	0.336	0.396	0.426	0.296	-0.174	-0.794	-0.054	-0.234	0.096	-0.074	0.386	-0.224	-0.064	0.460	1.376
PF3D7_102130(	PF3D7_1021300::apicoplast integral membrane protein, putative	0.068	-0.042	-0.262	0.128	0.198	0.338	0.228	0.028	-0.022	-0.582	0.338	-0.512	0.088	-0.027	0.255	-0.192	-0.028	0.282	1.216
PF3D7_101360(	PF3D7_1013600::conserved Plasmodium protein, unknown function	-0.132	-0.012	0.008	0.048	0.088	0.108	0.178	0.068	0.018	-0.302	0.108	-0.192	0.008	-0.022	0.125	-0.072	-0.025	0.147	1.107
PF3D7_072460(	PF3D7_0724600::protein kinase, putative	-0.037	0.023	-0.307	-0.007	0.073	0.343	0.493	0.183	0.183	-0.547	0.093	-0.407	-0.087	-0.082	0.303	-0.060	-0.134	0.385	1.306
PF3D7_122230(	PF3D7_1222300::endoplasmin, putative	0.098	-0.032	-0.042	0.078	0.328	0.448	0.158	0.198	0.178	-0.912	0.088	-0.532	-0.062	0.026	0.312	-0.178	-0.168	0.286	1.219
PF3D7_091790(	PF3D7_0917900::heat shock protein 70	0.094	-0.166	-0.226	0.064	0.254	0.664	0.264	0.074	0.314	-0.936	-0.036	-0.476	0.114	-0.059	0.394	-0.183	-0.133	0.453	1.368
PF3D7_052700(	PF3D7_0527000::DNA replication licensing factor MCM3, putative	0.081	-0.279	-0.219	-0.039	0.221	0.501	0.401	0.111	0.081	-0.979	0.141	-0.139	0.121	-0.114	0.374	-0.263	0.041	0.488	1.403
PF3D7_082380(	PF3D7_0823800::DnaJ protein, putative	-0.248	-0.268	-0.258	-0.008	0.212	0.572	0.412	0.232	0.212	-0.598	-0.088	-0.338	0.162	-0.195	0.399	-0.051	-0.088	0.594	1.510
PF3D7_081730(	PF3D7_0817300::conserved Plasmodium protein, unknown function	-0.039	-0.369	-0.029	-0.089	0.181	0.451	0.341	0.151	-0.049	-0.479	0.121	-0.309	0.121	-0.132	0.324	-0.126	-0.023	0.456	1.372
PF3D7_146230(	PF3D7_1462300::GTP-binding protein, putative	-0.042	-0.142	-0.002	0.108	0.298	0.428	0.368	0.348	0.068	-0.482	-0.102	-0.732	-0.122	-0.019	0.365	-0.022	-0.318	0.384	1.305
PF3D7_082610(	PF3D7_0826100::HECT-like E3 ubiquitin ligase, putative	-0.015	-0.215	-0.015	0.075	0.205	0.355	0.335	0.245	-0.025	-0.455	0.005	-0.435	-0.065	-0.042	0.299	-0.078	-0.165	0.341	1.266
PF3D7_062820(	PF3D7_0628200::eukaryotic translation initiation factor 2-alpha kinase	0.006	-0.264	-0.054	0.046	0.186	0.306	0.276	0.216	0.026	-0.404	0.016	-0.364	0.006	-0.066	0.256	-0.054	-0.114	0.323	1.250
PF3D7_060900(	PF3D7_0609000::nucleoporin NUP637, putative	-0.017	-0.097	-0.027	0.053	0.133	0.213	0.193	0.203	-0.037	-0.467	0.083	-0.267	0.033	-0.022	0.180	-0.100	-0.050	0.202	1.150
PF3D7_031430(	PF3D7_0314300::DER1-like protein	0.195	-0.175	-0.035	0.235	0.425	0.915	0.635	0.215	-0.145	-1.295	0.075	-0.935	-0.115	0.055	0.659	-0.408	-0.325	0.603	1.519
PF3D7_010870(	PF3D7_0108700::secreted ookinete protein, putative	0.061	-0.199	-0.049	0.071	0.081	0.601	0.411	0.231	0.031	-0.669	0.011	-0.509	-0.069	-0.029	0.364	-0.136	-0.189	0.393	1.313
PF3D7_146960(	PF3D7_1469600::acetyl-CoA carboxylase	-0.025	-0.215	-0.145	-0.415	0.075	0.205	0.325	0.025	0.175	-0.195	0.105	-0.085	0.165	-0.200	0.202	0.002	0.062	0.402	1.321
PF3D7_122880(	PF3D7_1228800::WD repeat-containing protein, putative	-0.202	-0.432	-0.042	0.008	0.108	0.188	0.168	0.158	0.148	-0.262	0.188	-0.122	0.088	-0.167	0.155	0.015	0.052	0.322	1.250
PF3D7_092650(	PF3D7_0926500::tetratricopeptide repeat protein, putative	-0.155	-0.395	-0.235	-0.255	0.045	0.255	0.315	0.045	0.275	-0.065	0.205	-0.225	0.185	-0.260	0.205	0.085	0.055	0.465	1.380
PF3D7_051680(	PF3D7_0516800::AP2 domain transcription factor AP2-O2, putative	-0.197	-0.557	-0.237	-0.047	0.023	0.363	0.453	0.193	0.303	-0.287	0.263	-0.467	0.193	-0.259	0.280	0.070	-0.004	0.539	1.453
PF3D7_060350(	PF3D7_0603500::cation/H+ antiporter	0.055	-0.505	-0.095	0.005	0.075	0.525	0.205	0.115	0.175	-0.335	0.235	-0.365	-0.085	-0.135	0.268	-0.015	-0.072	0.403	1.323
PF3D7_040360(	PF3D7_0403600::conserved Plasmodium protein, unknown function	0.005	-0.745	0.015	0.115	0.155	0.465	0.375	0.055	0.055	-0.325	0.035	-0.255	0.055	-0.153	0.331	-0.072	-0.055	0.484	1.399
PF3D7_101130(	PF3D7_1011300::protein ARV1, putative	-0.096	-0.206	-0.036	-0.106	0.054	0.214	0.074	0.044	0.234	0.724	0.074	-0.626	-0.346	-0.111	0.114	0.334	-0.299	0.225	1.169
PF3D7_092580(	PF3D7_0925800::regulator of nonsense transcripts 2, putative	-0.359	-0.519	0.001	0.031	0.121	0.171	0.341	0.221	0.011	0.391	-0.079	-0.419	0.091	-0.212	0.211	0.207	-0.136	0.423	1.340
PF3D7_021800(	PF3D7_0218000::replication factor C subunit 2, putative	-0.346	-0.056	0.084	0.104	0.194	0.184	0.184	0.224	0.144	0.054	-0.076	-0.826	0.134	-0.054	0.187	0.141	-0.256	0.241	1.182
PF3D7_140790(	PF3D7_1407900::plasmepsin I	0.685	0.355	0.265	0.225	0.075	0.185	0.215	0.205	-0.285	-0.505	-0.455	-0.725	-0.245	0.383	0.159	-0.195	-0.475	-0.224	0.856
PF3D7_146130(	PF3D7_1461300::40S ribosomal protein S28e, putative	0.323	0.703	0.603	0.373	0.183	0.023	0.013	0.113	-0.177	-0.917	-0.217	-0.707	-0.317	0.501	0.073	-0.327	-0.414	-0.428	0.744
PF3D7_144330(	PF3D7_1443300::U6 snRNA-associated Sm-like protein LSm5, putative	0.363	0.733	0.283	0.303	0.253	0.113	0.013	0.143	-0.027	-0.747	-0.267	-0.657	-0.507	0.421	0.126	-0.210	-0.477	-0.294	0.816
PF3D7_146070(	PF3D7_1460700::60S ribosomal protein L27	0.567	1.017	0.517	0.517	0.357	0.087	-0.213	0.027	-0.103	-0.903	-0.413	-1.063	-0.393	0.654	0.077	-0.326	-0.623	-0.578	0.670
PF3D7_131780(	PF3D7_1317800::40S ribosomal protein S19	0.405	1.005	0.525	0.515	0.375	0.095	-0.105	0.105	-0.245	-0.955	-0.475	-0.765	-0.485	0.613	0.122	-0.365	-0.575	-0.491	0.712

PF3D7_021010(	PF3D7_0210100::60S ribosomal protein L37ae, putative	0.390	0.760	0.565	0.300	0.180	0.030	-0.205	0.125	-0.220	-0.500	-0.280	-0.565	-0.575	0.503	0.001	-0.199	-0.474	-0.502	0.706
PF3D7_146590(	PF3D7_1465900::40S ribosomal protein S3	0.555	0.495	0.725	0.615	0.325	0.285	-0.055	0.035	-0.215	-0.745	-0.575	-0.895	-0.555	0.598	0.185	-0.308	-0.675	-0.413	0.751
PF3D7_144710(	PF3D7_1447100::conserved Plasmodium protein, unknown function	0.237	0.297	0.267	0.307	0.067	0.177	0.047	0.107	0.097	-0.453	-0.173	-0.603	-0.373	0.277	0.097	-0.083	-0.383	-0.180	0.883
PF3D7_132340(	PF3D7_1323400::60S ribosomal protein L23	0.495	0.735	0.455	0.505	0.345	0.365	0.105	0.205	-0.205	-0.885	-0.415	-1.065	-0.635	0.547	0.271	-0.295	-0.705	-0.276	0.826
PF3D7_051700(	PF3D7_0517000::60S ribosomal protein L12, putative	0.462	0.572	0.412	0.432	0.302	0.222	0.052	0.322	-0.058	-0.818	-0.478	-0.948	-0.468	0.469	0.192	-0.185	-0.632	-0.278	0.825
PF3D7_050380(	PF3D7_0503800::60S ribosomal protein L31	0.495	0.645	0.495	0.575	0.455	0.205	-0.015	0.075	-0.235	-1.235	-0.525	-0.585	-0.355	0.553	0.215	-0.465	-0.488	-0.338	0.791
PF3D7_133690(	PF3D7_1336900::tryptophan--tRNA ligase	0.267	0.287	0.217	0.237	0.087	-0.043	-0.283	0.147	-0.093	0.137	-0.093	-0.723	-0.143	0.252	-0.080	0.064	-0.320	-0.332	0.795
PF3D7_147060(	PF3D7_1470600::RAP protein, putative	0.210	0.300	0.470	0.210	0.120	0.190	0.000	0.220	-0.230	0.130	-0.590	-0.620	-0.410	0.298	0.103	0.040	-0.540	-0.194	0.874
PF3D7_124480(	PF3D7_1244800::translation machinery-associated protein 46, putative	0.279	0.289	0.589	0.209	0.139	0.079	-0.051	0.229	-0.251	0.099	-0.341	-0.951	-0.321	0.342	0.056	0.026	-0.537	-0.286	0.820
PF3D7_136480(	PF3D7_1364800::DNA-directed RNA polymerases I, II, and III subunit RF	0.396	0.306	0.936	0.626	0.136	0.146	0.186	0.396	-0.254	-0.354	-0.794	-0.934	-0.794	0.566	0.156	-0.071	-0.841	-0.410	0.753
PF3D7_146930(	PF3D7_1469300::pre-rRNA-processing protein PNO1, putative	0.155	0.765	0.425	0.145	-0.085	0.055	-0.215	0.015	-0.265	0.185	-0.335	-0.495	-0.345	0.372	-0.082	-0.022	-0.392	-0.454	0.730
PF3D7_122400(	PF3D7_1224000::GTP cyclohydrolase 1	0.142	0.732	0.292	0.232	0.082	0.102	-0.098	0.112	-0.208	0.152	-0.368	-0.838	-0.328	0.349	0.028	0.018	-0.512	-0.321	0.801
PF3D7_122600(	PF3D7_1226000::conserved protein, unknown function	0.297	0.577	0.387	0.217	0.087	0.047	-0.143	0.197	-0.213	-0.103	-0.413	-0.573	-0.363	0.369	-0.003	-0.040	-0.450	-0.373	0.772
PF3D7_102840(	PF3D7_1028400::ribosome biogenesis protein RPF2, putative	0.205	0.395	0.345	0.145	0.055	0.005	0.005	0.095	-0.265	-0.125	-0.195	-0.435	-0.225	0.272	0.021	-0.099	-0.285	-0.251	0.840
PF3D7_112640(	PF3D7_1126400::mediator of RNA polymerase II transcription subunit 21	0.270	0.560	0.340	0.200	0.090	0.170	0.070	0.060	-0.200	-0.180	-0.560	-0.320	-0.500	0.343	0.110	-0.107	-0.460	-0.233	0.851
PF3D7_124710(	PF3D7_1247100::conserved protein, unknown function	0.115	0.555	0.235	0.075	-0.135	-0.055	-0.045	0.085	0.105	0.105	-0.355	-0.515	-0.175	0.245	-0.078	0.099	-0.348	-0.323	0.799
PF3D7_072080(	PF3D7_0720800::Ham1-like protein, putative	0.086	0.586	0.186	0.186	-0.014	-0.094	-0.094	0.036	0.046	-0.044	-0.444	-0.294	-0.144	0.261	-0.067	0.013	-0.294	-0.328	0.796
PF3D7_062150(	PF3D7_0621500::ribonuclease P/MRP protein subunit RPP1, putative	0.219	0.709	0.209	0.129	-0.171	-0.011	-0.151	-0.201	-0.031	0.179	-0.341	-0.441	-0.101	0.317	-0.111	-0.017	-0.294	-0.428	0.744
PF3D7_120510(	PF3D7_1205100::O-phosphoseryl-tRNA(Sec) selenium transferase, puta	0.239	0.179	0.429	0.199	0.099	0.119	-0.151	-0.111	-0.191	0.119	-0.441	-0.211	-0.281	0.262	0.023	-0.061	-0.311	-0.239	0.847
PF3D7_103230(	PF3D7_1032300::conserved protein, unknown function	0.145	0.295	0.535	0.115	0.045	0.025	-0.115	0.125	-0.355	-0.095	-0.345	-0.055	-0.315	0.272	-0.015	-0.109	-0.239	-0.288	0.819
PF3D7_102260(	PF3D7_1022600::kelch protein K10	0.120	0.120	0.370	0.170	-0.060	-0.090	-0.140	0.050	-0.270	0.380	-0.310	-0.190	-0.150	0.195	-0.097	0.053	-0.217	-0.292	0.817
PF3D7_091560(	PF3D7_0915600::conserved Plasmodium protein, unknown function	0.098	0.458	0.648	0.128	-0.132	-0.002	-0.022	0.108	-0.312	0.458	-0.492	-0.592	-0.352	0.333	-0.052	0.085	-0.478	-0.385	0.766
PF3D7_071310(	PF3D7_0713100::Pfmc-2TM Maurer's cleft two transmembrane protein	0.112	0.482	0.512	0.122	0.072	-0.168	-0.088	0.212	-0.498	0.272	-0.248	-0.328	-0.458	0.307	-0.061	-0.004	-0.344	-0.368	0.775
PF3D7_091710(	PF3D7_0917100::N-glycosylase/DNA lyase, putative	0.040	0.260	0.520	0.080	0.010	-0.230	-0.070	0.110	-0.150	0.110	-0.150	-0.440	-0.090	0.225	-0.097	0.023	-0.227	-0.322	0.800
PF3D7_052230(	PF3D7_0522300::18S rRNA (guanine-N(7))-methyltransferase, putative	0.228	0.408	0.748	0.188	0.068	-0.362	-0.242	0.038	-0.362	-0.162	-0.342	-0.342	0.128	0.393	-0.178	-0.162	-0.185	-0.572	0.673
PF3D7_071870(	PF3D7_0718700::conserved Plasmodium membrane protein, unknown fu	0.258	0.338	0.348	0.458	-0.072	0.208	-0.062	0.058	-0.722	-0.192	-0.312	-0.042	-0.262	0.350	0.024	-0.286	-0.206	-0.326	0.798
PF3D7_113610(	PF3D7_1136100::conserved Plasmodium protein, unknown function	0.237	0.237	0.417	0.207	0.007	-0.003	0.017	-0.433	-0.123	0.107	-0.083	-0.213	-0.373	0.274	0.007	-0.150	-0.223	-0.268	0.831
PF3D7_147600(	PF3D7_1476000::conserved Plasmodium protein, unknown function	0.560	0.750	0.220	0.040	-0.450	-0.450	-0.400	0.220	0.800	0.120	-0.580	-0.170	-0.660	0.393	-0.433	0.380	-0.470	-0.826	0.564
PF3D7_146310(	PF3D7_1463100::conserved Plasmodium protein, unknown function, pse	0.386	1.046	0.266	0.086	-0.274	-0.114	-0.434	0.186	0.346	-0.044	-0.584	-0.244	-0.624	0.446	-0.274	0.163	-0.484	-0.720	0.607
PF3D7_072620(	PF3D7_0726200::serine/threonine protein kinase, FIKK family	0.248	0.368	0.168	0.148	-0.282	-0.022	-0.032	-0.102	0.158	0.018	-0.202	-0.132	-0.342	0.233	-0.112	0.025	-0.225	-0.345	0.787
PF3D7_060820(	PF3D7_0608200::conserved Plasmodium protein, unknown function	0.347	0.837	0.267	0.217	-0.063	-0.043	-0.123	-0.073	-0.093	-0.373	-0.303	-0.103	-0.493	0.417	-0.076	-0.180	-0.300	-0.493	0.710
PF3D7_042500(	PF3D7_0425000::Plasmodium exported protein, unknown function, pseu	1.559	1.849	1.389	0.899	0.159	-0.291	-0.991	-0.471	-0.241	-0.441	-1.061	-0.771	-1.591	1.424	-0.374	-0.384	-1.141	-1.798	0.288
PF3D7_011450(	PF3D7_0114500::Plasmodium exported protein (hyp10), unknown functi	0.704	0.944	0.774	0.294	-0.586	-0.626	-0.376	-0.356	-0.136	0.244	-0.236	-0.236	-0.406	0.679	-0.529	-0.083	-0.293	-1.208	0.433
PF3D7_070660(	PF3D7_0706600::conserved Plasmodium protein, unknown function	0.207	0.247	0.467	0.197	0.027	-0.133	-0.413	0.147	0.137	0.187	-0.533	0.037	-0.573	0.279	-0.173	0.157	-0.356	-0.453	0.731
PF3D7_121630(	PF3D7_1216300::signal recognition particle subunit SRP19	0.180	0.970	0.120	0.060	-0.180	0.060	0.070	0.030	-0.070	-0.250	-0.230	-0.600	-0.160	0.333	-0.017	-0.097	-0.330	-0.349	0.785
PF3D7_080380(	PF3D7_0803800::proteasome subunit beta type-4	0.184	0.684	0.094	0.034	-0.026	-0.136	-0.076	-0.066	0.104	-0.426	0.034	-0.306	-0.096	0.249	-0.079	-0.129	-0.123	-0.328	0.796
PF3D7_123660(	PF3D7_1236600::p25-alpha family protein, putative	0.289	1.029		-0.011	-0.541	-0.501	-0.341	-0.071	0.139	0.089	-0.051	-0.491	0.459	0.436	-0.461	0.053	-0.027	-0.897	0.537
PF3D7_051910(	PF3D7_0519100::50S ribosomal protein L14, mitochondrial, putative	0.265	0.585	0.145	0.065	-0.145	-0.405	-0.225	-0.035	0.025	-0.145	-0.085	-0.105	0.055	0.265	-0.258	-0.051	-0.045	-0.523	0.696
PF3D7_030980(	PF3D7_0309800::YTH domain-containing protein 2	0.548	0.118	0.288	0.178	0.078	-0.072	-0.222	0.078	-0.052	-0.632	-0.262	0.398	-0.442	0.283	-0.072	-0.202	-0.102	-0.355	0.782
PF3D7_145510(	PF3D7_1455100::protein tyrosine phosphatase, putative	-0.069	0.311	0.121	-0.079	-0.269	-0.409	-0.339	-0.309	-0.399	0.451	-0.059	1.291	-0.239	0.071	-0.339	-0.086	0.331	-0.410	0.753
PF3D7_145000(	PF3D7_1450000::serine/threonine protein kinase, putative	-0.178	0.232	-0.048	-0.118	-0.268	-0.418	-0.328	-0.118	-0.248	0.262	0.032	1.142	0.052	-0.028	-0.338	-0.034	0.409	-0.310	0.807
PF3D7_125280(	PF3D7_1252800::Plasmodium exported protein (PHISTb), unknown func	0.050	-0.010	0.210	0.000	-0.180	-0.400	-0.230	-0.030	-0.030	0.340	-0.010	0.450	-0.160	0.063	-0.270	0.093	0.093	-0.333	0.794
PF3D7_142550(	PF3D7_1425500::conserved Plasmodium protein, unknown function	0.282	-0.138	-0.218	-0.088	-0.588	-0.498	-0.398	-0.038	0.092	0.202	0.012	1.262	0.122	-0.041	-0.495	0.085	0.465	-0.454	0.730
PF3D7_140190(	PF3D7_1401900::conserved protein, unknown function	0.043	0.413	0.023	-0.207	-0.507	-0.607	-0.377	-0.377	0.293	0.113	0.193	1.163	-0.167	0.068	-0.497	0.010	0.396	-0.565	0.676
PF3D7_101050(	PF3D7_1010500::ankyrin-repeat protein, putative	-0.109	0.081	-0.219	-0.099	-0.369	-0.579	-0.599	-0.599	0.541	0.391	0.341	1.031	0.191	-0.087	-0.516	0.111	0.521	-0.429	0.743
PF3D7_135160(	PF3D7_1351600::glycerol kinase	-0.517	-0.677	-0.537	0.573	-0.717	-0.827	-0.807	0.043	1.233	0.233	0.093	1.853	0.053	-0.289	-0.784	0.503	0.666	-0.494	0.710
PF3D7_102020(	PF3D7_1020200::conserved Plasmodium protein, unknown function	-0.298	-0.518	-0.388	0.292	-0.588	-0.828	-0.648	-0.058	0.632	0.132	0.452	1.522	0.292	-0.228	-0.688	0.236	0.756	-0.460	0.727
PF3D7_140350(	PF3D7_1403500::dynein light chain, putative	-0.232	0.108	-0.252	-0.132	-0.342	-0.542	-0.352	-0.312	0.088	0.818	0.198	0.718	0.228	-0.127	-0.412	0.198	0.382	-0.285	0.821
PF3D7_041880(	PF3D7_0418800::MOLO1 domain-containing protein, putative	-0.099	-0.439	-0.179	0.101	-0.709	-0.919	-1.039	-0.529	0.451	1.621	-0.029	1.261	0.511	-0.154	-0.889	0.514	0.581	-0.735	0.601
PF3D7_146570(	PF3D7_1465700::plasmepsin VIII, putative	0.066	0.666	0.046	0.846	-1.304	-0.984	-0.954	-0.164	0.596	0.746	0.216	0.986	-0.764	0.406	-1.081	0.393	0.146	-1.487	0.357
PF3D7_082510(	PF3D7_0825100::ankyrin-repeat protein, putative	-0.090	0.200	-0.020	0.280	-0.240	-0.580	-0.560	0.210	0.290	0.490	0.030	0.150	-0.160	0.093	-0.460	0.330	0.007	-0.553	0.682
PF3D7_114110(	PF3D7_1141100::conserved Plasmodium protein, unknown function	0.125	0.385	-0.065	0.635	-0.365	-0.355	-0.345	0.045	0.525	0.175	-0.355	0.095	-0.505	0.270	-0.355	0.249	-0.255	-0.625	0.648
PF3D7_113950(	PF3D7_1139500::AAA family ATPase, putative	0.004	-0.121	-0.096	0.459	-0.271	-0.176	-0.231	0.114	0.474	0.529	-0.046	0.154	-0.796	0.062	-0.226	0.373	-0.229	-0.288	0.819
PF3D7_080470(	PF3D7_0804700::conserved Plasmodium protein, unknown function	0.023	0.063	0.043	0.043	-0.367	-0.217	-0.007	-0.047	0.613	0.413	-0.157	0.243	-0.647	0.043	-0.197	0.326	-0.187	-0.240	0.847
PF3D7_072680(	PF3D7_0726800::dolichyl-diphosphooligosaccharide--protein glycosyltrar	-0.052	0.188	-0.012	0.178	-0.222	-0.402	-0.212	-0.042	0.618	-0.162	-0.002	0.308	-0.192	0.076	-0.278	0.138	0.038	-0.354	0.782
PF3D7_053080(	PF3D7_0530800::CPW-WPC family protein	0.249	0.109	0.159	0.129	-0.461	-0.141	-0.091	0.189	0.599	-0.641	-0.231	0.449	-0.321	0.162	-0.231	0.049	-0.034	-0.393	0.762
PF3D7_142710(	PF3D7_1427100::lipase, putative	0.687	0.307	-0.163	-0.173	-0.443	-0.393	-0.213	-0.193	-0.313	0.757	-0.143	0.397	-0.113	0.164	-0.350	0.084	0.047	-0.514	0.700
PF3D7_114890(	PF3D7_1148900::Plasmodium exported protein, unknown function	0.680	0.430	-0.020	-0.010	-0.430	-0.230	-0.170	-0.120	-0.340	0.840	-0.290	-0.160	-0.180	0.270	-0.277	0.127	-0.210	-0.547	0.685
PF3D7_124450(	PF3D7_1244500::PIMMS57 protein	0.856	-0.104	0.126	-0.164	-0.344	-0.204	-0.334	0.066	-0.324	0.656	-0.194	0.086	-0.124	0.179	-0.294	0.133	-0.077	-0.473	0.721
PF3D7_083110(	PF3D7_0831100::surface-associated interspersed protein 8.1 (SURFIN 8	0.338	-0.032	0.198	0.008	-0.272	-0.172	-0.362	-0.022	-0.192	0.688	-0.092	0.028	-0.122	0.128	-0.268	0.158	-0.062	-0.397	0.760
PF3D7_100200(	PF3D7_1002000::Plasmodium exported protein (hyp2), unknown functio	0.747	0.577	-0.143	-0.153	-0.353	-0.403	-0.383	-0.223	0.067	0.447	0.037	0.057	-0.273	0.257	-0.380	0.097	-0.060	-0.637	0.643
PF3D7_110460(	PF3D7_1104600::radial spoke head protein, putative	0.316	0.076	0.046	0.056	-0.284	-0.424	0.036	-0.004	0.086	0.286	0.156	-0.014	-0.334	0.124	-0.224	0.123	-0.064	-0.348	0.786
PF3D7_140650(	PF3D7_1406500::WD repeat-containing protein 65, putative	-0.091	0.029	0.179	-0.031	-0.151	-0.311	-0.291	0.089	-0.081	1.019	-0.181	-0.231	0.049	0.022	-0.251	0.343	-0.121	-0.273	0.828
PF3D7_062550(	PF3D7_0625500::conserved Plasmodium membrane protein, unknown fu	-0.087	0.303	0.123	0.043	-0.337	-0.647	-0.677	-0.207	0.043	1.413	-0.137	0.023	0.143	0.096	-0.554	0.416	0.010	-0.649	0.638
PF3D7_112770(	PF3D7_1127700::conserved Plasmodium protein, unknown function	-0.035		0.065	-0.025	-0.255	-0.035	-0.295	-0.195	0.095	1.255	-0.475	-0.005	-0.095	0.002	-0.195	0.385	-0.192	-0.197	0.873
PF3D7_090260(	PF3D7_0902600::serine/threonine protein kinase, FIKK family	0.205	0.145	0.125	0.055	-0.275	-0.335	-0.335	-0.195	-0.085	1.225	-0.225	-0.115	-0.185	0.132	-0.315	0.315	-0.175	-0.448	0.733
PF3D7_121570(	PF3D7_1215700::conserved Plasmodium protein, unknown function	0.105	0.045	0.005	-0.145	-0.445	-0.465	-0.305	-0.115	0.205	0.955	-0.005	0.105	0.055	0.003	-0.405	0.349	0.052	-0.408	0.754
PF3D7_093170(	PF3D7_0931700::PIH1 domain-containing protein, putative	-0.005	-0.055	-0.005	0.005	-0.415	-0.275	-0.205	-0.215	0.315	0.945	0.095	-0.135	-0.045	-0.015	-0.299	0.348	-0.029	-0.283	0.822
PF3D7_113190(	PF3D7_1131900::conserved Plasmodium protein, unknown function	0.265	-0.165	-0.215	-0.315	-0.395	-0.695	-0.395	-0.325	-0.015	1.575	0.095	0.585	-0.005	-0.107	-0.495	0.412	0.225	-0.388	0.764
PF3D7_071410(	PF3D7_0714100::MAATS1 domain-containing protein, putative	-0.359	-0.359	-0.219	-0.039	-0.569	-0.479	-0.519	-0.289	0.191	1.861	-0.009	0.691	0.101	-0.244	-0.523	0.587	0.261	-0.278	0.825
PF3D7_040870(	PF3D7_0408700::perforin-like protein 1	-0.176	-0.246	0.034	-0.156	-0.546	-0.786	-0.436	-0.356	0.154	2.044	0.074	0.444	-0.046	-0.136	-0.589	0.614	0.157	-0.453	0.730
PF3D7_135400(	PF3D7_1354000::conserved Plasmodium protein, unknown function	-0.029	0.971	-0.269	-0.059	-0.549	-0.489	-0.319	-0.259	0.291	1.131	-0.329	0.141	-0.229	0.153	-0.453	0.387	-0.139	-0.606	0.657
PF3D7_060780(	PF3D7_0607800::GTP-binding protein, putative	-0.038	0.292	0.072	-0.118	-0.248	-0.258	-0.198	-0.058	0.072	0.462	-0.188	0.282	-0.078	0.052	-0.234	0.159	0.006	-0.287	0.820
PF3D7_053050(	PF3D7_0530500::conserved Plasmodium membrane protein, unknown fu	0.033	0.303	-0.057	0.073	-0.197	-0.187	-0.187	-0.177	-0.067	0.293	-0.027	0.193	0.003	0.088	-0.190	0.016	0.056	-0.278	0.825
PF3D7_040760(	PF3D7_0407600::conserved Plasmodium protein, unknown function	0.087	0.437	-0.203	-0.233	-0.363	-0.363	-0.573	-0.173	0.017	0.747	0.227	0.507	-0.113	0.022	-0.433	0.197	0.207	-0.455	0.730
PF3D7_142460(	PF3D7_1424600::small nucleolar RNA snoR28	0.235	0.165	0.165	0.105	-0.125	0.035	-0.185	0.085	-0.145	1.125	-0.495	-0.535	-0.435	0.168	-0.091	0.355	-0.488	-0.259	0.836
PF3D7_110920(	PF3D7_1109200::conserved Plasmodium protein, unknown function	0.209	0.089	0.249	0.069	-0.181	-0.061	-0.121	-0.161	-0.261	0.919	-0.141	-0.341	-0.271	0.154	-0.121	0.166	-0.251	-0.275	0.826
PF3D7_147610(	PF3D7_1476100::early transcribed membrane protein 14.2	0.107	0.567	0.197	0.147	-0.243	-0.503	-0.143	0.117	-0.113	0.827	-0.533	-0.193	-0.233	0.254	-0.296	0.277	-0.320	-0.551	0.683
PF3D7_122540(	PF3D7_1225400::rRNA-processing protein, putative	-0.062	0.338	0.328	0.118	-0.262	-0.522	-0.422	0.068	-0.002	0.908	-0.332	0.008	-0.172	0.181	-0.402	0.325	-0.165	-0.583	0.668
PF3D7_102950(	PF3D7_1029500::conserved Plasmodium protein, unknown function	0.023	0.563	0.383	0.093	-0.267	-0.417	-0.397	0.093	-0.117	0.783	-0.417	-0.137	-0.187	0.266	-0.360	0.253	-0.247	-0.626	0.648
PF3D7_060190(	PF3D7_0601900::conserved Plasmodium protein, unknown function	0.094	0.454	0.204	0.104	0.024	-0.406	-0.266	-0.046	-0.296	0.904	-0.136	-0.376	-0.256	0.214	-0.216	0.187	-0.256	-0.430	0.742
PF3D7_124490(	PF3D7_1244900::conserved Plasmodium protein, unknown function	0.072	0.512	0.012	-0.088	-0.158	-0.178	-0.258	-0.078	0.092	0.962	-0.288	-0.428	-0.178	0.127	-0.198	0.326	-0.298	-0.325	0.798
PF3D7_132440(	PF3D7_1324400::PRELI domain-containing protein, putative	0.158	0.688	0.048	-0.042	-0.242	-0.102	-0.122	-0.032	0.028	0.518	-0.332	-0.312	-0.262	0.213	-0.155	0.172	-0.302	-0.368	0.775
PF3D7_010640(	PF3D7_0106400::pre-rRNA-processing protein TSR2, putative	0.202	0.712	0.382	0.102	-0.098	-0.268	-0.188	-0.068	-0.188	1.032	-0.708	-0.528	-0.378	0.349	-0.185	0.258	-0.538	-0.534	0.691
PF3D7_146580(	PF3D7_1465800::dynein beta chain, putative	0.136	0.336	0.566	0.516	-0.694	-0.564	-0.424	-0.114	0.066	0.716	-0.244	-0.234	-0.064	0.389	-0.561	0.223	-0.181	-0.949	0.518
PF3D7_011420(	PF3D7_0114200::exported protein family 3	0.313	1.223	2.143	1.503	-1.157	-1.597	-1.007	0.213	0.373	1.883	-0.857	-1.427	-1.607	1.296	-1.254	0.823	-1.297	-2.549	0.171
PF3D7_022150(	PF3D7_0221500::Pfmc-2TM Maurer's cleft two transmembrane protein, p	-0.096	0.584	1.234	0.444	-1.046	-1.696	-1.266	1.994	0.524	2.274	-0.706	-1.106	-1.136	0.541	-1.336	1.597	-0.983	-1.878	0.272
PF3D7_102610(	PF3D7_1026100::conserved Plasmodium protein, unknown function	0.055	1.525	0.075	-0.115	-0.395	-0.305	-0.185	-0.475	-0.135	0.645	-0.305	-0.155	-0.235	0.385	-0.295	0.012	-0.231	-0.680	0.624
PF3D7_020180(	PF3D7_0201800::knob associated heat shock protein 40	0.083	0.533	0.093	0.123	-0.137	-0.357	-0.157	-0.067	-0.467	0.533	0.023	-0.287	0.083	0.208	-0.217	0.000	-0.060	-0.425	0.745
PF3D7_020170(	PF3D7_0201700::DnaJ protein, putative	0.065	0.615	-0.005	-0.025	-0.215	-0.385	-0.075	-0.135	-0.265	0.415	0.025	-0.095	0.075	0.163	-0.225	0.005	0.002	-0.388	0.764
PF3D7_020160(	PF3D7_0201600::PHISTb domain-containing RESA-like protein 1	0.161	0.631	0.101	0.051	-0.029	-0.229	-0.059	-0.089	-0.369	0.191	-0.069	-0.269	-0.019	0.236	-0.106	-0.089	-0.119	-0.342	0.789
PF3D7_100070(	PF3D7_1000700::Plasmodium exported protein (PHISTa), unknown func	-0.097		0.603	-0.047	-0.557	-0.357	-0.367	0.143	0.723	0.863	-1.087	-0.187	0.363	0.153	-0.427	0.577	-0.303	-0.580	0.669

Shown are the mean-centered $\log_2$ ratios of each sample against a 3D7 reference pool, the averaged $\log_2$ ratios for each parasite line grouped by PfCRT haplotype, and the fold change for $Dd2^{M343Lcrt}$ vs Dd2 lines.
