# Supplementary material for: Piperaquine-resistant PfCRT mutations differentially impact drug transport, hemoglobin catabolism and parasite physiology in Plasmodium falciparum asexual blood stages
Source: PLoS Pathog. 2022 Oct 28;18(10):e1010926. doi: 10.1371/journal.ppat.1010926 (PMC9645663; doi:10.1371/journal.ppat.1010926)
Supplement: S7 Table — Data show means ± SEM, calculated from four independent experiment with technical duplicates. Significance was determined using Mann-Whitney U tests. *p<0.05. MMV1557817 is also known as MIPS-1778. (PDF) [file ppat.1010926.s015.pdf]

S7 Table. *In vitro* 72 hr susceptibility of *pfcrt*-edited lines against hemoglobinase or aminopeptidase inhibitors.

	MMV1557817		E64		ALLN		Pepstatin	
Parasite Line	IC_{50} (nM)	IC_{90} (nM)	IC_{50} (nM)	IC_{90} (nM)	IC_{50} (nM)	IC_{90} (nM)	IC_{50} (nM)	IC_{90} (nM)
$Dd2^{Dd2crt}$	27.5 ± 1.2	68.4 ± 3.2	2349 ± 47.8	4468 ± 463.9	40.4 ± 1.7	256 ± 13.7	40071 ± 3102	91343 ± 6918
$Dd2^{F145Icrt}$	17.4 ± 2.5 *	57.1 ± 2.4	2535 ± 75.2	5320 ± 242.3	36.3 ± 1.9	250 ± 14.1	35830 ± 2500	80223 ± 7969
$Dd2^{M343Lcrt}$	18.3 ± 0.8 *	56.3 ± 2.9	2509 ± 38.4	5006 ± 253.1	45.3 ± 3.1	276 ± 14.4	42410 ± 3292	87920 ± 6995
$Dd2^{G353Vcrt}$	14.1 ± 1.2 *	50.6 ± 2.7	2523 ± 39.4	4960 ± 207	40.5 ± 2.1	301 ± 37.8	43887 ± 2860	89858 ± 6674
$Dd2^{3D7crt}$	26.4 ± 1.0	70.2 ± 3.6	2403 ± 64.8	4965 ± 206	45.3 ± 5.3	274 ± 19.9	41786 ± 2520	93596 ± 5993

Data show means ± SEM, calculated from four independent experiment with technical duplicates. Significance was determined using Mann-Whitney U tests. *p<0.05. MMV1557817 is also known as MIPS-1778.
